# DFT calculations bring insight to internal alkyne-to-vinylidene transformations at rhodium PNP- and PONOP-pincer complexes†

**DOI:** 10.1039/d0ra08764e

**Published:** 2021-03-23

**Authors:** Nasir A. Rajabi, Claire L. McMullin

**Affiliations:** Department of Chemistry, University of Bath Claverton Down Bath BA2 7AY UK N.A.Rajabi@gmail.com

## Abstract

Density Functional Theory (DFT) has been used to investigate the alkyne-to-vinylidene isomerisation reaction mediated by [Rh(PXNXP)]^+^ complexes (X = CH_2_: 2,6-bis(di-*tert*-butylphosphinomethyl)pyridine (PNP) and X = O: 2,6-bis(di-*tert*-butylphosphinito)pyridine (PONOP)) for terminal alkynes HC≡CR, where R = ^*t*^Bu and Ar′ (3,5-^*t*^Bu_2_C_6_H_3_). Calculations suggest the reaction mechanism proceeds *via* the slippage of π-bound alkyne at the Rh centre into a Rh–alkyne σ_C–H_ complex followed by an indirect 1,2-H shift to give the Rh–vinylidene species. NBO (Natural Bond Orbital) analysis of the transition states corresponding to the latter indirect 1,2-H shift step indicates that the migrating hydrogen atom exhibits protic character and hence, the basicity of the H-accepting centre (C_β_) is controlled by the substituents at that same atom and can tune the 1,2-H shift transition state. QTAIM (Quantum Theory of Atoms in Molecule) and NBO analyses of the Rh–vinylidene complexes indicate that these species exhibit a Rh ← C dative bond as well as π-back bonding from the Rh centre into the empty p_*z*_ orbital of the carbene centre (C_α_), showing the Rh–vinylidene complexes are Fischer type carbenes. Analysis of the alkyne and vinylidene complex HOMOs show that the equilibrium between the isomers can be tuned by the P–Rh–P bite angle of the [Rh(pincer)]^+^ fragment. Dictated by the nature of the pincer backbone, wider bite angles shift the equilibrium toward the formation of the Rh–vinylidene isomer (*e.g.*, X = CH_2_ and R = Ar′), while tighter bite angles shift the equilibrium more to the formation of the Rh–alkyne isomer (*e.g.*, X = O and R = Ar′).

## Abstract 2

Through DFT calculations, the equilibrium between Rh–alkyne and Rh–vinylidene species of PXNXP pincer ligated Rh cationic complexes is shown to be tuned by the P–Rh–P bite angle, which in turn is dictated by the nature of the X moiety of the pincer ligand.

## Graphical abstract

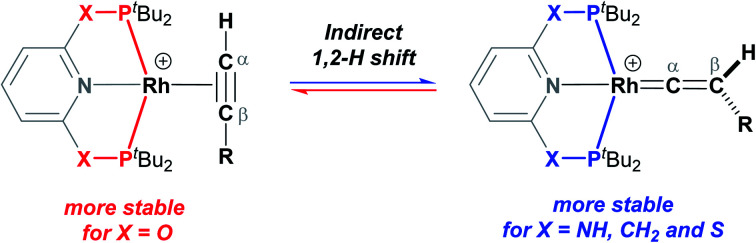

## Introduction

Transition metal (TM) vinylidene complexes are key species in organometallic chemistry.^1–9^ These compounds can be subjected to a variety of reactions such as hydrogenation, dehydrogenation and C–C bond formation processes.^10^ The most general strategy to form TM–vinylidene complexes under facile and simple conditions is the reaction of TM complexes with alkynes. This process can lead to the coordination of alkynes to a metal centre to give η^2^-alkyne complexes, which are stable enough to be characterised experimentally^11–17^ or can act as transient intermediates that undergo C–R (R = C_6_H_5_, Ar′ and H) bond cleavage and migration of the R group to yield TM–vinylidene complexes.^18–31^ Such reactivity can be feasibly modulated by factors such as changing the nature of alkynes or the transition metal.

**Fig. 1:**
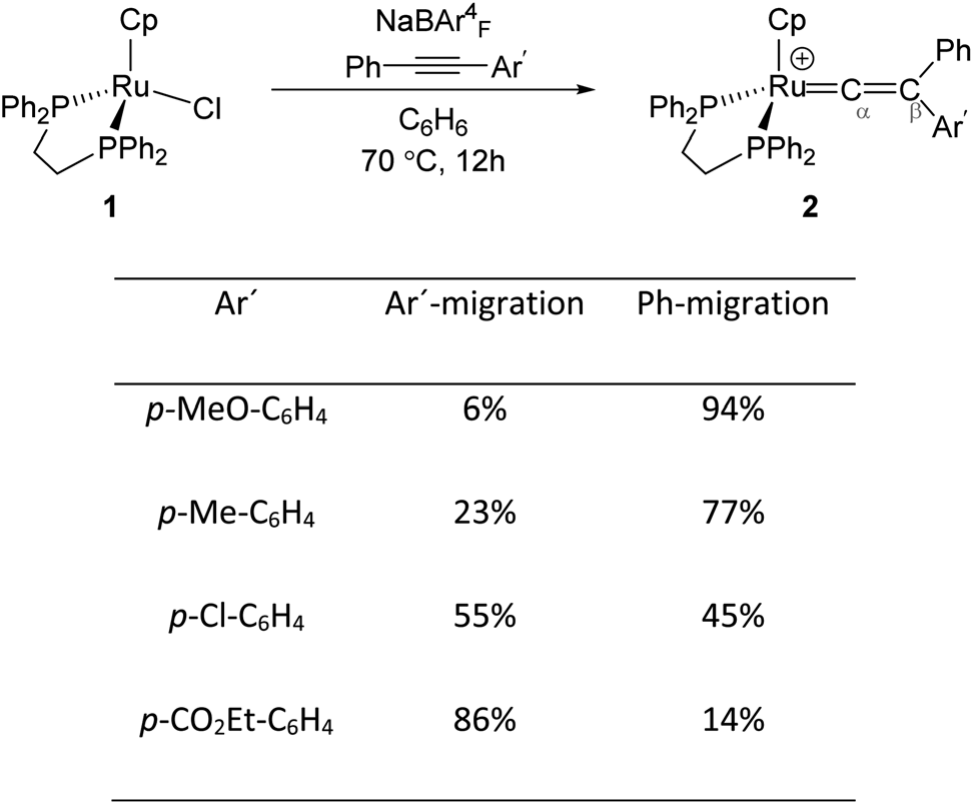
Reaction of the Ru-precursor 1 with internal alkyne substrates PhC≡CAr′ to give the Ru–vinylidene species 2.^16^

Mutoh, Ishii and co-workers described the reaction of [CpRu(dppe)]^+^ (1) (dppe = 1,2-diphenylphosphinoethane) with internal alkynes (PhC≡CAr′) and showed that both aryl substituents undergo the 1,2-migration process to generate the disubstituted Ru–vinylidene species 2 (Fig. 1).^16,32^ They demonstrated that groups featuring electron-donating substituents decrease the relative migratory aptitude of the Ar′ group over Ph. However, the migratory aptitude becomes opposite when Ar′ groups have electron-withdrawing substituents. Based on these results, the authors suggested that this is due to the stabilisation of the negative charge on the Ar′ group in the 1,2-Ar′ shift transition state, suggesting the 1,2-Ar′ migration proceeds *via* an electrophilic mechanism (Fig. 1).^16^ DFT (Density Functional Theory) studies by Tsuchida, Takano and co-workers on internal alkyne transformations at [CpRu((C_6_H_5_)C≡CC_6_H_4_R-p)(dppe)]^+^ (R = OMe, CO_2_Et) into the corresponding vinylidene complexes shows that this reaction proceeds with a direct 1,2-migration mechanism.^33^ In accordance with the experimental findings of Ishii *et al.*, Tsuchida, Takano and co-workers showed that alkyne substrates with an electron-donating substituent (R = OMe) favours the Ph-migration over the Ar′-migration reaction. However, using NBO (Natural Bond Orbital) donor–acceptor interaction analysis, they stated that this reaction proceeds *via* a nucleophilic mechanism; as in the 1,2-Ar′/Ph migration process, the electron-donating substituted Ar′ group stabilises the positive charge of the accepting carbon centre (C_β_), which facilitates the 1,2-Ph migration process.

**Scheme 1:**
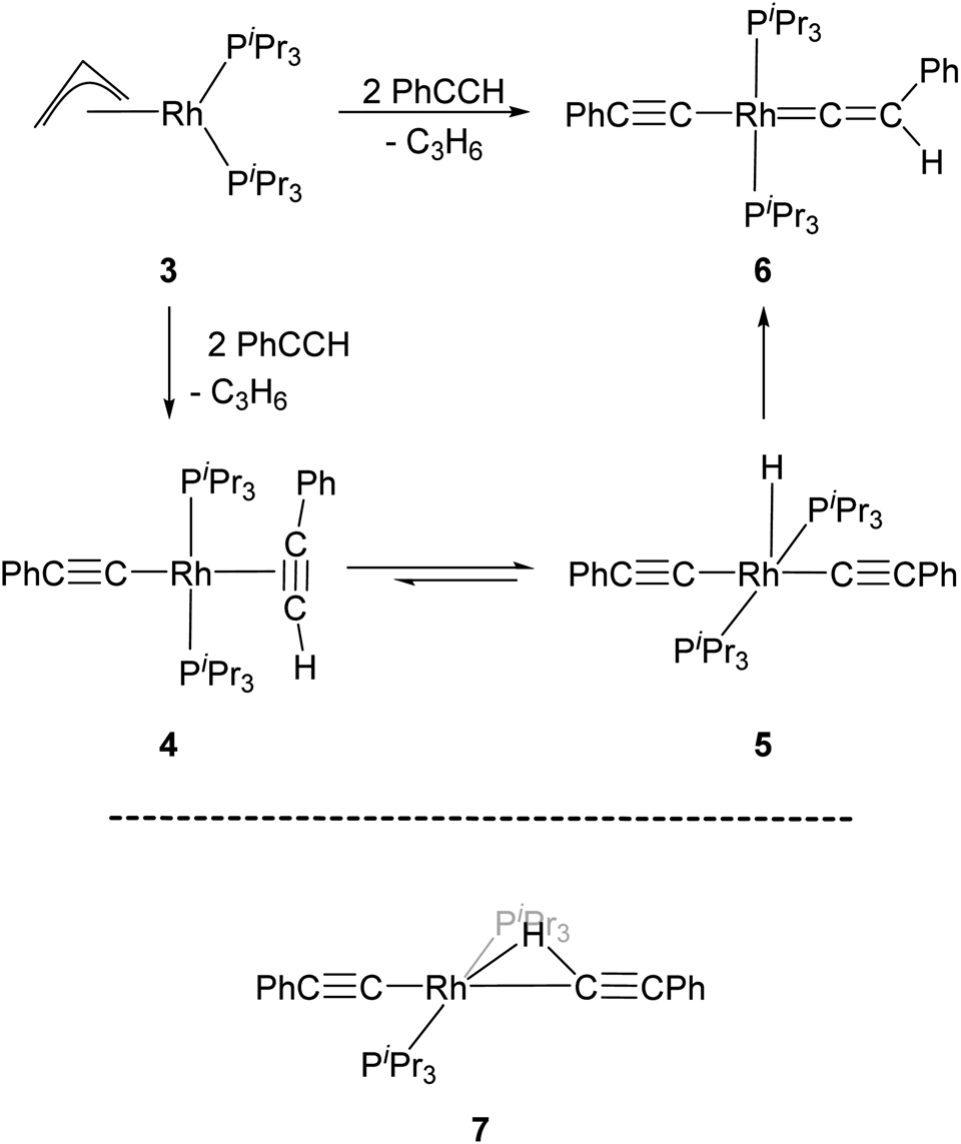
Reaction scheme for the formation of the Rh–vinylidene species 6,^34^ and the DFT computed Rh–H alkenyl intermediate 7 featuring a bridging hydrogen between the Rh and C centres.^35^

Werner and co-workers proposed the reaction of [(η^3^-C_3_H_5_)Rh(P^*i*^Pr_3_)_2_] (3) with terminal alkynes HC≡CR (R = H, Me, ^*t*^Bu and Ph) eliminates C_3_H_6_ and yields the Rh–alkyne species 4, which undergoes a C–H bond oxidative cleavage at the Rh centre to form the Rh–H species 5 (Scheme 1).^34^ Complex 5 then undergoes a further transformation and gives the Rh–vinylidene species, 6. A DFT study by Hall and co-workers featured these reaction systems and shows that oxidative cleavage of the first HC≡CR substrate (R = Ph) followed by a C–H reductive elimination at the Rh centre releases propene and forms a Rh-alkenyl intermediate.^35^ Addition of a second HC≡CR substrate to the Rh-alkenyl intermediate forms the bridging hydrogen intermediate 7 (Scheme 1), through which the H-migration proceeds with a 1,2-H shift mechanism to give [(PhC≡C)Rh(P^*i*^Pr_3_)_2_(C=C(Ph)(H)] (6).

A DFT study by Angelis and co-workers on the isomerisation of [(Cp)(PMe_3_)_2_Ru(HC≡CR)]^+^ to the vinylidene isomer shows that this reaction proceeds *via* an indirect 1,2-H shift reaction.^36^ The second possible pathway to from the vinylidene isomer is the C–H oxidative cleavage of the alkyne at the Ru centre followed by a 1,3-H migration reaction to give the Ru–vinylidene complex. However, this was reported to be kinetically inaccessible, as the energy barrier of the 1,3-H shift reaction is significantly higher than the 1,2-H shift pathway. Later on, the same authors demonstrated that upon increasing the electron-richness of the Ru centre, the oxidative cleavage product can be both kinetically and thermodynamically accessible.^37^ Particularly notable is the joint experimental and computational study by Lynam, Fey and co-workers on factors such as the nature of substituents, the metal and the ligands, which can control the thermodynamic preference of metal–vinylidene isomers over the corresponding metal–alkyne isomers.^38^ The authors proposed a protocol to design ideal conditions to stabilise metal–vinylidene isomers. For instance in [RuCl_2_(PR′_3_)(=C=CHR)], with electron-withdrawing substituents (R) and phosphine ligands with electron-rich groups (R′), formation of the Ru–vinylidene isomer is thermodynamically favoured over the Ru–alkyne isomer.

**Scheme 2:**
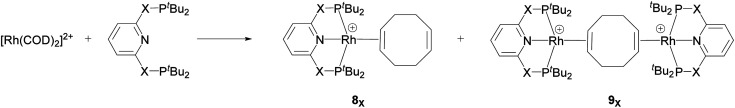
Reaction of the pincer ligand with the dimeric Rh precursor, [Rh(COD)_2_]^2+^, to form 8_X_ and 9_X_. The counter ions (BAr^F^_4_) are omitted for clarity.^39^

Recently, Chaplin and co-workers described the substitution reaction in [Rh(COD)_2_][BAr^F^_4_] with the pincer ligands PXNXP (COD = 1,5-cyclooctadiene, Ar^F^ = 3,5-(CF_3_)_2_C_6_H_3_)) (X = CH_2_: 2,6-bis(di-*tert*-butylphosphinomethyl)pyridine (PNP) and X = O: 2,6-bis(di-*tert*-butylphosphinito)pyridine (PONOP)) which leads to the formation of the corresponding PNP and PONOP-pincer complexes 8_X_ and 9_X_.^39^ As shown in Scheme 2, this reaction gives the monomeric complex 8_X_ ([Rh(PXNXP)(η^2^-COD)][BAr^F^_4_]) as the major product and the dimeric complex 9_X_ ([{Rh(PXNXP)}_2_(μ-η^2^:η^2^-COD)][BAr^F^_4_]_2_) as the minor product.

**Scheme 3:**
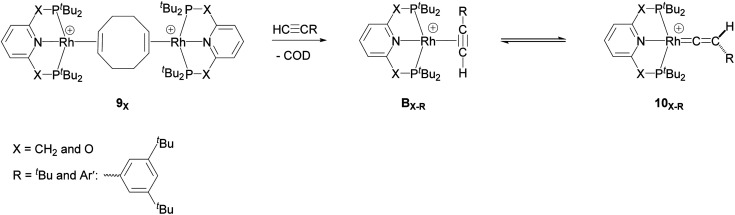
Reaction of the dimeric {Rh(PNP)}^2+^ complex 9_X_ with terminal alkynes HC≡CR (R = ^*t*^Bu and Ar′ (3,5-^*t*^Bu_2_C_6_H_3_)) to form the Rh–alkyne (B_X–R_) and the Rh–vinylidene complex (10_X–R_). The counter ions (BAr^F^_4_) are omitted for clarity.^39^

They demonstrated that reaction of the dimeric and monomeric Rh complexes 8_X_ and 9_X_ with L-type ligands such as CO results in the formation of the Rh–CO adduct, inferring the COD ligand dissociation from the Rh species forms the cationic 14e^−^ {Rh(pincer)}^+^ species, which can be trapped out by CO to form the adduct. The authors also explored the reactivity of the active species, {Rh(pincer)}^+^, by the reaction of the dimeric complex 9_X_ with HC≡CR (R = ^*t*^Bu and Ar′ (3,5-^*t*^Bu_2_C_6_H_3_)) in 1,2-difluorobenzene (DFB) solvent at room temperature which gives the Rh–vinylidene complex 10_X–R_ (Scheme 3).^39^ With the dimeric {Rh(PNP)}^2+^, this process was found to be very fast (*ca*. five minutes) whereas, with the dimeric {Rh(PONOP)}^+^, it took significantly longer (*ca*. 18 h). It should be noted that with the dimeric {Rh(PONOP)}^2+^ and R = Ar′, this process forms the Rh–alkyne as the major product and the Rh–vinylidene complex as the minor product (70% *vs.* 30%). This suggests that the alkyne-to-vinylidene transformation process is reversible, a process that is also seen at [RuCl(η^5^-C_9_H_7_)(PPh_3_)_2_]^17^ and [CpRu(dppe)]^+^ complexes.^32^

Herein, DFT calculations were carried out to rationalise the mechanism of the formation of these Rh–vinylidene complexes, to characterise the nature of the Rh–carbene bond in the Rh–vinylidene complexes and to understand factors that can affect the equilibrium between the Rh–alkyne and Rh–vinylidene isomers.

## Computational methodology

Gas phase DFT calculations were run with Gaussian 09 (Revision D.01).^40^ The Rh and P centres were described with the Stuttgart RECPs and associated basis sets,^41^ and the 6-31G** basis set was used for all other atoms (BS1).^42^ A polarization function was also added to P (*ζ*_d_ = 0.387) and S (*ζ*_d_ = 0.503) atoms. Initial BP86 (ref. 43 and 44) optimizations were performed using the ‘grid = ultrafine’ option, with all stationary points being fully characterized *via* analytical frequency calculations as either minima (all positive eigenvalues) or transition states (one negative eigenvalue). IRC (Intrinsic Reaction Coordinate) calculations and subsequent geometry optimizations were used to confirm the minima are linked by the isolated transition state. All energies were recomputed with a larger basis set featuring cc-pVTZ-PP for Rh, cc-pVTZ-dk for S and the 6-311++G** basis set for all other atoms (BS2). 1,2-Difluorobenzene is not a parameter defined solvent in the Gaussian program. To overcome this its dielectric constant (*ε* = 13.4) was specified when applying a polarizable continuum model solvent correction with BS1.^45^ It should be noted that similar trends to the 1,2-difluorobenzene results were obtained when using fluorobenzene (*ε* = 5.42, see ESI† for more details) as the solvation medium. Additional single-point dispersion corrections to the BP86 results employed Grimme's D3 parameter set with Becke–Johnson damping as implemented in Gaussian 09.^46^ QTAIM (Quantum Theory of Atoms in Molecules)^47^ and NBO (Natural Bonding Orbital)^48^ were performed on the BP86-optimised geometries of the Rh–vinylidene complexes 10_X–R_ to characterise the nature of the Rh–carbene bond. The complete computational approach is described in shorthand as BP86-D3BJ (BS2,1,2-difluorobenzene)//BP86(BS1). All free energies are reported in kcal mol^−1^ and are given relative to 9_X_.

## Results and discussion

**Scheme 4:**
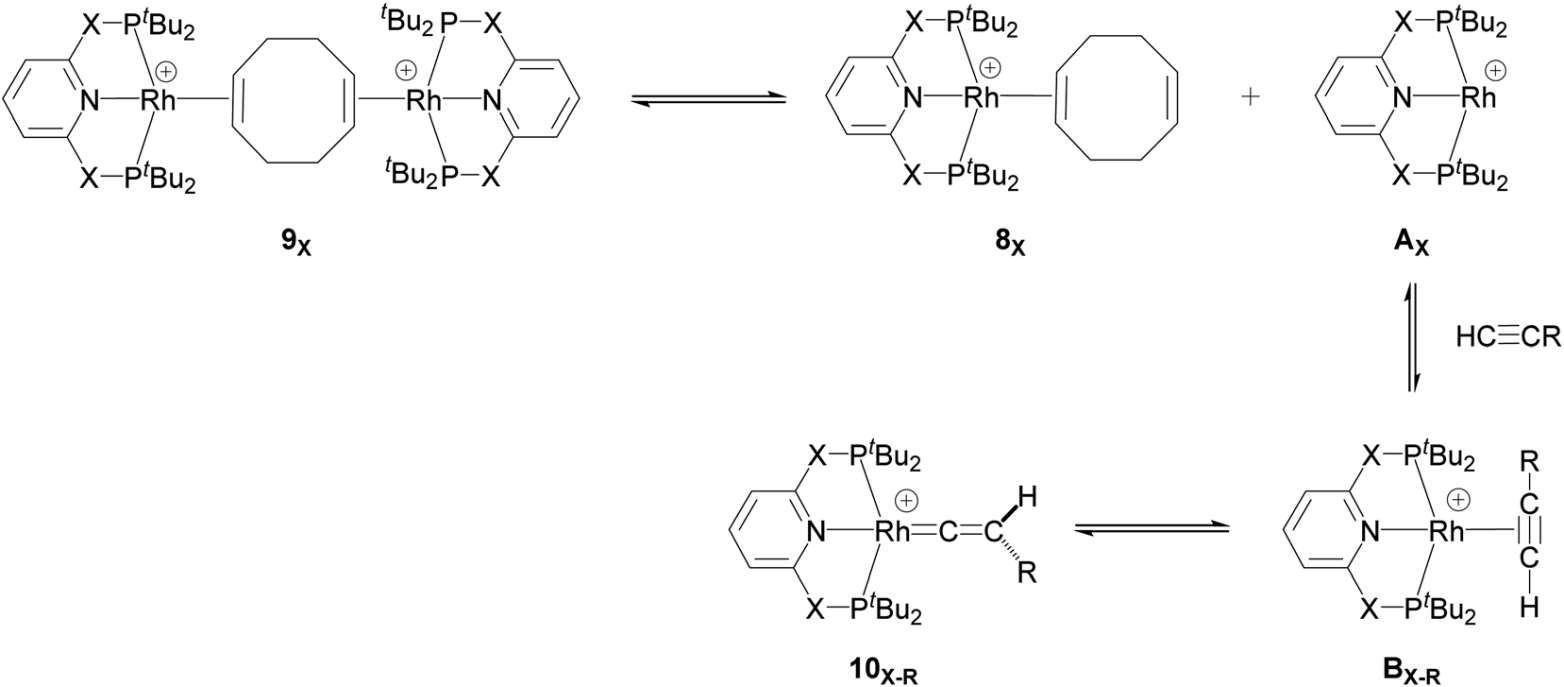
Formation of the 14e^−^ Rh species A_X_ and its onward reaction with the alkyne substrates to form the Rh–alkyne (B_X–R_) and Rh–vinylidene species (10_X–R_).

Our computed mechanism starts with the splitting of 9_X_ (as reported by Chaplin and co-workers^39^) to give the 14e^−^ Rh species A_X_ (Scheme 4, X = CH_2_ and O, R = ^*t*^Bu and Ar′). This process is endergonic by Δ*G* = +20.0 kcal mol^−1^ for X = CH_2_ and Δ*G* = +23.0 kcal mol^−1^ for X = O. The cationic Rh moiety can be stabilised by the coordination of HC≡CR to A_X_ in a π-binding mode to the Rh centre to give the Rh–alkyne species B_X–R_. For X = CH_2_ and O and R = ^*t*^Bu, B_X_–*t*_Bu_ lies at a free energy of −0.2 and −3.7 kcal mol^−1^ respectively, showing formation of the PONOP complex (B_O–_*_t_*_Bu_) is energetically favoured by 3.5 kcal mol^−1^. Coordination of HC≡CAr′ to the Rh centre significantly stabilises the cationic Rh species to form B_X–Ar_ at Δ*G* = −12.6 kcal mol^−1^ for X = CH_2_ and Δ*G* = −11.0 kcal mol^−1^ for X = O.

**Scheme 5:**
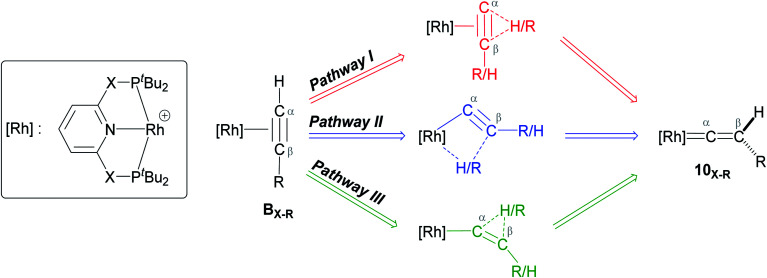
Transition states for three potential mechanisms (Pathways I, II and III) to form the Rh–vinylidene complex 10_X–R_.

As shown in Scheme 5, once the Rh–alkyne π-complex B_X–R_ has formed, it can undergo an intra-molecular transformation *via* three possible pathways (I, II and III) to afford the Rh–vinylidene complex 10_X–R_. In Pathway I, the Rh–alkyne species B_X–R_ undergoes a direct 1,2-R/H migration to form 10_X–R_. In Pathway II, transfer of the R/H groups onto the Rh centre occurs *via* a four-member transition structure to yield 10_X–R_. Pathway III proceeds with the initial formation of the Rh–C bond that induces the 1,2-H/R shift reaction simultaneously to give 10_X–R_.

Both the ^*t*^Bu and Ar′ alkyne substituents could migrate from the C_β_ centre to the C_α_ centre from B_X–R_ to form 10_X–R_. However, the energy barriers for migration of Ar′ or ^*t*^Bu groups *via* the pathways shown in Scheme 3 are computed to be too high (between 37 to 58 kcal mol^−1^)^49^ and hence, unsurmountable under the experimental reaction conditions of 9_X_ and HC≡CR^39^ (see ESI, Tables S2 and S3†).

The most obvious route to form the Rh–vinylidene species involves H migration. In Pathway I (Table 1) for the PNP-pincer ligand and R = ^*t*^Bu, the direct 1,2-H shift reaction in B_C–_*_t_*_Bu_ proceeds *via*TS(B-10)_C–_*_t_*_Bu_ at Δ*G*^‡^ = +40.1 kcal mol^−1^ to afford the Rh–vinylidene species 10_C–_*_t_*_Bu_ at Δ*G* = −12.1 kcal mol^−1^. The free energy barrier for this process (
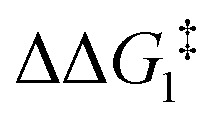
) is computed to be 40.3 kcal mol^−1^ (relative to B_C–_*_t_*_Bu_), which is too high to be accessible under the reaction conditions. Changing the pincer ligand from PNP to PONOP slightly lowers the free energy (by 0.7 kcal mol^−1^) of the transition state corresponding to the 1,2-H shift process (TS(B-10)_O–_*_t_*_Bu_) to +39.4 kcal mol^−1^ to give the Rh–vinylidene species 10_O–_*_t_*_Bu_ at −8.9 kcal mol^−1^. The free energy barrier for this process is still too high (
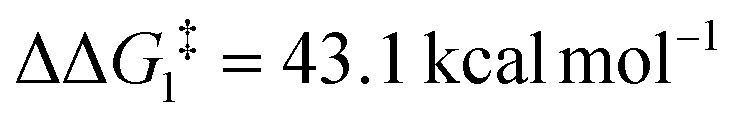
), going from R = ^*t*^Bu to R = Ar′, with the PNP-pincer ligand the transition state TS(B-10)_C–Ar_ lies at 
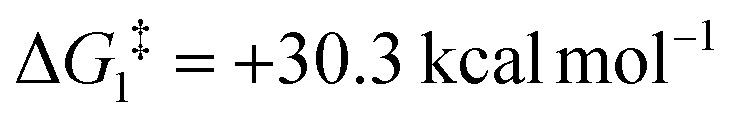
; significantly lower than when R = ^*t*^Bu. With respect to B_O–Ar_, the free energy barrier for this process is computed to be 42.9 kcal mol^−1^ – again too high and not accessible at room temperature. A similar trend can be seen for the PONOP-pincer ligand when R = Ar′ (
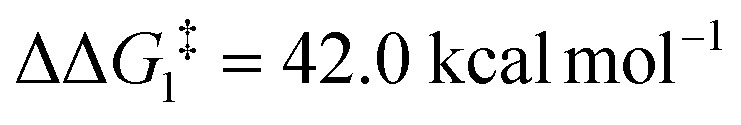
). Therefore, the high energy barriers associated with the direct 1,2-H shift reaction in Pathway I are not in agreement with experiment.

**Table 1:** DFT calculated free energies^a^ (kcal mol^−1^) for the transformation of the Rh–alkyne species B_X–R_ to the Rh–vinylidene species 10_X–R_*via* Pathway I

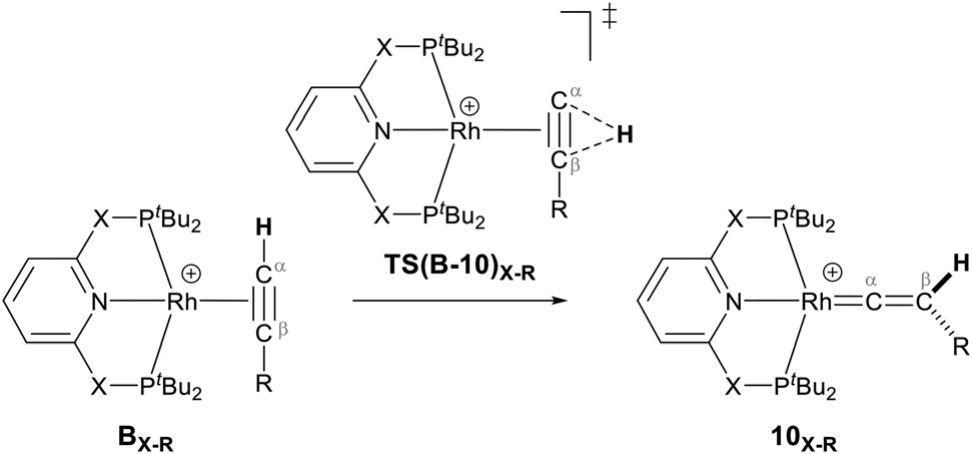					
X	R	B_X–R_	TS(B-10)_X_–_R_	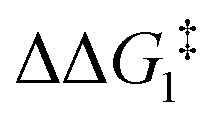	10_X–R_
CH_2_	^*t*^Bu	−0.2	+40.1	40.3	−12.1
O	^*t*^Bu	−3.7	+39.4	43.1	−8.9
CH_2_	Ar′	−12.6	+30.3	42.9	−15.7
O	Ar′	−11.0	+31.0	42.0	−10.9

a DFT method = BP86-D3(BJ)-1,2-difluorobenzene/BS2//BP86/BS1. Computed energy barrier (
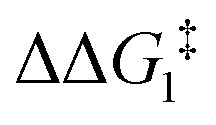
) is relative to B_X–R_. All free energies are quoted relative to 9_X_ (X = CH_2_ and O, R = ^*t*^Bu and Ar′).

For Pathway II (Table 2), B_C–_*_t_*_Bu_ undergoes slippage of the π-coordination mode to a σ_C–H_ coordination mode *via*TS(B-C)_C–_*_t_*_Bu_ at 
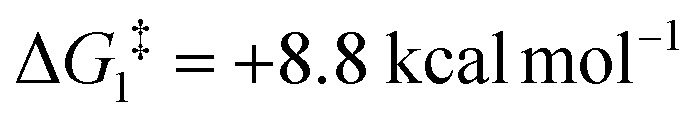
 to give C_C–_*_t_*_Bu_, featuring an elongated C_α_–H bond (1.22 Å) to give a short Rh⋯H of 1.73 Å. NBO analysis of C_C–_*_t_*_Bu_ indicates a donor–acceptor interaction between the C_α_–H bonding orbital and a vacant Rh orbital with stabilisation energy (Δ*E*^(2)^) of 82.9 kcal mol^−1^, showing a C_α_–H agostic interaction with the Rh centre (see ESI, Fig. S3† for details). With respect to B_C–_*_t_*_Bu_, formation of C_C–_*_t_*_Bu_ proceeds with a free energy barrier (
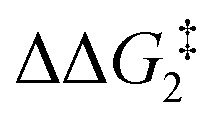
) of 9.0 kcal mol^−1^ and is energetically downhill by 2.8 kcal mol^−1^. C_C–_*_t_*_Bu_ can undergo the H-transfer into the bridging position between the C_β_ and Rh centre *via*TS(C-D)_C–_*_t_*_Bu_ at +37.7 kcal mol^−1^ to give the four-membered intermediate D_C–_*_t_*_Bu_ at Δ*G* = +36.4 kcal mol^−1^.

**Table 2:** DFT calculated free energies^c^ (kcal mol^−1^) for the transformation of the Rh–alkyne species B_X–R_ to the Rh–vinylidene species 10_X–R_*via* Pathway II

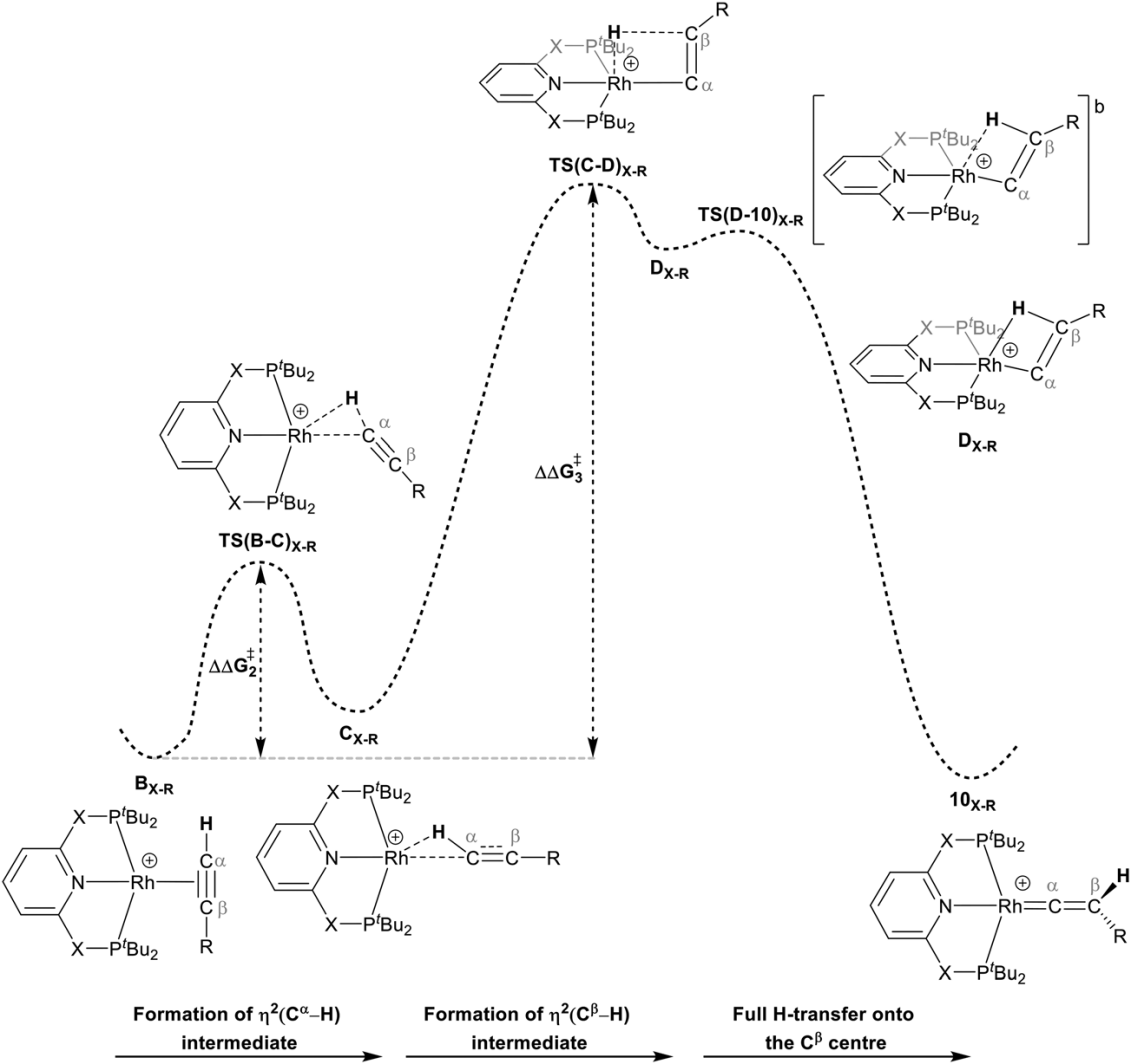								
X	R	TS(B-C)_X–R_	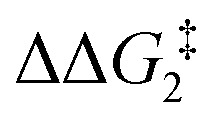	C_X–R_	TS(C-D)_X–R_	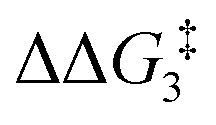	D_X–R_	10_X–R_
CH_2_	^*t*^Bu	+8.8	9.0	−3.0	+37.7	37.9	+36.4	−12.1
O	^*t*^Bu	+6.5	10.2	+1.3	+38.5	42.2	+37.0	−8.9
CH_2_	Ar′	+0.2	12.8	−4.5	+29.8^a^	42.4^a^	—b	−15.7
O	Ar′	+2.8	13.8	−1.0	+31.6^a^	42.6^a^	—b	−10.9

a Estimated free energies by freezing the key distances in TS(C-D)_X–Ar_.

b Transition state is not calculated.

c DFT method = BP86-D3(BJ)-1,2-difluorobenzene/BS2//BP86/BS1. Computed energy barriers (
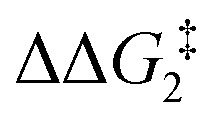
 and 
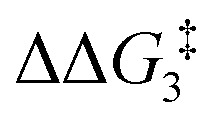
) are relative to B_X–R_. All free energies are quoted relative to 8_X_ (X = CH_2_ and O, R = ^*t*^Bu and Ar′).

The H atom then fully transfers onto the C_β_ centre to give 10_C–_*_t_*_Bu_. Attempts to locate the transition state (TS(D-10)_X–R_) corresponding to this process were inconclusive as it was a very flat potential free energy surface. A systematic increase of the Rh–H distance revealed that the energy barrier for this process is approximately 1.0 kcal mol^−1^ (relative to D_C–_*_t_*_Bu_). Thus, formation of 10_C–_*_t_*_Bu_*via* Pathway II is a high energy-process that requires a free energy barrier (
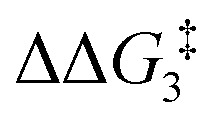
) of 37.9 kcal mol^−1^ (relative to B_C–_*_t_*_Bu_). Likewise, for the PONOP ligand and R = ^*t*^Bu formation of the σ_C–H_ intermediate C_O–_*_t_*_Bu_ (C_α_–H: 1.21 Å and Rh⋯H: 1.75 Å, Δ*E*^(2)^ = 72.2 kcal mol^−1^) proceeds with a low free energy barrier (
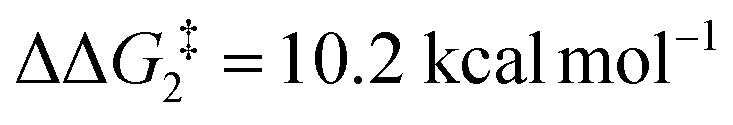
). However, this process is energetically uphill by 5.0 kcal mol^−1^, suggesting it can be reversed to reform the Rh–alkyne species B_O–_*_t_*_Bu_. The H transfer into the bridging position between the Rh and C_β_ centres occurs *via*TS(C-D)_O–_*_t_*_Bu_ at Δ*G*^‡^ = +38.5 kcal mol^−1^ to give D_O–_*_t_*_Bu_ at Δ*G* = +37.0 kcal mol^−1^. A very flat free energy surface sees the full H-transfer onto the C_β_ centre to give 10_O–_*_t_*_Bu_ at Δ*G* = −8.9 kcal mol^−1^. Thus, for Pathway II, formation of the Rh–vinylidene species 10_O–_*_t_*_Bu_ requires a high free energy barrier of 42.2 kcal mol^−1^. For R = Ar′ and the PNP-pincer ligand, the transition state (TS(C-D)_C–Ar_) corresponding to the formation of the intermediate C_C–Ar_ (C_α_–H: 1.19 Å and Rh⋯H: 1.78 Å, Δ*E*^(2)^ = 73.9 kcal mol^−1^) has a free energy of 12.8 kcal mol^−1^ – higher than when R = ^*t*^Bu and computed to be energetically uphill by 8.1 kcal mol^−1^. Unfortunately, all attempts to locate the four-member transition state (TS(C-D)_X–Ar_) corresponding to the H transfer onto the C_β_ centre and the subsequent intermediate D_X–Ar_ remained allusive. Thus, the transition state free energy value was estimated by freezing the corresponding bond distances and TS(C-D)_C–Ar_ was calculated to be approximately Δ*G*^‡^ = +29.8 kcal mol^−1^,^50^ with a free energy barrier of 42.4 kcal mol^−1^ that again is too high to be accessible under the reaction conditions. In comparison to the PNP ligand, the PONOP intermediate C_O–Ar_ (C_α_–H: 1.18 Å and Rh⋯H: 1.82 Å, Δ*E*^(2)^ = 67.1 kcal mol^−1^) formed *via* a slightly higher energy barrier (
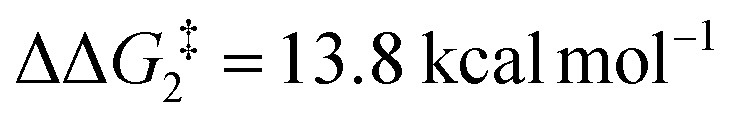
). This process is computed to be more energetically uphill (by 1.9 kcal mol^−1^). The H transfer process onto the C_β_ occurs with a high free energy barrier of +42.6 kcal mol^−1^ to form the Rh–vinylidene species. Thus, as outlined above, although formation of the σ_C–H_ intermediate C_X–R_ occur with low free energy barriers, the hydrogen transfer onto the C_β_ involves a large free energy barrier, which is not accessible under reaction conditions.

The indirect 1,2-H shift reaction *via* Pathway III involves the Rh sigma C–H complex C_X–R_. As shown in Table 3, in C_X–R_, the H centre can either transfer to the Rh centre to give E_X–R_ or it can transfer onto the C_β_ centre to form 10_X–R_. The H-transfer onto the Rh centre occurs with a C–H oxidative cleavage at the Rh centre *via*TS(C-E)_X–R_ to form the terminal Rh(iii)–H species E_X–R_. As listed in Table 3, in all cases, the H-transfer process to the Rh centre proceeds with a very flat free energy surface, an essentially barrierless process to give E_X–R_, suggesting a facile reaction and importantly consistent with the findings reported by Hall and co-workers.^35^ With respect to C_X–R_, formation of the terminal Rh–H species E_X–R_ is slightly exergonic. However, E_X–R_ is considerably less thermodynamically favoured than the experimentally observed Rh–vinylidene species 10_X–R_ (by *x* kcal mol^−1^).

**Table 3:** DFT calculated free energies^a^ (kcal mol^−1^) for the transformation of the Rh–alkyne species B_X–R_ to the Rh–vinylidene species 10_X–R_ (*via* Pathway III) and the Rh–H species E_X–R_

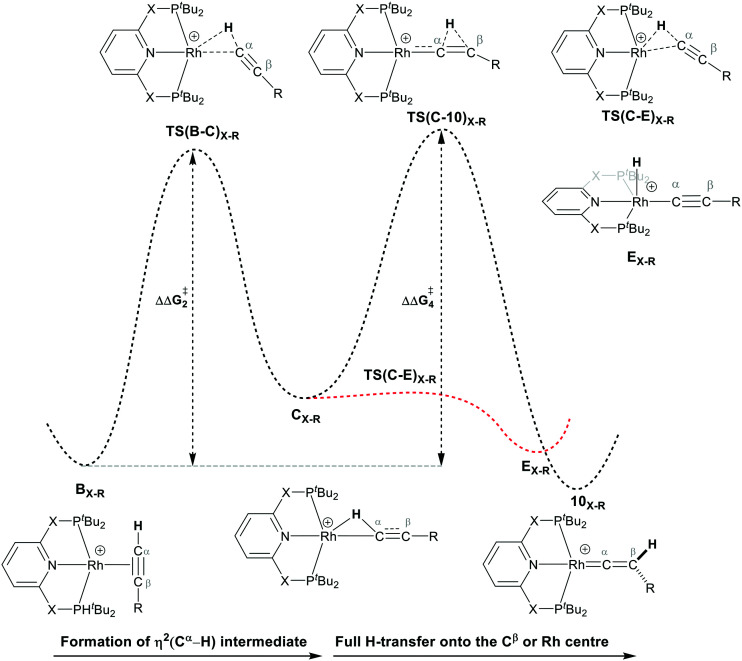							
X	R	TS(C-10)_X–R_	C_X–R_	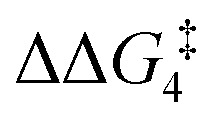	10_X–R_	TS(C-E)_X–R_	E_X–R_
CH_2_	^*t*^Bu	+8.7	−3.0	8.9	−12.1	−2.8	−8.1
O	^*t*^Bu	+10.2	+1.3	13.9	−8.9	+0.8	−0.6
CH_2_	Ar′	+7.6	−4.5	20.2	−15.7	−5.5	−11.4
O	Ar′	+9.1	−1.0	20.1	−10.9	−1.2	−4.4

a DFT method = BP86-D3(BJ)-1,2-difluorobenzene/BS2//BP86/BS1. Computed energy barrier (
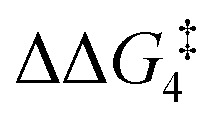
) is relative to B_X–R_. All free energies are quoted relative to 9_X_ (X = CH_2_ and O, R = ^*t*^Bu and Ar′).

For the H transfer process onto the C_β_ centre *via*C_X–R_, with R = ^*t*^Bu and the PNP-pincer ligand, the bridging hydrogen indirectly can transfer onto the C_β_ centre *via*TS(C-10)_C–_*_t_*_Bu_, which lies at Δ*G*^‡^ = +8.7 kcal mol^−1^ to generate 10_C–_*_t_*_Bu_. Interestingly, with respect to B_C–_*_t_*_Bu_, the free energy barrier for this process (
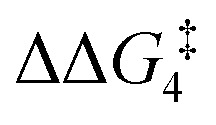
) is computed to be only 8.9 kcal mol^−1^, significantly lower than those in Pathways I and II. With the PONOP-pincer ligand, the indirect 1,2-H shift process proceeds with a free energy barrier of 13.9 kcal mol^−1^, which is 4.0 kcal mol^−1^ higher than with the PNP-pincer ligand. The free energy barriers of the indirect 1,2-H migration process for the aryl-substituted alkyne with the PNP or the PONOP-pincer ligands increase to 20.2 kcal mol^−1^ and 20.1 kcal mol^−1^ respectively. However, they are significantly lower than those in Pathways I and II and values would be accessible at room temperature. Thus, Pathway III exhibits the energetically viable mechanism, consistent with the facile formation of Rh vinylidene species 10_X–R_ seen experimentally.

It should be noted that the Rh–H species E_X–R_ may undergo a reverse process *via* low free energy barriers (1.4–5.9 kcal mol^−1^) to reform C_X–R_ and then follow the H-transfer reaction onto the C_β_ centre to generate 10_X–R_ which is more stable than the Rh–H species E_X–R_. For R = ^*t*^Bu and X = CH_2_, the rate-determining step (RDS) is the indirect 1,2-H shift (TS(C-10)_X–R_) while for other systems, RDS is the formation of the 14e^−^ Rh intermediate A_X–_*_t_*_Bu_.

### Hydrogen character in the indirect 1,2-H shift reaction: protic or hydridic?

We have shown that formation of the Rh–vinylidene species proceeds *via* Pathway III, in which 1,2-H migration is a key step. However, it is not clear whether in the H-migration process, the H centre migrates as a proton or hydride (*i.e.*, *via* an electrophilic or a nucleophilic mechanism respectively). In this regard, as listed in Table 4, both TS(C-10)_C–_*_t_*_Bu_ and TS(C-10)_O–_*_t_*_Bu_ exhibit a shorter C_α_⋯H distance than C_β_⋯H by 0.36 Å and 0.35 Å respectively. This shows that the H centre exhibits a greater tendency to interact with the C_α_ centre than with the C_β_ centre. A similar trend can also be seen in TS(C-10)_C–Ar_ and TS(C-10)_O–Ar_ (0.19 Å and 0.12 Å respectively). However, for R = Ar′ the H centre has a considerably shorter distance to C_β_, showing a stronger interaction between the H and C_β_ centres when R = Ar′. NBO charge analysis of the transition state TS(C-10)_X–R_ shows that for R = ^*t*^Bu, the Rh centre exhibits a very similar negative charge value (*q*_Rh_ = −0.54 to −0.55), implying the nature of the X moiety in the pincer ligands has a negligible effect on the charge of the Rh centre. For R = Ar′, the Rh centre features a slightly more negative charge (*q*_Rh_ = −0.64 to −0.66) than when R = ^*t*^Bu, which can be attributed to the π-electrons of the aryl group of the alkyne. In all cases, the H centre features similar positive charges (*q*_H_ = +0.39 to +0.40), implying the H centre exhibits protic character, showing it can transfer *via* an electrophilic mechanism. For R = ^*t*^Bu, the C_β_ centre displays small positive charges, while for R = Ar′, the C_β_ centre possesses negative charges, indicating the electronic character of the C_β_ centre is sensitive to the nature of the R substituent. Interestingly, as the charge of the C_β_ centre becomes slightly negative (*e.g.*, R = Ar′), the transition state TS(C-10)_X–R_ lies at a lower free energy (by 1.1 kcal mol^−1^ for both PNP and PONOP relative to R = ^*t*^Bu), whereas, when the C_β_ has a positive charge (*e.g.*, R = ^*t*^Bu), the transition state TS(C-10)_X–R_ lies at a higher free energy. This suggests that π-donor R substituents donate electron density to the empty C_β_ p_*z*_ orbital and hence increases its basicity, consistent with the electrophilic mechanism in the 1,2-H migration process. In order to examine this, the indirect 1,2-H shift process was computed for X = CH_2_ and R = NMe_2_. Our calculations show that the transition state TS(C-10)_C–NMe2_ exhibits a short C_β_–H distance of 1.26 Å, showing a stronger interaction between the H and the C_β_ centres. This is due to NMe_2_ being a strong π-donor substituent that significantly populates the C_β_ p_*z*_ orbital to give a more stable transition state (at Δ*G*^‡^ = +4.7 kcal mol^−1^), supporting the electrophilic character of the H centre in the indirect 1,2-H shift process.

**Table 4:** Key atomic distances (Å) and the NBO charges (a.u.) of selected atoms in the transition state TS(C-10)_X–R_ – corresponding to indirect 1,2-H shift reaction

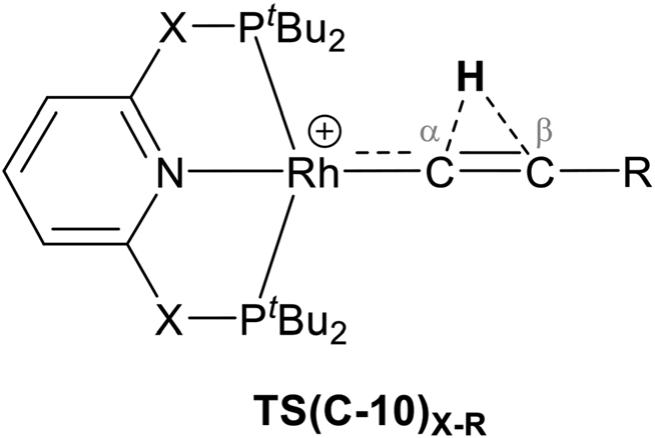						
Structure	C_α_⋯H	C_β_⋯H	Rh	H	C_α_	C_β_
TS(C-10)_C–_*_t_*_Bu_	1.18	1.54	−0.54	+0.39	−0.28	+0.06
TS(C-10)_O–_*_t_*_Bu_	1.18	1.55	−0.64	+0.39	−0.29	+0.09
TS(C-10)_C–Ar_	1.22	1.41	−0.55	+0.40	−0.20	−0.04
TS(C-10)_O–Ar_	1.24	1.36	−0.66	+0.40	−0.17	−0.06

### Characterisation of the Rh–vinylidene complexes 10_X–R_: Fischer or Schrock-type complex?

**Fig. 2:**
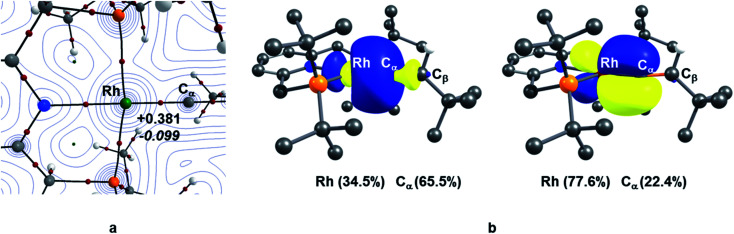
(a) QTAIM molecular graph of 10_C–_*_t_*_Bu_; the electron density contours are computed in the {Rh/P/C_α_} plane with bond critical points (BCPs) shown as small red spheres; for the Rh–C_α_ BCP, the Laplacian of the electron density (∇^2^*ρ*(*r*) in eÅ^−5^, in plain) and total energy density (*H*(*r*) in a.u., in italic) (b) NBO σ- and π-bonding orbitals of the Rh–C_α_ interaction and the contribution of the Rh and C_α_ centres in the corresponding interactions in 10_C–_*_t_*_Bu_.

TM–carbene complexes can be described as a Fischer or Schrock carbene complex, depending on the nature of the metal–carbene bond. In Fischer type carbene complexes, the carbene centre donates two electrons to the metal centre *via* a dative interaction whereas the metal centre donates electron density to the p_*z*_ orbital of the carbene centre through π-back bonding (*i.e.*, singlet carbene). In Schrock type carbene complexes, the carbene centre shares two single electron with the metal centre to form a σ and π metal–carbene bonds (*i.e.*, triplet carbene). Thus, the nature of the metal–carbene interactions in 10_X–R_ can provide useful information whether these species are Fischer or Schrock-type carbene complexes. In this context, in order to elucidate the nature of the metal–carbene bond in the Rh–vinylidene species 10_X–R_, QTAIM and NBO analyses were performed on the BP86-optimised geometries of 10_X–R_. As shown in Fig. 2, the QTAIM plot of 10_C–_*_t_*_Bu_ exhibits a BCP (Bond Critical Point) along the Rh⋯C_α_ bond path, which is characterised by a positive Laplacian of electron density and small negative total energy density. This is indicative of a dative interaction between the Rh and carbene centres.^51^ A similar trend can also be observed for the other Rh–vinylidene species (see ESI, Fig. S4†). NBO analysis of 10_C–_*_t_*_Bu_ identifies a Rh σ-bonding orbital that is dominated by carbene character (Rh (34.5%) and C_α_ (66.5%)), showing a Rh ← C_α_ dative interaction. Additionally, the NBO analysis reveals the presence of π-back donation from the Rh centre into the empty p_*z*_ orbital of the carbene, which is dominated by the Rh character (Rh (77.6%) and C_α_ (22.4%), see ESI, Fig. S4† for more details). Thus, the QTAIM and NBO data are fully consistent with a Rh(i) centre ligated by vinylidene (C_α_=C_β_HR) ligand and hence the Rh–vinylidene complexes 10_X–R_ can be described as a Fischer TM–carbene complex, seen previously in d^8^ TM–carbene complexes.^52^ It should be noted that in all the Rh–vinylidene complexes, the Rh–C_α_ σ-bond is polarised toward the C_α_ centre, while the Rh–C_α_ π-interaction is polarised toward the Rh centre (see ESI, Fig. S4†).

### Stability of Rh–alkyne *vs.* Rh–vinylidene complexes

As shown above, for R = Ar′ and the PNP pincer ligand, the Rh–vinylidene isomer is more stable than the Rh–alkyne isomer, while with the PONOP pincer ligand, the Rh–alkyne isomer is more stable. In order to explore the equilibrium between the Rh–alkyne and Rh–vinylidene isomers, the difference between the free energies of B_X–R_ and 10_X–R_ species were calculated and is represented by Δ*G*_r_ ((Δ*G*_r_ = Δ*G*(10_X–R_) − Δ*G*(B_X–R_), Table 5). A negative Δ*G*_r_ value indicates that the equilibrium is toward the Rh–vinylidene isomer while a positive value indicates the equilibrium is toward the Rh–alkyne isomer. As listed in Table 5, our computational findings show that for R = ^*t*^Bu and both PNP or PONOP-pincer ligands, Δ*G*_r_ is negative, with the PNP-pincer complex being significantly more negative than the PONOP-pincer complex (−11.9 and −5.2 kcal mol^−1^, respectively). For R = Ar′ and the PNP-pincer ligand, Δ*G*_r_ is also negative (−3.1 kcal mol^−1^) yet for the PONOP-pincer ligand Δ*G*_r_ becomes slightly positive (+0.1 kcal mol^−1^). This trend implies that with the aryl substituent, the Rh–vinylidene complex is less stable than the alkyl substituent. This becomes critically important when switching from the PNP pincer ligand to the PONOP pincer ligand, as in this case, the Rh–alkyne isomer becomes more stable than the Rh–vinylidene isomer. This highlights the influence of the nature of both substituent and pincer ligand on the stability of the Rh–vinylidene species.

**Table 5:** The difference between the free energies of B_X–Ar_ and 10_X–Ar_ represented by Δ*G*_r_ (in kcal mol^−1^) and HOMOs of B_X–Ar_ and 10_X–Ar_ and their difference represented by *Δ*_HOMO_ (in eV; X = NH, CH_2_, O and S)

X	R	Δ*G*_r_	HOMO (B_X–R_)	HOMO (10_X–R_)	*Δ*_HOMO_
CH_2_	^*t*^Bu	−11.9	−7.05	−7.31	+0.26
O	^*t*^Bu	−5.2	−7.54	−7.65	+0.11
S	Ar′	−3.2	−7.25	−7.31	−0.06
CH_2_	Ar′	−3.1	−6.87	−6.96	−0.09
NH	Ar′	−2.1	−7.01	−7.04	−0.03
O	Ar′	+0.1	−7.29	−7.23	+0.06

**Fig. 3:**
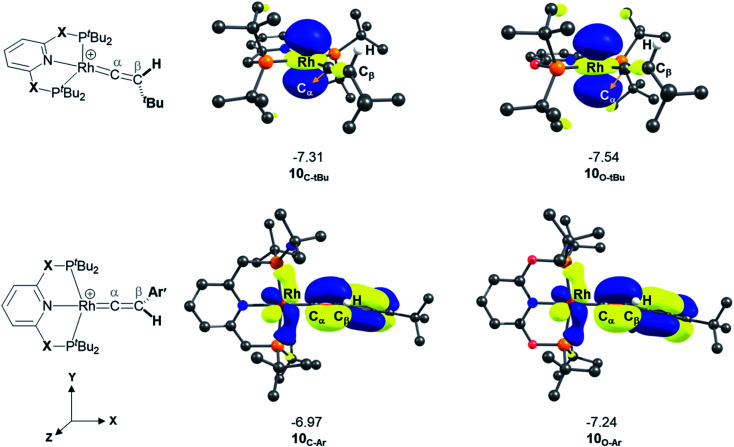
HOMOs (in eV) of Rh–vinylidene species 10_X–R_ (X = CH_2_ and O; R = ^*t*^Bu and Ar′). Substituent hydrogen atoms removed for clarity.

A detailed analysis of the electronic structure of the Rh–alkyne and Rh–vinylidene complexes can help to understand the origin of the equilibrium between the two isomers. As shown in Fig. 3, the molecular orbital analysis of the Rh–vinylidene species 10_X–R_ shows that when R = ^*t*^Bu, the HOMO of 10_X–R_ predominantly consists of the Rh d_*z*^2^_ character, whereas for R = Ar′, the HOMO consists of the Rh d_*xy*_ orbital and the π-system of the aryl moiety. With the PNP-pincer ligand, when R = Ar′, the Rh–vinylidene complex exhibits a higher HOMO than when R = ^*t*^Bu (−7.31 eV and −6.96 eV, respectively). This can be attributed to the presence of the π-system of the aryl moiety, which in conjugation with the C_α_–C_β_ π-bond and the {Rh(pincer)}^+^ moiety can destabilise the HOMO of the system and hence affects the stability of the Rh–vinylidene complex. Thus, it suggests that the HOMO energy is a reasonable descriptor that can capture the stability of the Rh–vinylidene isomer *versus* the Rh–alkyne isomer.

Table 5 lists the energy difference between the HOMOs of B_X–R_ and 10_X–R_ species, represented by *Δ*_HOMO_ (*Δ*_HOMO_ = *Δ*_HOMO_(10_X–R_) − *Δ*_HOMO_(B_X–R_)). For R = ^*t*^Bu and the PNP pincer ligand, *Δ*_HOMO_ is +0.26 eV, showing the Rh–vinylidene isomer possess a more stable HOMO than the Rh–alkyne isomer. Similarly, for R = ^*t*^Bu and the PONOP pincer ligand, *Δ*_HOMO_ is also a positive value (+0.11 eV). However, the PONOP analogue has a smaller *Δ*_HOMO_ than the PNP complex. A similar trend can also be seen for R = Ar′ (+0.09 eV). Interestingly, for R = Ar′, when the pincer ligand is PONOP, the Rh–alkyne complex features a more stable HOMO than the Rh–vinylidene isomer (*Δ*_HOMO_ = −0.06 eV). This suggests that for R = Ar′, the nature of the pincer ligand plays an important role on the stability of the HOMO of the Rh–vinylidene isomer *versus* the Rh–alkyne isomer. In order to investigate this further, the relative free energies of the vinylidene complex against the alkyne isomer was also computed for X = NH and S, and R = Ar′. Both X = NH and S showed a similar trend to X = CH_2_, with the Rh–vinylidene complex being more stable than the Rh–alkyne complex (Δ*G*_r_ = −2.1 and −3.2 kcal mol^−1^ respectively) in which the Rh–vinylidene isomer features a more stable HOMO than the Rh–alkyne isomer (*Δ*_HOMO_ = −0.03 and −0.06 eV respectively). Interestingly, a reasonable correlation (*R*^2^ = 0.95, Fig. S1†) was found between Δ*G*_r_ and *Δ*_HOMO_ values, showing when *Δ*_HOMO_ is negative, the Rh–vinylidene species is more stable than the Rh–alkyne species and when *Δ*_HOMO_ is positive, the Rh–alkyne species is more stable than the Rh–vinylidene species. This therefore indicates that for R = Ar′, *Δ*_HOMO_ can capture the stability of the Rh–vinylidene *versus* the Rh–alkyne species, however, raises the question; why is *Δ*_HOMO_ negative for X = CH_2_, NH or S, while for X = O, *Δ*_HOMO_ is positive?

**Fig. 4:**
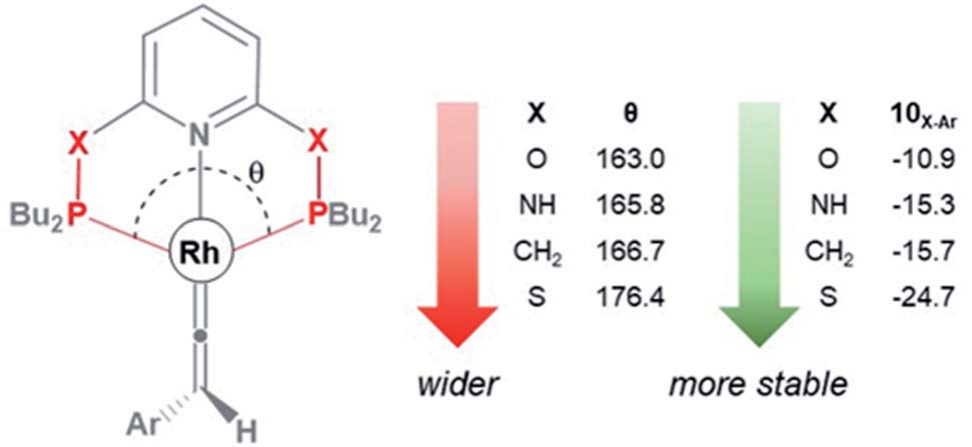
The P–Rh–P bite angle (*θ*, in degree) and the free energy (kcal mol^−1^) of the Rh–vinylidene species 10_X–Ar_.

As shown in Fig. 3, the HOMO of the Rh–vinylidene complex 10_X–Ar_ consists of the Rh d_*xy*_ orbital that is affected by the P–Rh–P bite angle (*θ*) of the supporting pincer ligand. For X = O, the *θ* value of 10_O–Ar_ is 163.0° and its HOMO energy is −7.23 eV (Fig. 4). In comparison to X = O, for X = S, the *θ* value of 10_S–Ar_ is considerably larger (176.4°) and its HOMO is more stable than 10_O–Ar_ (−7.31 eV). Interestingly, an excellent correlation (*R*^2^ = 0.9999, Fig. S2†) was found between the free energy of the Rh–vinylidene species 10_X–R_ and its *θ* value, showing tighter P–Rh–P bite angles result in more reactive Rh–vinylidene species (*i.e.*, higher HOMOs), while wider bite angles give more stable Rh–vinylidene species 10_X–R_ (*i.e.*, lower HOMO), consistent with recent work from Mansell.^53^ Thus, the bite angle is dictated by the nature of the X moiety and can therefore tune the reactivity of the Rh–vinylidene species *versus* the Rh–alkyne species B_X–R_.

## Conclusions

DFT calculations on the transformation of Rh–alkyne B_X–R_ into the Rh–vinylidene complex 10_X–R_ reveal that this process involves the slippage of the Rh–alkyne π-complex into the Rh–alkyne σ_C–H_ complex – followed by an indirect 1,2-H shift reaction. The H atom in the indirect 1,2-H shift transition state has protic character. The presence of π-donor groups at the accepting carbon centre (C_β_) can populate the C_β_ empty p_*z*_ orbital and hence increase its basicity to give stronger C_β_⋯H interactions, which consequently lower the free energy of the 1,2-H shift transition state. The equilibrium between the alkyne and vinylidene species can be tuned by the P–Rh–P bite angle, which is dictated by the nature of the X moiety in the PXNXP pincer ligand coordinated to the Rh-centre.

## Conflicts of interest

There are no conflicts to declare.

## Supplementary Material

RA-011-D0RA08764E-s001
