# Supplementary material for: DFT calculations bring insight to internal alkyne-to-vinylidene transformations at rhodium PNP- and PONOP-pincer complexes
Source: RSC Adv. 2021 Mar 23;11(20):11793–803. doi: 10.1039/d0ra08764e (PMC8696595; doi:10.1039/d0ra08764e)
Supplement: RA-011-D0RA08764E-s001 [file RA-011-D0RA08764E-s001.pdf]

Electronic Supplementary Material (ESI) for RSC Advances.
This journal is © The Royal Society of Chemistry 2021

DFT Calculations Bring Insight to Internal Alkyne-to-Vinylidene Transformations at Rhodium PNP- and PONOP-Pincer Complexes

Nasir A. Rajabi* and Claire L. McMullin

Department of Chemistry, University of Bath, Claverton Down, Bath, BA2 7AY, UK.

Breakdown of Energy Contributions

The following tables detail the evolution of the relative energies as the successive corrections to the initial SCF energy are included. Terms used are:

ΔE_{BS1}	SCF energy computed with the BP86 functional with BS1
ΔH_{BS1}	Enthalpy at 0 K with BS1
ΔG_{BS1}	Free energy at 298.15 K and 1 atm with BS1
$\Delta G_{BS1/DFB}$	Free energy corrected for 1,2-difluorobenzene (DFB) solvent with BS1
$\Delta G_{BS1/DFB+D3}$	Free energy corrected for DFB and dispersion effects with BS1
ΔE_{BS2}	SCF energy computed with the BP86 functional with BS2
ΔG_{DFB}	Free energy corrected for basis set (BS2), dispersion effects and DFB solvent
ΔG_{FB}	Free energy corrected for basis set (BS2), dispersion effects and fluorobenzene solvent (FB, in blue)

In each case the final data used in the main article is highlighted in bold.

Table S1 – Relative energies (kcal/mol) for computed structures in Pathways **I**, **II** and **III**. Data in bold are those used in the main text. All energies are quoted relative to $\mathbf{9_X}$ **and HC≡CR** at 0.0 kcal mol^{-1} (X = CH_2 and O; R = ^{t}Bu and Ar′ (3,5-$^t Bu_2C_6H_3$)).

	ΔE_{BS1}	ΔH_{BS1}	ΔG_{BS1}	$\Delta G_{BS1/DFB}$	$\Delta G_{BS1/DFB+D3}$	ΔE_{BS2}	ΔG_{DFB}	ΔG_{FB}
**9_X + HC≡CR**	0.0	0.0	0.0	0.0	0.0	0.0	**0.0**	0.0
**A_C**	-9.1	-10.9	-26.3	-2.8	+23.5	-12.5	**+20.0**	+18.1
**$B_{C\text{-}tBu}$**	-38.0	-37.7	-38.2	-7.5	-1.9	-36.3	**-0.2**	-3.7
**TS(B-10)$_{C\text{-}tBu}$**	+3.7	1.7	+1.5	+31.5	+37.8	+6.0	**+40.1**	+36.8
**TS(B-C)$_{C\text{-}tBu}$**	-30.7	-32.2	-34.3	-2.8	+7.2	-29.1	**+8.8**	+5.2
**$C_{C\text{-}tBu}$**	-40.5	-42.0	-45.1	-14.2	-2.9	-40.6	**-3.0**	-6.4
**TS(C-D)$_{C\text{-}tBu}$**	+2.6	0.8	0.0	+28.5	35.9	+4.4	**+37.7**	+34.8
**$D_{C\text{-}tBu}$**	+2.0	0.5	-0.3	+27.8	+35.0	+3.4	**+36.4**	+33.6
**TS(C-10)$_{C\text{-}tBu}$**	-29.7	-32.6	-35.1	-3.9	+7.8	-28.7	**+8.7**	+5.3
**TS(C-E)$_{C\text{-}tBu}$**	-38.2	-40.7	-42.7	-13.5	-2.8	-38.2	**-2.8**	-5.8
**$E_{C\text{-}tBu}$**	-42.9	-44.4	-46.3	-23.6	-13.7	-42.3	**-8.1**	-10.6
**$10_{C\text{-}tBu}$**	-52.3	-52.4	-53.9	-27.0	-17.1	-50.6	**-12.1**	-15.5
**A_O**	-5.3	-7.2	-22.5	+1.8	+26.0	-8.3	**23.0**	+20.9
**$B_{O\text{-}tBu}$**	-41.1	-41.6	-42.7	-9.8	-5.5	-39.2	**-3.7**	-7.7
**TS(B-10)$_{\text{-}tBu}$**	+2.5	-0.1	-1.6	+30.9	+36.9	4.9	**+39.4**	+35.5
**TS(B-C)$_{O\text{-}tBu}$**	-32.7	-34.7	-37.7	-4.4	+4.6	-30.8	**+6.5**	+2.4
**$C_{O\text{-}tBu}$**	-38.4	-40.0	-43.6	-9.9	+0.9	-38.0	**+1.3**	-2.8
**TS(C-D)$_{O\text{-}tBu}$**	+1.6	-1.1	-2.3	29.7	+36.5	+3.7	**+38.5**	+34.7
**$D_{O\text{-}tBu}$**	+1.5	-0.7	-2.6	28.7	+35.2	+3.3	**+37.0**	+33.4
**TS(C-10)$_{O\text{-}tBu}$**	-28.5	-31.6	-35.4	-2.2	+8.9	-27.3	**+10.2**	+6.2
**TS(C-E)$_{O\text{-}tBu}$**	-35.2	-38.0	-41.7	-9.5	+0.3	-34.7	**+0.8**	-2.9
**$E_{O\text{-}tBu}$**	-39.1	-40.8	-42.2	-11.2	-1.8	-37.9	**-0.6**	-4.0
**$10_{O\text{-}tBu}$**	-49.7	-50.2	-53.4	-20.1	-10.9	-47.6	**-8.9**	-12.9
**$B_{C\text{-}Ar}$**	-47.6	-48.0	-48.9	-16.7	-13.7	-46.5	**-12.6**	-16.1
**TS(B-10)$_{C\text{-}Ar}$**	-3.4	-5.8	-7.0	+24.8	+28.5	-1.6	**+30.3**	+26.9
**TS(B-C)$_{\text{-}Ar}$**	-41.7	-43.1	-43.9	-10.0	-1.3	-40.2	**+0.2**	-3.7
**$C_{C\text{-}Ar}$**	-44.5	-45.8	-47.5	-15.2	-4.2	-44.8	**-4.5**	-8.1
**TS(C-D)$_{\text{-}Ar}$**	-4.3	-6.4	-8.0	+23.0	28.2	-2.7	**+29.8**	+26.5
**TS(C-10)$_{\text{-}Ar}$**	-32.8	-35.9	-37.9	-4.7	+7.0	-32.2	**+7.6**	3.9
**TS(C-E)$_{\text{-}Ar}$**	-40.8	-43.5	-45.4	-15.6	-5.0	-41.3	**-5.5**	-8.5
**$D_{C\text{-}Ar}$**	-45.4	-47.1	-49.5	-21.8	-11.5	-45.3	**-11.4**	-13.7
**$10_{C\text{-}Ar}$**	-53.9	-54.2	-56.3	-25.7	-16.6	-52.9	**-15.7**	-18.9
**$B_{O\text{-}Ar}$**	-47.8	-48.2	-49.4	-14.4	-12.6	-46.2	**-11.0**	-15.3
**TS(B-10)$_{O\text{-}Ar}$**	-4.6	-7.3	-8.5	26.2	+28.9	-2.5	**+31.0**	+26.8
**TS(B-C)$_{O\text{-}Ar}$**	-40.2	-41.7	-44.1	-7.9	+1.1	-38.6	**+2.8**	-1.6

**$C_{O\text{-}Ar}$**	-42.1	-43.7	-47.0	-11.6	-1.1	-41.9	**-1.0**	-5.2
**$TS(C\text{-}D)_{O\text{-}Ar}$**	-4.5	-6.8	-7.8	+26.5	+29.8	-2.7	**+31.6**	+27.6
**$TS(C\text{-}10)_{O\text{-}Ar}$**	-31.7	-34.9	-38.6	-3.0	+8.2	-30.8	**+9.1**	+4.9
**$TS(C\text{-}E)_{O\text{-}Ar}$**	-37.8	-40.7	-43.9	-10.9	-1.3	-37.7	**-1.2**	-5.0
**$E_{O\text{-}Ar}$**	-41.4	-43.2	-45.8	-14.3	-5.1	-40.7	**-4.4**	-7.7
**$10_{O\text{-}Ar}$**	-51.1	-51.5	-54.5	-20.5	-12.6	-49.4	**-10.9**	-15.0
**$TS(C\text{-}10)_{C\text{-}NMe2}$**	-37.4	-39.7	-41.8	-10.0	+3.0	-35.7	**+4.7**	+1.2
**A_{NH}**	-6.8	-8.9	-25.1	-1.8	23.7	-10.1	**+20.3**	18.4
**B_{NH}**	-48.2	-48.8	-50.4	-17.4	-14.5	-46.9	**-13.2**	-17.0
**10_{NH}**	-52.9	-53.5	-57.0	-25.2	-16.7	-51.5	**-15.3**	-18.9
**A_{S}**	-19.5	-22.1	-39.0	-10.2	16.2	-23.1	**+12.6**	9.5
**B_{S}**	-56.4	-56.9	-60.9	-26.4	-23.3	-54.7	**-21.5**	-25.6
**10_{S}**	-64.7	-65.0	-69.4	-35.6	-26.2	-63.2	**-24.7**	-28.7

Table S2 – DFT calculated free energies in kcal mol^{-1} for the direct 1,2-R transfer in the Rh-alkyne species $\mathbf{B_{X\text{-}R}}$ to form the Rh-vinylidene species $\mathbf{10_{X\text{-}R}}$ *via* Pathway **I**. All free energies are quoted with respect to $\mathbf{9_X}$ and **HC≡CR** (X = CH_2 and O, R = ^{t}Bu and Ar′ (3,5-$^t Bu_2C_6H_3$)).

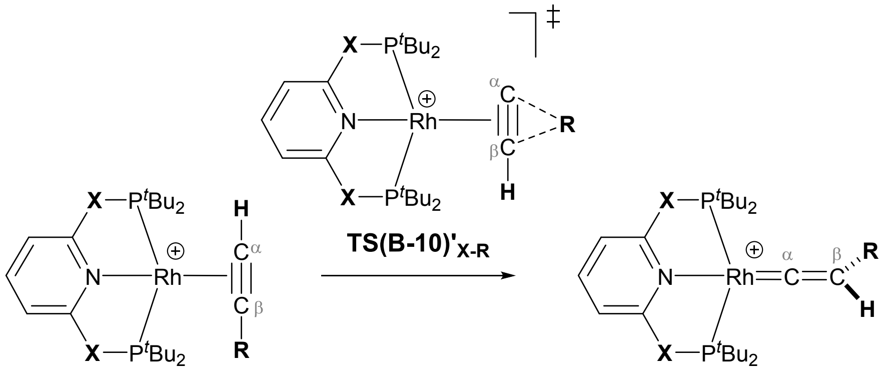

	ΔE_{BS1}	ΔH_{BS1}	ΔG_{BS1}	$\Delta G_{BS1/DFB}$	$\Delta G_{BS1/DFB+D3}$	ΔE_{BS2}	ΔG_{DFB}	ΔG_{FB}
**9_X + HC≡CR**	0.0	0.0	0.0	0.0	0.0	0.0	**0.0**	0.0
**$B_{C\text{-}tBu}$**	-38.0	-37.7	-38.2	-7.5	-1.9	-36.3	**-0.2**	-3.7
**TS(B-10)'$_{C\text{-}tBu}$**	21.9	20.3	20.3	50.3	56.5	22.8	**57.4**	54.2
**$B_{C\text{-}Ar}$**	-47.6	-48.0	-48.9	-16.7	-13.7	-46.5	**-12.6**	-16.1
**TS(B-10)'$_{C\text{-}Ar}$**	6.0	4.7	5.6	38.6	41.3	8.2	**43.5**	39.8
**$B_{O\text{-}tBu}$**	-41.1	-41.6	-42.7	-9.8	-5.5	-39.2	**-3.7**	-7.7
**TS(B-10)'$_{O\text{-}tBu}$**	15.8	13.2	11.3	43.4	48.5	16.3	**49.0**	45.3
**$B_{O\text{-}Ar}$**	-47.8	-48.2	-49.4	-14.4	-12.6	-46.2	**-11.0**	-15.3
**TS(B-10)'$_{O\text{-}Ar}$**	0.8	-0.6	-0.3	35.3	36.7	3.3	**39.2**	34.9

Table S3 – DFT calculated free energies in kcal mol^{-1} for the indirect 1,2-R transfer in the Rh-alkyne species $\mathbf{B_{X\text{-}R}}$ to form the Rh-vinylidene species $\mathbf{10_{X\text{-}R}}$ *via* Pathway **III**. All free energies are quoted with respect to $\mathbf{9_X}$ and **HC≡CR** (X = CH_2 and O, R = ^{t}Bu and Ar′ (3,5-$^t Bu_2C_6H_3$)).

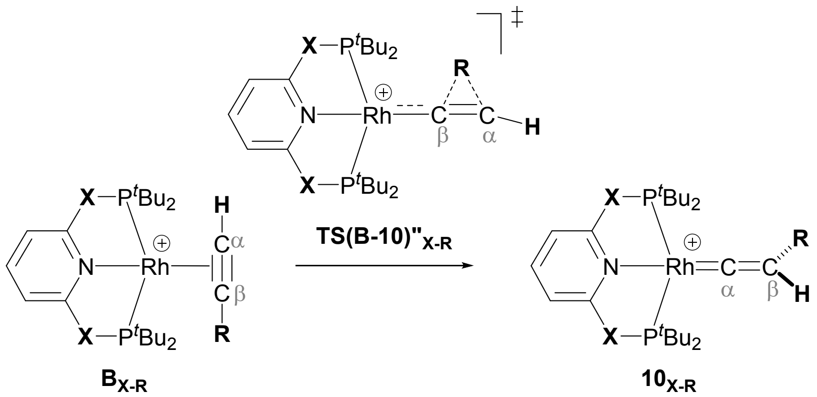

	ΔE_{BS1}	ΔH_{BS1}	ΔG_{BS1}	$\Delta G_{BS1/DFB}$	$\Delta G_{BS1/DFB+D3}$	ΔE_{BS2}	ΔG_{DFB}	ΔG_{FB}
**9_X + HC≡CR**	0.0	0.0	0.0	0.0	0.0	0.0	**0.0**	0.0
**$B_{C\text{-}tBu}$**	-38.0	-37.7	-38.2	-7.5	-1.9	-36.3	**-0.2**	-3.7
**TS(B-10)"$_{C\text{-}tBu}$**	5.3	3.8	3.6	34.4	39.6	6.3	**40.6**	37.2
**$B_{C\text{-}Ar}$**	-47.6	-48.0	-48.9	-16.7	-13.7	-46.5	**-12.6**	-16.1
**TS(B-10)"$_{C\text{-}Ar}$**	-5.8	-7.6	-7.9	25.0	28.1	-4.2	**29.8**	26.1
**$B_{O\text{-}tBu}$**	-41.1	-41.6	-42.7	-9.8	-5.5	-39.2	**-3.7**	-7.7
**TS(B-10)"$_{O\text{-}tBu}$**	2.4	0.9	0.4	32.7	37.3	4.0	**38.9**	35.0
**$B_{O\text{-}Ar}$**	-47.8	-48.2	-49.4	-14.4	-12.6	-46.2	**-11.0**	-15.3
**TS(B-10)"$_{O\text{-}Ar}$**	-8.9	-11.1	-12.4	22.5	24.7	-7.2	**26.4**	22.2

Table S4 – DFT calculated free energies in kcal mol^{-1} for transfer of the R group onto the Rh in **$10_{X\text{-}Ar}$** *via* **$TS(10\text{-}F)_{X\text{-}R}$** to give the Rh—R species **$F_{X\text{-}Ar}$**. Free energies are reported with respect to **9_X** and HC≡CAr′ (X = CH_2 and O).

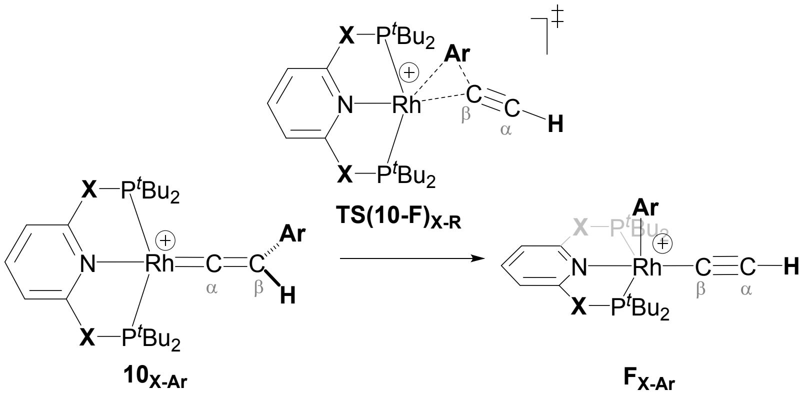

	ΔE$_{BS1}$	ΔH$_{BS1}$	ΔG$_{BS1}$	ΔG$_{BS1/DFB}$	ΔG$_{BS1/DFB+D3}$	ΔE$_{BS2}$	ΔG$_{DFB}$	ΔG$_{FB}$
**9_X + HC≡CAr′**	0.0	0.0	0.0	0.0	0.0	0.0	**0.0**	0.0
**$B_{C\text{-}Ar}$**	-47.6	-48.0	-48.9	-16.7	-13.7	-46.5	**-12.6**	-16.1
**$10_{C\text{-}tBu}$**	-52.3	-52.4	-53.9	-27.0	-17.1	-50.6	**-12.1**	-15.5
**$TS(10\text{-}F)_{C\text{-}Ar}$**	+6.8	+5.1	+6.1	+37.6	+32.6	+6.9	**32.7**	+29.3
**$F_{C\text{-}Ar}$**	-19.9	-20.9	-20.3	+9.1	+1.2	-19.9	**+1.1**	-1.8
**$B_{O\text{-}Ar}$**	-47.8	-48.2	-49.4	-14.4	-12.6	-46.2	**-11.0**	-15.3
**$10_{O\text{-}Ar}$**	-51.1	-51.5	-54.5	-20.5	-12.6	-49.4	**-10.9**	-15.0
**$TS(10\text{-}F)_{O\text{-}Ar}$**	+10.5	+9.0	+9.7	+44.3	+38.3	+11.2	**+39.0**	+34.9
**$F_{O\text{-}Ar}$**	-15.9	-16.9	-16.7	+16.8	+7.4	-15.2	**+8.1**	+4.2

Table S5 – DFT calculated free energies in kcal mol^{-1} for transfer of the R group onto the Rh in $\mathbf{B_{X\text{-}tBu}}$ *via* $\mathbf{TS(B\text{-}F)_{X\text{-}tBu}}$ to give the Rh-^{t}Bu species $\mathbf{F_{X\text{-}tBu}}$. Free energies are reported with respect to $\mathbf{9_X}$ and HC≡C^{t}Bu (X = CH_2 and O).

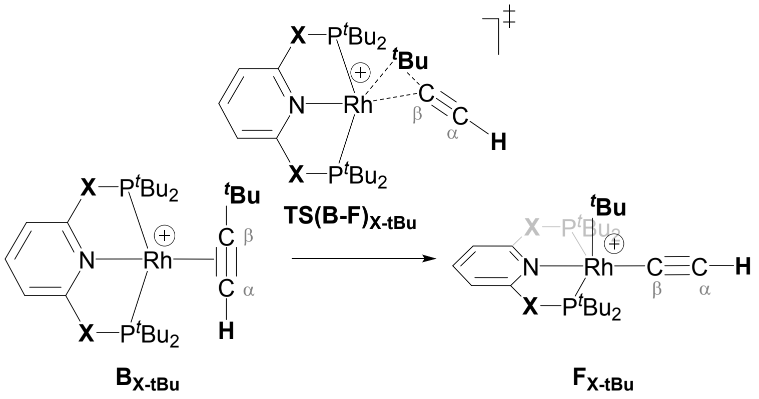

	ΔE_{BS1}	ΔH_{BS1}	ΔG_{BS1}	$\Delta G_{BS1/DFB}$	$\Delta G_{BS1/DFB+D3}$	ΔE_{BS2}	ΔG_{DFB}	ΔG_{FB}
**9_X + HC≡CtBu**	0.0	0.0	0.0	0.0	0.0	0.0	**0.0**	0.0
**$B_{C\text{-}tBu}$**	-38.0	-37.7	-38.2	-7.5	-1.9	-36.3	**-0.2**	-3.7
**$TS(B\text{-}F)_{C\text{-}tBu}$**	16.7	15.1	15.4	46.1	46.1	17.1	**46.6**	43.1
**$F_{C\text{-}tBu}$**	4.6	4.1	4.4	33.8	32.0	5.4	**32.8**	29.7
**$B_{O\text{-}tBu}$**	-41.1	-41.6	-42.7	-9.8	-5.5	-39.2	**-3.7**	-7.7
**$TS(B\text{-}F)_{O\text{-}tBu}$**	16.4	15.0	14.9	47.9	47.2	17.0	**47.8**	43.8
**$F_{O\text{-}tBu}$**	5.7	5.0	5.0	37.4	34.3	7.3	**35.9**	32.0

Table S6 – The P—Rh—P bite angles (θ, in degrees) in species $\mathbf{A_X}$, $\mathbf{B_{X\text{-}R}}$ and $\mathbf{10_{X\text{-}R}}$ (X = CH_2 and O, R = ^{t}Bu and Ar′).

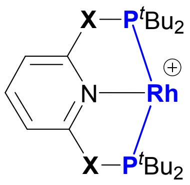

$\mathbf{A_X}$

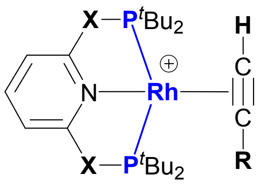

$\mathbf{B_{X\text{-}R}}$

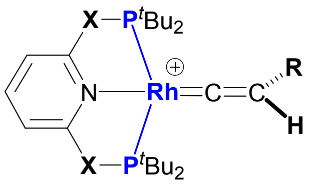

$\mathbf{10_{X\text{-}R}}$

Species	P—Rh—P	Species	P—Rh—P	Species	P—Rh—P	Species	P—Rh—P
$\mathbf{A_C}$	171.1	$\mathbf{A_O}$	166.0	$\mathbf{A_C}$	171.1	$\mathbf{A_O}$	166.0
$\mathbf{B_{C\text{-}tBu}}$	161.1	$\mathbf{B_{O\text{-}tBu}}$	162.5	$\mathbf{B_{C\text{-}Ar}}$	163.4	$\mathbf{B_{O\text{-}Ar}}$	162.5
$\mathbf{10_{C\text{-}tBu}}$	166.3	$\mathbf{10_{O\text{-}tBu}}$	162.9	$\mathbf{10_{C\text{-}Ar}}$	166.7	$\mathbf{10_{O\text{-}Ar}}$	163.0

Table S7 – The HOMOs (in eV) of $\mathbf{A_X}$, $\mathbf{B_{X\text{-}R}}$ and $\mathbf{10_{X\text{-}R}}$ (X = CH_2 and O, R = ^{t}Bu and Ar).

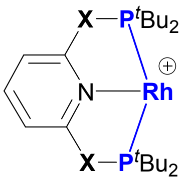

$\mathbf{A_X}$

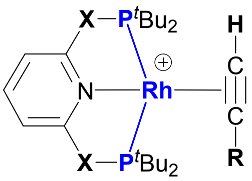

$\mathbf{B_{X\text{-}R}}$

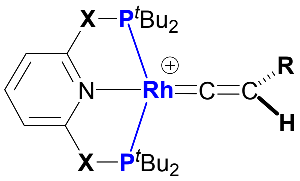

$\mathbf{10_{X\text{-}R}}$

**Species**	HOMO	**Species**	HOMO	**Species**	HOMO	**Species**	HOMO
$\mathbf{A_C}$	-7.03	$\mathbf{A_O}$	-7.38	$\mathbf{A_C}$	-7.03	$\mathbf{A_O}$	-7.38
$\mathbf{B_{C\text{-}tBu}}$	-7.05	$\mathbf{B_{O\text{-}tBu}}$	-7.54	$\mathbf{B_{C\text{-}Ar}}$	-6.87	$\mathbf{B_{O\text{-}Ar}}$	-7.30
$\mathbf{10_{C\text{-}tBu}}$	-7.31	$\mathbf{10_{O\text{-}tBu}}$	-7.65	$\mathbf{10_{C\text{-}Ar}}$	-6.97	$\mathbf{10_{O\text{-}Ar}}$	-7.23

Table S8 – The P—Rh—P bite angles (θ, in degrees) in species **A**$_{X}$, **B**$_{X\text{-}R}$ and **10**$_{X\text{-}R}$ (X = NH, CH_2, O and S).

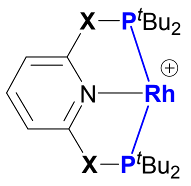

A$_{X}$

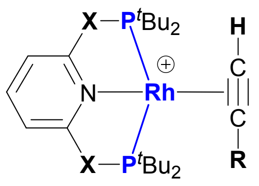

B$_{X\text{-}R}$

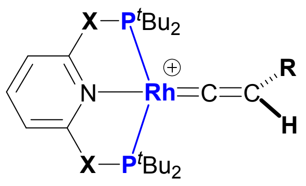

10$_{X\text{-}R}$

**Species**	P—Rh—P	**Species**	P—Rh—P	**Species**	P—Rh—P	**Species**	P—Rh—P
**A**$_{C}$	171.1	**A**$_{O}$	166.0	**A**$_{NH}$	169.0	**A**$_{S}$	178.8
**B**$_{C\text{-}Ar}$	163.4	**B**$_{O\text{-}Ar}$	162.5	**B**$_{NH\text{-}Ar}$	169.9	**B**$_{S\text{-}Ar}$	167.5
**10**$_{C\text{-}Ar}$	166.7	**10**$_{O\text{-}Ar}$	163.0	**10**$_{NH\text{-}Ar}$	165.8	**10**$_{S\text{-}Ar}$	176.4

Table S9 – The HOMOs (in eV) of $\mathbf{A_X}$, $\mathbf{B_{X\text{-}Ar}}$ and $\mathbf{10_{X\text{-}Ar}}$ (X = CH_2 and NH, O and S).

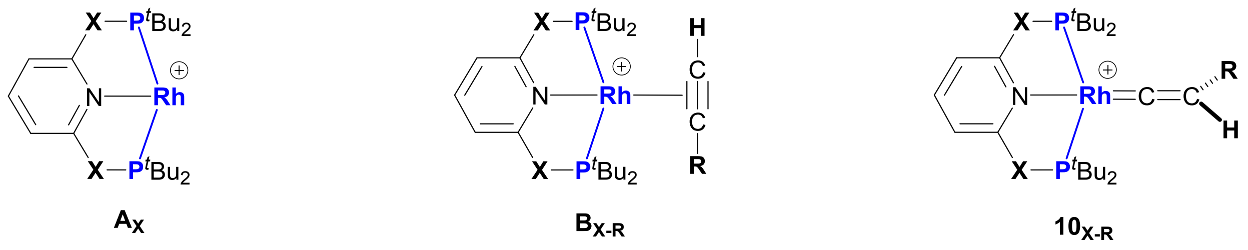

**Species**	HOMO	**Species**	HOMO	**Species**	HOMO	**Species**	HOMO
$\mathbf{A_C}$	-7.03	$\mathbf{A_O}$	-7.38	$\mathbf{A_S}$	-7.25	$\mathbf{A_{NH}}$	-7.00
$\mathbf{B_{C\text{-}Ar}}$	-6.87	$\mathbf{B_{O\text{-}Ar}}$	-7.30	$\mathbf{B_{S\text{-}Ar}}$	-7.25	$\mathbf{B_{NH\text{-}Ar}}$	-7.01
$\mathbf{10_{C\text{-}Ar}}$	-6.97	$\mathbf{10_{O\text{-}Ar}}$	-7.24	$\mathbf{10_{S\text{-}Ar}}$	-7.31	$\mathbf{10_{NH\text{-}Ar}}$	-7.04

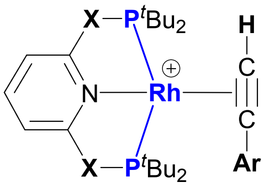

$\mathbf{B}_{\mathbf{X\text{-}Ar}}$

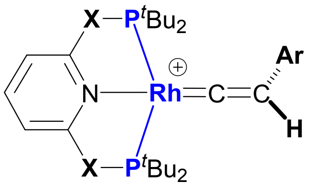

$\mathbf{10}_{\mathbf{X\text{-}Ar}}$

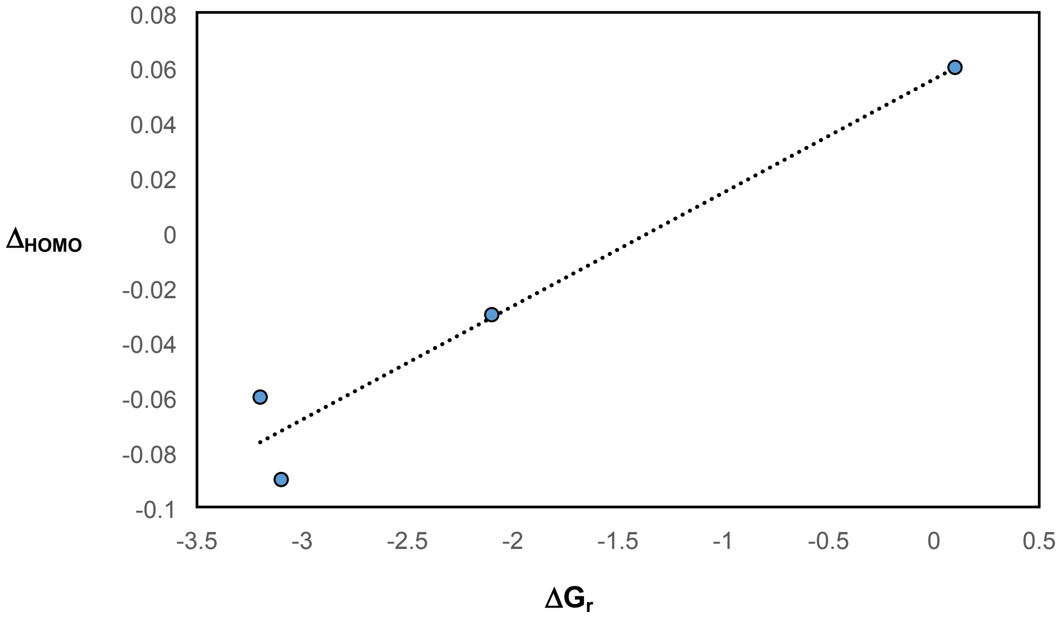

Figure S1 – Plot of the difference between the free energies of $\mathbf{B}_{\mathbf{X\text{-}Ar}}$ and $\mathbf{10}_{\mathbf{X\text{-}Ar}}$ ($\Delta\mathbf{G}_{\mathbf{r}}$, in kcal mol^{-1}) and difference between the HOMOs of $\mathbf{B}_{\mathbf{X\text{-}Ar}}$ and $\mathbf{10}_{\mathbf{X\text{-}Ar}}$ ($\Delta_{\mathbf{HOMO}}$ (in eV); X = NH, CH_2, O and S).

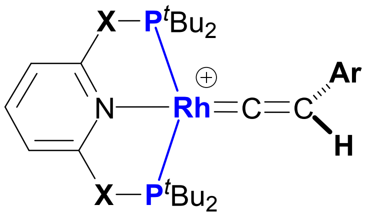

$10_{X\text{-}R}$

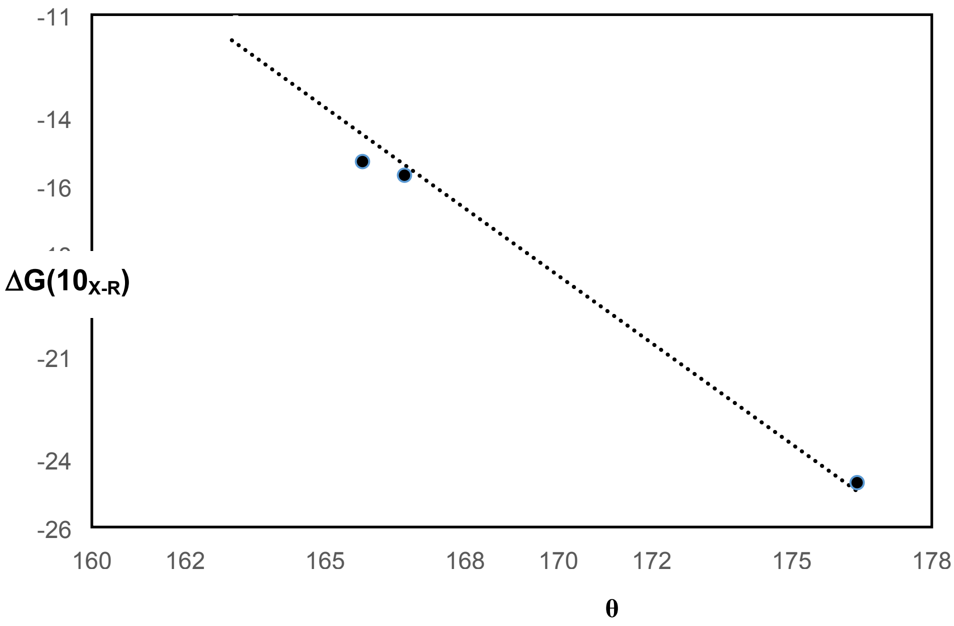

Figure S2 – Plot of the relative free energy of **$10_{X\text{-}R}$** with respect to **$B_{X\text{-}R}$** (ΔG, in kcal mol^{-1},) *vs* the P—Rh—P bite angle of **$10_{X\text{-}R}$** (θ (in degrees); X = NH, CH_2, O and S).

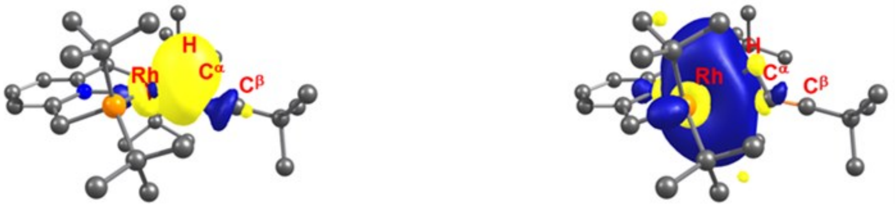

σ(C$^{\alpha}$—H)→LP*(Rh)

82.9 kcal mol^{-1}

C$_{C\text{-}tBu}$

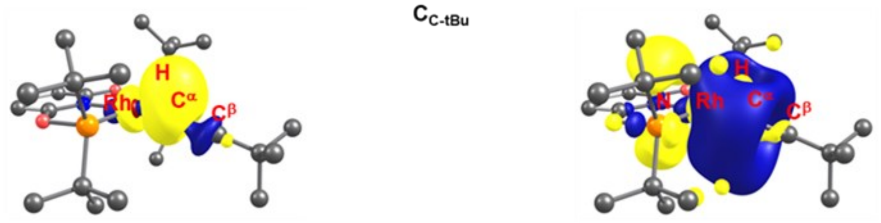

σ(C$^{\alpha}$—H)→LP*(Rh)

72.2 kcal mol^{-1}

C$_{O\text{-}tBu}$

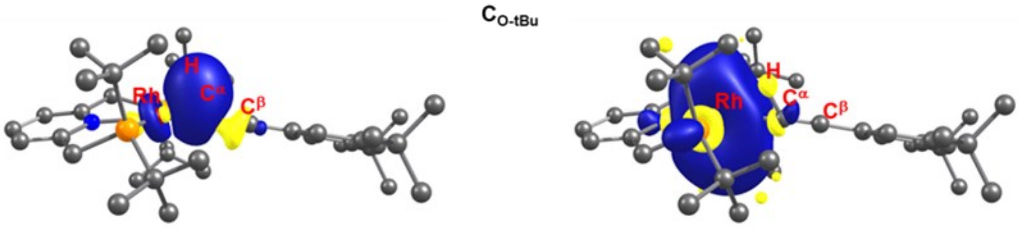

σ(C$^{\alpha}$—H)→LP*(Rh)

73.9 kcal mol^{-1}

C$_{C\text{-}Ar}$

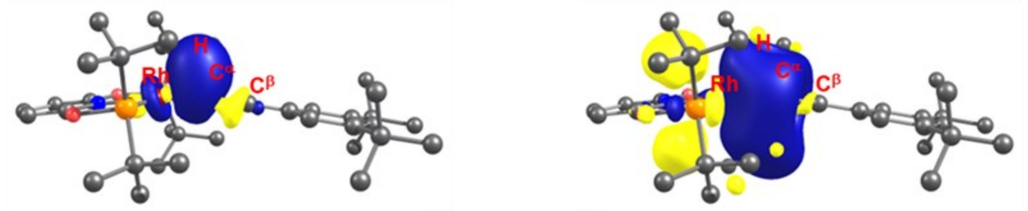

σ(C$^{\alpha}$—H)→LP*(Rh)

67.1 kcal mol^{-1}

C$_{O\text{-}Ar}$

Figure **S3**. NBO donor-acceptor interactions derived from second-order perturbation theory, showing the interactions between the C$^{\alpha}$⍰H bonding orbital (left) and an empty Rh orbital (LP*, right) in **C$_{X\text{-}R}$** with the stabilisation energies (in kcal mol^{-1}).

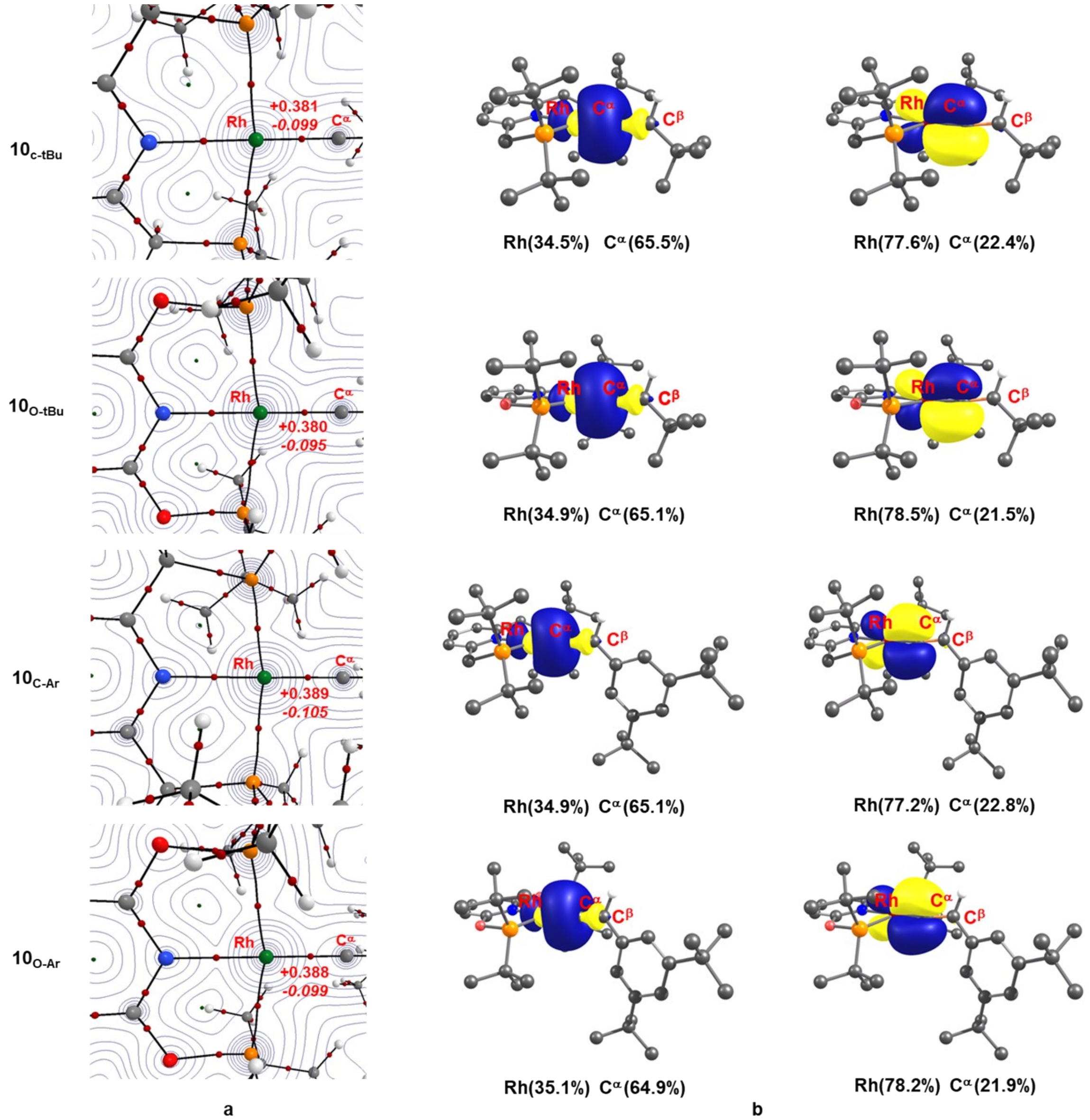

Figure **S4**. (**a**) QTAIM molecular graph of $\mathbf{10_{X\text{-}R}}$; the electron density contours are computed in the {Rh/P/C$^\alpha$} plane with bond critical points (BCPs) shown as small red spheres; for the Rh—C$^\alpha$ BCP, the Laplacian of the electron density ($\nabla^2\rho(r)$ in eÅ^{-5}) is shown in plain and total energy densities (H(r) in a.u.) is shown in italic; (**b**) NBO σ- and π- bonding orbitals of the Rh—C$^\alpha$ interaction and the contribution of the Rh and C$^\alpha$ centres in the corresponding interactions in $\mathbf{10_{X\text{-}R}}$.

Cartesian Coordinates and Computed Energies (in Hartrees)

9_C

SCF (BP86) Energy = -2473.63991160
Enthalpy 0K = -2472.254901
Enthalpy 298K = -2472.173582
Free Energy 298K = -2472.365403
Lowest Frequency = 7.6621 cm^{-1}
Second Frequency = 14.0646 cm^{-1}
SCF (BP86-D3BJ) Energy = -2474.111384
SCF (FB) Energy = -2473.763495
SCF (DFB) Energy = -2473.78008
SCF (BS2) Energy = -3812.790739

Rh	2.70768	-2.09034	1.15331
P	4.14307	-2.74359	-0.61842
P	1.82376	-1.84213	3.41662
N	4.02373	-3.47044	2.23615
C	1.11905	-1.47635	-0.23631
H	1.43377	-1.92392	-1.18720
C	1.84738	-0.32656	0.16302
H	2.64532	-0.01583	-0.52382
C	1.26907	0.86581	0.90858
H	2.09724	1.49017	1.28802
H	0.68662	0.54923	1.78817
C	-0.35043	-1.73662	0.03443
H	-0.60610	-1.52407	1.08418
H	-0.56427	-2.80826	-0.12871
C	5.23812	-3.84529	1.72348
C	6.10940	-4.69678	2.42265
H	7.06908	-4.96522	1.97274
C	5.74636	-5.17927	3.68355
H	6.41464	-5.83950	4.24421
C	4.50590	-4.80210	4.20831
H	4.17240	-5.16657	5.18352
C	3.66495	-3.95766	3.46616
C	5.63597	-3.29315	0.38766
H	6.26484	-4.01267	-0.16262
H	6.24845	-2.38598	0.53903
C	2.31108	-3.58168	3.97282
H	2.24554	-3.71677	5.06339
H	1.56306	-4.24712	3.50734
C	3.61789	-4.37599	-1.55684
C	3.39912	-5.44983	-0.46497
H	4.33686	-5.75121	0.03020
H	2.97785	-6.35432	-0.93995
H	2.68727	-5.10724	0.30555
C	4.67170	-4.87977	-2.56405
H	4.73144	-4.24288	-3.46044
H	4.37651	-5.88894	-2.90473
H	5.68131	-4.97054	-2.13024
C	2.27406	-4.16052	-2.28844
H	1.45552	-3.96366	-1.57803
H	2.01849	-5.08554	-2.83652
H	2.31036	-3.34416	-3.02805
C	5.01237	-1.45755	-1.80206
C	5.37781	-0.22422	-0.93825
H	4.52449	0.18496	-0.37731
H	5.76526	0.56942	-1.60248
H	6.17793	-0.44566	-0.21174
C	4.03155	-1.04928	-2.92057
H	3.82644	-1.88217	-3.61276
H	4.47943	-0.23343	-3.51694
H	3.07028	-0.68280	-2.52511
C	6.32083	-1.98400	-2.43743
H	7.06057	-2.30868	-1.68788
H	6.78594	-1.15491	-3.00163
H	6.15948	-2.80729	-3.14584
C	0.00514	-1.83534	4.16815
C	-0.74455	-3.05619	3.57666
H	-0.61687	-3.15227	2.48580
H	-1.82522	-2.94096	3.77674
H	-0.43835	-4.00448	4.04684
C	-0.75716	-0.55186	3.77795
H	-0.26729	0.36559	4.13565
H	-1.76096	-0.58982	4.23854
H	-0.90038	-0.46580	2.68932
C	-0.00126	-1.95593	5.70917
H	0.55020	-2.83574	6.07786
H	-1.04820	-2.07444	6.04465
H	0.39682	-1.05917	6.20865
C	2.98876	-0.66573	4.47583
C	4.27304	-0.43326	3.64272
H	4.88446	-1.34532	3.55859
H	4.89064	0.33387	4.14575
H	4.04333	-0.08283	2.62064
C	3.38773	-1.27383	5.83729
H	2.52877	-1.47047	6.49581
H	4.04441	-0.55744	6.36409
H	3.96352	-2.20702	5.72267
C	2.30529	0.70208	4.69531
H	3.03562	1.39079	5.15747
H	1.44403	0.64297	5.37887
H	1.97651	1.16243	3.74883
C	-1.11905	1.47635	0.23631
H	-1.43377	1.92392	1.18720
C	-1.84738	0.32656	-0.16302
H	-2.64532	0.01583	0.52382
C	-1.26907	-0.86581	-0.90858
H	-2.09724	-1.49017	-1.28802
H	-0.68662	-0.54923	-1.78817
C	0.35043	1.73662	-0.03443
H	0.60610	1.52407	-1.08418
H	0.56427	2.80826	0.12871
Rh	-2.70768	2.09034	-1.15331
P	-4.14307	2.74359	0.61842
P	-1.82376	1.84213	-3.41662
N	-4.02373	3.47044	-2.23615
C	-5.23812	3.84529	-1.72348
C	-6.10940	4.69678	-2.42265
H	-7.06908	4.96522	-1.97274
C	-5.74636	5.17927	-3.68355
H	-6.41464	5.83950	-4.24421
C	-4.50590	4.80210	-4.20831
H	-4.17240	5.16657	-5.18352
C	-3.66495	3.95766	-3.46616
C	-5.63597	3.29315	-0.38766
H	-6.26484	4.01267	0.16262
H	-6.24845	2.38598	-0.53903
C	-2.31108	3.58168	-3.97282
H	-2.24554	3.71677	-5.06339
H	-1.56306	4.24712	-3.50734
C	-3.61789	4.37599	1.55684
C	-3.39912	5.44983	0.46497
H	-4.33686	5.75121	-0.03020
H	-2.97785	6.35432	0.93995

H -2.68727 5.10724 -0.30555
C -4.67170 4.87977 2.56405
H -4.73144 4.24288 3.46044
H -4.37651 5.88894 2.90473
H -5.68131 4.97054 2.13024
C -2.27406 4.16052 2.28844
H -1.45552 3.96366 1.57803
H -2.01849 5.08554 2.83652
H -2.31036 3.34416 3.02805
C -5.01237 1.45755 1.80206
C -5.37781 0.22422 0.93825
H -4.52449 -0.18496 0.37731
H -5.76526 -0.56942 1.60248
H -6.17793 0.44566 0.21174
C -4.03155 1.04928 2.92057
H -3.82644 1.88217 3.61276
H -4.47943 0.23343 3.51694
H -3.07028 0.68280 2.52511
C -6.32083 1.98400 2.43743
H -7.06057 2.30868 1.68788
H -6.78594 1.15491 3.00163
H -6.15948 2.80729 3.14584
C -0.00514 1.83534 -4.16815
C 0.74455 3.05619 -3.57666
H 0.61687 3.15227 -2.48580
H 1.82522 2.94096 -3.77674
H 0.43835 4.00448 -4.04684
C 0.75716 0.55186 -3.77795
H 0.26729 -0.36559 -4.13565
H 1.76096 0.58982 -4.23854
H 0.90038 0.46580 -2.68932
C 0.00126 1.95593 -5.70917
H -0.55020 2.83574 -6.07786
H 1.04820 2.07444 -6.04465
H -0.39682 1.05917 -6.20865
C -2.98876 0.66573 -4.47583
C -4.27304 0.43326 -3.64272
H -4.88446 1.34532 -3.55859
H -4.89064 -0.33387 -4.14575
H -4.04333 0.08283 -2.62064
C -3.38773 1.27383 -5.83729
H -2.52877 1.47047 -6.49581
H -4.04441 0.55744 -6.36409
H -3.96352 2.20702 -5.72267
C -2.30529 -0.70208 -4.69531
H -3.03562 -1.39079 -5.15747
H -1.44403 -0.64297 -5.37887
H -1.97651 -1.16243 -3.74883

8

SCF (BP86) Energy = -1392.85822202
Enthalpy 0K = -1392.077791
Enthalpy 298K = -1392.033095
Free Energy 298K = -1392.148433
Lowest Frequency = 23.4026 cm^{-1}
Second Frequency = 38.6423 cm^{-1}
SCF (BP86-D3BJ) Energy = -1393.106437
SCF (FB) Energy = -1392.899301
SCF (DFB) Energy = -1392.90509
SCF (BS2) Energy = -2062.471272

C 2.83461 4.38591 0.49869
H 3.83161 4.69329 0.84081
C 2.57506 4.50396 -0.82070
H 3.37973 4.89938 -1.45446
C 1.29403 4.13313 -1.54417
H 1.33329 4.53883 -2.56899
H 0.42448 4.61783 -1.05717
C 1.90801 3.85388 1.57618
H 0.93032 4.37375 1.53194
H 2.33511 4.09486 2.56415
C 0.05142 2.14729 -0.55439
H -0.97196 2.46056 -0.80546
C 0.32868 2.00572 0.82838
H -0.50864 2.20235 1.51185
C 1.66116 2.30542 1.48961
H 1.65797 1.87404 2.50686
H 2.49678 1.84019 0.94540
C 1.03405 2.58561 -1.61851
H 1.99254 2.05609 -1.52102
H 0.62382 2.32516 -2.61128
Rh -0.16282 0.04177 0.01916
P -2.50575 0.26510 0.11932
P 2.04905 -0.97459 -0.08797
N -0.72296 -2.05381 -0.06666
C -1.96264 -2.45921 0.35692
C -2.35929 -3.80430 0.29425
H -3.35634 -4.08633 0.64301
C -1.46750 -4.76614 -0.19473
H -1.75673 -5.82017 -0.24454
C -0.20051 -4.35325 -0.62008
H 0.52180 -5.07074 -1.01849
C 0.14461 -2.99258 -0.55577
C -2.86519 -1.40496 0.92612
H -3.92321 -1.70536 0.86661
H -2.61856 -1.26473 1.99321
C 1.46342 -2.49553 -1.05872
H 2.20802 -3.30625 -1.08468
H 1.33071 -2.13157 -2.09275
C -3.37873 0.14936 -1.62909
C -2.36809 -0.52641 -2.58724
H -2.17952 -1.57818 -2.31801
H -2.78697 -0.51602 -3.61061
H -1.39860 0.00155 -2.59741
C -4.67362 -0.69080 -1.59974
H -5.43662 -0.29025 -0.91545
H -5.11407 -0.69632 -2.61364
H -4.47933 -1.74273 -1.33184
C -3.68470 1.56520 -2.16547
H -2.79357 2.21558 -2.16823
H -4.02771 1.48001 -3.21265
H -4.48685 2.06858 -1.60338
C -3.47086 1.47023 1.31471
C -2.92915 1.21911 2.74504
H -1.82708 1.19247 2.78631
H -3.26997 2.03896 3.40234
H -3.31308 0.28238 3.18177
C -3.20652 2.94740 0.93629
H -3.56625 3.20230 -0.07084
H -3.74994 3.59334 1.64974
H -2.14130 3.21698 1.00073
C -4.99333 1.21111 1.30638
H -5.25019 0.16249 1.53149
H -5.46158 1.83389 2.09076
H -5.46351 1.48990 0.35026
C 3.64386 -0.55641 -1.16758
C 3.16178 -0.35307 -2.62664

H   2.27594 0.29862 -2.69870
H   3.97302 0.12437 -3.20513
H   2.93398 -1.30712 -3.12991
C   4.33956 0.73695 -0.68593
H   4.69640 0.66990 0.35211
H   5.22546 0.91227 -1.32358
H   3.70369 1.63140 -0.77146
C   4.68067 -1.70261 -1.14987
H   4.26020 -2.67365 -1.45890
H   5.48157 -1.45760 -1.87177
H   5.16189 -1.82565 -0.16714
C   2.59242 -1.72774 1.64579
C   1.35254 -1.67019 2.57083
H   0.55592 -2.35536 2.24010
H   1.65298 -1.97620 3.59040
H   0.92454 -0.65419 2.62111
C   3.06412 -3.19416 1.54399
H   3.93713 -3.32426 0.88685
H   3.35945 -3.54063 2.55150
H   2.26112 -3.86655 1.19880
C   3.70814 -0.86928 2.28166
H   3.88464 -1.22860 3.31198
H   4.66563 -0.95078 1.74432
H   3.43200 0.19522 2.34989

HCCtBu
SCF (BP86) Energy = -234.586240585
Enthalpy 0K = -234.449584
Enthalpy 298K = -234.440795
Free Energy 298K = -234.480158
Lowest Frequency = 169.3417 cm^{-1}
Second Frequency = 169.4574 cm^{-1}
SCF (BP86-D3BJ) Energy = -234.6063448
SCF (FB) Energy = -234.5884013
SCF (DFB) Energy = -234.5888902
SCF (BS2) Energy = -234.647662

C   2.39701 0.00001 0.00004
H   3.47016 -0.00003 0.00003
C   1.17635 0.00003 0.00005
C -0.29938 0.00002 0.00004
C -0.81204 1.24798 0.76779
C -0.81201 -1.28898 0.69686
H -0.45850 -2.18995 0.16902
H -0.45806 -1.34117 1.73961
H -1.91621 -1.29967 0.70297
H -0.45855 1.24157 1.81198
H -1.91624 1.25862 0.77405
H -0.45817 2.17711 0.29151
C -0.81192 0.04094 -1.46473
H -0.45806 -0.83615 -2.03114
H -1.91612 0.04120 -1.47711
H -0.45825 0.94842 -1.98121

A_C
SCF (BP86) Energy = -1080.79612092
Enthalpy 0K = -1080.194471
Enthalpy 298K = -1080.158071
Free Energy 298K = -1080.258904
Lowest Frequency = 24.9345 cm^{-1}
Second Frequency = 28.1670 cm^{-1}
SCF (BP86-D3BJ) Energy = -1080.977565
SCF (FB) Energy = -1080.844237
SCF (DFB) Energy = -1080.851914
SCF (BS2) Energy = -1750.339368

Rh -0.00020 -0.43189 0.00068
P -2.31385 -0.25110 -0.13326
P   2.31373 -0.25129 0.13349
N -0.00013 1.55263 0.00051
C -1.12436 2.25687 -0.39461
C -1.13732 3.65867 -0.38500
H -2.04758 4.17598 -0.70027
C   0.00035 4.37570 -0.00005
H   0.00052 5.46918 -0.00025
C   1.13778 3.65846 0.38520
H   2.04821 4.17559 0.70027
C   1.12434 2.25666 0.39536
C -2.32245 1.48182 -0.88088
H -3.25133 2.04940 -0.70831
H -2.22842 1.33103 -1.97068
C   2.32205 1.48131 0.88195
H   3.25110 2.04899 0.71062
H   2.22702 1.32963 1.97155
C -3.34269 -0.09742 1.49588
C -2.48842 0.77891 2.44301
H -2.36653 1.80802 2.06347
H -2.99607 0.84822 3.42228
H -1.48460 0.34859 2.59964
C -4.72341 0.56358 1.30128
H -5.39354 -0.02048 0.65419
H -5.21453 0.65376 2.28742
H -4.64764 1.58470 0.89125
C -3.50271 -1.50189 2.11851
H -2.53101 -2.01150 2.24027
H -3.95204 -1.40207 3.12307
H -4.16934 -2.15157 1.52828
C -3.10274 -1.44681 -1.43138
C -2.50228 -1.08849 -2.81094
H -1.40455 -0.96504 -2.76181
H -2.71744 -1.90958 -3.51814
H -2.94464 -0.17326 -3.23914
C -2.60900 -2.86214 -1.04083
H -2.96968 -3.18473 -0.05162
H -2.97223 -3.59514 -1.78368
H -1.50365 -2.91710 -1.04205
C -4.64069 -1.41435 -1.51106
H -5.02709 -0.40506 -1.73124
H -4.97279 -2.07796 -2.33055
H -5.11821 -1.77848 -0.58743
C   3.10428 -1.44695 1.43060
C   2.50430 -1.08963 2.81063
H   1.40647 -0.96686 2.76220
H   2.72033 -1.91092 3.51735
H   2.94631 -0.17431 3.23900
C   2.61142 -2.86254 1.03978
H   2.97173 -3.18445 0.05021
H   2.97575 -3.59553 1.78210
H   1.50613 -2.91840 1.04168
C   4.64224 -1.41335 1.50956
H   5.02802 -0.40380 1.72964
H   4.97517 -2.07679 2.32885
H   5.11961 -1.77705 0.58570
C   3.34130 -0.09665 -1.49639
C   2.48589 0.77919 -2.44294
H   2.36379 1.80825 -2.06335

H   2.99281 0.84865 -3.42257
H   1.48216 0.34836 -2.59876
C   4.72166 0.56534 -1.30255
H   5.39261 -0.01830 -0.65592
H   5.21214 0.65598 -2.28896
H   4.64536 1.58635 -0.89236
C   3.50193 -1.50092 -2.11932
H   3.95051 -1.40062 -3.12417
H   4.16942 -2.15020 -1.52965
H   2.53052 -2.01123 -2.24052

B_{C-tBu}
SCF (BP86) Energy = -1315.42848687
Enthalpy 0K = -1314.686830
Enthalpy 298K = -1314.641976
Free Energy 298K = -1314.758068
Lowest Frequency = 21.0087 cm^{-1}
Second Frequency = 32.1843 cm^{-1}
SCF (BP86-D3BJ) Energy = -1315.662926
SCF (FB) Energy = -1315.469708
SCF (DFB) Energy = -1315.475431
SCF (BS2) Energy = -1985.025037

Rh 0.02956 0.15491 0.10794
P   2.39016 -0.17053 0.01237
P -2.31358 -0.30501 0.21738
N   0.08858 -1.93374 -0.41823
C   1.19585 -2.48121 -1.01430
C   1.23904 -3.83680 -1.37821
H   2.13744 -4.23410 -1.85807
C   0.13058 -4.65520 -1.13343
H   0.14331 -5.71092 -1.42057
C -0.98706 -4.10297 -0.49731
H -1.85931 -4.71681 -0.25738
C -0.97880 -2.74694 -0.13312
C   2.35607 -1.56518 -1.26895
H   3.30249 -2.12626 -1.33390
H   2.20764 -1.05693 -2.23741
C -2.09796 -2.13634 0.65203
H -3.02575 -2.72175 0.56054
H -1.80699 -2.13697 1.71721
C   3.10029 -1.00239 1.63004
C   1.92596 -1.72209 2.33634
H   1.56459 -2.59100 1.76286
H   2.27679 -2.09541 3.31624
H   1.07142 -1.04373 2.50274
C   4.21034 -2.03762 1.34977
H   5.08618 -1.61118 0.83987
H   4.56111 -2.45010 2.31366
H   3.83981 -2.88981 0.75557
C   3.62393 0.10562 2.57119
H   2.86793 0.88873 2.75181
H   3.87332 -0.34480 3.54909
H   4.53960 0.58646 2.19319
C   3.69583 1.03667 -0.77554
C   3.26869 1.25231 -2.24846
H   2.18266 1.41147 -2.35523
H   3.77781 2.15072 -2.64122
H   3.56680 0.41036 -2.89516
C   3.62324 2.38402 -0.02321
H   3.97195 2.30683 1.01766
H   4.27133 3.11898 -0.53451
H   2.59739 2.78095 -0.00613
C   5.14334 0.50269 -0.75836
H   5.23954 -0.48202 -1.24622
H   5.78307 1.20667 -1.32222
H   5.56014 0.43382 0.25849
C -3.45897 0.27272 1.67937
C -2.72597 -0.07557 2.99821
H -1.67207 0.24893 2.98431
H -3.22846 0.44949 3.83039
H -2.76119 -1.15190 3.23510
C -3.62199 1.80649 1.61381
H -4.12121 2.14546 0.69414
H -4.24223 2.13670 2.46715
H -2.64625 2.31130 1.68688
C -4.83990 -0.41613 1.67117
H -4.76621 -1.51639 1.70361
H -5.39758 -0.10364 2.57329
H -5.45156 -0.13319 0.80011
C -3.28275 -0.38671 -1.48466
C -2.20027 -0.43551 -2.58974
H -1.59997 -1.35883 -2.54398
H -2.69269 -0.40818 -3.57949
H -1.50938 0.42249 -2.52160
C -4.17921 -1.63854 -1.60209
H -4.94057 -1.69985 -0.80862
H -4.71623 -1.59833 -2.56745
H -3.59421 -2.57300 -1.60290
C -4.14447 0.87569 -1.69482
H -4.57206 0.84606 -2.71362
H -4.98843 0.93989 -0.99037
H -3.55465 1.80256 -1.61760
C   0.11034 1.77560 1.42902
H   0.28547 1.74738 2.49986
C -0.05511 2.38386 0.32594
C -0.26218 3.62740 -0.49992
C   0.28075 3.48099 -1.93918
C   0.46749 4.79630 0.22156
H   0.08102 4.93259 1.24486
H   1.55408 4.62606 0.28214
H   0.29992 5.73361 -0.33695
H   1.37429 3.35913 -1.94766
H   0.04059 4.38749 -2.52026
H -0.17115 2.61393 -2.44995
C -1.77013 3.97737 -0.57226
H -2.19312 4.15347 0.42898
H -1.90300 4.89680 -1.16781
H -2.34371 3.17378 -1.05875

TS(B-10)$_{C-tBu}$
SCF (BP86) Energy = -1315.36208646
Enthalpy 0K = -1314.624048
Enthalpy 298K = -1314.579593
Free Energy 298K = -1314.694761
Lowest Frequency = -595.8778 cm^{-1}
Second Frequency = 24.5848 cm^{-1}
SCF (BP86-D3BJ) Energy = -1315.595362
SCF (FB) Energy = -1315.404228
SCF (DFB) Energy = -1315.410234
SCF (BS2) Energy = -1984.957568

Rh 0.05131 0.14752 0.10433
P   2.40033 -0.16280 0.02204
P -2.29902 -0.32117 0.23109
N   0.11314 -1.88862 -0.52659

C	1.22822	-2.41130	-1.13462
C	1.27725	-3.74825	-1.55776
H	2.17984	-4.12000	-2.05013
C	0.17130	-4.58238	-1.35509
H	0.18794	-5.62321	-1.69143
C	-0.94461	-4.06449	-0.68811
H	-1.81118	-4.69351	-0.46739
C	-0.94246	-2.72658	-0.26323
C	2.39190	-1.48495	-1.33038
H	3.33892	-2.04318	-1.41092
H	2.25363	-0.92328	-2.27072
C	-2.04735	-2.16545	0.57651
H	-2.97062	-2.75706	0.48544
H	-1.72876	-2.20778	1.63307
C	3.03977	-1.06166	1.63325
C	1.83162	-1.79622	2.26374
H	1.48577	-2.63869	1.64360
H	2.14455	-2.21096	3.23985
H	0.98293	-1.11066	2.43093
C	4.15294	-2.09441	1.35772
H	5.06257	-1.65400	0.92502
H	4.44154	-2.56467	2.31564
H	3.80823	-2.90679	0.69583
C	3.52073	0.00702	2.64046
H	2.74683	0.77173	2.82721
H	3.73222	-0.48640	3.60639
H	4.45017	0.50629	2.32423
C	3.74707	1.07065	-0.65867
C	3.39183	1.36415	-2.13624
H	2.31669	1.56228	-2.27879
H	3.94491	2.26200	-2.46627
H	3.69085	0.54221	-2.80724
C	3.68547	2.39210	0.14161
H	3.85221	2.25472	1.22006
H	4.47089	3.07504	-0.23052
H	2.72095	2.90644	0.00192
C	5.18158	0.50296	-0.60096
H	5.27317	-0.47039	-1.11126
H	5.85782	1.20613	-1.12142
H	5.55689	0.39672	0.42863
C	-3.35066	0.18963	1.78775
C	-2.51242	-0.14531	3.04551
H	-1.49906	0.28574	2.99038
H	-3.01760	0.29377	3.92492
H	-2.43753	-1.22922	3.23539
C	-3.58536	1.71630	1.77329
H	-4.16517	2.05485	0.90187
H	-4.15614	1.99628	2.67732
H	-2.62944	2.26481	1.80681
C	-4.69768	-0.56174	1.85490
H	-4.57010	-1.65733	1.87042
H	-5.21176	-0.28471	2.79345
H	-5.37521	-0.30190	1.02646
C	-3.38028	-0.34569	-1.40955
C	-2.37168	-0.27168	-2.58000
H	-1.69952	-1.14519	-2.60281
H	-2.92711	-0.25044	-3.53595
H	-1.74017	0.63045	-2.52296
C	-4.21008	-1.64176	-1.54404
H	-4.90065	-1.80003	-0.70020
H	-4.82535	-1.57102	-2.45958
H	-3.57551	-2.53622	-1.65538
C	-4.33685	0.86379	-1.48574
H	-4.80815	0.87578	-2.48555
H	-5.15082	0.80606	-0.74605
H	-3.82057	1.82637	-1.35762
C	-0.05823	1.76829	1.42654
H	0.97998	2.50113	0.98025
C	-0.03635	2.40421	0.25681
C	-0.32158	3.64545	-0.57697
C	0.51305	3.68295	-1.87569
C	-0.00021	4.88511	0.30144
H	-0.60456	4.89050	1.22269
H	1.06601	4.91527	0.58548
H	-0.22138	5.80430	-0.26666
H	1.59265	3.75746	-1.67092
H	0.22560	4.56743	-2.46874
H	0.33205	2.78393	-2.48778
C	-1.82003	3.65577	-0.95424
H	-2.46685	3.62432	-0.06428
H	-2.04108	4.58280	-1.50958
H	-2.06339	2.80414	-1.60821

TS(B–C)$_{C\text{-}tBu}$

SCF (BP86) Energy = -1315.41692298
Enthalpy 0K = -1314.678080
Enthalpy 298K = -1314.633086
Free Energy 298K = -1314.751863
Lowest Frequency = -143.3884 cm^{-1}
Second Frequency = 13.1341 cm^{-1}
SCF (BP86-D3BJ) Energy = -1315.644376
SCF (FB) Energy = -1315.457148
SCF (DFB) Energy = -1315.462639
SCF (BS2) Energy = -1985.01344

Rh	0.05616	-0.05106	0.18849
P	2.40589	-0.00807	0.02443
P	-2.19184	-0.72892	0.22979
N	0.35070	-2.05620	-0.42557
C	1.52693	-2.44864	-1.02108
C	1.74124	-3.77264	-1.43314
H	2.69388	-4.03657	-1.90067
C	0.73287	-4.72850	-1.26508
H	0.87977	-5.76172	-1.59247
C	-0.46642	-4.33033	-0.66356
H	-1.27666	-5.04559	-0.49725
C	-0.62939	-3.00312	-0.23843
C	2.56086	-1.38504	-1.25962
H	3.57208	-1.81625	-1.33130
H	2.34330	-0.89501	-2.22522
C	-1.86346	-2.57017	0.49991
H	-2.72404	-3.21483	0.26149
H	-1.67169	-2.66667	1.58337
C	3.34864	-0.70011	1.58423
C	2.36656	-1.65972	2.29843
H	2.12288	-2.54059	1.68223
H	2.83883	-2.02456	3.22935
H	1.41962	-1.15678	2.55959
C	4.63180	-1.48098	1.22820
H	5.37157	-0.87560	0.68343
H	5.11144	-1.81958	2.16492
H	4.41700	-2.38617	0.63640
C	3.68905	0.45992	2.54497
H	2.81296	1.09502	2.76326
H	4.03723	0.03860	3.50556
H	4.49847	1.10052	2.16016
C	3.36611	1.45166	-0.81886

C	2.67080	1.69378	-2.18119
H	1.57196	1.73293	-2.07996
H	3.01304	2.66203	-2.58951
H	2.92526	0.92689	-2.93167
C	3.20715	2.72845	0.03787
H	3.71196	2.65461	1.01210
H	3.66463	3.57741	-0.50291
H	2.14648	2.96565	0.21488
C	4.86242	1.15668	-1.05028
H	5.03147	0.22163	-1.61065
H	5.30111	1.97735	-1.64751
H	5.42927	1.10662	-0.10687
C	-3.34668	-0.30088	1.73245
C	-2.55082	-0.65086	3.01419
H	-1.53102	-0.22822	2.99587
H	-3.07797	-0.22585	3.88718
H	-2.47317	-1.73694	3.18790
C	-3.63743	1.21661	1.75255
H	-4.24269	1.54508	0.89505
H	-4.20917	1.45576	2.66781
H	-2.70973	1.80947	1.76659
C	-4.67196	-1.09051	1.71743
H	-4.51741	-2.18115	1.65903
H	-5.22065	-0.88973	2.65604
H	-5.32997	-0.78569	0.88792
C	-3.13519	-0.67440	-1.47545
C	-2.03898	-0.74013	-2.56612
H	-1.49479	-1.69877	-2.55102
H	-2.51540	-0.64363	-3.55947
H	-1.29905	0.07045	-2.44932
C	-4.11218	-1.85233	-1.67711
H	-4.89373	-1.90472	-0.90396
H	-4.62173	-1.72704	-2.65017
H	-3.59152	-2.82340	-1.71459
C	-3.89366	0.66184	-1.61655
H	-4.25469	0.76201	-2.65646
H	-4.77658	0.71579	-0.95965
H	-3.24464	1.52893	-1.40608
C	-0.20547	1.86723	0.90879
H	0.06844	1.62847	1.95203
C	-0.55449	2.93071	0.32296
C	-0.95549	4.16750	-0.35454
C	-0.66609	4.03191	-1.87973
C	-0.09786	5.33400	0.22841
H	-0.26825	5.45416	1.31041
H	0.97705	5.16676	0.05695
H	-0.39065	6.27269	-0.27171
H	0.40426	3.85860	-2.07032
H	-0.96283	4.97026	-2.37803
H	-1.24289	3.20473	-2.32383
C	-2.46218	4.46748	-0.12877
H	-2.69092	4.57346	0.94389
H	-2.72912	5.41195	-0.63225
H	-3.09607	3.66686	-0.54215

TS(C-D)$_{C\text{-}tBu}$

SCF (BP86) Energy = -1315.36375662
Enthalpy 0K = -1314.625498
Enthalpy 298K = -1314.580950
Free Energy 298K = -1314.697117
Lowest Frequency = -280.8519 cm^{-1}
Second Frequency = 12.2741 cm^{-1}
SCF (BP86-D3BJ) Energy = -1315.595234
SCF (FB) Energy = -1315.407748
SCF (DFB) Energy = -1315.414368
SCF (BS2) Energy = -1984.960123

Rh	-0.00824	0.03417	-0.01530
P	2.35048	-0.30849	-0.07934
P	-2.38664	-0.26789	0.07017
N	-0.04254	-2.08401	-0.04720
C	1.07810	-2.78528	-0.42741
C	1.10904	-4.18841	-0.40030
H	2.01977	-4.70656	-0.71166
C	-0.02496	-4.90184	0.00040
H	-0.01882	-5.99565	0.02053
C	-1.16860	-4.18735	0.37076
H	-2.07558	-4.70518	0.69386
C	-1.15647	-2.78370	0.34693
C	2.24886	-1.99676	-0.92338
H	3.18223	-2.57737	-0.85627
H	2.08305	-1.75863	-1.98900
C	-2.35718	-2.00553	0.79948
H	-3.28569	-2.56756	0.60629
H	-2.29227	-1.86036	1.89377
C	3.13606	-0.68325	1.66413
C	1.97229	-1.13484	2.57959
H	1.53862	-2.09648	2.25912
H	2.35845	-1.27208	3.60651
H	1.16159	-0.38536	2.60940
C	4.20175	-1.79879	1.62552
H	5.05286	-1.56302	0.96944
H	4.60344	-1.93847	2.64581
H	3.77794	-2.76848	1.31499
C	3.74277	0.61311	2.24350
H	3.02503	1.45083	2.23097
H	4.02336	0.43449	3.29738
H	4.65668	0.92333	1.71280
C	3.57370	0.61658	-1.27661
C	2.95889	0.55760	-2.69657
H	1.90070	0.87487	-2.70345
H	3.52491	1.25063	-3.34485
H	3.04383	-0.44075	-3.15792
C	3.68045	2.09798	-0.86527
H	4.11491	2.24060	0.13526
H	4.33531	2.62032	-1.58602
H	2.69367	2.58160	-0.89746
C	4.97535	-0.03068	-1.28472
H	4.95146	-1.10606	-1.52853
H	5.58212	0.46329	-2.06537
H	5.50781	0.10234	-0.32938
C	-3.48890	0.65685	1.37780
C	-2.57141	0.96841	2.58389
H	-1.75208	1.65753	2.32190
H	-3.17270	1.45030	3.37628
H	-2.12075	0.06120	3.02294
C	-4.02356	1.97624	0.78137
H	-4.80375	1.80050	0.02380
H	-4.48312	2.57477	1.58889
H	-3.22785	2.58611	0.32548
C	-4.67561	-0.20331	1.87223
H	-4.35169	-1.11042	2.40831
H	-5.26382	0.39726	2.58998
H	-5.35665	-0.50285	1.06247
C	-3.27853	-0.46610	-1.64127
C	-2.44423	-1.49699	-2.43812
H	-2.47659	-2.50722	-1.99553

H	-2.86518	-1.57439	-3.45683
H	-1.39278	-1.17775	-2.53154
C	-4.73301	-0.96253	-1.51563
H	-5.39512	-0.21036	-1.05829
H	-5.12454	-1.16221	-2.52988
H	-4.82311	-1.90357	-0.94582
C	-3.23368	0.88018	-2.39884
H	-3.63349	0.72043	-3.41693
H	-3.85175	1.65608	-1.92247
H	-2.20082	1.25700	-2.49110
C	-0.08892	1.63649	-1.40745
H	-0.60626	1.78107	0.55776
C	-0.16397	2.41432	-0.32716
C	0.14461	3.86137	0.10176
C	0.71169	4.64229	-1.10689
C	1.13222	3.88284	1.29065
H	0.72932	3.32733	2.15621
H	2.10626	3.44589	1.02232
H	1.30593	4.92390	1.61336
H	1.67193	4.23059	-1.45435
H	0.87601	5.69600	-0.82330
H	0.00686	4.61990	-1.95400
C	-1.17769	4.53780	0.54729
H	-1.61863	4.03246	1.42382
H	-0.98405	5.58727	0.83062
H	-1.92009	4.53863	-0.26803

C_{C-tBu}

SCF (BP86) Energy = -1315.43251607
Enthalpy 0K = -1314.693645
Enthalpy 298K = -1314.648370
Free Energy 298K = -1314.768392
Lowest Frequency = 8.3428 cm^{-1}
Second Frequency = 15.9817 cm^{-1}
SCF (BP86-D3BJ) Energy = -1315.657877
SCF (FB) Energy = -1315.473546
SCF (DFB) Energy = -1315.479325
SCF (BS2) Energy = -1985.031789

Rh	0.01978	-0.18737	0.08799
P	2.35959	-0.31827	-0.01394
P	-2.29113	-0.56620	0.13965
N	0.12773	-2.27812	-0.16500
C	1.29753	-2.87914	-0.56944
C	1.37804	-4.26627	-0.76730
H	2.32816	-4.70447	-1.08482
C	0.24702	-5.06624	-0.57565
H	0.29367	-6.14766	-0.73394
C	-0.94520	-4.45292	-0.17618
H	-1.85017	-5.04255	-0.00643
C	-0.98353	-3.06608	0.03306
C	2.49523	-2.00645	-0.84641
H	3.43131	-2.53307	-0.59400
H	2.53588	-1.79719	-1.93157
C	-2.24023	-2.40995	0.54122
H	-3.13598	-2.95312	0.19603
H	-2.24139	-2.46307	1.64611
C	3.23933	-0.53512	1.69483
C	2.60461	-1.78334	2.35190
H	2.85962	-2.71712	1.82292
H	2.99029	-1.87678	3.38324
H	1.50585	-1.69734	2.40582
C	4.76520	-0.72816	1.58750
H	5.28047	0.18157	1.24062
H	5.16644	-0.96627	2.58960
H	5.04327	-1.56302	0.92160
C	2.91506	0.69674	2.57051
H	1.82689	0.81809	2.70357
H	3.36197	0.55365	3.57102
H	3.31973	1.63358	2.15775
C	3.33428	0.82195	-1.23343
C	2.39233	1.04263	-2.44129
H	1.44423	1.51205	-2.13534
H	2.89579	1.70540	-3.16883
H	2.15663	0.10313	-2.97222
C	3.60813	2.18061	-0.55292
H	4.34751	2.09966	0.26034
H	4.02557	2.87794	-1.30207
H	2.68346	2.62850	-0.15256
C	4.65892	0.19857	-1.72741
H	4.50760	-0.75452	-2.26123
H	5.12656	0.89540	-2.44679
H	5.38361	0.03003	-0.91764
C	-3.38994	0.13739	1.56870
C	-2.49214	0.18917	2.82784
H	-1.64799	0.88567	2.70360
H	-3.09918	0.53940	3.68249
H	-2.08041	-0.79758	3.10246
C	-3.84174	1.57090	1.21576
H	-4.58706	1.58486	0.40417
H	-4.31935	2.02419	2.10336
H	-2.99187	2.21100	0.92610
C	-4.62015	-0.74994	1.86584
H	-4.34171	-1.76424	2.19691
H	-5.19310	-0.29053	2.69180
H	-5.30081	-0.84096	1.00691
C	-3.14067	-0.45142	-1.59497
C	-2.33299	-1.38672	-2.52642
H	-2.43056	-2.45065	-2.25197
H	-2.71721	-1.27797	-3.55680
H	-1.26111	-1.12334	-2.53670
C	-4.62175	-0.87832	-1.59564
H	-5.26123	-0.17443	-1.03969
H	-4.98842	-0.89468	-2.63840
H	-4.77473	-1.89063	-1.18362
C	-2.99512	0.99693	-2.11444
H	-3.35185	1.03889	-3.15975
H	-3.59227	1.71561	-1.53276
H	-1.94384	1.32729	-2.09310
C	-0.10227	1.82555	0.14820
H	0.00265	1.16002	1.16690
C	-0.20064	3.06742	0.05724
C	-0.29420	4.52639	-0.12955
C	0.17328	4.87753	-1.57187
C	0.63204	5.23312	0.90101
H	0.32998	4.99848	1.93490
H	1.68316	4.92931	0.76744
H	0.56825	6.32610	0.76346
H	1.21574	4.56271	-1.74074
H	0.11254	5.96944	-1.71928
H	-0.46544	4.39126	-2.32719
C	-1.75684	5.00563	0.07284
H	-2.11178	4.77908	1.09178
H	-1.81628	6.09752	-0.07486
H	-2.43582	4.52403	-0.64985

$TS(C\text{-}10)_{C-tBu}$

SCF (BP86) Energy = -1315.41521036
Enthalpy 0K = -1314.678637
Enthalpy 298K = -1314.633195
Free Energy 298K = -1314.752998
Lowest Frequency = -789.3127 cm^{-1}
Second Frequency = 12.4710 cm^{-1}
SCF (BP86-D3BJ) Energy = -1315.639942
SCF (FB) Energy = -1315.455734
SCF (DFB) Energy = -1315.46151764
SCF (BS2) Energy = -1985.012905

Rh	-0.00084	-0.16828	0.03687
P	2.31618	-0.43715	-0.05461
P	-2.31802	-0.44190	0.09179
N	-0.00115	-2.29063	-0.04013
C	1.13887	-2.98365	-0.37358
C	1.16060	-4.38589	-0.42756
H	2.09137	-4.89459	-0.69342
C	-0.00524	-5.11264	-0.16497
H	-0.00638	-6.20558	-0.21155
C	-1.17036	-4.40828	0.15577
H	-2.10346	-4.93574	0.37229
C	-1.14559	-3.00663	0.22265
C	2.36805	-2.19424	-0.74223
H	3.28633	-2.73992	-0.46610
H	2.38949	-2.07379	-1.84126
C	-2.37887	-2.24776	0.63852
H	-3.29293	-2.76871	0.30626
H	-2.41602	-2.21522	1.74327
C	3.20027	-0.56261	1.66412
C	2.49736	-1.71187	2.42457
H	2.69560	-2.70003	1.97651
H	2.87964	-1.74014	3.46122
H	1.40518	-1.55819	2.46699
C	4.71182	-0.85126	1.57705
H	5.27820	-0.00592	1.15471
H	5.10327	-1.02726	2.59591
H	4.93812	-1.75377	0.98394
C	2.94959	0.75221	2.43681
H	1.86823	0.93768	2.55461
H	3.38955	0.66856	3.44730
H	3.40490	1.62829	1.94897
C	3.36621	0.53126	-1.36240
C	2.44095	0.71405	-2.58923
H	1.51297	1.24550	-2.32506
H	2.97757	1.29825	-3.35943
H	2.15824	-0.24786	-3.05161
C	3.73369	1.92055	-0.79873
H	4.46915	1.85973	0.01983
H	4.19078	2.52685	-1.60214
H	2.84312	2.45937	-0.43478
C	4.64678	-0.21280	-1.80276
H	4.43162	-1.19551	-2.25415
H	5.15572	0.38924	-2.57777
H	5.36271	-0.35981	-0.98105
C	-3.40621	0.41368	1.44758
C	-2.52280	0.50085	2.71467
H	-1.60241	1.07924	2.53559
H	-3.09560	0.99707	3.51945
H	-2.22234	-0.49173	3.09265
C	-3.77167	1.84150	0.98872
H	-4.49463	1.83928	0.15692
H	-4.24393	2.38241	1.82905
H	-2.88133	2.41274	0.67743
C	-4.69151	-0.37314	1.79044
H	-4.47943	-1.38306	2.17878
H	-5.23475	0.16748	2.58713
H	-5.37622	-0.46912	0.93549
C	-3.15909	-0.42404	-1.65250
C	-2.41587	-1.49050	-2.49145
H	-2.59982	-2.51704	-2.13221
H	-2.77759	-1.43855	-3.53451
H	-1.32688	-1.31075	-2.49802
C	-4.66655	-0.74370	-1.63466
H	-5.25971	0.05029	-1.15345
H	-5.02728	-0.83115	-2.67606
H	-4.89200	-1.70080	-1.13364
C	-2.90722	0.95923	-2.29463
H	-3.28030	0.94724	-3.33508
H	-3.42779	1.77306	-1.76620
H	-1.82993	1.19331	-2.31757
C	-0.00574	1.78792	0.03446
H	0.11054	2.06216	1.18098
C	-0.01246	3.06484	0.01283
C	0.00011	4.54002	-0.01826
C	0.06209	4.92769	-1.52930
C	1.25429	5.09037	0.71359
H	1.24488	4.81698	1.78191
H	2.18320	4.70794	0.26127
H	1.26343	6.19109	0.64503
H	0.97594	4.54048	-2.00706
H	0.06872	6.02820	-1.60464
H	-0.81442	4.54915	-2.07908
C	-1.28791	5.12519	0.61916
H	-1.36843	4.84732	1.68309
H	-1.26332	6.22614	0.55618
H	-2.18955	4.77003	0.09489

$\mathbf{D}_{C\text{-}tBu}$

SCF (BP86) Energy = -1315.36477713
Enthalpy 0K = -1314.625878
Enthalpy 298K = -1314.581042
Free Energy 298K = -1314.697676
Lowest Frequency = 10.4549 cm^{-1}
Second Frequency = 31.3418 cm^{-1}
SCF (BP86-D3BJ) Energy = -1315.596551
SCF (FB) Energy = -1315.409167
SCF (DFB) Energy = -1315.415953
SCF (BS2) Energy = -1984.961713

Rh	-0.00042	0.04022	0.02809
P	2.37557	-0.24572	-0.04501
P	-2.36095	-0.31604	0.10572
N	0.03537	-2.06646	-0.18151
C	1.17001	-2.70638	-0.62130
C	1.23116	-4.10533	-0.71176
H	2.15050	-4.57605	-1.06963
C	0.11500	-4.87413	-0.36442
H	0.14472	-5.96535	-0.43710
C	-1.03898	-4.22022	0.07802
H	-1.92989	-4.78408	0.36671
C	-1.05664	-2.81902	0.17143
C	2.32131	-1.84864	-1.04227
H	3.26846	-2.41064	-1.02908
H	2.14663	-1.51243	-2.07985
C	-2.25899	-2.10365	0.71248

H	-3.18078	-2.67551	0.51717
H	-2.15763	-2.02755	1.81104
C	3.09956	-0.75316	1.69053
C	1.91777	-1.31419	2.51920
H	1.53653	-2.26537	2.11292
H	2.26582	-1.51092	3.55007
H	1.07735	-0.59758	2.56952
C	4.20035	-1.83114	1.60472
H	5.07671	-1.51058	1.02265
H	4.55180	-2.06036	2.62749
H	3.82335	-2.77587	1.17815
C	3.63685	0.50876	2.40128
H	2.88544	1.31597	2.43923
H	3.89424	0.24816	3.44394
H	4.55096	0.90225	1.92974
C	3.63054	0.80615	-1.09404
C	3.13189	0.79870	-2.56032
H	2.05728	1.04359	-2.63465
H	3.69482	1.56956	-3.11708
H	3.32774	-0.16044	-3.06877
C	3.61823	2.26386	-0.58831
H	3.93856	2.36169	0.45994
H	4.31889	2.85715	-1.20348
H	2.61533	2.70132	-0.69847
C	5.06366	0.23563	-1.03326
H	5.11812	-0.82204	-1.34193
H	5.69399	0.80941	-1.73707
H	5.52023	0.33580	-0.03567
C	-3.48188	0.46231	1.49248
C	-2.52924	0.81597	2.65807
H	-1.78568	1.57509	2.36638
H	-3.12052	1.22255	3.49878
H	-1.98182	-0.06443	3.03917
C	-4.17325	1.74277	0.98054
H	-4.97471	1.51979	0.25840
H	-4.64217	2.26117	1.83662
H	-3.46851	2.44532	0.51227
C	-4.56080	-0.52160	2.00373
H	-4.13451	-1.40152	2.51204
H	-5.18255	0.00544	2.75017
H	-5.23475	-0.87143	1.20721
C	-3.26802	-0.42986	-1.60355
C	-2.39657	-1.34682	-2.49362
H	-2.36142	-2.38739	-2.12828
H	-2.83877	-1.37231	-3.50597
H	-1.36875	-0.95761	-2.58382
C	-4.69057	-1.01406	-1.48579
H	-5.37564	-0.34944	-0.93605
H	-5.10507	-1.13523	-2.50322
H	-4.71124	-2.01017	-1.01083
C	-3.31048	0.97232	-2.25207
H	-3.72396	0.87033	-3.27208
H	-3.95723	1.67486	-1.70549
H	-2.29849	1.40328	-2.33624
C	-0.01802	1.58178	-1.46678
H	-0.08100	1.69252	0.62340
C	-0.04184	2.38517	-0.41890
C	-0.06044	3.86220	-0.00433
C	0.43187	4.68454	-1.22232
C	0.85057	4.11342	1.22003
H	0.51699	3.52583	2.09464
H	1.90093	3.85696	1.01211
H	0.81256	5.17926	1.50229
H	1.46800	4.42793	-1.49407
H	0.39558	5.76031	-0.97859
H	-0.20696	4.50635	-2.10246
C	-1.49957	4.30780	0.35184
H	-1.87851	3.77527	1.24039
H	-1.51047	5.38703	0.58524
H	-2.18943	4.13270	-0.48998

TS(C-E)$_{C-tBu}$
SCF (BP86) Energy = -1315.42880900
Enthalpy 0K = -1314.691619
Enthalpy 298K = -1314.646611
Free Energy 298K = -1314.765219
Lowest Frequency = -642.3505 cm^{-1}
Second Frequency = 15.5195 cm^{-1}
SCF (BP86-D3BJ) Energy = -1315.655218
SCF (FB) Energy = -1315.471692
SCF (DFB) Energy = -1315.47812
SCF (BS2) Energy = -1985.027935

Rh	0.00849	-0.17359	-0.00867
P	-2.32329	-0.47347	0.05735
P	2.34308	-0.39595	-0.09672
N	0.03912	-2.30854	-0.00318
C	-1.09168	-3.02164	0.31300
C	-1.08620	-4.42498	0.33603
H	-2.00594	-4.96034	0.58709
C	0.09606	-5.12015	0.05824
H	0.11772	-6.21371	0.08004
C	1.25051	-4.39039	-0.24785
H	2.19089	-4.89884	-0.47739
C	1.19994	-2.98846	-0.28374
C	-2.33546	-2.25576	0.69330
H	-3.24317	-2.80575	0.39249
H	-2.37269	-2.17232	1.79567
C	2.40991	-2.18847	-0.69559
H	3.34110	-2.70357	-0.40553
H	2.41844	-2.11551	-1.79957
C	-3.20613	-0.55456	-1.65829
C	-2.48644	-1.66258	-2.46297
H	-2.65401	-2.66871	-2.04296
H	-2.88616	-1.66835	-3.49302
H	-1.39970	-1.48222	-2.52173
C	-4.70878	-0.88472	-1.55233
H	-5.28499	-0.06849	-1.08881
H	-5.11387	-1.03162	-2.57012
H	-4.90361	-1.81401	-0.99008
C	-3.00570	0.79404	-2.38557
H	-1.93727	1.04144	-2.49691
H	-3.44273	0.72062	-3.39781
H	-3.49827	1.63286	-1.87126
C	-3.33698	0.46313	1.40866
C	-2.37417	0.62428	2.60991
H	-1.48222	1.20996	2.33505
H	-2.90374	1.15508	3.42204
H	-2.04405	-0.34576	3.02384
C	-3.71703	1.86309	0.87993
H	-4.48073	1.81677	0.08660
H	-4.14699	2.45238	1.71019
H	-2.83496	2.40513	0.50030
C	-4.60186	-0.29986	1.86093
H	-4.37196	-1.28688	2.29601
H	-5.10006	0.28883	2.65263
H	-5.33207	-0.44051	1.05039

C	3.41263	0.52735	-1.41402
C	2.52892	0.68475	-2.67404
H	1.66487	1.34087	-2.48474
H	3.13597	1.14370	-3.47539
H	2.15426	-0.27918	-3.06093
C	3.78246	1.92999	-0.88562
H	4.52188	1.88731	-0.06977
H	4.23975	2.50990	-1.70768
H	2.89468	2.48224	-0.53617
C	4.69096	-0.25819	-1.78714
H	4.46972	-1.23128	-2.25633
H	5.25907	0.33193	-2.52884
H	5.35592	-0.43061	-0.92807
C	3.14323	-0.40871	1.66370
C	2.37115	-1.47696	2.47490
H	2.52052	-2.49905	2.08820
H	2.73738	-1.46318	3.51729
H	1.28705	-1.26845	2.50205
C	4.64571	-0.75309	1.66235
H	5.25490	0.03355	1.18995
H	4.99184	-0.84656	2.70806
H	4.86356	-1.71272	1.16273
C	2.90288	0.97115	2.31868
H	3.21223	0.92129	3.37854
H	3.48743	1.77179	1.84143
H	1.83916	1.25857	2.28077
C	-0.01916	1.79220	0.03300
H	-0.05794	0.79880	-1.22722
C	-0.04392	3.03568	-0.03208
C	-0.09709	4.51293	-0.00023
C	-0.51194	4.97529	1.42516
C	-1.14652	5.01499	-1.03074
H	-0.88137	4.69995	-2.05355
H	-2.15126	4.62540	-0.79749
H	-1.19206	6.11763	-1.01113
H	-1.50515	4.58199	1.69709
H	-0.55512	6.07766	1.46032
H	0.21501	4.63360	2.18017
C	1.29162	5.11191	-0.34979
H	1.61501	4.80311	-1.35774
H	1.24305	6.21441	-0.32895
H	2.05622	4.78978	0.37660

E_{C-tBu}

SCF (BP86) Energy = -1315.43634721
Enthalpy 0K = -1314.697420
Enthalpy 298K = -1314.652265
Free Energy 298K = -1314.770842
Lowest Frequency = 16.5130 cm^{-1}
Second Frequency = 19.8705 cm^{-1}
SCF (BP86-D3BJ) Energy = -1315.66336
SCF (FB) Energy = -1315.481579
SCF (DFB) Energy = -1315.488927
SCF (BS2) Energy = -1985.034525

Rh	0.00869	-0.18129	-0.00992
P	-2.32851	-0.45237	0.05546
P	2.34078	-0.42189	-0.09678
N	0.02045	-2.32493	0.02694
C	-1.12671	-3.02204	0.31015
C	-1.14448	-4.42560	0.31033
H	-2.07624	-4.95055	0.53831
C	0.03159	-5.13462	0.04022
H	0.03631	-6.22857	0.04612
C	1.20119	-4.41861	-0.23965
H	2.13647	-4.93853	-0.46477
C	1.17181	-3.01521	-0.25621
C	-2.36130	-2.23776	0.68728
H	-3.27621	-2.77488	0.38533
H	-2.39895	-2.15519	1.78988
C	2.39999	-2.22894	-0.65154
H	3.31913	-2.74307	-0.32354
H	2.44288	-2.18622	-1.75642
C	-3.24119	-0.49523	-1.64331
C	-2.59796	-1.63694	-2.46493
H	-2.78748	-2.63314	-2.03109
H	-3.03791	-1.63261	-3.47827
H	-1.50838	-1.50469	-2.57474
C	-4.75492	-0.75771	-1.50404
H	-5.28059	0.07649	-1.01360
H	-5.19095	-0.86854	-2.51345
H	-4.97811	-1.68662	-0.95154
C	-2.99006	0.84732	-2.36627
H	-1.91406	1.06771	-2.45611
H	-3.41701	0.78927	-3.38379
H	-3.46596	1.69724	-1.85429
C	-3.28493	0.50391	1.43342
C	-2.28428	0.63816	2.60744
H	-1.39703	1.22323	2.31278
H	-2.78023	1.16710	3.44168
H	-1.95417	-0.34020	3.00210
C	-3.64212	1.91425	0.91687
H	-4.42544	1.88853	0.14201
H	-4.03767	2.51113	1.75874
H	-2.75490	2.43349	0.51753
C	-4.55424	-0.23103	1.91728
H	-4.33629	-1.22297	2.34740
H	-5.01993	0.36910	2.72008
H	-5.30647	-0.35539	1.12446
C	3.39462	0.48259	-1.43535
C	2.49384	0.61714	-2.68567
H	1.61300	1.24551	-2.48071
H	3.07999	1.09346	-3.49235
H	2.14848	-0.35890	-3.07145
C	3.75684	1.89523	-0.92867
H	4.50073	1.86945	-0.11600
H	4.20678	2.46507	-1.76166
H	2.86497	2.44475	-0.58556
C	4.67457	-0.30092	-1.80547
H	4.45708	-1.28656	-2.24983
H	5.22867	0.27710	-2.56690
H	5.35113	-0.44760	-0.95044
C	3.13912	-0.39901	1.66392
C	2.37086	-1.45515	2.49458
H	2.52181	-2.48328	2.12533
H	2.73776	-1.42260	3.53620
H	1.28534	-1.25226	2.52082
C	4.64201	-0.74209	1.66283
H	5.24769	0.03605	1.17239
H	4.99259	-0.81509	2.70868
H	4.85883	-1.71086	1.18072
C	2.90114	0.99232	2.29482
H	3.20812	0.95979	3.35593
H	3.48809	1.78288	1.80433
H	1.83947	1.28780	2.25076
C	0.00121	1.77722	-0.02733
H	-0.02948	-0.15793	-1.52447
C	-0.00743	3.02067	-0.04218

C -0.04509 4.50031 -0.01237
C -0.45498 4.97496 1.40953
C -1.08688 5.01210 -1.04544
H -0.82555 4.68675 -2.06598
H -2.09660 4.63604 -0.81094
H -1.11932 6.11555 -1.03264
H -1.45261 4.59370 1.68332
H -0.48657 6.07795 1.44213
H 0.26769 4.62820 2.16669
C 1.34932 5.08534 -0.36277
H 1.66983 4.76948 -1.36950
H 1.31340 6.18860 -0.34547
H 2.11056 4.75728 0.36476

$10_{C\text{-}tBu}$
SCF (BP86) Energy = -1315.45119958
Enthalpy 0K = -1314.710171
Enthalpy 298K = -1314.665198
Free Energy 298K = -1314.783080
Lowest Frequency = 18.7166 cm^{-1}
Second Frequency = 31.6497 cm^{-1}
SCF (BP86-D3BJ) Energy = -1315.678862
SCF (FB) Energy = -1315.492862
SCF (DFB) Energy = -1315.498767
SCF (BS2) Energy = -1985.047832

Rh 0.00502 -0.11476 0.04181
P 2.34241 -0.37102 -0.03130
P -2.32635 -0.42062 0.10508
N 0.02572 -2.27589 -0.13237
C 1.18154 -2.93408 -0.46670
C 1.21614 -4.33334 -0.57963
H 2.15400 -4.82821 -0.84608
C 0.04770 -5.07386 -0.36970
H 0.05601 -6.16405 -0.46318
C -1.13119 -4.39783 -0.03611
H -2.06017 -4.94534 0.14462
C -1.11801 -2.99944 0.09011
C 2.41182 -2.11481 -0.76677
H 3.32470 -2.66013 -0.47243
H 2.47465 -1.96800 -1.86091
C -2.35529 -2.26301 0.53830
H -3.26555 -2.76769 0.17247
H -2.39860 -2.30071 1.64271
C 3.17536 -0.54323 1.70585
C 2.43884 -1.69993 2.42183
H 2.62477 -2.68007 1.95092
H 2.80502 -1.76308 3.46253
H 1.34933 -1.52606 2.45616
C 4.68502 -0.84831 1.64809
H 5.26898 0.00137 1.26019
H 5.04817 -1.04999 2.67250
H 4.91909 -1.73968 1.04114
C 2.91992 0.76076 2.49541
H 1.84257 0.98595 2.55401
H 3.30312 0.63720 3.52467
H 3.42952 1.63112 2.05448
C 3.40650 0.63196 -1.29428
C 2.50704 0.81276 -2.54040
H 1.54994 1.29745 -2.28874
H 3.03686 1.44342 -3.27744
H 2.28108 -0.14543 -3.04076
C 3.72718 2.01533 -0.68859
H 4.46357 1.94982 0.12884
H 4.16625 2.66032 -1.47120
H 2.82229 2.51504 -0.30465
C 4.71227 -0.07932 -1.71277
H 4.52992 -1.05884 -2.18546
H 5.22740 0.54634 -2.46437
H 5.40983 -0.22425 -0.87519
C -3.39664 0.33430 1.52874
C -2.48461 0.34916 2.77865
H -1.56951 0.93850 2.61041
H -3.04051 0.79892 3.62145
H -2.18130 -0.66425 3.09529
C -3.78223 1.78471 1.16627
H -4.51138 1.82817 0.34098
H -4.25777 2.25870 2.04419
H -2.90049 2.38745 0.89428
C -4.66741 -0.48778 1.83998
H -4.44115 -1.51909 2.15798
H -5.19959 -0.00499 2.67981
H -5.36733 -0.53289 0.99301
C -3.18024 -0.31840 -1.62747
C -2.42871 -1.32110 -2.53470
H -2.58685 -2.37040 -2.23308
H -2.80676 -1.21771 -3.56791
H -1.34348 -1.11904 -2.55048
C -4.68229 -0.66270 -1.61026
H -5.27925 0.09170 -1.07366
H -5.05484 -0.69096 -2.65067
H -4.88824 -1.65201 -1.16671
C -2.95808 1.10521 -2.18624
H -3.34454 1.15169 -3.22065
H -3.48238 1.87684 -1.60171
H -1.88475 1.35728 -2.20832
C -0.02789 1.70408 0.34089
H -0.19945 3.10991 1.79857
C -0.08578 2.97366 0.70702
C -0.04845 4.28652 -0.10137
C 0.17023 4.02365 -1.60440
C 1.09677 5.16976 0.45200
H 0.96035 5.37790 1.52725
H 2.07915 4.68616 0.32027
H 1.12020 6.13776 -0.07856
H 1.14207 3.53802 -1.79159
H 0.15936 4.97681 -2.16004
H -0.62487 3.38151 -2.01993
C -1.39789 5.01904 0.10617
H -1.59721 5.20361 1.17586
H -1.38102 5.99699 -0.40653
H -2.23777 4.43454 -0.30561

$TS(B\text{-}10)'_{C\text{-}tBu}$
SCF (BP86) Energy = -1315.33306337
Enthalpy 0K = -1314.594363
Enthalpy 298K = -1314.549666
Free Energy 298K = -1314.664721
Lowest Frequency = -464.8404 cm^{-1}
Second Frequency = 23.6300 cm^{-1}
SCF (BP86-D3BJ) Energy = -1315.56652
SCF (FB) Energy = -1315.37512
SCF (DFB) Energy = -1315.38131
SCF (BS2) Energy = -1984.930783

Rh 0.05269 0.06265 -0.01206
P 2.42484 -0.09675 -0.04898

P -2.26940 -0.46700 0.09051
N 0.23647 -2.07309 -0.05566
C 1.40674 -2.67891 -0.44760
C 1.53144 -4.07499 -0.52461
H 2.48427 -4.50518 -0.84492
C 0.44362 -4.89298 -0.20624
H 0.52341 -5.98234 -0.26611
C -0.74766 -4.28110 0.19477
H -1.62320 -4.87710 0.46603
C -0.82591 -2.88148 0.26850
C 2.56639 -1.80364 -0.82434
H 3.52436 -2.30120 -0.59787
H 2.54642 -1.62466 -1.91535
C -2.07767 -2.22134 0.75786
H -2.96033 -2.85090 0.55852
H -2.00599 -2.10284 1.85488
C 3.23707 -0.29258 1.70862
C 2.56487 -1.51160 2.38174
H 2.83031 -2.46481 1.89508
H 2.91139 -1.57262 3.42956
H 1.46609 -1.41335 2.39142
C 4.76283 -0.51149 1.67527
H 5.31011 0.38287 1.33852
H 5.11059 -0.73798 2.70003
H 5.05718 -1.36305 1.03891
C 2.90178 0.96135 2.54778
H 1.81255 1.05490 2.69269
H 3.36228 0.86023 3.54736
H 3.28811 1.89217 2.10176
C 3.54247 0.94216 -1.26133
C 2.72656 1.13395 -2.56146
H 1.76904 1.64915 -2.37939
H 3.32823 1.73934 -3.26415
H 2.50963 0.17663 -3.06788
C 3.85139 2.32332 -0.64326
H 4.49015 2.25077 0.25225
H 4.40196 2.93029 -1.38488
H 2.93238 2.87158 -0.38390
C 4.86610 0.22413 -1.61512
H 4.70413 -0.75315 -2.09871
H 5.40995 0.85148 -2.34484
H 5.53036 0.07969 -0.75184
C -3.52126 0.22275 1.42066
C -2.65802 0.58974 2.65049
H -1.85669 1.30160 2.39868
H -3.30245 1.04271 3.42612
H -2.18020 -0.29648 3.10338
C -4.25121 1.47393 0.89354
H -4.96237 1.23098 0.08768
H -4.83437 1.92656 1.71610
H -3.55913 2.24282 0.51922
C -4.58205 -0.81761 1.85287
H -4.14255 -1.70164 2.34136
H -5.24750 -0.34301 2.59717
H -5.21540 -1.15786 1.02045
C -3.11047 -0.72295 -1.64281
C -2.17538 -1.65743 -2.44631
H -2.13949 -2.67992 -2.03499
H -2.55728 -1.73360 -3.48069
H -1.14720 -1.25879 -2.49273
C -4.51902 -1.34503 -1.57169
H -5.25633 -0.67029 -1.10825
H -4.87003 -1.54899 -2.60002
H -4.53601 -2.30642 -1.03045
C -3.16203 0.63863 -2.36788
H -3.51707 0.48108 -3.40277
H -3.86068 1.34366 -1.88980
H -2.16350 1.10350 -2.41662
C 0.35643 2.15067 0.48063
H 0.85784 2.52798 1.36645
C 0.15826 2.07370 -0.81272
C -0.75715 3.83118 -0.06963
C 0.38162 4.79012 -0.38482
C -1.34668 4.05873 1.32533
H -2.05773 3.27381 1.61881
H -0.57716 4.15073 2.10840
H -1.90697 5.01409 1.30016
H 1.17753 4.76648 0.37765
H -0.02828 5.81859 -0.40031
H 0.82551 4.58213 -1.36999
C -1.83718 3.82948 -1.14336
H -1.41043 3.78565 -2.15471
H -2.53805 2.99189 -1.02762
H -2.42094 4.76729 -1.03957

TS(B-10)"$_{C-tBu}$

SCF (BP86) Energy = -1315.35950815
Enthalpy 0K = -1314.620639
Enthalpy 298K = -1314.575751
Free Energy 298K = -1314.691455
Lowest Frequency = -435.6637 cm^{-1}
Second Frequency = 21.0132 cm^{-1}
SCF (BP86-D3BJ) Energy = -
1315.594527
SCF (FB) Energy = -1315.400572
SCF (DFB) Energy = -1315.406347
SCF (BS2) Energy = -1984.957103

Rh -0.05205 -0.10420 0.04730
P 2.29373 0.29797 0.14765
P -2.37745 0.22589 -0.03225
N -0.07970 2.03094 -0.12124
C 1.01586 2.77538 0.24864
C 1.05676 4.16927 0.09541
H 1.95629 4.71189 0.39870
C -0.05747 4.84657 -0.41267
H -0.04527 5.93317 -0.53781
C -1.19485 4.09944 -0.73529
H -2.09765 4.58498 -1.11611
C -1.18406 2.70383 -0.58173
C 2.14345 2.02923 0.88988
H 3.08047 2.60631 0.86383
H 1.88509 1.85612 1.94945
C -2.39454 1.88770 -0.92797
H -3.32004 2.46176 -0.75242
H -2.37126 1.63509 -2.00385
C 3.21139 0.64305 -1.55361
C 2.09794 0.89429 -2.59810
H 1.51407 1.80093 -2.37184
H 2.56163 1.03398 -3.59246
H 1.39277 0.04803 -2.65679
C 4.12732 1.88505 -1.48974
H 4.91151 1.80389 -0.72057
H 4.63644 1.99928 -2.46437
H 3.55884 2.81341 -1.31691
C 4.04752 -0.57308 -2.00642
H 3.45032 -1.49615 -2.07074
H 4.44130 -0.37187 -3.01959

H	4.91598	-0.75977	-1.35507
C	3.48608	-0.52534	1.44578
C	2.77393	-0.43962	2.81869
H	1.72256	-0.76928	2.75597
H	3.29781	-1.10025	3.53324
H	2.79949	0.57532	3.24840
C	3.66995	-2.01811	1.10215
H	4.16017	-2.18118	0.13067
H	4.30523	-2.49042	1.87392
H	2.69893	-2.53602	1.10424
C	4.86059	0.17011	1.53489
H	4.77772	1.24589	1.76312
H	5.44147	-0.28997	2.35555
H	5.45462	0.05438	0.61437
C	-3.56116	-0.78465	-1.19086
C	-2.71633	-1.11658	-2.44531
H	-1.72565	-1.52376	-2.18564
H	-3.25456	-1.85395	-3.06856
H	-2.54789	-0.22592	-3.07516
C	-3.99808	-2.07688	-0.46744
H	-4.74360	-1.86888	0.31727
H	-4.47281	-2.76386	-1.19173
H	-3.15184	-2.60576	-0.00319
C	-4.81730	-0.00932	-1.65059
H	-4.57037	0.91360	-2.20029
H	-5.38711	-0.65176	-2.34719
H	-5.49176	0.25011	-0.82233
C	-3.20093	0.58568	1.69049
C	-2.40352	1.74791	2.32735
H	-2.53578	2.69976	1.78618
H	-2.76752	1.90380	3.35931
H	-1.32553	1.51784	2.37528
C	-4.68981	0.97689	1.60790
H	-5.32921	0.13720	1.29266
H	-5.03359	1.28517	2.61244
H	-4.87057	1.82837	0.92991
C	-3.02808	-0.66280	2.58415
H	-3.41194	-0.43753	3.59606
H	-3.58085	-1.53699	2.20679
H	-1.96379	-0.93637	2.67555
C	-0.05138	-2.04991	0.53368
C	-0.17811	-2.75591	1.57972
H	-0.27565	-3.40418	2.43617
C	0.26438	-3.66405	-0.42853
C	-1.08783	-4.15989	-0.92288
H	-0.90597	-5.01276	-1.60508
H	-1.72255	-4.52219	-0.09929
H	-1.63491	-3.39532	-1.48816
C	1.00287	-4.78318	0.33193
H	0.36698	-5.28716	1.07736
H	1.29901	-5.54845	-0.40972
H	1.92176	-4.43100	0.82374
C	1.14449	-3.13190	-1.55880
H	2.10667	-2.76348	-1.17835
H	1.35284	-3.97656	-2.24574
H	0.64936	-2.32403	-2.11658

TS(B-F)$_{C-tBu}$

SCF (BP86) Energy = -1315.34138384
Enthalpy 0K = -1314.602636
Enthalpy 298K = -1314.557787
Free Energy 298K = -1314.672611
Lowest Frequency = -228.9709 cm^{-1}
Second Frequency = 33.9755 cm^{-1}
SCF (BP86-D3BJ) Energy = -1315.584824
SCF (FB) Energy = -1315.38253
SCF (DFB) Energy = -1315.388362
SCF (BS2) Energy = -1984.939804

Rh	-0.04265	-0.32745	-0.05144
P	2.32360	0.13856	0.25066
P	-2.37106	0.12657	0.02308
N	-0.05720	1.75409	-0.63434
C	0.99676	2.57660	-0.33288
C	1.00269	3.93210	-0.70194
H	1.87080	4.54919	-0.45444
C	-0.10832	4.48103	-1.35092
H	-0.11896	5.53393	-1.64762
C	-1.22069	3.66170	-1.57964
H	-2.12546	4.05780	-2.04854
C	-1.17679	2.31078	-1.20440
C	2.10345	2.00082	0.50092
H	3.04221	2.55798	0.35247
H	1.82570	2.11981	1.56464
C	-2.36462	1.40971	-1.37184
H	-3.29835	1.99061	-1.44728
H	-2.26083	0.81848	-2.29879
C	3.64701	0.16554	-1.21270
C	2.98162	0.96167	-2.36170
H	2.90144	2.03745	-2.13659
H	3.60491	0.86526	-3.26926
H	1.97236	0.58943	-2.60594
C	4.97686	0.85407	-0.83451
H	5.60586	0.21564	-0.19559
H	5.54668	1.04157	-1.76334
H	4.85009	1.82781	-0.33433
C	3.97804	-1.25273	-1.72254
H	3.10510	-1.76080	-2.15602
H	4.73499	-1.16949	-2.52395
H	4.40623	-1.89911	-0.93997
C	3.12284	-0.36173	1.95954
C	2.00852	-0.23026	3.02504
H	1.12654	-0.84029	2.77873
H	2.41404	-0.57973	3.99254
H	1.68827	0.81544	3.17695
C	3.57375	-1.83662	1.89765
H	4.39004	-1.99904	1.17464
H	3.95836	-2.13494	2.89006
H	2.73335	-2.50600	1.65421
C	4.30740	0.53534	2.38259
H	4.05237	1.60827	2.39198
H	4.59015	0.26591	3.41661
H	5.19963	0.39637	1.75717
C	-3.87493	-1.00494	-0.48332
C	-3.67677	-1.38090	-1.97275
H	-2.65421	-1.71874	-2.20170
H	-4.36375	-2.20757	-2.22765
H	-3.91999	-0.54310	-2.64706
C	-3.86285	-2.27807	0.39797
H	-4.24416	-2.07709	1.40971
H	-4.52530	-3.03817	-0.05477
H	-2.85702	-2.70981	0.50794
C	-5.24832	-0.30846	-0.36283
H	-5.30479	0.62917	-0.94130
H	-6.01818	-0.98777	-0.77346
H	-5.53052	-0.09816	0.67980
C	-2.81574	1.11770	1.65156

C	-1.52260	1.75883	2.20978
H	-1.15271	2.57898	1.57485
H	-1.74909	2.18816	3.20325
H	-0.71825	1.01306	2.32869
C	-3.84558	2.24118	1.40535
H	-4.81052	1.87826	1.02429
H	-4.04450	2.75140	2.36578
H	-3.46064	3.00705	0.71087
C	-3.33254	0.11663	2.70951
H	-3.41729	0.64012	3.67902
H	-4.33078	-0.28009	2.47002
H	-2.63778	-0.72947	2.84713
C	-0.15481	-2.04112	0.87869
C	-0.30603	-2.88605	1.78533
H	-0.41590	-3.62190	2.56210
C	0.18616	-2.88561	-1.14029
C	0.26976	-2.09562	-2.44341
H	1.09727	-1.37545	-2.46736
H	0.44963	-2.83780	-3.25496
H	-0.66402	-1.57080	-2.69114
C	-0.98609	-3.84711	-1.14831
H	-0.83869	-4.50941	-2.02763
H	-1.02259	-4.46845	-0.24410
H	-1.95570	-3.35419	-1.28493
C	1.48440	-3.57719	-0.76772
H	1.34496	-4.27442	0.06929
H	1.81845	-4.15249	-1.65667
H	2.28659	-2.87661	-0.51256

F_{C-tBu}

SCF (BP86) Energy = -1315.36066149
Enthalpy 0K = -1314.620224
Enthalpy 298K = -1314.575252
Free Energy 298K = -1314.690048
Lowest Frequency = 18.8888 cm^{-1}
Second Frequency = 38.0231 cm^{-1}
SCF (BP86-D3BJ) Energy = -1315.655218
SCF (FB) Energy = -1315.471692
SCF (DFB) Energy = -1315.47812
SCF (BS2) Energy = -1985.027935

Rh	-0.07153	-0.50051	-0.06287
P	2.38526	-0.05216	0.25489
P	-2.41384	0.04537	0.02593
N	0.00089	1.69202	-0.49304
C	1.05315	2.44506	-0.04521
C	1.07778	3.84128	-0.20660
H	1.94499	4.40678	0.14551
C	-0.01899	4.49186	-0.78139
H	-0.01373	5.57681	-0.92221
C	-1.14549	3.73263	-1.12261
H	-2.04782	4.20924	-1.51505
C	-1.11723	2.33992	-0.95047
C	2.12173	1.74984	0.75520
H	3.06271	2.32284	0.74085
H	1.78629	1.71906	1.80885
C	-2.33699	1.49908	-1.19385
H	-3.24958	2.11766	-1.17542
H	-2.28489	1.01986	-2.18807
C	3.76772	0.25367	-1.15386
C	3.23615	1.36898	-2.08680
H	3.26298	2.36266	-1.61233
H	3.89217	1.41966	-2.97461
H	2.21086	1.19068	-2.44612
C	5.10555	0.74092	-0.55419
H	5.67124	-0.07673	-0.08351
H	5.72651	1.12691	-1.38364
H	4.99756	1.56006	0.17461
C	4.06900	-1.00215	-1.99981
H	3.22893	-1.29511	-2.64479
H	4.92202	-0.77026	-2.66361
H	4.36242	-1.87138	-1.38936
C	3.16500	-0.81782	1.87439
C	2.05739	-0.81543	2.95373
H	1.15224	-1.35072	2.63030
H	2.45321	-1.33323	3.84682
H	1.78845	0.20546	3.27843
C	3.55608	-2.27682	1.55220
H	4.35806	-2.33809	0.79736
H	3.93708	-2.75856	2.47088
H	2.68821	-2.86059	1.20486
C	4.37448	-0.04680	2.45047
H	4.17368	1.02989	2.58427
H	4.58739	-0.45704	3.45429
H	5.29027	-0.16066	1.85713
C	-4.10228	-0.80917	-0.53563
C	-4.23169	-0.75316	-2.07990
H	-3.34849	-1.10701	-2.63109
H	-5.07375	-1.40437	-2.37456
H	-4.47254	0.26245	-2.43445
C	-4.12356	-2.27045	-0.03481
H	-4.07914	-2.33412	1.06250
H	-5.07039	-2.74015	-0.35796
H	-3.29401	-2.86624	-0.43650
C	-5.34103	-0.06330	0.01437
H	-5.36785	0.99768	-0.28448
H	-6.23741	-0.54151	-0.42146
H	-5.44366	-0.12984	1.10659
C	-2.64395	0.78668	1.82461
C	-1.25404	1.22236	2.34978
H	-0.88480	2.13603	1.86060
H	-1.34289	1.43857	3.43053
H	-0.50251	0.41596	2.24284
C	-3.57485	2.01608	1.88390
H	-4.61956	1.79259	1.63244
H	-3.56584	2.40831	2.91768
H	-3.22258	2.83189	1.22955
C	-3.13739	-0.35471	2.74519
H	-3.13140	0.01050	3.78831
H	-4.16508	-0.67769	2.52372
H	-2.46936	-1.23168	2.69100
C	-0.30091	-2.14549	0.94838
C	-0.51226	-3.14043	1.65306
H	-0.65190	-3.99705	2.28751
C	0.09368	-1.90176	-1.75201
C	0.29052	-0.96047	-2.93749
H	1.20694	-0.35980	-2.86362
H	0.37023	-1.56450	-3.86641
H	-0.56558	-0.27843	-3.07644
C	-1.15825	-2.75163	-1.94566
H	-0.99899	-3.39859	-2.83288
H	-1.34050	-3.40306	-1.07985
H	-2.05269	-2.15272	-2.14620
C	1.26388	-2.86711	-1.52901
H	1.00879	-3.61325	-0.76328
H	1.46265	-3.40159	-2.48112
H	2.19310	-2.37668	-1.22629

9$_O$

SCF (BP86) Energy = -2617.37083069
Enthalpy 0K = -2616.080830
Enthalpy 298K = -2615.999755
Free Energy 298K = -2616.193459
Lowest Frequency = 10.7412 cm^{-1}
Second Frequency = 15.1242 cm^{-1}
SCF (BP86-D3BJ) Energy = -2617.80779
SCF (FB) Energy = -2617.495255
SCF (DFB) Energy = -2617.512123
SCF (BS2) Energy = -3956.582084

Rh	2.75469	-2.03675	1.15829
P	4.02833	-2.84698	-0.62848
P	2.03521	-1.69508	3.40281
N	4.07000	-3.33930	2.18531
C	1.20827	-1.38514	-0.27483
H	1.57293	-1.74683	-1.24618
C	1.87025	-0.24072	0.23584
H	2.71598	0.14022	-0.35437
C	1.17746	0.86858	1.01191
H	1.93826	1.51883	1.47564
H	0.56438	0.46110	1.83381
C	-0.25426	-1.73510	-0.07389
H	-0.56186	-1.59581	0.97481
H	-0.40269	-2.80580	-0.29933
C	5.13093	-3.92665	1.54600
C	6.02688	-4.79113	2.18866
H	6.85114	-5.22387	1.61994
C	5.81471	-5.07288	3.54332
H	6.49423	-5.74755	4.07198
C	4.73739	-4.49480	4.22333
H	4.53808	-4.68450	5.27898
C	3.89232	-3.63044	3.51195
O	5.31810	-3.66238	0.23748
O	2.85950	-3.06338	4.15618
C	3.34877	-4.37571	-1.61640
C	2.36767	-5.07739	-0.64650
H	2.88203	-5.47062	0.24627
H	1.90734	-5.93742	-1.16591
H	1.56425	-4.40361	-0.30410
C	4.46692	-5.36724	-2.00650
H	5.17173	-4.95178	-2.74071
H	3.99238	-6.25176	-2.46842
H	5.03939	-5.71291	-1.13322
C	2.59659	-3.91605	-2.88466
H	1.79902	-3.18288	-2.67564
H	2.11096	-4.79793	-3.34002
H	3.27466	-3.49344	-3.64296
C	5.16053	-1.68441	-1.66126
C	5.84130	-0.76199	-0.62112
H	5.10695	-0.23049	0.00831
H	6.44451	-0.00782	-1.15752
H	6.51972	-1.32478	0.03944
C	4.29654	-0.83946	-2.62447
H	3.82255	-1.44629	-3.41102
H	4.95247	-0.10628	-3.12694
H	3.51039	-0.26713	-2.10445
C	6.23554	-2.45407	-2.45689
H	6.81069	-3.14499	-1.82152
H	6.94570	-1.72145	-2.88109
H	5.80800	-3.01654	-3.30188
C	0.31764	-2.18071	4.15249
C	-0.05136	-3.51914	3.46825
H	-0.06310	-3.43661	2.36855
H	-1.06100	-3.82217	3.79938
H	0.64733	-4.32328	3.74708
C	-0.75115	-1.11545	3.82766
H	-0.54895	-0.14902	4.31381
H	-1.72485	-1.47659	4.20481
H	-0.86618	-0.94370	2.74514
C	0.38736	-2.38428	5.68163
H	1.17944	-3.08999	5.97276
H	-0.57665	-2.80648	6.01917
H	0.53356	-1.43760	6.22578
C	2.91679	-0.27113	4.41077
C	4.09703	0.20144	3.52861
H	4.83012	-0.60404	3.35561
H	4.62431	1.02339	4.04648
H	3.76468	0.56988	2.54439
C	3.47620	-0.77693	5.75924
H	2.69626	-1.15146	6.43743
H	3.97208	0.07396	6.26054
H	4.22886	-1.56868	5.62899
C	1.94880	0.90465	4.66693
H	2.52567	1.74348	5.09647
H	1.16498	0.64570	5.39658
H	1.46889	1.27836	3.74857
C	-1.20827	1.38514	0.27483
H	-1.57293	1.74683	1.24618
C	-1.87025	0.24072	-0.23584
H	-2.71598	-0.14022	0.35437
C	-1.17746	-0.86858	-1.01191
H	-1.93826	-1.51883	-1.47564
H	-0.56438	-0.46110	-1.83381
C	0.25426	1.73510	0.07389
H	0.56186	1.59581	-0.97481
H	0.40269	2.80580	0.29933
Rh	-2.75469	2.03675	-1.15829
P	-4.02833	2.84698	0.62848
P	-2.03521	1.69508	-3.40281
N	-4.07000	3.33930	-2.18531
C	-5.13093	3.92665	-1.54600
C	-6.02688	4.79113	-2.18866
H	-6.85114	5.22387	-1.61994
C	-5.81471	5.07288	-3.54332
H	-6.49423	5.74755	-4.07198
C	-4.73739	4.49480	-4.22333
H	-4.53808	4.68450	-5.27898
C	-3.89232	3.63044	-3.51195
O	-5.31810	3.66238	-0.23748
O	-2.85950	3.06338	-4.15618
C	-3.34877	4.37571	1.61640
C	-2.36767	5.07739	0.64650
H	-2.88203	5.47062	-0.24627
H	-1.90734	5.93742	1.16591
H	-1.56425	4.40361	0.30410
C	-4.46692	5.36724	2.00650
H	-5.17173	4.95178	2.74071
H	-3.99238	6.25176	2.46842
H	-5.03939	5.71291	1.13322
C	-2.59659	3.91605	2.88466
H	-1.79902	3.18288	2.67564
H	-2.11096	4.79793	3.34002
H	-3.27466	3.49344	3.64296
C	-5.16053	1.68441	1.66126
C	-5.84130	0.76199	0.62112

H -5.10695 0.23049 -0.00831
H -6.44451 0.00782 1.15752
H -6.51972 1.32478 -0.03944
C -4.29654 0.83946 2.62447
H -3.82255 1.44629 3.41102
H -4.95247 0.10628 3.12694
H -3.51039 0.26713 2.10445
C -6.23554 2.45407 2.45689
H -6.81069 3.14499 1.82152
H -6.94570 1.72145 2.88109
H -5.80800 3.01654 3.30188
C -0.31764 2.18071 -4.15249
C 0.05136 3.51914 -3.46825
H 0.06310 3.43661 -2.36855
H 1.06100 3.82217 -3.79938
H -0.64733 4.32328 -3.74708
C 0.75115 1.11545 -3.82766
H 0.54895 0.14902 -4.31381
H 1.72485 1.47659 -4.20481
H 0.86618 0.94370 -2.74514
C -0.38736 2.38428 -5.68163
H -1.17944 3.08999 -5.97276
H 0.57665 2.80648 -6.01917
H -0.53356 1.43760 -6.22578
C -2.91679 0.27113 -4.41077
C -4.09703 -0.20144 -3.52861
H -4.83012 0.60404 -3.35561
H -4.62431 -1.02339 -4.04648
H -3.76468 -0.56988 -2.54439
C -3.47620 0.77693 -5.75924
H -2.69626 1.15146 -6.43743
H -3.97208 -0.07396 -6.26054
H -4.22886 1.56868 -5.62899
C -1.94880 -0.90465 -4.66693
H -2.52567 -1.74348 -5.09647
H -1.16498 -0.64570 -5.39658
H -1.46889 -1.27836 -3.74857

8_O

SCF (BP86) Energy = -1464.72451816
Enthalpy 0K = -1463.992143
Enthalpy 298K = -1463.947274
Free Energy 298K = -1464.064842
Lowest Frequency = 18.1088 cm^{-1}
Second Frequency = 26.4435 cm^{-1}
SCF (BP86-D3BJ) Energy = -1464.956261
SCF (FB) Energy = -1464.764617
SCF (DFB) Energy = -1464.770104
SCF (BS2) Energy = -2134.367708

C 2.88639 4.41089 0.38931
H 3.91954 4.70427 0.61640
C 2.45792 4.58215 -0.87857
H 3.17113 5.00425 -1.59832
C 1.08808 4.24106 -1.43343
H 0.98931 4.68412 -2.43839
H 0.29698 4.70964 -0.81449
C 2.09755 3.82691 1.54604
H 1.11198 4.32595 1.62989
H 2.62828 4.04105 2.48867
C -0.01118 2.21116 -0.35481
H -1.05488 2.54921 -0.41945
C 0.46227 1.96922 0.95543
H -0.27317 2.07677 1.76619
C 1.87299 2.27690 1.42565
H 2.03641 1.80296 2.40879
H 2.62833 1.86678 0.73591
C 0.80480 2.69908 -1.53324
H 1.76003 2.15842 -1.61450
H 0.24389 2.48882 -2.46191
Rh -0.14766 0.04047 0.06270
P -2.46198 0.24957 0.09594
P 2.00287 -0.96863 -0.02790
N -0.70209 -1.98116 -0.18091
C -2.00834 -2.37612 -0.05518
C -2.41885 -3.70850 -0.20104
H -3.47518 -3.95595 -0.08742
C -1.44580 -4.66935 -0.50035
H -1.73568 -5.71683 -0.62438
C -0.10534 -4.29629 -0.64163
H 0.68436 -5.01293 -0.87142
C 0.22834 -2.94352 -0.46926
O -2.94041 -1.44128 0.21650
O 1.51212 -2.57596 -0.59463
C -3.37750 0.66691 -1.56585
C -2.41000 0.20126 -2.68079
H -2.25570 -0.89075 -2.66155
H -2.84895 0.45574 -3.66274
H -1.42133 0.68352 -2.60317
C -4.71427 -0.09316 -1.71155
H -5.47669 0.23950 -0.99256
H -5.10921 0.10279 -2.72493
H -4.58764 -1.18077 -1.60512
C -3.62850 2.18593 -1.68944
H -2.71209 2.78915 -1.57985
H -4.02860 2.39142 -2.69880
H -4.37663 2.54719 -0.96607
C -3.38551 0.86117 1.66811
C -2.68940 0.13810 2.84654
H -1.60119 0.31998 2.86055
H -3.11370 0.51570 3.79417
H -2.85758 -0.95018 2.81223
C -3.20118 2.38941 1.81054
H -3.71482 2.95516 1.01825
H -3.63904 2.70538 2.77428
H -2.14180 2.69359 1.82258
C -4.88739 0.50926 1.65710
H -5.06482 -0.55822 1.45331
H -5.30586 0.73393 2.65498
H -5.45137 1.11144 0.92738
C 3.31855 -0.74703 -1.43117
C 2.49270 -0.66174 -2.73763
H 1.74480 0.14770 -2.70541
H 3.17941 -0.45810 -3.57894
H 1.97059 -1.60799 -2.95062
C 4.13236 0.55151 -1.23833
H 4.75935 0.52993 -0.33422
H 4.81112 0.66340 -2.10304
H 3.50797 1.45815 -1.20260
C 4.28568 -1.94824 -1.51120
H 3.75471 -2.90877 -1.59066
H 4.90611 -1.83153 -2.41844
H 4.97487 -1.98907 -0.65279
C 2.80612 -1.53803 1.66208
C 1.70177 -1.36227 2.73151
H 0.81978 -1.98983 2.51939
H 2.10278 -1.67153 3.71427

H	1.35908	-0.31838	2.81214
C	3.23078	-3.02336	1.62987
H	3.99624	-3.23627	0.87003
H	3.66220	-3.27306	2.61635
H	2.37872	-3.69823	1.45884
C	4.02903	-0.66520	2.01945
H	4.34738	-0.91644	3.04748
H	4.88777	-0.86300	1.35849
H	3.81260	0.41384	2.00162

$\mathbf{A_O}$

SCF (BP86) Energy = -1152.65482641
Enthalpy 0K = -1152.100117
Enthalpy 298K = -1152.063943
Free Energy 298K = -1152.164526
Lowest Frequency = 20.3372 cm^{-1}
Second Frequency = 31.2022 cm^{-1}
SCF (BP86-D3BJ) Energy = -1152.821592
SCF (FB) Energy = -1152.703619
SCF (DFB) Energy = -1152.711718
SCF (BS2) Energy = -1822.227633

Rh	0.00000	-0.57528	0.10578
P	2.28202	-0.30044	0.05120
P	-2.28202	-0.30044	0.05120
N	0.00000	1.40174	-0.13732
C	1.18250	2.10813	-0.22983
C	1.21181	3.49636	-0.41054
H	2.18071	3.99385	-0.47595
C	0.00000	4.18928	-0.50024
H	0.00000	5.27348	-0.64204
C	-1.21181	3.49636	-0.41054
H	-2.18070	3.99386	-0.47595
C	-1.18250	2.10813	-0.22983
O	2.35554	1.44590	-0.13995
O	-2.35554	1.44590	-0.13995
C	3.17744	-0.89649	-1.53424
C	2.48271	-0.16531	-2.70695
H	2.70280	0.91407	-2.70594
H	2.85585	-0.58686	-3.65734
H	1.38712	-0.30228	-2.68050
C	4.69260	-0.62389	-1.56772
H	5.24288	-1.21371	-0.81742
H	5.08232	-0.91628	-2.55963
H	4.92613	0.44315	-1.42265
C	2.88591	-2.41426	-1.63367
H	1.80002	-2.62038	-1.64221
H	3.30039	-2.79611	-2.58386
H	3.34694	-2.99452	-0.81803
C	3.30022	-0.47383	1.66478
C	2.35315	0.00896	2.79008
H	1.41181	-0.56697	2.81393
H	2.86167	-0.11792	3.76275
H	2.09951	1.07681	2.68311
C	3.62729	-1.97084	1.86476
H	4.35449	-2.34536	1.12608
H	4.07754	-2.10634	2.86421
H	2.72567	-2.60832	1.82651
C	4.58258	0.38407	1.67322
H	4.36707	1.44339	1.46291
H	5.03153	0.32788	2.68139
H	5.33823	0.03102	0.95727
C	-3.17744	-0.89649	-1.53424
C	-2.48270	-0.16533	-2.70695
H	-1.38711	-0.30230	-2.68049
H	-2.85584	-0.58687	-3.65734
H	-2.70278	0.91407	-2.70595
C	-2.88593	-2.41426	-1.63366
H	-3.34696	-2.99452	-0.81801
H	-3.30041	-2.79611	-2.58384
H	-1.80004	-2.62039	-1.64220
C	-4.69260	-0.62387	-1.56772
H	-4.92612	0.44317	-1.42267
H	-5.08232	-0.91627	-2.55963
H	-5.24288	-1.21368	-0.81741
C	-3.30022	-0.47383	1.66478
C	-2.35315	0.00897	2.79008
H	-2.09952	1.07682	2.68311
H	-2.86167	-0.11792	3.76275
H	-1.41181	-0.56697	2.81393
C	-4.58259	0.38407	1.67322
H	-5.33823	0.03101	0.95727
H	-5.03153	0.32788	2.68139
H	-4.36707	1.44339	1.46291
C	-3.62729	-1.97084	1.86476
H	-4.07754	-2.10634	2.86421
H	-4.35448	-2.34536	1.12607
H	-2.72566	-2.60832	1.82652

$\mathbf{B_{O\text{-}tBu}}$

SCF (BP86) Energy = -1387.29807486
Enthalpy 0K = -1386.604571
Enthalpy 298K = -1386.559577
Free Energy 298K = -1386.676898
Lowest Frequency = 23.1101 cm^{-1}
Second Frequency = 27.6162 cm^{-1}
SCF (BP86-D3BJ) Energy = -1387.516554
SCF (FB) Energy = -1387.33849
SCF (DFB) Energy = -1387.34399
SCF (BS2) Energy = -2056.924554

Rh	-0.00009	0.11249	0.16550
P	2.31227	-0.24084	0.06419
P	-2.31223	-0.24083	0.06424
N	-0.00004	-1.96299	-0.13477
C	1.17566	-2.66846	-0.19393
C	1.21442	-4.06231	-0.34477
H	2.18109	-4.56593	-0.38812
C	-0.00006	-4.75291	-0.42207
H	-0.00007	-5.84075	-0.53701
C	-1.21454	-4.06233	-0.34438
H	-2.18121	-4.56598	-0.38741
C	-1.17576	-2.66850	-0.19342
O	2.34072	-2.00217	-0.09444
O	-2.34079	-2.00227	-0.09308
C	3.42012	-0.10083	1.62579
C	2.65270	-0.86331	2.73277
H	2.61629	-1.94612	2.53249
H	3.17837	-0.71541	3.69310
H	1.61890	-0.49671	2.84892
C	4.82175	-0.71794	1.45040
H	5.45783	-0.12295	0.77615
H	5.32212	-0.73552	2.43570
H	4.77912	-1.75440	1.07985
C	3.52882	1.39548	2.00029
H	2.54108	1.87201	2.10772

```
H   4.05204  1.47779  2.97003
H   4.11288  1.97146  1.26514
C   3.21837  0.15409 -1.59349
C   2.12374  0.01428 -2.67907
H   1.26205  0.67533 -2.48339
H   2.55331  0.28765 -3.66000
H   1.75445 -1.02227 -2.75806
C   3.73747  1.60620 -1.55175
H   4.58689  1.72517 -0.85993
H   4.09444  1.88715 -2.55892
H   2.95127  2.32616 -1.26943
C   4.37087 -0.82068 -1.91438
H   4.04066 -1.87029 -1.89841
H   4.73541 -0.59694 -2.93352
H   5.22452 -0.71285 -1.23040
C  -3.42081 -0.09994  1.62524
C  -2.65398 -0.86188  2.73301
H  -1.62026 -0.49523  2.84962
H  -3.18021 -0.71356  3.69297
H  -2.61742 -1.94477  2.53321
C  -3.52984  1.39655  1.99894
H  -4.11360  1.97209  1.26319
H  -4.05355  1.47930  2.96836
H  -2.54221  1.87325  2.10660
C  -4.82235 -0.71718  1.44949
H  -4.77954 -1.75373  1.07923
H  -5.32306 -0.73450  2.43462
H  -5.45821 -0.12240  0.77485
C  -3.21746  0.15336 -1.59410
C  -2.12240  0.01254 -2.67913
H  -1.75348 -1.02419 -2.75748
H  -2.55143  0.28562 -3.66037
H  -1.26053  0.67334 -2.48336
C  -4.37016 -0.82121 -1.91484
H  -5.22415 -0.71243 -1.23144
H  -4.73404 -0.59820 -2.93438
H  -4.04041 -1.87095 -1.89779
C  -3.73600  1.60570 -1.55356
H  -4.09264  1.88601 -2.56102
H  -4.58551  1.72557 -0.86201
H  -2.94954  2.32554 -1.27164
C  -0.00043  1.64620  1.59599
H  -0.00094  1.51156  2.67265
C  -0.00010  2.32205  0.51974
C   0.00008  3.64548 -0.19993
C   0.00067  3.49674 -1.73695
C   1.26031  4.43723  0.24791
H   1.26279  4.59359  1.33888
H   2.19013  3.91306 -0.02646
H   1.27009  5.42589 -0.24270
H   0.89281  2.95519 -2.09094
H   0.00052  4.49394 -2.20847
H  -0.89085  2.95458 -2.09156
C  -1.26065  4.43698  0.24694
H  -1.26407  4.59320  1.33793
H  -1.27013  5.42570 -0.24355
H  -2.19017  3.91272 -0.02830
```

TS(B-10)$_{O-tBu}$

SCF (BP86) Energy = -1387.22856413
Enthalpy 0K = -1386.538460
Enthalpy 298K = -1386.493833
Free Energy 298K = -1386.611358
Lowest Frequency = -590.3387 cm^{-1}
Second Frequency = 12.6015 cm^{-1}
SCF (BP86-D3BJ) Energy = -1387.44418
SCF (FB) Energy = -1387.26948
SCF (DFB) Energy = -1387.275165
SCF (BS2) Energy = -2056.854194

```
Rh  0.02479  0.11215  0.11548
P   2.32986 -0.24047  0.06198
P  -2.29041 -0.28628  0.08266
N   0.05166 -1.88661 -0.49136
C   1.23168 -2.55248 -0.71215
C   1.28804 -3.90042 -1.09027
H   2.26133 -4.36435 -1.25577
C   0.08401 -4.60018 -1.22908
H   0.09649 -5.65455 -1.51971
C  -1.13395 -3.95801 -0.98618
H  -2.09510 -4.46702 -1.07010
C  -1.11078 -2.60554 -0.61333
O   2.39186 -1.88189 -0.56448
O  -2.28129 -1.99282 -0.37085
C   3.21411 -0.53576  1.74833
C   2.32305 -1.55540  2.49723
H   2.32367 -2.54307  2.00823
H   2.72313 -1.68802  3.51850
H   1.28233 -1.19935  2.58372
C   4.64053 -1.09971  1.59596
H   5.34966 -0.34519  1.22034
H   5.00011 -1.41336  2.59271
H   4.67450 -1.98173  0.93701
C   3.23630  0.79014  2.54160
H   2.21829  1.16451  2.74114
H   3.71238  0.60200  3.52082
H   3.82399  1.57902  2.04397
C   3.44506  0.55717 -1.28725
C   2.52883  0.67283 -2.52941
H   1.59596  1.21734 -2.31264
H   3.07278  1.21038 -3.32723
H   2.25991 -0.32187 -2.92213
C   3.88489  1.95462 -0.79958
H   4.62932  1.89570  0.01105
H   4.35640  2.49804 -1.63806
H   3.03738  2.56905 -0.44843
C   4.67845 -0.29361 -1.65948
H   4.40052 -1.32392 -1.92875
H   5.15691  0.16637 -2.54319
H   5.43228 -0.32987 -0.86068
C  -3.15083 -0.47661  1.79227
C  -2.18591 -1.33529  2.64459
H  -1.18926 -0.86909  2.71998
H  -2.60089 -1.42010  3.66515
H  -2.07627 -2.35594  2.24316
C  -3.27743  0.92328  2.43361
H  -3.98139  1.57783  1.89669
H  -3.66095  0.80245  3.46278
H  -2.29759  1.42753  2.50803
C  -4.52286 -1.17445  1.70114
H  -4.45634 -2.15147  1.19619
H  -4.89350 -1.35386  2.72660
H  -5.27910 -0.55944  1.18805
C  -3.50349  0.30145 -1.31134
C  -2.59531  0.71293 -2.49336
H  -1.98639 -0.13285 -2.85514
H  -3.22678  1.05499 -3.33331
H  -1.90651  1.53008 -2.22374
```

C -4.45203 -0.82657 -1.77540
H -5.10450 -1.19459 -0.97016
H -5.10227 -0.41642 -2.56948
H -3.90463 -1.68268 -2.19570
C -4.33790 1.50678 -0.82675
H -4.86072 1.94656 -1.69516
H -5.10941 1.20945 -0.09917
H -3.72859 2.30378 -0.37368
C -0.05061 1.66566 1.53192
H   1.04852 2.34620 1.20049
C   0.04980 2.41680 0.44161
C -0.11334 3.76695 -0.24321
C   0.43499 3.77180 -1.68627
C   0.64765 4.81393 0.61788
H   0.25564 4.84893 1.64739
H   1.72916 4.59584 0.65982
H   0.52607 5.81286 0.16711
H   1.52091 3.59102 -1.71375
H   0.24727 4.75749 -2.14414
H -0.05961 3.00622 -2.30599
C -1.61877 4.12854 -0.26314
H -2.05911 4.09085 0.74622
H -1.73405 5.15335 -0.65435
H -2.18067 3.45605 -0.92843

TS(B–C)$_{O\text{-}tBu}$
SCF (BP86) Energy = -1387.28468888
Enthalpy 0K = -1386.593586
Enthalpy 298K = -1386.548514
Free Energy 298K = -1386.668911
Lowest Frequency = -136.0195 cm^{-1}
Second Frequency = 12.9844 cm^{-1}
SCF (BP86-D3BJ) Energy = -1387.495595
SCF (FB) Energy = -1387.324574
SCF (DFB) Energy = -1387.329988
SCF (BS2) Energy = -2056.911156

Rh 0.00002 -0.04585 0.20532
P   2.28076 -0.38253 0.09431
P -2.28069 -0.38276 0.09430
N   0.00012 -2.04369 -0.38977
C   1.17306 -2.72738 -0.57959
C   1.21328 -4.07716 -0.95569
H   2.18133 -4.56205 -1.08988
C   0.00026 -4.74976 -1.14274
H   0.00031 -5.80363 -1.43499
C -1.21284 -4.07727 -0.95576
H -2.18084 -4.56224 -1.08999
C -1.17276 -2.72748 -0.57965
O   2.34170 -2.07214 -0.40084
O -2.34145 -2.07234 -0.40096
C   3.28246 -0.52890 1.72828
C   2.46416 -1.50056 2.61290
H   2.45228 -2.52162 2.19905
H   2.93006 -1.54742 3.61360
H   1.42017 -1.16281 2.73649
C   4.70836 -1.08073 1.53251
H   5.37233 -0.35395 1.03834
H   5.14390 -1.29485 2.52543
H   4.71538 -2.01940 0.95607
C   3.32679 0.86088 2.40210
H   2.31938 1.28236 2.55377
H   3.79608 0.75627 3.39675
H   3.92352 1.59087 1.83294
C   3.26707 0.36088 -1.37904
C   2.25143 0.39800 -2.54684
H   1.34807 0.97680 -2.28864
H   2.73154 0.86867 -3.42420
H   1.93354 -0.61512 -2.84477
C   3.69140 1.79830 -1.00915
H   4.49977 1.81460 -0.26026
H   4.07439 2.30390 -1.91398
H   2.84555 2.39472 -0.62470
C   4.48853 -0.48230 -1.79975
H   4.21346 -1.52913 -2.00072
H   4.89985 -0.05674 -2.73317
H   5.29380 -0.47041 -1.05137
C -3.28235 -0.52935 1.72828
C -2.46395 -1.50101 2.61280
H -1.41998 -1.16319 2.73637
H -2.92981 -1.54796 3.61352
H -2.45201 -2.52204 2.19889
C -3.32678 0.86037 2.40221
H -3.92356 1.59037 1.83311
H -3.79608 0.75565 3.39684
H -2.31940 1.28191 2.55393
C -4.70821 -1.08129 1.53249
H -4.71516 -2.01991 0.95596
H -5.14371 -1.29553 2.52540
H -5.37225 -0.35452 1.03840
C -3.26709 0.36065 -1.37898
C -2.25148 0.39794 -2.54680
H -1.93350 -0.61513 -2.84480
H -2.73164 0.86863 -3.42412
H -1.34817 0.97680 -2.28857
C -4.48848 -0.48262 -1.79973
H -5.29374 -0.47086 -1.05134
H -4.89985 -0.05703 -2.73311
H -4.21332 -1.52941 -2.00077
C -3.69156 1.79800 -1.00900
H -4.07462 2.30362 -1.91378
H -4.49991 1.81417 -0.26008
H -2.84575 2.39448 -0.62453
C -0.00009 1.86496 1.02429
H -0.00005 1.51912 2.07305
C -0.00018 3.01029 0.49365
C -0.00032 4.33446 -0.13206
C -0.00055 4.14409 -1.68134
C   1.27296 5.11995 0.29608
H   1.30549 5.26061 1.38844
H   2.19107 4.59793 -0.01705
H   1.26102 6.11582 -0.17789
H   0.89517 3.59956 -2.01921
H -0.00066 5.14192 -2.15143
H -0.89632 3.59949 -2.01894
C -1.27354 5.11983 0.29646
H -1.30577 5.26048 1.38883
H -1.26182 6.11571 -0.17750
H -2.19170 4.59774 -0.01641

C$_{O\text{-}tBu}$
SCF (BP86) Energy = -1387.29375677
Enthalpy 0K = -1386.601980
Enthalpy 298K = -1386.556743
Free Energy 298K = -1386.678182
Lowest Frequency = 9.7432 cm^{-1}
Second Frequency = 19.2155 cm^{-1}

SCF (BP86-D3BJ) Energy = -1387.501871
SCF (FB) Energy = -1387.333129
SCF (DFB) Energy = -1387.338486
SCF (BS2) Energy = -2056.922584

```
Rh -0.00011 -0.10366  0.10463
P   2.28958 -0.43326  0.06845
P  -2.29003 -0.43159  0.06841
N  -0.00085 -2.14190 -0.25206
C   1.17756 -2.83356 -0.38757
C   1.21393 -4.20990 -0.65014
H   2.17975 -4.70815 -0.74463
C  -0.00184 -4.89147 -0.78000
H  -0.00223 -5.96589 -0.98492
C  -1.21712 -4.20899 -0.65027
H  -2.18330 -4.70652 -0.74487
C  -1.17976 -2.83267 -0.38770
O   2.34411 -2.16650 -0.26170
O  -2.34582 -2.16475 -0.26196
C   3.24545 -0.39615  1.73028
C   2.47180 -1.35054  2.67093
H   2.55443 -2.40113  2.34972
H   2.90326 -1.27227  3.68481
H   1.40227 -1.08406  2.73358
C   4.71232 -0.85312  1.60950
H   5.33040 -0.12890  1.05557
H   5.14010 -0.93609  2.62499
H   4.80294 -1.84058  1.12924
C   3.16491  1.04496  2.28496
H   2.12282  1.38469  2.40582
H   3.63835  1.06282  3.28289
H   3.69642  1.77568  1.65541
C   3.28045  0.18231 -1.45392
C   2.27797  0.10823 -2.63139
H   1.37727  0.71565 -2.44103
H   2.77120  0.49335 -3.54224
H   1.96400 -0.92880 -2.83893
C   3.67907  1.65309 -1.20082
H   4.46503  1.74568 -0.43350
H   4.08599  2.07530 -2.13724
H   2.81439  2.27161 -0.90373
C   4.51521 -0.68397 -1.77807
H   4.25171 -1.74656 -1.89584
H   4.93987 -0.33483 -2.73663
H   5.30675 -0.60179 -1.01952
C  -3.24576 -0.39399  1.73032
C  -2.47282 -1.34916  2.67077
H  -1.40305 -1.08361  2.73332
H  -2.90410 -1.27065  3.68471
H  -2.55640 -2.39963  2.34943
C  -3.16402  1.04696  2.28522
H  -3.69487  1.77824  1.65577
H  -3.63749  1.06507  3.28314
H  -2.12164  1.38579  2.40620
C  -4.71301 -0.84973  1.60958
H  -4.80446 -1.83706  1.12920
H  -5.14079 -0.93248  2.62508
H  -5.33052 -0.12495  1.05576
C  -3.28059  0.18486 -1.45380
C  -2.27827  0.11021 -2.63135
H  -1.96505 -0.92701 -2.83905
H  -2.77128  0.49580 -3.54212
H  -1.37711  0.71697 -2.44099
C  -4.51599 -0.68051 -1.77794
H  -5.30741 -0.59786 -1.01933
H  -4.94048 -0.33096 -2.73643
H  -4.25324 -1.74327 -1.89586
C  -3.67815  1.65590 -1.20048
H  -4.08487  2.07850 -2.13680
H  -4.46395  1.74896 -0.43306
H  -2.81300  2.27378 -0.90343
C   0.00066  1.92279  0.21355
H   0.00034  1.25213  1.21864
C   0.00129  3.16668  0.11172
C   0.00212  4.62577 -0.08430
C   0.00422  4.91551 -1.61454
C   1.27220  5.24338  0.56350
H   1.29971  5.05052  1.64844
H   2.18983  4.83173  0.11230
H   1.27217  6.33575  0.40891
H   0.89923  4.49367 -2.09986
H   0.00488  6.00711 -1.77505
H  -0.88988  4.49442 -2.10216
C  -1.26908  5.24450  0.56021
H  -1.29955  5.05168  1.64508
H  -1.26771  6.33687  0.40562
H  -2.18590  4.83364  0.10665
```

TS(C-D)$_{O\text{-}tBu}$
SCF (BP86) Energy = -1387.22994128
Enthalpy 0K = -1386.540024
Enthalpy 298K = -1386.495682
Free Energy 298K = -1386.612452
Lowest Frequency = -48.4631 cm^{-1}
Second Frequency = 16.1993 cm^{-1}
SCF (BP86-D3BJ) Energy = -1387.444468
SCF (FB) Energy = -1387.271412
SCF (DFB) Energy = -1387.277305
SCF (BS2) Energy = -2056.856196

```
Rh  0.00004  0.05567  0.02913
P  -2.31985 -0.28585 -0.00464
P   2.31990 -0.28566 -0.00481
N   0.00011 -2.01684 -0.09852
C  -1.17973 -2.72321 -0.10544
C  -1.21493 -4.12464 -0.12799
H  -2.18123 -4.63070 -0.13362
C   0.00025 -4.81813 -0.13959
H   0.00031 -5.91193 -0.15458
C   1.21536 -4.12449 -0.12896
H   2.18173 -4.63044 -0.13535
C   1.18002 -2.72307 -0.10637
O  -2.34251 -2.05156 -0.10090
O   2.34272 -2.05127 -0.10274
C  -3.32338  0.06976 -1.61398
C  -2.28534  0.04275 -2.76128
H  -1.79425 -0.94091 -2.85111
H  -2.80472  0.24360 -3.71584
H  -1.50079  0.80648 -2.62985
C  -4.40515 -0.99831 -1.88473
H  -5.19082 -1.01527 -1.11597
H  -4.88915 -0.75510 -2.84795
H  -3.97774 -2.00867 -1.96796
C  -3.96401  1.47042 -1.51710
H  -3.23597  2.25308 -1.24906
H  -4.38170  1.73565 -2.50493
```

H	-4.79406	1.49868	-0.79347
C	-3.32192	-0.09004	1.62240
C	-2.46789	-0.78731	2.70863
H	-1.46048	-0.34387	2.77676
H	-2.96655	-0.64545	3.68424
H	-2.38176	-1.87258	2.53558
C	-3.43538	1.41485	1.95551
H	-4.05430	1.96645	1.23065
H	-3.92171	1.51156	2.94293
H	-2.44310	1.89010	2.02155
C	-4.71638	-0.74345	1.54130
H	-4.66831	-1.79785	1.22561
H	-5.16901	-0.71717	2.54898
H	-5.39685	-0.19797	0.86796
C	3.32361	0.07158	-1.61363
C	2.28570	0.04615	-2.76108
H	1.50145	0.81008	-2.62898
H	2.80528	0.24775	-3.71538
H	1.79420	-0.93722	-2.85196
C	3.96450	1.47200	-1.51500
H	4.79469	1.49911	-0.79149
H	4.38206	1.73849	-2.50255
H	3.23667	2.25444	-1.24575
C	4.40518	-0.99640	-1.88553
H	3.97748	-2.00648	-1.97058
H	4.88973	-0.75180	-2.84812
H	5.19046	-1.01483	-1.11641
C	3.32171	-0.09131	1.62260
C	2.46742	-0.78954	2.70802
H	2.38121	-1.87464	2.53396
H	2.96592	-0.64866	3.68385
H	1.46005	-0.34605	2.77640
C	4.71612	-0.74478	1.54114
H	5.39676	-0.19878	0.86839
H	5.16861	-0.71940	2.54891
H	4.66800	-1.79890	1.22453
C	3.43523	1.41323	1.95722
H	3.92140	1.50891	2.94482
H	4.05431	1.96550	1.23302
H	2.44296	1.88848	2.02358
C	0.00047	1.67308	1.43215
H	-0.00027	1.68640	-0.58794
C	0.00004	2.50752	0.42473
C	-0.00031	3.94521	-0.06708
C	-0.00090	4.83362	1.20542
C	-1.26297	4.24045	-0.90975
H	-1.30092	3.60974	-1.81536
H	-2.18026	4.07320	-0.32159
H	-1.25768	5.29382	-1.23830
H	-0.89451	4.64587	1.82185
H	-0.00096	5.89571	0.90652
H	0.89233	4.64616	1.82249
C	1.26253	4.24130	-0.90921
H	1.30141	3.61044	-1.81467
H	1.25654	5.29459	-1.23797
H	2.17967	4.07490	-0.32057

D_{O-tBu}

SCF (BP86) Energy = -1387.23016835
Enthalpy 0K = -1386.539397
Enthalpy 298K = -1386.494342
Free Energy 298K = -1386.612997
Lowest Frequency = 20.1225 cm^{-1}
Second Frequency = 21.2946 cm^{-1}
SCF (BP86-D3BJ) Energy = -1387.445262
SCF (FB) Energy = -1387.272307
SCF (DFB) Energy = -1387.278507
SCF (BS2) Energy = -2056.856702

Rh	-0.00001	0.06477	-0.02880
P	-2.32579	-0.27632	-0.03406
P	2.32571	-0.27651	-0.03399
N	-0.00009	-1.99756	0.11217
C	-1.18032	-2.70298	0.16875
C	-1.21478	-4.09872	0.29607
H	-2.18151	-4.60203	0.34045
C	-0.00023	-4.78965	0.36197
H	-0.00028	-5.87879	0.46369
C	1.21440	-4.09881	0.29651
H	2.18107	-4.60219	0.34123
C	1.18009	-2.70306	0.16919
O	-2.34457	-2.03848	0.09343
O	2.34439	-2.03863	0.09442
C	-3.25397	-0.12195	-1.71741
C	-2.17354	-0.36117	-2.79957
H	-1.74117	-1.37411	-2.73489
H	-2.63946	-0.26347	-3.79693
H	-1.35074	0.37103	-2.73200
C	-4.37739	-1.16724	-1.88396
H	-5.20770	-1.01766	-1.17941
H	-4.78810	-1.06769	-2.90507
H	-4.00469	-2.19648	-1.77032
C	-3.81877	1.30854	-1.84907
H	-3.05790	2.08481	-1.66325
H	-4.18605	1.44930	-2.88148
H	-4.67065	1.48313	-1.17279
C	-3.38963	0.11420	1.51732
C	-2.58589	-0.44978	2.71349
H	-1.57863	-0.00406	2.76963
H	-3.12551	-0.18927	3.64175
H	-2.50479	-1.54841	2.67527
C	-3.51223	1.64819	1.66104
H	-4.09066	2.10783	0.84410
H	-4.04934	1.86160	2.60273
H	-2.52272	2.12831	1.72228
C	-4.78326	-0.54303	1.45373
H	-4.72974	-1.62556	1.25671
H	-5.27163	-0.40678	2.43547
H	-5.43508	-0.07209	0.70069
C	3.25352	-0.12306	-1.71763
C	2.17281	-0.36290	-2.79939
H	1.34983	0.36912	-2.73182
H	2.63840	-0.26544	-3.79692
H	1.74071	-1.37594	-2.73423
C	3.81820	1.30739	-1.85016
H	4.67026	1.48236	-1.17420
H	4.18518	1.44767	-2.88273
H	3.05733	2.08369	-1.66449
C	4.37694	-1.16838	-1.88391
H	4.00439	-2.19757	-1.76935
H	4.78721	-1.06955	-2.90526
H	5.20754	-1.01820	-1.17983
C	3.38991	0.11462	1.51698
C	2.58654	-0.44897	2.71358
H	2.50559	-1.54762	2.67581
H	3.12635	-0.18799	3.64160
H	1.57923	-0.00337	2.76979

C	4.78356	-0.54255	1.45323
H	5.43513	-0.07183	0.69984
H	5.27219	-0.40592	2.43479
H	4.73007	-1.62515	1.25661
C	3.51248	1.64867	1.66004
H	4.05001	1.86251	2.60140
H	4.09050	2.10803	0.84264
H	2.52296	2.12874	1.72154
C	0.00008	1.62175	1.46138
H	-0.00116	1.71522	-0.62866
C	-0.00013	2.42344	0.41612
C	0.00038	3.88810	-0.01737
C	0.00113	4.73477	1.28065
C	-1.26149	4.20841	-0.85225
H	-1.28375	3.62182	-1.78818
H	-2.18158	3.99991	-0.28187
H	-1.26836	5.27603	-1.13148
H	-0.89141	4.52722	1.89248
H	0.00146	5.80659	1.01879
H	0.89393	4.52653	1.89188
C	1.26193	4.20739	-0.85308
H	1.28313	3.62073	-1.78899
H	1.26940	5.27499	-1.13238
H	2.18224	3.99825	-0.28329

TS(C-10)$_{O-tBu}$

SCF (BP86) Energy = -1387.27797551
Enthalpy 0K = -1386.588668
Enthalpy 298K = -1386.543222
Free Energy 298K = -1386.665148
Lowest Frequency = -743.9309 cm^{-1}
Second Frequency = 13.7542 cm^{-1}
SCF (BP86-D3BJ) Energy = -1387.48558
SCF (FB) Energy = -1387.317865
SCF (DFB) Energy = -1387.323448
SCF (BS2) Energy = -2056.905489

Rh	0.00204	-0.08086	0.04590
P	2.28143	-0.40518	0.01550
P	-2.27223	-0.44132	0.02910
N	0.01829	-2.16194	-0.05271
C	1.19825	-2.85383	-0.14084
C	1.24830	-4.25278	-0.21867
H	2.21776	-4.74871	-0.28394
C	0.03944	-4.95880	-0.20994
H	0.04775	-6.05090	-0.27048
C	-1.18000	-4.27638	-0.12588
H	-2.14187	-4.79097	-0.11887
C	-1.15123	-2.87672	-0.04956
O	2.35863	-2.16107	-0.15617
O	-2.32188	-2.20693	0.03571
C	3.26413	-0.22664	1.65732
C	2.49289	-1.08720	2.68654
H	2.56974	-2.16295	2.46174
H	2.93010	-0.91987	3.68733
H	1.42422	-0.81200	2.72845
C	4.72672	-0.70296	1.56709
H	5.34841	-0.02885	0.95658
H	5.15977	-0.71235	2.58389
H	4.80656	-1.72450	1.16239
C	3.19991	1.25566	2.08930
H	2.15806	1.59886	2.20519
H	3.69595	1.36073	3.07092
H	3.71413	1.92994	1.38606
C	3.26910	0.06835	-1.56129
C	2.26862	-0.13128	-2.72581
H	1.35196	0.46667	-2.58633
H	2.75255	0.18128	-3.66907
H	1.97695	-1.18926	-2.83584
C	3.65469	1.56036	-1.45687
H	4.43837	1.73712	-0.70194
H	4.05806	1.89332	-2.43015
H	2.78469	2.19813	-1.22192
C	4.51363	-0.80999	-1.80670
H	4.26285	-1.88209	-1.80984
H	4.92618	-0.55846	-2.80059
H	5.31024	-0.63938	-1.06849
C	-3.30355	-0.14127	1.62167
C	-2.33331	-0.44987	2.78761
H	-1.42041	0.16790	2.73970
H	-2.84721	-0.24364	3.74403
H	-2.02757	-1.50936	2.79413
C	-3.71127	1.34780	1.66129
H	-4.48196	1.58884	0.91110
H	-4.13886	1.57546	2.65425
H	-2.85081	2.02290	1.50890
C	-4.54091	-1.05530	1.74049
H	-4.27392	-2.11888	1.64240
H	-4.98585	-0.91105	2.74173
H	-5.31792	-0.82015	0.99934
C	-3.21874	-0.11749	-1.61110
C	-2.38784	-0.83888	-2.69958
H	-2.42661	-1.93444	-2.58831
H	-2.80705	-0.58632	-3.69016
H	-1.33082	-0.51964	-2.68312
C	-4.66535	-0.64681	-1.62041
H	-5.32856	-0.06122	-0.96401
H	-5.06699	-0.56117	-2.64650
H	-4.72178	-1.70772	-1.32872
C	-3.18793	1.40466	-1.87775
H	-3.62440	1.59721	-2.87445
H	-3.77961	1.97589	-1.14442
H	-2.15696	1.79507	-1.87684
C	-0.01533	1.88808	0.06768
H	0.02882	2.17273	1.21459
C	-0.02879	3.16262	0.02786
C	-0.04652	4.63499	-0.00994
C	-0.13340	5.00922	-1.52411
C	1.25641	5.21745	0.60029
H	1.34951	4.95439	1.66700
H	2.14719	4.84683	0.06837
H	1.23799	6.31732	0.52217
H	0.73694	4.63204	-2.08434
H	-0.14831	6.10907	-1.60553
H	-1.05272	4.61498	-1.98554
C	-1.28929	5.18762	0.73846
H	-1.25866	4.92579	1.80923
H	-1.30613	6.28742	0.65885
H	-2.22322	4.79416	0.30640

TS(C-E)$_{O-tBu}$

SCF (BP86) Energy = -1387.28865618
Enthalpy 0K = -1386.598844
Enthalpy 298K = -1386.553762
Free Energy 298K = -1386.675244
Lowest Frequency = -628.8056 cm^{-1}
Second Frequency = 5.9072 cm^{-1}

SCF (BP86-D3BJ) Energy = -1387.498408
SCF (FB) Energy = -1387.329755
SCF (DFB) Energy = -1387.335665
SCF (BS2) Energy = -2056.91727

```
Rh  0.00016 -0.09021 -0.00391
P  -2.29534 -0.42468 -0.02704
P   2.29569 -0.42427 -0.02716
N   0.00029 -2.18115  0.04911
C  -1.17999 -2.87807  0.07563
C  -1.21760 -4.27818  0.12923
H  -2.18176 -4.78816  0.14693
C   0.00055 -4.96925  0.15576
H   0.00065 -6.06235  0.19701
C   1.21857 -4.27797  0.12865
H   2.18282 -4.78780  0.14592
C   1.18072 -2.87786  0.07509
O  -2.34344 -2.19102  0.04706
O   2.34404 -2.19064  0.04603
C  -3.26802 -0.14048 -1.65078
C  -2.48912 -0.91415 -2.74132
H  -2.52381 -2.00343 -2.57923
H  -2.95709 -0.70401 -3.71945
H  -1.43339 -0.59847 -2.79669
C  -4.72157 -0.64980 -1.58504
H  -5.34698 -0.03768 -0.91648
H  -5.16300 -0.58140 -2.59555
H  -4.78156 -1.70278 -1.26621
C  -3.22634  1.37388 -1.96038
H  -2.19872  1.77360 -1.96296
H  -3.65559  1.53600 -2.96521
H  -3.82256  1.96579 -1.24862
C  -3.23750 -0.02782  1.59258
C  -2.20926 -0.30251  2.71768
H  -1.30799  0.32568  2.60980
H  -2.67442 -0.06179  3.69060
H  -1.90572 -1.36281  2.75186
C  -3.59535  1.47495  1.57255
H  -4.40625  1.69890  0.86060
H  -3.95271  1.76562  2.57672
H  -2.72221  2.10617  1.33169
C  -4.48681 -0.90468  1.81303
H  -4.24888 -1.97896  1.76737
H  -4.88570 -0.69103  2.82111
H  -5.28909 -0.69287  1.09168
C   3.26839 -0.13890 -1.65072
C   2.49087 -0.91366 -2.74146
H   1.43469 -0.59949 -2.79702
H   2.95871 -0.70268 -3.71948
H   2.52714 -2.00290 -2.57950
C   3.22470  1.37540 -1.96036
H   3.81759  1.96892 -1.24725
H   3.65594  1.53808 -2.96426
H   2.19615  1.77276 -1.96522
C   4.72265 -0.64611 -1.58451
H   4.78410 -1.69900 -1.26562
H   5.16423 -0.57714 -2.59492
H   5.34698 -0.03302 -0.91584
C   3.23762 -0.02818  1.59282
C   2.20938 -0.30401  2.71765
H   1.90624 -1.36443  2.75116
H   2.67437 -0.06373  3.69076
H   1.30786  0.32390  2.61005
C   4.48714 -0.90489  1.81266
H   5.28938 -0.69232  1.09150
H   4.88594 -0.69185  2.82091
H   4.24949 -1.97920  1.76622
C   3.59502  1.47470  1.57395
H   3.95258  1.76466  2.57826
H   4.40562  1.69945  0.86192
H   2.72166  2.10588  1.33377
C   0.00092  1.88187  0.03925
H   0.00038  0.85786 -1.24304
C   0.00336  3.12441 -0.02113
C   0.00137  4.60136  0.01776
C  -0.27698  5.06756  1.47495
C  -1.11126  5.14585 -0.91984
H  -0.94099  4.83167 -1.96299
H  -2.10549  4.78746 -0.60608
H  -1.11765  6.24904 -0.89070
H  -1.25694  4.70624  1.82762
H  -0.27970  6.17042  1.51662
H   0.49829  4.69721  2.16555
C   1.37978  5.14644 -0.44442
H   1.60490  4.83301 -1.47729
H   1.37696  6.24963 -0.41397
H   2.18890  4.78709  0.21274
```

E_{O-tBu}

SCF (BP86) Energy = -1387.29489217
Enthalpy 0K = -1386.603342
Enthalpy 298K = -1386.559120
Free Energy 298K = -1386.676023
Lowest Frequency = -4.2366 cm^{-1}
Second Frequency = 21.3155 cm^{-1}
SCF (BP86-D3BJ) Energy = -1387.505307
SCF (FB) Energy = -1387.337383
SCF (DFB) Energy = -1387.343817
SCF (BS2) Energy = -2056.922463

```
Rh -0.00045 -0.09834  0.02127
P   2.29497 -0.43061  0.03626
P  -2.29612 -0.42767  0.03653
N  -0.00174 -2.19526 -0.08376
C   1.17757 -2.88729 -0.10822
C   1.21585 -4.28732 -0.16798
H   2.17929 -4.79867 -0.18817
C  -0.00353 -4.97709 -0.19733
H  -0.00424 -6.06998 -0.24312
C  -1.22202 -4.28580 -0.16680
H  -2.18612 -4.79595 -0.18607
C  -1.18195 -2.88582 -0.10713
O   2.34107 -2.19427 -0.07987
O  -2.34456 -2.19135 -0.07785
C   3.29632 -0.16493  1.64423
C   2.60726 -1.02078  2.73253
H   2.67317 -2.09919  2.51753
H   3.11795 -0.83555  3.69401
H   1.54602 -0.74946  2.86567
C   4.77289 -0.58990  1.51024
H   5.33505  0.06584  0.82690
H   5.25117 -0.51008  2.50285
H   4.87878 -1.63369  1.17314
C   3.17809  1.33384  2.00829
H   2.12957  1.67306  2.04170
H   3.62197  1.48520  3.00845
```

H	3.72410	1.98098	1.30430
C	3.19919	0.01963	-1.58990
C	2.13163	-0.18502	-2.69326
H	1.25609	0.47133	-2.53962
H	2.57294	0.07862	-3.67138
H	1.79310	-1.23329	-2.75747
C	3.58673	1.51331	-1.51690
H	4.41560	1.69311	-0.81338
H	3.92982	1.83703	-2.51595
H	2.73028	2.14984	-1.23303
C	4.42123	-0.87373	-1.88335
H	4.15811	-1.94308	-1.88517
H	4.80267	-0.61863	-2.88856
H	5.24393	-0.71749	-1.17100
C	-3.29716	-0.15859	1.64415
C	-2.61124	-1.01649	2.73281
H	-1.54893	-0.74920	2.86564
H	-3.12104	-0.82871	3.69426
H	-2.68135	-2.09475	2.51838
C	-3.17392	1.33987	2.00780
H	-3.71450	1.98938	1.30185
H	-3.62022	1.49331	3.00658
H	-2.12392	1.67424	2.04413
C	-4.77512	-0.57851	1.50987
H	-4.88456	-1.62196	1.17287
H	-5.25327	-0.49698	2.50241
H	-5.33490	0.07915	0.82641
C	-3.19914	0.02192	-1.59054
C	-2.13243	-0.18870	-2.69360
H	-1.79764	-1.23827	-2.75589
H	-2.57292	0.07469	-3.67216
H	-1.25446	0.46476	-2.54140
C	-4.42406	-0.86818	-1.88184
H	-5.24647	-0.70711	-1.17024
H	-4.80432	-0.61466	-2.88790
H	-4.16452	-1.93842	-1.88058
C	-3.58169	1.51699	-1.52067
H	-3.92446	1.83963	-2.52018
H	-4.40938	1.70110	-0.81687
H	-2.72292	2.15126	-1.23871
C	0.00004	1.86675	0.03877
H	-0.00030	-0.05416	1.53655
C	-0.00121	3.10905	0.04208
C	0.00221	4.58752	-0.00033
C	0.25356	5.05659	-1.46052
C	1.13119	5.13253	0.91687
H	0.98070	4.81504	1.96201
H	2.11951	4.77433	0.58422
H	1.13800	6.23602	0.89026
H	1.22622	4.69401	-1.83230
H	0.25735	6.15954	-1.50343
H	-0.53480	4.68765	-2.13731
C	-1.36604	5.13578	0.48759
H	-1.57306	4.81928	1.52330
H	-1.36295	6.23926	0.45978
H	-2.18783	4.77919	-0.15557

$10_{O\text{-}tBu}$

SCF (BP86) Energy = -1387.31173059
Enthalpy 0K = -1386.618202
Enthalpy 298K = -1386.573141
Free Energy 298K = -1386.693896
Lowest Frequency = 12.5499 cm^{-1}
Second Frequency = 21.5117 cm^{-1}
SCF (BP86-D3BJ) Energy = -1387.522322
SCF (FB) Energy = -1387.35165
SCF (DFB) Energy = -1387.357062
SCF (BS2) Energy = -2056.937959

Rh	-0.01781	-0.02705	0.05775
P	2.27166	-0.45351	0.03231
P	-2.32391	-0.29120	0.04299
N	-0.09254	-2.12213	-0.15412
C	1.06183	-2.85268	-0.25620
C	1.05044	-4.24836	-0.39365
H	1.99509	-4.78813	-0.47205
C	-0.19143	-4.89532	-0.42229
H	-0.23024	-5.98351	-0.52850
C	-1.38442	-4.16928	-0.31410
H	-2.36520	-4.64642	-0.33008
C	-1.29600	-2.77653	-0.17772
O	2.24601	-2.20810	-0.22905
O	-2.43132	-2.05755	-0.05789
C	3.20004	-0.41005	1.71023
C	2.31346	-1.22426	2.68283
H	2.28536	-2.29398	2.41943
H	2.73757	-1.14044	3.69943
H	1.27914	-0.83871	2.71190
C	4.61420	-1.01848	1.65221
H	5.31249	-0.39604	1.07044
H	5.01404	-1.07943	2.68062
H	4.61252	-2.03816	1.23480
C	3.24963	1.06303	2.17760
H	2.24876	1.52466	2.19377
H	3.65380	1.09031	3.20550
H	3.90781	1.68438	1.55040
C	3.33351	0.02467	-1.49289
C	2.34449	0.01163	-2.68365
H	1.50037	0.70420	-2.52581
H	2.88255	0.32213	-3.59752
H	1.93744	-0.99743	-2.86617
C	3.87483	1.45470	-1.27516
H	4.67159	1.48670	-0.51465
H	4.31164	1.81710	-2.22288
H	3.08201	2.16533	-0.98408
C	4.48582	-0.96226	-1.77399
H	4.12185	-1.99381	-1.89621
H	4.97431	-0.66259	-2.71874
H	5.25551	-0.95446	-0.98884
C	-3.32838	-0.04344	1.65726
C	-2.35467	-0.43017	2.79672
H	-1.43426	0.17733	2.77669
H	-2.85957	-0.26480	3.76558
H	-2.06938	-1.49464	2.74879
C	-3.69411	1.45409	1.76631
H	-4.47885	1.74293	1.04849
H	-4.09063	1.64831	2.77899
H	-2.82070	2.11247	1.61864
C	-4.58795	-0.93029	1.74019
H	-4.34885	-1.99678	1.60790
H	-5.03240	-0.80689	2.74424
H	-5.35576	-0.65006	1.00497
C	-3.24937	0.15695	-1.57575
C	-2.41766	-0.49777	-2.70516
H	-2.46139	-1.59822	-2.66607
H	-2.83327	-0.18029	-3.67821
H	-1.35967	-0.18261	-2.66807

C -4.70182 -0.35406 -1.62702
H -5.35952 0.18955 -0.93056
H -5.09833 -0.18673 -2.64486
H -4.77212 -1.43304 -1.41562
C -3.19778 1.69374 -1.73165
H -3.59922 1.96033 -2.72578
H -3.80756 2.21769 -0.97916
H -2.16495 2.07445 -1.67000
C   0.02140 1.79668 0.39736
H -0.15272 3.22548 1.84304
C   0.02085 3.06401 0.76341
C   0.23233 4.34936 -0.06318
C   0.42427 4.03357 -1.56043
C   1.48449 5.07555 0.48669
H   1.37566 5.31112 1.55922
H   2.39154 4.46042 0.36205
H   1.63892 6.02605 -0.05344
H   1.30215 3.38721 -1.72917
H   0.58057 4.96762 -2.12610
H -0.46028 3.52817 -1.98350
C -1.01236 5.25071 0.12624
H -1.17500 5.49481 1.19013
H -0.87849 6.20053 -0.42050
H -1.92311 4.76143 -0.25782

TS(B-10)'$_{O-tBu}$
SCF (BP86) Energy = -1387.20741801
Enthalpy 0K = -1386.517314
Enthalpy 298K = -1386.472305
Free Energy 298K = -1386.590702
Lowest Frequency = -164.5946 cm^{-1}
Second Frequency = 19.6956 cm^{-1}
SCF (BP86-D3BJ) Energy = -1387.424559
SCF (FB) Energy = -1387.248829
SCF (DFB) Energy = -1387.254707
SCF (BS2) Energy = -2056.836092

Rh 0.10962 0.06024 -0.12832
P   2.44696 0.11162 -0.07447
P -2.12808 -0.44319 -0.11213
N   0.46497 -1.92281 0.31494
C   1.73192 -2.41617 0.48009
C   1.98784 -3.75694 0.78023
H   3.01780 -4.09474 0.90144
C   0.88620 -4.61738 0.91136
H   1.05164 -5.67373 1.14392
C -0.41526 -4.14306 0.74384
H -1.28793 -4.79125 0.83531
C -0.61621 -2.77863 0.43390
O   2.78067 -1.56446 0.34872
O -1.84182 -2.32070 0.25778
C   3.31856 0.93769 1.42624
C   2.34286 0.72441 2.60889
H   2.24069 -0.34336 2.86427
H   2.73786 1.24567 3.49983
H   1.33533 1.11876 2.38763
C   4.68256 0.30896 1.77968
H   5.45732 0.52146 1.02928
H   5.02628 0.73716 2.73878
H   4.60758 -0.78194 1.90700
C   3.46898 2.44587 1.13519
H   2.51129 2.90717 0.83511
H   3.80975 2.95834 2.05275
H   4.21661 2.64832 0.35095
C   3.43290 0.19412 -1.72074
C   2.80566 -0.90185 -2.61560
H   1.71069 -0.78474 -2.69844
H   3.23541 -0.82031 -3.63015
H   3.02291 -1.91336 -2.23743
C   3.17700 1.57700 -2.36077
H   3.57030 2.40976 -1.75534
H   3.68632 1.61455 -3.34040
H   2.10253 1.74540 -2.54110
C   4.94430 -0.06036 -1.56238
H   5.15311 -0.99019 -1.00986
H   5.39396 -0.16102 -2.56688
H   5.45786 0.77517 -1.06034
C -3.25697 -0.25988 1.45184
C -2.31108 -0.38904 2.66860
H -1.51198 0.37140 2.65092
H -2.89890 -0.25014 3.59404
H -1.84024 -1.38334 2.71517
C -3.97080 1.10296 1.49339
H -4.63686 1.26015 0.63149
H -4.58870 1.14764 2.40851
H -3.25214 1.93177 1.52918
C -4.32190 -1.37921 1.50029
H -3.87948 -2.38210 1.42402
H -4.84508 -1.30790 2.47087
H -5.08467 -1.26075 0.71384
C -3.09097 -0.76601 -1.78441
C -2.06645 -0.62353 -2.93216
H -1.21928 -1.31934 -2.81383
H -2.57344 -0.86673 -3.88347
H -1.66830 0.39939 -3.01425
C -3.68581 -2.19235 -1.83430
H -4.39585 -2.39309 -1.01936
H -4.23786 -2.28630 -2.78694
H -2.90865 -2.96852 -1.81082
C -4.22733 0.26542 -1.95941
H -4.68138 0.11744 -2.95597
H -5.03089 0.12929 -1.21706
H -3.86251 1.30217 -1.90115
C -0.36973 1.79012 -1.02947
H   0.11070 2.52880 -1.66310
C -1.55557 1.46019 -0.54948
C -1.28821 3.89944 0.29027
C -0.20394 4.66340 -0.42363
C -1.01714 3.60109 1.73187
H -1.81970 3.03214 2.22057
H -0.06029 3.06999 1.86494
H -0.92143 4.56843 2.27254
H   0.80839 4.29103 -0.20130
H -0.24521 5.71036 -0.05147
H -0.35814 4.71739 -1.51325
C -2.68260 4.20413 -0.16432
H -2.78997 4.10613 -1.25685
H -3.44871 3.59749 0.33516
H -2.89106 5.27113 0.07418

TS(B-10)"$_{O-tBu}$
SCF (BP86) Energy = -1387.22870948
Enthalpy 0K = -1386.536772
Enthalpy 298K = -1386.492190
Free Energy 298K = -1386.608164
Lowest Frequency = -384.4696 cm^{-1}
Second Frequency = 17.4560 cm^{-1}

SCF (BP86-D3BJ) Energy = -
1387.446754
SCF (FB) Energy = -1387.269765
SCF (DFB) Energy = -1387.27551
SCF (BS2) Energy = -2056.855701

Rh 0.00002 0.13682 -0.08958
P 2.29605 -0.24113 0.01122
P -2.29599 -0.24121 0.01120
N 0.00006 -1.96109 -0.11197
C 1.16989 -2.67117 -0.17955
C 1.21204 -4.06944 -0.27971
H 2.18101 -4.56851 -0.32514
C 0.00011 -4.76663 -0.32809
H 0.00013 -5.85729 -0.40983
C -1.21183 -4.06949 -0.27972
H -2.18079 -4.56859 -0.32515
C -1.16974 -2.67121 -0.17958
O 2.33497 -1.99649 -0.15532
O -2.33484 -1.99657 -0.15537
C 3.23174 -0.14740 1.71370
C 2.12386 -0.19556 2.79181
H 1.54784 -1.13488 2.74392
H 2.59063 -0.13867 3.79224
H 1.40965 0.63915 2.69416
C 4.18288 -1.34755 1.92016
H 4.99471 -1.38258 1.17907
H 4.64796 -1.24504 2.91762
H 3.65000 -2.30914 1.89326
C 4.03247 1.16665 1.83503
H 3.42108 2.06308 1.65141
H 4.42431 1.24458 2.86534
H 4.90027 1.18904 1.15654
C 3.43615 0.14243 -1.48880
C 2.65517 -0.37024 -2.72271
H 1.64356 0.06797 -2.77759
H 3.20667 -0.08540 -3.63700
H 2.55712 -1.46761 -2.71728
C 3.61767 1.67273 -1.59998
H 4.19132 2.09477 -0.75989
H 4.18201 1.89408 -2.52410
H 2.64887 2.19373 -1.66712
C 4.80499 -0.56301 -1.41115
H 4.70160 -1.64974 -1.26471
H 5.33924 -0.40352 -2.36551
H 5.44343 -0.15531 -0.61102
C -3.23175 -0.14753 1.71367
C -2.12390 -0.19535 2.79183
H -1.40994 0.63958 2.69422
H -2.59072 -0.13860 3.79224
H -1.54758 -1.13449 2.74397
C -4.03278 1.16635 1.83488
H -4.90052 1.18854 1.15630
H -4.42476 1.24421 2.86515
H -3.42156 2.06290 1.65132
C -4.18262 -1.34788 1.92019
H -3.64955 -2.30936 1.89328
H -4.64768 -1.24545 2.91767
H -4.99448 -1.38309 1.17914
C -3.43612 0.14229 -1.48881
C -2.65517 -0.37045 -2.72272
H -2.55727 -1.46783 -2.71733
H -3.20660 -0.08551 -3.63702
H -1.64349 0.06762 -2.77757
C -4.80496 -0.56313 -1.41110
H -5.44336 -0.15544 -0.61094
H -5.33925 -0.40363 -2.36543
H -4.70156 -1.64987 -1.26469
C -3.61761 1.67258 -1.60006
H -4.18220 1.89389 -2.52404
H -4.19104 2.09470 -0.75985
H -2.64882 2.19355 -1.66749
C 0.00000 2.05306 -0.67237
C 0.00008 2.71659 -1.75455
H 0.00025 3.30282 -2.65947
C -0.00017 3.65631 0.19616
C -1.27335 3.57398 1.03435
H -1.32303 4.47304 1.67794
H -2.17507 3.56536 0.40257
H -1.26719 2.68010 1.67446
C -0.00026 4.98469 -0.60661
H -0.90022 5.10759 -1.23043
H -0.00040 5.80764 0.13098
H 0.89975 5.10780 -1.23030
C 1.27293 3.57419 1.03449
H 2.17473 3.56561 0.40280
H 1.32246 4.47330 1.67802
H 1.26681 2.68035 1.67466

$TS(B\text{-}F)_{O\text{-}tBu}$
SCF (BP86) Energy = -1387.20634180
Enthalpy 0K = -1386.514360
Enthalpy 298K = -1386.469775
Free Energy 298K = -1386.585035
Lowest Frequency = -263.8705 cm^{-1}
Second Frequency = 14.1794 cm^{-1}
SCF (BP86-D3BJ) Energy = -
1387.432793
SCF (FB) Energy = -1387.246558
SCF (DFB) Energy = -1387.252043
SCF (BS2) Energy = -2056.834875

Rh 0.02204 -0.32970 -0.06580
P 2.35051 0.10112 0.04303
P -2.34739 0.08788 0.00562
N 0.01544 1.77148 -0.29150
C 1.17022 2.50459 -0.18529
C 1.20115 3.90321 -0.28742
H 2.15997 4.41750 -0.20930
C -0.00742 4.58142 -0.47277
H -0.01528 5.67209 -0.55450
C -1.20860 3.86728 -0.53009
H -2.17900 4.35307 -0.64052
C -1.15716 2.47044 -0.41773
O 2.32787 1.86134 0.03634
O -2.31603 1.79217 -0.43697
C 3.49729 -0.04312 -1.52731
C 2.61235 0.41339 -2.71426
H 2.38474 1.49042 -2.65348
H 3.16977 0.24840 -3.65381
H 1.66012 -0.13389 -2.78491
C 4.72461 0.89468 -1.46501
H 5.50320 0.53139 -0.78020
H 5.17101 0.93032 -2.47550
H 4.45453 1.92345 -1.18400
C 3.98110 -1.49535 -1.72013
H 3.16122 -2.22627 -1.79069
H 4.55121 -1.55410 -2.66483

H	4.65922	-1.81543	-0.91249
C	3.28264	-0.11597	1.71773
C	2.31373	0.42392	2.79635
H	1.35070	-0.10910	2.78654
H	2.78108	0.27051	3.78598
H	2.13386	1.50515	2.67829
C	3.54666	-1.62028	1.94843
H	4.24932	-2.04269	1.21131
H	4.01288	-1.74138	2.94312
H	2.61418	-2.20558	1.94338
C	4.60542	0.67575	1.78690
H	4.48792	1.72704	1.48164
H	4.94778	0.67026	2.83739
H	5.40310	0.21437	1.18616
C	-3.73797	-0.36141	-1.30509
C	-3.02116	-0.75619	-2.61481
H	-2.51135	-1.72784	-2.53740
H	-3.77514	-0.84557	-3.41745
H	-2.28938	0.00526	-2.93589
C	-4.62091	-1.53071	-0.81610
H	-5.28296	-1.23439	0.01226
H	-5.26899	-1.84656	-1.65334
H	-4.04817	-2.41698	-0.49992
C	-4.63930	0.86180	-1.60350
H	-4.08176	1.68164	-2.07802
H	-5.42008	0.52827	-2.31104
H	-5.14715	1.25734	-0.71344
C	-3.09659	0.25086	1.77948
C	-2.02230	0.97876	2.62114
H	-1.85645	2.01340	2.27694
H	-2.37799	1.03342	3.66603
H	-1.06208	0.43885	2.61216
C	-4.40396	1.06827	1.80959
H	-5.24335	0.54629	1.32438
H	-4.68833	1.22300	2.86622
H	-4.28573	2.06193	1.34869
C	-3.32462	-1.16665	2.35274
H	-3.62390	-1.06511	3.41187
H	-4.13234	-1.70777	1.83767
H	-2.40516	-1.77236	2.32143
C	0.02772	-1.84069	1.18714
C	0.01987	-2.44581	2.27390
H	0.02097	-2.95252	3.22223
C	0.03503	-2.87583	-0.62366
C	0.21967	-2.38632	-2.06678
H	1.24728	-2.06230	-2.27114
H	0.02446	-3.25953	-2.72714
H	-0.47298	-1.58966	-2.37381
C	-1.31426	-3.56703	-0.45144
H	-1.34827	-4.41612	-1.16296
H	-1.45369	-3.95666	0.56537
H	-2.15258	-2.90236	-0.69944
C	1.19475	-3.79791	-0.24518
H	1.00419	-4.33114	0.69595
H	1.30995	-4.54002	-1.06082
H	2.15001	-3.26156	-0.15304

$F_{O\text{-}tBu}$

SCF (BP86) Energy = -1387.22340978
Enthalpy 0K = -1386.530277
Enthalpy 298K = -1386.485284
Free Energy 298K = -1386.600743
Lowest Frequency = 28.4797 cm^{-1}
Second Frequency = 33.1132 cm^{-1}

Rh	-0.06452	-0.55796	0.01497
P	-2.36827	0.01344	0.09712
P	2.36065	-0.04429	0.20167
N	-0.02653	1.52208	-0.57331
C	-1.17898	2.15985	-0.94057
C	-1.21958	3.52266	-1.26270
H	-2.15997	3.97318	-1.58377
C	-0.04254	4.26691	-1.09928
H	-0.03979	5.33601	-1.33085
C	1.11454	3.66307	-0.59209
H	2.02825	4.22451	-0.39124
C	1.07466	2.28706	-0.31270
O	-2.32427	1.43630	-0.95355
O	2.14700	1.71167	0.27257
C	-2.67802	0.79409	1.85455
C	-1.29968	1.22713	2.40337
H	-0.89230	2.10682	1.88050
H	-1.41483	1.50111	3.46814
H	-0.56461	0.40565	2.35796
C	-3.59594	2.03451	1.84838
H	-4.64880	1.80397	1.64539
H	-3.54865	2.49286	2.85305
H	-3.25912	2.79449	1.12497
C	-3.23049	-0.33280	2.75820
H	-2.59411	-1.23504	2.73068
H	-3.24253	0.03226	3.80110
H	-4.26365	-0.61302	2.50156
C	-4.02939	-0.70455	-0.62853
C	-3.92970	-0.71333	-2.17360
H	-3.10126	-1.31808	-2.56572
H	-4.86465	-1.15018	-2.56732
H	-3.83713	0.30637	-2.57748
C	-4.24824	-2.12841	-0.06215
H	-4.50317	-2.11121	1.00813
H	-5.10141	-2.58485	-0.59487
H	-3.38077	-2.79172	-0.18732
C	-5.24150	0.18115	-0.26134
H	-5.10979	1.22901	-0.57296
H	-6.11820	-0.21711	-0.80355
H	-5.48627	0.14576	0.81026
C	3.08966	-0.41068	1.94997
C	2.01667	-0.01270	2.98610
H	1.10498	-0.61882	2.86938
H	2.42013	-0.21662	3.99452
H	1.76786	1.06036	2.93904
C	3.29021	-1.94646	2.00209
H	4.07767	-2.29930	1.31893
H	3.60382	-2.21475	3.02725
H	2.35227	-2.48711	1.79119
C	4.40154	0.32536	2.28667
H	4.30552	1.41929	2.19588
H	4.65252	0.10228	3.33941
H	5.25263	-0.01556	1.67804
C	3.83721	0.06188	-1.12645
C	3.22303	0.25600	-2.53125
H	2.52498	1.10992	-2.56714
H	4.04378	0.48485	-3.23471
H	2.71184	-0.63722	-2.91462
C	4.72046	1.30571	-0.85588
H	5.20420	1.30317	0.12768
H	5.52210	1.30441	-1.61673
H	4.15859	2.24420	-0.96491
C	4.71635	-1.20713	-1.09391
H	5.42583	-1.15692	-1.93968

H   5.31655 -1.27242 -0.17316
H   4.14802 -2.14417 -1.20389
C -0.30971 -3.11589 1.90056
C -0.18716 -2.16841 1.11474
C   0.00161 -1.94617 -1.69062
H -0.38690 -3.93637 2.59138
C -0.14506 -1.00304 -2.88304
H   0.62319 -0.21851 -2.91159
H -0.04742 -1.59362 -3.81892
H -1.13295 -0.51824 -2.91096
C   1.33391 -2.69578 -1.64953
H   1.38073 -3.36860 -2.53064
H   2.20596 -2.03632 -1.70316
H   1.41029 -3.31908 -0.74615
C -1.11075 -2.99267 -1.65851
H -1.04095 -3.58925 -2.59122
H -1.00697 -3.67408 -0.80313
H -2.11235 -2.55394 -1.63824

B_{C-Ar}
SCF (BP86) Energy = -1703.74570599
Enthalpy 0K = -1702.817243
Enthalpy 298K = -1702.761544
Free Energy 298K = -1702.902945
Lowest Frequency = 10.5454 cm^{-1}
Second Frequency = 12.8652 cm^{-1}
SCF (BP86-D3BJ) Energy = -
1704.034476
SCF (FB) Energy = -1703.785879
SCF (DFB) Energy = -1703.791802
SCF (BS2) Energy = -2373.429615

Rh 1.19341 -0.01302 -0.42300
P   1.40872 -2.36315 -0.21731
P   1.62443 2.32108 -0.40556
N   2.69102 -0.04002 1.10041
C   2.89011 -1.17375 1.85200
C   3.87056 -1.22270 2.85503
H   3.99707 -2.13984 3.43650
C   4.66078 -0.09532 3.10798
H   5.42267 -0.11393 3.89304
C   4.46498 1.05239 2.33265
H   5.07601 1.94575 2.48779
C   3.48622 1.05514 1.32506
C   1.99605 -2.35280 1.58337
H   2.47197 -3.29370 1.90490
H   1.06907 -2.23495 2.17233
C   3.32935 2.24493 0.41577
H   3.58497 3.17871 0.94396
H   4.05332 2.14507 -0.41386
C   2.94499 -3.00647 -1.23415
C   3.90273 -1.80486 -1.42482
H   4.35895 -1.47914 -0.47604
H   4.72424 -2.10943 -2.09966
H   3.38477 -0.93872 -1.87456
C   3.72049 -4.14133 -0.53221
H   3.11611 -5.04420 -0.36373
H   4.57451 -4.43324 -1.17090
H   4.14265 -3.81795 0.43416
C   2.46613 -3.46351 -2.63023
H   1.87372 -2.68355 -3.13855
H   3.35024 -3.67109 -3.26020
H   1.86957 -4.38837 -2.59456
C -0.02040 -3.68232 -0.20291
C -0.96618 -3.33518 0.96970
H -1.27489 -2.27705 0.96585
H -1.88373 -3.94242 0.86757
H -0.52926 -3.58254 1.95188
C -0.81479 -3.56024 -1.52513
H -0.20380 -3.78294 -2.41257
H -1.64433 -4.29050 -1.50555
H -1.24993 -2.55687 -1.64695
C   0.48429 -5.12886 -0.01829
H   1.09275 -5.25315 0.89359
H -0.39196 -5.79545 0.08326
H   1.06538 -5.49118 -0.88057
C   2.04836 3.18675 -2.09125
C   2.79610 2.13198 -2.94178
H   2.20741 1.21109 -3.07024
H   2.99827 2.55851 -3.94127
H   3.77301 1.85354 -2.50911
C   0.73292 3.57271 -2.80313
H   0.22872 4.41853 -2.30896
H   0.96015 3.88941 -3.83736
H   0.02642 2.72741 -2.85596
C   2.95629 4.42833 -1.94628
H   3.91518 4.19952 -1.45145
H   3.20049 4.80337 -2.95720
H   2.47500 5.25230 -1.40118
C   0.61291 3.46775 0.78812
C   0.69405 2.78660 2.17470
H   1.71050 2.81540 2.60279
H   0.02995 3.32437 2.87547
H   0.36436 1.73449 2.13171
C   1.15336 4.90812 0.89384
H   0.96734 5.48978 -0.02254
H   0.62423 5.42425 1.71605
H   2.23115 4.95478 1.12532
C -0.86196 3.49914 0.33164
H -1.45117 4.06792 1.07423
H -0.99211 3.99965 -0.64007
H -1.29587 2.49005 0.26270
C -0.00521 0.01295 -2.14548
H   0.22938 -0.00155 -3.20404
C -0.83120 0.03822 -1.17314
C -2.12892 0.04379 -0.54099
C -3.27873 0.00704 -1.37186
C -2.28125 0.08563 0.86503
H -3.13783 -0.02440 -2.45487
H -1.37494 0.11960 1.47606
C -4.56809 0.01486 -0.81576
C -3.55998 0.08750 1.45368
C -4.67684 0.05363 0.59279
H -5.67790 0.05925 1.03674
C -5.84534 -0.01620 -1.68348
C -6.67700 -1.27712 -1.32626
C -5.52658 -0.05718 -3.19418
H -4.95947 0.83191 -3.52068
H -4.95210 -0.95839 -3.47096
H -6.46718 -0.07710 -3.76960
H -6.10616 -2.19979 -1.52760
H -7.59955 -1.30739 -1.93153
H -6.97480 -1.28873 -0.26452
C -3.77533 0.13017 2.98250
C -2.44239 0.13534 3.76250
C -4.58995 -1.11489 3.42436
H -5.57702 -1.15709 2.93520
H -4.05358 -2.04808 3.18080

H -4.75936 -1.09216 4.51497
H -1.84445 -0.77084 3.55969
H -2.64677 0.16057 4.84609
H -1.82707 1.01965 3.52151
C -4.56130 1.41637 3.35304
H -4.00296 2.32170 3.05915
H -5.54545 1.45311 2.85738
H -4.73381 1.45851 4.44265
C -6.68753 1.25593 -1.39743
H -6.12368 2.17046 -1.64903
H -7.60963 1.24511 -2.00408
H -6.98641 1.32382 -0.33812

TS(B-10) $_{\mathbf{C-Ar}}$
SCF (BP86) Energy = -1703.67519659
Enthalpy 0K = -1702.749993
Enthalpy 298K = -1702.694684
Free Energy 298K = -1702.836213
Lowest Frequency = -650.9261 cm^{-1}
Second Frequency = 7.7380 cm^{-1}
SCF (BP86-D3BJ) Energy = -1703.962758
SCF (FB) Energy = -1703.71588
SCF (DFB) Energy = -1703.721913
SCF (BS2) Energy = -2373.358009

Rh 1.24522 0.08455 -0.36527
P 2.26615 -2.03615 -0.19680
P 0.74762 2.40331 -0.23348
N 3.04278 0.70711 0.58736
C 3.87229 -0.20184 1.19719
C 5.08348 0.19143 1.78704
H 5.71570 -0.56199 2.26447
C 5.45673 1.54039 1.77385
H 6.39229 1.86447 2.23930
C 4.61517 2.46447 1.14534
H 4.88103 3.52374 1.09481
C 3.42426 2.02617 0.54438
C 3.41331 -1.63259 1.25120
H 4.26818 -2.32242 1.33998
H 2.79215 -1.76481 2.15444
C 2.55284 2.97195 -0.23163
H 2.68810 4.01185 0.10572
H 2.85819 2.92979 -1.29226
C 3.45681 -2.43417 -1.68194
C 3.94573 -1.06718 -2.21992
H 4.56852 -0.52885 -1.48585
H 4.57040 -1.24430 -3.11496
H 3.10008 -0.41950 -2.50584
C 4.68502 -3.28249 -1.29054
H 4.42389 -4.27530 -0.89653
H 5.30291 -3.44107 -2.19326
H 5.32768 -2.77193 -0.55365
C 2.64838 -3.13457 -2.79615
H 1.74671 -2.55966 -3.07017
H 3.27827 -3.20552 -3.70145
H 2.35524 -4.16165 -2.52482
C 1.33642 -3.62281 0.43959
C 0.76865 -3.29228 1.84064
H 0.26379 -2.31053 1.85858
H 0.02070 -4.05948 2.11014
H 1.54026 -3.31000 2.62812
C 0.14228 -3.93298 -0.49462
H 0.43203 -4.05173 -1.54967
H -0.32121 -4.88373 -0.17353
H -0.63865 -3.15835 -0.42178
C 2.25363 -4.85957 0.54132
H 3.15667 -4.67048 1.14586
H 1.69542 -5.67583 1.03591
H 2.56483 -5.23384 -0.44670
C 0.00992 3.38090 -1.74353
C 0.87945 3.04530 -2.97936
H 1.01521 1.95769 -3.10692
H 0.36349 3.42480 -3.87981
H 1.86779 3.53452 -2.95287
C -1.42488 2.87663 -2.02065
H -2.11131 3.03575 -1.17641
H -1.82973 3.43113 -2.88685
H -1.43353 1.80679 -2.28076
C -0.00000 4.90777 -1.52069
H 0.99688 5.30849 -1.26990
H -0.32083 5.40005 -2.45703
H -0.70945 5.21457 -0.73564
C 0.06163 3.05424 1.47109
C 0.50723 2.02868 2.54043
H 1.60342 1.99485 2.65488
H 0.08200 2.32516 3.51729
H 0.15890 1.01037 2.29912
C 0.61166 4.44301 1.86262
H 0.37778 5.22768 1.12796
H 0.15161 4.74467 2.82149
H 1.70220 4.42928 2.02538
C -1.48131 3.09160 1.42949
H -1.85818 3.29524 2.44870
H -1.86365 3.89381 0.77836
H -1.91380 2.13157 1.10098
C 0.11142 -0.25678 -2.06767
H -0.43313 -1.52268 -1.67533
C -0.74911 -0.52060 -1.06771
C -2.10997 -0.54674 -0.51042
C -2.31739 -0.67694 0.87846
C -3.21190 -0.48155 -1.39546
H -1.43936 -0.72573 1.52738
H -3.01977 -0.38897 -2.46714
C -3.62224 -0.73480 1.40650
C -4.52684 -0.53690 -0.90222
C -4.69630 -0.66302 0.49480
H -5.71668 -0.71039 0.88985
C -3.90789 -0.88608 2.91641
C -4.75795 0.31703 3.40499
C -2.61174 -0.93578 3.75445
H -1.97744 -1.79728 3.48135
H -2.01395 -0.01403 3.64461
H -2.86516 -1.03975 4.82275
H -4.22180 1.26999 3.25586
H -4.97933 0.21415 4.48143
H -5.72100 0.38564 2.87258
C -5.76340 -0.46879 -1.82422
C -5.37575 -0.33296 -3.31311
C -6.62849 0.75834 -1.43029
H -6.97560 0.70168 -0.38505
H -6.06288 1.69843 -1.54880
H -7.52230 0.81432 -2.07530
H -4.80153 0.58941 -3.50868
H -6.28907 -0.28819 -3.92939
H -4.78281 -1.19438 -3.66661
C -4.69412 -2.20291 3.15707
H -4.11252 -3.08002 2.82531

H	-4.91326	-2.32405	4.23209
H	-5.65564	-2.21297	2.61769
C	-6.59881	-1.76603	-1.65348
H	-6.01160	-2.65717	-1.93355
H	-6.94511	-1.90153	-0.61528
H	-7.49231	-1.72873	-2.30030

TS(B–C)$_{C\text{-}Ar}$

SCF (BP86) Energy = -1703.73631205
Enthalpy 0K = -1702.809367
Enthalpy 298K = -1702.754165
Free Energy 298K = -1702.895027
Lowest Frequency = -36.3608 cm^{-1}
Second Frequency = 8.8987 cm^{-1}
SCF (BP86-D3BJ) Energy = -1704.01612
SCF (FB) Energy = -1703.774232
SCF (DFB) Energy = -1703.77957
SCF (BS2) Energy = -2373.419562

Rh	1.59996	-0.05507	0.24332
P	2.11989	2.21942	0.17555
P	1.58849	-2.38025	0.07123
N	3.49657	-0.25025	-0.69608
C	4.14124	0.84339	-1.22749
C	5.39472	0.73483	-1.84855
H	5.86719	1.63439	-2.25314
C	6.01408	-0.51443	-1.96197
H	6.98697	-0.61653	-2.45131
C	5.35604	-1.62950	-1.43226
H	5.80462	-2.62533	-1.48895
C	4.11386	-1.47602	-0.79794
C	3.43685	2.17449	-1.17420
H	4.16130	3.00335	-1.09909
H	2.88534	2.32031	-2.12177
C	3.44096	-2.66070	-0.15535
H	3.67536	-3.59185	-0.69857
H	3.84181	-2.78020	0.86850
C	3.04792	2.89859	1.73565
C	4.30169	2.00915	1.91087
H	5.04020	2.15437	1.10473
H	4.79790	2.27517	2.86202
H	4.03514	0.93902	1.95204
C	3.47837	4.37407	1.61745
H	2.61967	5.06437	1.62071
H	4.10792	4.63539	2.48798
H	4.07982	4.56964	0.71325
C	2.14133	2.70514	2.97218
H	1.87939	1.64262	3.11014
H	2.68644	3.03491	3.87540
H	1.20935	3.28892	2.91684
C	0.85785	3.49985	-0.54308
C	0.07275	2.73713	-1.63699
H	-0.43646	1.84870	-1.23118
H	-0.68685	3.41343	-2.07132
H	0.72059	2.40518	-2.46714
C	-0.11969	3.93534	0.56995
H	0.36413	4.57570	1.32537
H	-0.93738	4.52894	0.12062
H	-0.57108	3.06874	1.08170
C	1.52196	4.74257	-1.17677
H	2.19842	4.48307	-2.00811
H	0.73092	5.38954	-1.59903
H	2.08481	5.34651	-0.45042
C	1.20970	-3.49306	1.61322
C	1.75226	-2.72342	2.84099
H	1.26877	-1.74082	2.95603
H	1.55186	-3.31554	3.75274
H	2.84276	-2.55926	2.79307
C	-0.31737	-3.66093	1.76563
H	-0.74749	-4.29427	0.97264
H	-0.53338	-4.15492	2.73059
H	-0.83565	-2.68719	1.76241
C	1.89084	-4.87877	1.55424
H	2.98951	-4.80765	1.49369
H	1.65708	-5.42749	2.48511
H	1.53881	-5.49490	0.71435
C	0.79198	-3.07121	-1.55507
C	1.52567	-2.36373	-2.71907
H	2.58169	-2.67035	-2.80388
H	1.02857	-2.63226	-3.66910
H	1.48816	-1.26594	-2.61161
C	0.91294	-4.59811	-1.72403
H	0.30151	-5.15092	-0.99283
H	0.54811	-4.87819	-2.72959
H	1.95471	-4.95360	-1.64776
C	-0.69215	-2.64445	-1.58808
H	-1.12866	-2.92752	-2.56362
H	-1.28913	-3.13609	-0.80407
H	-0.79726	-1.55358	-1.46863
C	-0.18430	0.13825	1.20744
H	0.17587	0.24385	2.25172
C	-1.42010	0.17045	0.88175
C	-2.77274	0.19714	0.50583
C	-3.54064	-1.00710	0.50604
C	-3.39824	1.43285	0.15698
H	-3.03943	-1.93623	0.78496
H	-2.78996	2.33938	0.17247
C	-4.89944	-0.99225	0.16809
C	-4.75425	1.47776	-0.18979
C	-5.47004	0.25679	-0.17515
H	-6.53203	0.28052	-0.44218
C	-5.77717	-2.26154	0.16278
C	-6.94095	-2.08059	1.17414
C	-4.97805	-3.52152	0.56074
H	-4.14856	-3.72161	-0.14009
H	-4.56415	-3.44301	1.58110
H	-5.64293	-4.40093	0.54249
H	-6.55938	-1.93017	2.19830
H	-7.58239	-2.97855	1.17650
H	-7.57953	-1.21785	0.92086
C	-5.47996	2.78483	-0.57259
C	-4.53773	4.00732	-0.52430
C	-6.64939	3.02915	0.41864
H	-7.38543	2.20807	0.40166
H	-6.28037	3.13162	1.45328
H	-7.18271	3.95772	0.15202
H	-4.12740	4.17207	0.48727
H	-5.09684	4.91538	-0.80460
H	-3.69650	3.90662	-1.23248
C	-6.04045	2.65413	-2.01421
H	-5.22983	2.48614	-2.74370
H	-6.75641	1.82035	-2.10635
H	-6.57062	3.57918	-2.29881
C	-6.35622	-2.47730	-1.26110
H	-5.55088	-2.61584	-2.00252
H	-6.99429	-3.37752	-1.27686
H	-6.97609	-1.62655	-1.59013

C_{C-Ar}

SCF (BP86) Energy = -1703.74066861
Enthalpy 0K = -1702.813665
Enthalpy 298K = -1702.757994
Free Energy 298K = -1702.900785
Lowest Frequency = 7.1847 cm^{-1}
Second Frequency = 13.4312 cm^{-1}
SCF (BP86-D3BJ) Energy = -1704.01675
SCF (FB) Energy = -1703.780561
SCF (DFB) Energy = -1703.786443
SCF (BS2) Energy = -2373.426879

Rh	1.69118	0.00607	0.10953
P	2.03275	2.31229	-0.12944
P	1.85518	-2.32410	0.25398
N	3.77072	-0.09845	-0.22526
C	4.46181	0.98727	-0.71253
C	5.84131	0.92710	-0.96293
H	6.35195	1.81551	-1.34456
C	6.54066	-0.26395	-0.74285
H	7.61454	-0.32732	-0.94130
C	5.83463	-1.37299	-0.26430
H	6.34320	-2.32168	-0.07202
C	4.46012	-1.26959	-0.00338
C	3.69138	2.24380	-1.02752
H	4.31074	3.13989	-0.85230
H	3.44010	2.24107	-2.10452
C	3.70997	-2.43472	0.58671
H	4.15279	-3.39251	0.26557
H	3.80535	-2.39194	1.68793
C	2.39973	3.26191	1.51509
C	3.60716	2.54574	2.16467
H	4.53904	2.67943	1.58976
H	3.77788	2.97545	3.16839
H	3.41898	1.46494	2.28373
C	2.73238	4.75320	1.31059
H	1.86295	5.33316	0.96237
H	3.04546	5.18621	2.27832
H	3.56377	4.91035	0.60214
C	1.17916	3.10783	2.45081
H	0.95593	2.04555	2.64611
H	1.40654	3.58726	3.42024
H	0.27082	3.58383	2.05067
C	0.94044	3.32367	-1.36352
C	0.57604	2.34113	-2.50227
H	0.03041	1.46342	-2.12183
H	-0.06638	2.86577	-3.23309
H	1.46424	1.98305	-3.05267
C	-0.35431	3.76869	-0.65022
H	-0.16804	4.54308	0.11167
H	-1.04225	4.20499	-1.39714
H	-0.86830	2.91593	-0.17678
C	1.66678	4.54962	-1.96053
H	2.57522	4.27370	-2.52167
H	0.98715	5.04427	-2.67832
H	1.94260	5.29734	-1.20273
C	1.11166	-3.28831	1.75959
C	1.20964	-2.34365	2.98126
H	0.59560	-1.43829	2.85365
H	0.84117	-2.87997	3.87469
H	2.24431	-2.02448	3.19579
C	-0.37586	-3.60184	1.48917
H	-0.50460	-4.36712	0.70672
H	-0.83180	-4.00065	2.41355
H	-0.93874	-2.69884	1.19917
C	1.88148	-4.59150	2.07362
H	2.93461	-4.40681	2.34353
H	1.40865	-5.07698	2.94679
H	1.85864	-5.31459	1.24550
C	1.58765	-3.23599	-1.43195
C	2.56332	-2.57222	-2.43272
H	3.62142	-2.76659	-2.18899
H	2.37663	-2.98901	-3.43902
H	2.40976	-1.48054	-2.48539
C	1.86022	-4.75180	-1.37604
H	1.11633	-5.28868	-0.76620
H	1.79821	-5.16422	-2.39990
H	2.86666	-4.99123	-0.99184
C	0.14241	-2.96440	-1.90760
H	0.02173	-3.36394	-2.93111
H	-0.60972	-3.45537	-1.27116
H	-0.07793	-1.88478	-1.92912
C	-0.30960	0.07924	0.27938
H	0.35146	0.13557	1.27726
C	-1.56517	0.08383	0.22944
C	-2.97876	0.07415	0.12344
C	-3.67124	-1.15292	-0.07007
C	-3.71428	1.28724	0.21905
H	-3.08450	-2.07125	-0.14359
H	-3.16209	2.21699	0.37210
C	-5.06980	-1.18182	-0.17478
C	-5.11432	1.28941	0.13115
C	-5.75866	0.04742	-0.06719
H	-6.85134	0.03689	-0.14134
C	-5.86400	-2.48731	-0.39582
C	-6.84060	-2.70025	0.79160
C	-4.94138	-3.72202	-0.49288
H	-4.23833	-3.64477	-1.34070
H	-4.35897	-3.87470	0.43256
H	-5.55106	-4.62724	-0.65153
H	-6.29350	-2.78916	1.74569
H	-7.42302	-3.62608	0.64301
H	-7.55790	-1.86857	0.89048
C	-5.95556	2.57948	0.24047
C	-5.07774	3.83208	0.45426
C	-6.92850	2.45528	1.44344
H	-7.61507	1.59953	1.33238
H	-6.37631	2.32463	2.38972
H	-7.54438	3.36725	1.52940
H	-4.49242	3.77084	1.38836
H	-5.72031	4.72551	0.52613
H	-4.38000	3.99441	-0.38586
C	-6.77060	2.77194	-1.06631
H	-6.10399	2.87062	-1.94008
H	-7.45252	1.92638	-1.25618
H	-7.38520	3.68637	-0.99934
C	-6.67169	-2.37784	-1.71673
H	-6.00205	-2.23210	-2.58152
H	-7.25206	-3.30163	-1.88473
H	-7.38445	-1.53666	-1.69647

TS(C-D)$_{C-Ar}$

SCF (BP86) Energy = -1703.67671836
Enthalpy 0K = -1702.750836
Enthalpy 298K = -1702.695386
Free Energy 298K = -1702.837846
Lowest Frequency = -289.2775 cm^{-1}
Second Frequency = 4.9307 cm^{-1}

SCF (BP86-D3BJ) Energy = -
1703.962095
SCF (FB) Energy = -1703.718223
SCF (DFB) Energy = -1703.724606
SCF (BS2) Energy = -2373.359839

Rh 1.31222 0.07110 -0.22090
P 0.57222 2.28205 -0.45817
P 2.69396 -1.87229 0.04827
N 2.99750 1.01139 0.68404
C 3.18105 2.37106 0.57196
C 4.23034 3.03125 1.22793
H 4.32772 4.11518 1.12231
C 5.15285 2.29561 1.98053
H 5.97885 2.79347 2.49656
C 5.00683 0.90575 2.03629
H 5.71862 0.28845 2.59130
C 3.92942 0.28465 1.38444
C 2.26149 3.11550 -0.35529
H 2.20504 4.18405 -0.09807
H 2.68027 3.05115 -1.37575
C 3.76558 -1.21028 1.45287
H 4.74961 -1.70519 1.51194
H 3.21988 -1.47519 2.37826
C -0.44541 3.12852 0.96447
C -0.05568 2.38200 2.26195
H 1.02190 2.46157 2.48409
H -0.60430 2.82958 3.11081
H -0.31398 1.31196 2.20835
C -0.10808 4.62806 1.12437
H -0.26595 5.20928 0.20255
H -0.77385 5.05332 1.89721
H 0.92533 4.79506 1.47079
C -1.95863 2.96496 0.71237
H -2.24872 1.91690 0.53516
H -2.50339 3.31005 1.60981
H -2.30612 3.57884 -0.13400
C -0.04666 2.74496 -2.23394
C 1.01896 2.23035 -3.23303
H 1.25026 1.16343 -3.06845
H 0.61448 2.33157 -4.25620
H 1.95676 2.81024 -3.20588
C -1.36414 1.99670 -2.52999
H -2.19252 2.29603 -1.87210
H -1.66691 2.21856 -3.56917
H -1.22898 0.90652 -2.44561
C -0.23730 4.26714 -2.40231
H 0.67557 4.83953 -2.16450
H -0.48887 4.47940 -3.45752
H -1.06520 4.65577 -1.78779
C 2.11293 -3.57684 0.77500
C 1.02200 -3.28089 1.83222
H 0.09135 -2.92158 1.36966
H 0.78556 -4.22002 2.36435
H 1.34333 -2.54949 2.59446
C 1.49901 -4.44575 -0.34538
H 2.26263 -4.82493 -1.04296
H 1.01347 -5.32583 0.11429
H 0.73477 -3.89856 -0.92205
C 3.26950 -4.33427 1.46671
H 3.65852 -3.79702 2.34747
H 2.88094 -5.30353 1.82910
H 4.10927 -4.54996 0.79191
C 3.88582 -2.09066 -1.46613
C 4.56883 -0.71968 -1.68695
H 5.25371 -0.44811 -0.86592
H 5.16967 -0.77212 -2.61284
H 3.82954 0.09091 -1.80838
C 4.97630 -3.16323 -1.26186
H 4.56289 -4.18361 -1.23310
H 5.67401 -3.12282 -2.11845
H 5.57639 -2.99925 -0.34997
C 3.03935 -2.42240 -2.71654
H 3.70236 -2.41877 -3.60093
H 2.57250 -3.41663 -2.65707
H 2.23521 -1.68699 -2.87441
C 0.01461 -1.25283 -1.30476
H -0.14395 -0.96789 0.69157
C -0.80295 -1.03312 -0.25486
C -2.24632 -0.85015 -0.04323
C -3.14075 -0.95912 -1.13354
C -2.75958 -0.67118 1.26099
H -2.72950 -1.13407 -2.13282
H -2.05669 -0.61893 2.10033
C -4.53117 -0.88924 -0.93828
C -4.14541 -0.59186 1.49526
C -5.00403 -0.69782 0.37969
H -6.08119 -0.64723 0.54440
C -5.48216 -1.04817 -2.14612
C -5.25692 -2.44462 -2.78599
C -5.17428 0.05385 -3.19448
H -5.33895 1.06023 -2.77231
H -4.13306 0.00038 -3.55480
H -5.83590 -0.05990 -4.07066
H -4.21860 -2.57630 -3.13375
H -5.92148 -2.57387 -3.65784
H -5.47728 -3.25094 -2.06596
C -4.67646 -0.43278 2.93791
C -4.11309 0.87346 3.55729
C -4.21087 -1.64638 3.78714
H -4.60009 -2.59208 3.37346
H -3.11103 -1.72333 3.82936
H -4.57877 -1.54951 4.82338
H -3.01003 0.87356 3.58557
H -4.47310 0.99059 4.59424
H -4.44042 1.75690 2.98233
C -6.96910 -0.93234 -1.74500
H -7.26563 -1.71618 -1.02699
H -7.20414 0.05155 -1.30323
H -7.60207 -1.05141 -2.64036
C -6.21876 -0.36705 2.99382
H -6.61718 0.49258 2.42773
H -6.68568 -1.28726 2.60298
H -6.54479 -0.25255 4.04120

TS(C-10)$_{C-Ar}$
SCF (BP86) Energy = -1703.72209941
Enthalpy 0K = -1702.797832
Enthalpy 298K = -1702.741976
Free Energy 298K = -1702.885532
Lowest Frequency = -659.6256 cm^{-1}
Second Frequency = 6.9136 cm^{-1}
SCF (BP86-D3BJ) Energy = -
1703.997127
SCF (FB) Energy = -1703.760689
SCF (DFB) Energy = -1703.766389
SCF (BS2) Energy = -2373.40685

Rh -1.65729 0.02392 0.05280
P -1.99746 -2.26094 0.37411
P -1.84415 2.31413 -0.32378
N -3.77385 0.06898 -0.10320
C -4.49509 -1.09323 -0.24355
C -5.89504 -1.08367 -0.34259
H -6.42896 -2.03203 -0.44918
C -6.58701 0.13199 -0.32666
H -7.67737 0.15637 -0.41202
C -5.85247 1.31586 -0.20081
H -6.35356 2.28755 -0.17860
C -4.45516 1.26315 -0.07974
C -3.73749 -2.39266 -0.34801
H -4.32139 -3.22578 0.07912
H -3.58955 -2.62610 -1.41880
C -3.66459 2.52870 0.13382
H -4.14173 3.38237 -0.37710
H -3.66551 2.76517 1.21411
C -2.19287 -2.81428 2.22047
C -3.33895 -1.95499 2.80423
H -4.32068 -2.20575 2.36858
H -3.40637 -2.14040 3.89177
H -3.15170 -0.87788 2.65215
C -2.52387 -4.30859 2.40192
H -1.68502 -4.96284 2.11520
H -2.73744 -4.50344 3.46905
H -3.41699 -4.61703 1.83199
C -0.89106 -2.45769 2.97402
H -0.67614 -1.37781 2.89720
H -1.01327 -2.70031 4.04546
H -0.01684 -3.01857 2.60723
C -1.02186 -3.55299 -0.68880
C -0.77954 -2.87055 -2.05638
H -0.22891 -1.92252 -1.94772
H -0.19109 -3.55210 -2.69820
H -1.72047 -2.65405 -2.59180
C 0.34128 -3.84189 -0.02392
H 0.23897 -4.40494 0.91795
H 0.95036 -4.46163 -0.70743
H 0.90471 -2.91542 0.17808
C -1.78705 -4.87653 -0.91181
H -2.75128 -4.72658 -1.42518
H -1.17855 -5.53181 -1.56185
H -1.97557 -5.42625 0.02182
C -0.98340 3.58837 0.85330
C -0.96143 2.92386 2.25028
H -0.42558 1.96147 2.23549
H -0.45372 3.60002 2.96268
H -1.97504 2.73666 2.64571
C 0.46899 3.82417 0.38532
H 0.51727 4.39685 -0.55501
H 1.00210 4.41484 1.15294
H 1.01468 2.87616 0.24609
C -1.72948 4.93813 0.94889
H -2.75688 4.82773 1.33366
H -1.19080 5.59079 1.66027
H -1.77679 5.47077 -0.01218
C -1.73578 2.83400 -2.18470
C -2.80681 1.99606 -2.92221
H -3.83539 2.27892 -2.64116
H -2.70567 2.16610 -4.00960
H -2.67435 0.91700 -2.73127
C -1.98885 4.33275 -2.43955
H -1.18573 4.96912 -2.03475
H -2.02485 4.51095 -3.53010
H -2.95107 4.67835 -2.02391
C -0.34511 2.42172 -2.71940
H -0.30711 2.60970 -3.80804
H 0.47486 2.99259 -2.25681
H -0.15837 1.34824 -2.54987
C 0.28024 0.00242 0.10968
H 0.74275 -0.36501 1.17993
C 1.56330 0.01579 0.09986
C 2.99392 -0.02172 0.06899
C 3.75001 1.02520 0.65352
C 3.65549 -1.10340 -0.56557
H 3.21615 1.84900 1.13670
H 3.04845 -1.89606 -1.01268
C 5.15423 1.00317 0.61753
C 5.05835 -1.14663 -0.63276
C 5.77548 -0.08796 -0.03141
H 6.86557 -0.11343 -0.07124
C 5.95728 2.15052 1.26921
C 5.60882 2.22190 2.78021
C 5.57500 3.49138 0.58690
H 5.81489 3.47249 -0.48982
H 4.49969 3.71513 0.69044
H 6.13464 4.32397 1.04707
H 4.53448 2.40845 2.94818
H 6.16850 3.04334 3.25991
H 5.87389 1.28261 3.29470
C 5.75648 -2.32658 -1.34438
C 5.29888 -2.36869 -2.82709
C 5.35887 -3.65289 -0.64279
H 5.67511 -3.65443 0.41423
H 4.26929 -3.82390 -0.67077
H 5.84331 -4.50781 -1.14539
H 4.20732 -2.49966 -2.91892
H 5.78153 -3.21297 -3.34905
H 5.57333 -1.43946 -3.35463
C 7.48295 1.95298 1.13133
H 7.82689 1.02685 1.62342
H 7.80387 1.92728 0.07564
H 8.00727 2.79497 1.61318
C 7.29591 -2.20419 -1.31413
H 7.64906 -1.29309 -1.82720
H 7.69147 -2.20020 -0.28374
H 7.74311 -3.06779 -1.83401

TS(C-E)$_{C-Ar}$
SCF (BP86) Energy = -1703.73482687
Enthalpy 0K = -1702.809971
Enthalpy 298K = -1702.754397
Free Energy 298K = -1702.897367
Lowest Frequency = -647.5198 cm^{-1}
Second Frequency = 6.7275 cm^{-1}
SCF (BP86-D3BJ) Energy = -1704.011606
SCF (FB) Energy = -1703.777826
SCF (DFB) Energy = -1703.784687
SCF (BS2) Energy = -2373.421257

Rh -1.65788 -0.00230 -0.00240
P -1.89144 -2.30913 0.35446
P -1.93085 2.30512 -0.34502
N -3.78879 -0.00705 0.07171
C -4.46795 -1.12121 0.50434
C -5.87045 -1.15424 0.53363

H	-6.37787	-2.05726	0.88392
C	-6.60147	-0.02797	0.13824
H	-7.69505	-0.03525	0.16642
C	-5.90685	1.10721	-0.29463
H	-6.44332	2.00274	-0.61998
C	-4.50411	1.09379	-0.33422
C	-3.66452	-2.29324	1.01152
C	-3.74424	2.27575	-0.88657
C	-0.92871	-3.22737	1.75633
C	-0.73847	-2.21184	2.90791
H	-1.69115	-1.79107	3.27463
H	-0.26183	-2.73052	3.75938
H	-0.08336	-1.37819	2.61003
C	-1.70104	-4.45678	2.28746
H	-1.88784	-5.21488	1.51286
H	-1.09391	-4.93625	3.07652
H	-2.66559	-4.18588	2.74820
C	0.46102	-3.65292	1.23480
H	1.00636	-2.80490	0.78840
H	1.05903	-4.02770	2.08506
H	0.39813	-4.46922	0.49724
C	-1.96375	-3.30394	-1.30251
C	-3.05849	-2.62324	-2.15891
H	-2.85138	-1.54954	-2.31289
H	-3.07944	-3.10305	-3.15401
H	-4.06704	-2.72513	-1.72421
C	-0.60649	-3.14547	-2.02607
H	0.20920	-3.67494	-1.51125
H	-0.69114	-3.57432	-3.04114
H	-0.31710	-2.08587	-2.12074
C	-2.30787	-4.79546	-1.12136
H	-3.25042	-4.95312	-0.56939
H	-2.43558	-5.25618	-2.11813
H	-1.50565	-5.35024	-0.60968
C	-1.90692	3.40681	1.24067
C	-2.97984	2.81973	2.18814
H	-2.80955	1.74697	2.38123
H	-2.92710	3.35134	3.15533
H	-4.00463	2.94903	1.80101
C	-0.52408	3.27680	1.91745
H	0.28944	3.69006	1.30227
H	-0.53973	3.83707	2.86976
H	-0.27936	2.22697	2.14740
C	-2.22225	4.88874	0.95363
H	-3.17408	5.02641	0.41254
H	-2.31554	5.42629	1.91484
H	-1.42091	5.38308	0.38197
C	-1.04512	3.10347	-1.86478
C	-0.98016	1.98962	-2.93783
H	-1.98086	1.64539	-3.25626
H	-0.47790	2.39067	-3.83701
H	-0.40652	1.11940	-2.57988
C	-1.79797	4.32545	-2.43563
H	-1.86641	5.15975	-1.72208
H	-1.24589	4.69401	-3.31929
H	-2.81681	4.07762	-2.77786
C	0.39408	3.49711	-1.46709
H	0.94379	3.79936	-2.37683
H	0.41490	4.35527	-0.77573
H	0.93659	2.65169	-1.01222
C	0.29734	0.00163	-0.08003
H	-0.67443	0.18491	1.19834
C	1.54451	0.00761	-0.00378
C	2.97106	0.01043	-0.00306
C	3.68578	1.18303	0.35155
C	3.69521	-1.15873	-0.34970
H	3.11454	2.07671	0.62141
H	3.13122	-2.05434	-0.62815
C	5.09067	1.20150	0.37231
C	5.10031	-1.17035	-0.35466
C	5.77110	0.01711	0.01291
H	6.86200	0.01966	0.01915
C	5.83322	2.49440	0.77879
C	5.42256	2.88652	2.22329
C	5.44336	3.63764	-0.19579
H	5.72821	3.38942	-1.23245
H	4.35841	3.83708	-0.18229
H	5.95908	4.57225	0.08629
H	4.33662	3.06104	2.30902
H	5.93652	3.81507	2.52777
H	5.69428	2.09422	2.94147
C	5.85383	-2.45917	-0.75390
C	5.46282	-2.85079	-2.20398
C	5.45737	-3.60580	0.21400
H	5.72825	-3.35792	1.25452
H	4.37364	-3.81035	0.18657
H	5.98093	-4.53752	-0.06310
H	4.37887	-3.03022	-2.30347
H	5.98499	-3.77632	-2.50364
H	5.73953	-2.05592	-2.91736
C	7.36864	2.32990	0.74051
H	7.71803	1.54947	1.43829
H	7.73267	2.07993	-0.27097
H	7.84864	3.27726	1.03864
C	7.38788	-2.28767	-0.69624
H	7.74237	-1.50496	-1.38884
H	7.73809	-2.03705	0.31997
H	7.87593	-3.23250	-0.98930
H	-4.26731	3.22012	-0.65956
H	-3.71968	2.19127	-1.98922
H	-4.19661	-3.24263	0.83198
H	-3.55874	-2.19355	2.10840

E_{C-Ar}

SCF (BP86) Energy = -1703.74219930
Enthalpy 0K = -1702.815783
Enthalpy 298K = -1702.759968
Free Energy 298K = -1702.903927
Lowest Frequency = 5.4161 cm^{-1}
Second Frequency = 10.9140 cm^{-1}
SCF (BP86-D3BJ) Energy = -1704.019347
SCF (FB) Energy = -1703.787548
SCF (DFB) Energy = -1703.795464
SCF (BS2) Energy = -2373.427734

Rh	1.66394	-0.00229	-0.00788
P	1.88623	-2.29887	-0.43372
P	1.93704	2.29449	0.40683
N	3.80386	-0.02383	-0.00976
C	4.48304	-1.12460	-0.46828
C	5.88623	-1.15793	-0.48412
H	6.39763	-2.05251	-0.85016
C	6.61283	-0.04055	-0.05650
H	7.70662	-0.04767	-0.07373
C	5.91444	1.08689	0.39108
H	6.44813	1.97633	0.73726
C	4.51124	1.07142	0.41814

C	3.68380	-2.28171	-1.02240
C	3.74054	2.24134	0.98471
C	0.94717	-3.13127	-1.89979
C	0.81998	-2.05897	-3.00754
H	1.79886	-1.67974	-3.35200
H	0.32478	-2.51603	-3.88332
H	0.20978	-1.20560	-2.67307
C	1.70156	-4.35909	-2.45841
H	1.83266	-5.15872	-1.71448
H	1.11204	-4.78038	-3.29262
H	2.69232	-4.09942	-2.86755
C	-0.46997	-3.53314	-1.43540
H	-1.00228	-2.68536	-0.97298
H	-1.05179	-3.85589	-2.31755
H	-0.45421	-4.38026	-0.73065
C	1.88636	-3.35298	1.18703
C	2.97280	-2.73423	2.09924
H	2.79156	-1.66150	2.29011
H	2.95291	-3.25136	3.07539
H	3.99057	-2.84592	1.68952
C	0.51255	-3.19243	1.87766
H	-0.29951	-3.67716	1.31559
H	0.55677	-3.66707	2.87466
H	0.24070	-2.13214	2.01214
C	2.20051	-4.84385	0.95321
H	3.15301	-5.00158	0.41877
H	2.29151	-5.34759	1.93305
H	1.39904	-5.35715	0.39895
C	1.92480	3.46387	-1.12664
C	3.04547	2.96862	-2.07079
H	2.91965	1.90660	-2.34097
H	3.00471	3.55710	-3.00485
H	4.05337	3.10409	-1.64382
C	0.56295	3.31525	-1.84165
H	-0.27279	3.69763	-1.23621
H	0.58801	3.89657	-2.78104
H	0.34584	2.26426	-2.09211
C	2.18265	4.93982	-0.75919
H	3.12204	5.08205	-0.19769
H	2.26939	5.52836	-1.69054
H	1.35647	5.37579	-0.17591
C	1.01021	3.00347	1.94522
C	0.93663	1.83327	2.95676
H	1.93379	1.47608	3.27251
H	0.41909	2.18243	3.86881
H	0.36441	0.98380	2.54724
C	1.73644	4.20150	2.59514
H	1.81064	5.07139	1.92621
H	1.16014	4.51883	3.48320
H	2.75034	3.94749	2.94764
C	-0.42506	3.39874	1.53510
H	-0.99808	3.64806	2.44649
H	-0.44163	4.29063	0.88786
H	-0.94515	2.57042	1.02570
C	-0.28464	0.00692	0.00153
H	1.62873	0.26254	-1.50193
C	-1.53129	0.00169	-0.02669
C	-2.96025	0.00728	-0.01518
C	-3.67655	1.14575	-0.46378
C	-3.68481	-1.12331	0.43956
H	-3.10550	2.01009	-0.81698
H	-3.12049	-1.99384	0.78849
C	-5.08180	1.16722	-0.47280
C	-5.09015	-1.12998	0.45921
C	-5.76272	0.02218	-0.00416
H	-6.85362	0.02794	0.00021
C	-5.82419	2.42258	-0.98420
C	-5.42786	2.68434	-2.46181
C	-5.42058	3.64586	-0.11851
H	-5.69634	3.49126	0.93866
H	-4.33500	3.83780	-0.15975
H	-5.93538	4.55455	-0.47699
H	-4.34220	2.84491	-2.57394
H	-5.94161	3.58483	-2.84185
H	-5.71043	1.83291	-3.10412
C	-5.84212	-2.37727	0.97634
C	-5.43734	-2.64355	2.45084
C	-5.45856	-3.60471	0.10746
H	-5.74068	-3.44683	-0.94753
H	-4.37481	-3.80836	0.14027
H	-5.98042	-4.50784	0.46974
H	-4.35266	-2.81602	2.55459
H	-5.95794	-3.53840	2.83485
H	-5.70564	-1.78913	3.09534
C	-7.35973	2.26934	-0.91578
H	-7.71921	1.43207	-1.53849
H	-7.71355	2.11043	0.11751
H	-7.83962	3.18905	-1.29097
C	-7.37641	-2.20745	0.91992
H	-7.72198	-1.36646	1.54548
H	-7.73651	-2.04449	-0.11056
H	-7.86329	-3.12200	1.29870
H	4.27109	3.18978	0.79566
H	3.69163	2.12705	2.08396
H	4.20023	-3.23937	-0.84060
H	3.62150	-2.16365	-2.12090

10$_{C\text{-}Ar}$

SCF (BP86) Energy = -1703.75576952

Enthalpy 0K = -1702.826981

Enthalpy 298K = -1702.771327

Free Energy 298K = -1702.914878

Lowest Frequency = 6.7929 cm^{-1}

Second Frequency = 10.8392 cm^{-1}

SCF (BP86-D3BJ) Energy = -1704.034853

SCF (FB) Energy = -1703.797932

SCF (DFB) Energy = -1703.804302

SCF (BS2) Energy = -2373.439885

Rh	-1.50254	0.04525	-0.20774
P	-1.48273	2.38226	0.04315
P	-2.05428	-2.23378	-0.36000
N	-3.58825	0.25777	0.32708
C	-4.05395	1.44386	0.83505
C	-5.40491	1.60363	1.18297
H	-5.74758	2.56126	1.58421
C	-6.29067	0.53105	1.02988
H	-7.34440	0.63730	1.30497
C	-5.80883	-0.67882	0.51723
H	-6.47597	-1.53340	0.37547
C	-4.45572	-0.79159	0.16000
C	-3.06818	2.56402	1.06440
H	-3.55285	3.54469	0.92011
H	-2.73984	2.52979	2.11987
C	-3.93645	-2.05708	-0.47838
H	-4.46871	-2.93999	-0.08542
H	-4.15726	-2.01466	-1.56123

C -1.83747 3.34720 -1.59462
C -3.17668 2.79791 -2.13967
H -4.03628 3.05475 -1.49774
H -3.36541 3.24539 -3.13229
H -3.14385 1.70172 -2.26394
C -1.94224 4.87350 -1.40720
H -0.97590 5.32926 -1.13915
H -2.26179 5.32921 -2.36230
H -2.68804 5.16103 -0.64634
C -0.72056 3.00392 -2.60655
H -0.64612 1.91520 -2.76155
H -0.95946 3.47566 -3.57707
H 0.26710 3.37367 -2.29146
C -0.16652 3.23763 1.16752
C 0.11399 2.23739 2.31332
H 0.47745 1.27117 1.92800
H 0.89119 2.66074 2.97471
H -0.77537 2.04544 2.93945
C 1.13060 3.45512 0.35803
H 1.01244 4.23096 -0.41581
H 1.92550 3.79891 1.04403
H 1.48384 2.52673 -0.12024
C -0.65138 4.57534 1.76963
H -1.55610 4.45986 2.38988
H 0.14122 4.96921 2.43150
H -0.85057 5.34204 1.00693
C -1.63123 -3.23946 -1.95507
C -1.73136 -2.23398 -3.12690
H -1.04941 -1.37917 -2.99283
H -1.46273 -2.75155 -4.06576
H -2.75263 -1.83494 -3.25810
C -0.18035 -3.76086 -1.85640
H -0.07671 -4.56297 -1.10784
H 0.11250 -4.18882 -2.83234
H 0.53287 -2.95623 -1.61303
C -2.60141 -4.41400 -2.21305
H -3.64618 -4.08360 -2.33814
H -2.30867 -4.91038 -3.15616
H -2.57224 -5.17586 -1.42050
C -1.75722 -3.22646 1.27201
C -2.52857 -2.47162 2.38019
H -3.62254 -2.51717 2.24548
H -2.29964 -2.94082 3.35412
H -2.22433 -1.41196 2.43637
C -2.23856 -4.68913 1.21033
H -1.63674 -5.29845 0.51763
H -2.13613 -5.14192 2.21355
H -3.30025 -4.77767 0.92249
C -0.24796 -3.16674 1.59906
H -0.07500 -3.63538 2.58485
H 0.36935 -3.70385 0.86322
H 0.10540 -2.12342 1.64623
C 0.23592 -0.12436 -0.75602
H 1.44905 -0.33754 -2.37325
C 1.45667 -0.24117 -1.27238
C 2.79108 -0.22793 -0.63383
C 3.92773 -0.06020 -1.45306
C 2.96626 -0.39356 0.75864
H 3.78323 0.06202 -2.53252
H 2.08194 -0.55187 1.38570
C 5.22703 -0.04668 -0.91249
C 4.25009 -0.39164 1.33513
C 5.36090 -0.21246 0.48170
H 6.36147 -0.21212 0.91561
C 6.44181 0.13938 -1.84874
C 6.31620 1.49871 -2.58754
C 6.46771 -1.01157 -2.88990
H 6.56236 -1.99253 -2.39373
H 5.55256 -1.03138 -3.50551
H 7.32678 -0.88921 -3.57248
H 5.39625 1.55491 -3.19372
H 7.17368 1.64407 -3.26763
H 6.30160 2.33866 -1.87201
C 4.40958 -0.60619 2.85821
C 3.82388 -1.99101 3.24306
C 3.64264 0.50480 3.62250
H 4.04054 1.50332 3.37240
H 2.56583 0.49785 3.38261
H 3.74379 0.36002 4.71251
H 2.75281 -2.06738 2.98916
H 3.92487 -2.16161 4.32940
H 4.35389 -2.80390 2.71824
C 7.78290 0.12808 -1.08245
H 7.84230 0.94407 -0.34160
H 7.95157 -0.82899 -0.55936
H 8.61513 0.26642 -1.79309
C 5.88657 -0.56641 3.30966
H 6.48555 -1.36099 2.83285
H 6.36022 0.40533 3.08769
H 5.94334 -0.72042 4.40058

TS(B-10)'$_{C-Ar}$
SCF (BP86) Energy = -1703.66028863
Enthalpy 0K = -1702.733191
Enthalpy 298K = -1702.678258
Free Energy 298K = -1702.816169
Lowest Frequency = -290.2984 cm^{-1}
Second Frequency = 17.3842 cm^{-1}
SCF (BP86-D3BJ) Energy = -1703.949513
SCF (FB) Energy = -1703.699365
SCF (DFB) Energy = -1703.70501
SCF (BS2) Energy = -2373.342504

Rh 1.13582 -0.04828 -0.32868
P 1.79062 -2.26059 0.13328
P 1.07590 2.32745 -0.55239
N 3.11657 0.37210 0.25826
C 3.86790 -0.54930 0.95008
C 5.19131 -0.28332 1.33678
H 5.74345 -1.04657 1.89186
C 5.78112 0.94525 1.02441
H 6.80797 1.16877 1.32761
C 5.02639 1.87576 0.30148
H 5.45117 2.83957 0.00808
C 3.71305 1.56633 -0.08029
C 3.21925 -1.85349 1.30700
H 3.96778 -2.65932 1.38646
H 2.74218 -1.75804 2.30028
C 2.91556 2.50714 -0.93243
H 3.28828 3.53994 -0.84872
H 3.01848 2.19801 -1.98792
C 2.61843 -3.13721 -1.38528
C 3.71358 -2.18115 -1.91288
H 4.54808 -2.05761 -1.20222
H 4.13461 -2.60535 -2.84259
H 3.29921 -1.18542 -2.14577
C 3.26160 -4.49415 -1.03295

H 2.51830 -5.25410 -0.74628
H 3.78971 -4.87713 -1.92556
H 4.01061 -4.41326 -0.22669
C 1.55734 -3.32003 -2.49380
H 1.11673 -2.35410 -2.78901
H 2.04642 -3.75941 -3.38276
H 0.73827 -3.99029 -2.19437
C 0.78462 -3.51637 1.23051
C -0.06523 -2.66242 2.19659
H -0.80944 -2.06547 1.64999
H -0.60093 -3.33645 2.89010
H 0.54879 -1.98081 2.81224
C -0.15096 -4.39800 0.37472
H 0.40607 -5.11213 -0.25330
H -0.78728 -4.99423 1.05431
H -0.80152 -3.78495 -0.26607
C 1.72183 -4.42688 2.06129
H 2.32142 -3.86952 2.79907
H 1.09184 -5.13218 2.63312
H 2.40256 -5.02950 1.44211
C 0.29113 3.29142 -2.05544
C 0.77257 2.56864 -3.34049
H 0.68567 1.47060 -3.28077
H 0.16410 2.91269 -4.19586
H 1.82193 2.80569 -3.58194
C -1.25523 3.25254 -1.98970
H -1.65246 3.88140 -1.18041
H -1.65925 3.64933 -2.93868
H -1.66380 2.24108 -1.84870
C 0.74459 4.76665 -2.12299
H 1.84161 4.87702 -2.13874
H 0.36159 5.21376 -3.05884
H 0.34302 5.36764 -1.29183
C 0.86330 3.31983 1.11757
C 1.21431 2.33931 2.26196
H 2.27887 2.05524 2.25051
H 1.01212 2.83384 3.23015
H 0.61997 1.41165 2.20872
C 1.79787 4.54480 1.21887
H 1.64736 5.27552 0.40984
H 1.59285 5.06573 2.17217
H 2.86151 4.25519 1.23717
C -0.60450 3.77137 1.27437
H -0.75885 4.13292 2.30745
H -0.85815 4.60549 0.60092
H -1.31836 2.94925 1.09734
C -0.67047 -0.15053 -1.43528
H -0.87088 0.42860 -2.33279
C -0.61349 -1.37917 -0.90541
C -2.02931 -0.50830 -0.51316
C -3.17600 -0.83929 -1.28989
C -2.19051 0.03418 0.78862
H -3.01945 -1.26791 -2.28320
H -1.28431 0.27953 1.34943
C -4.46644 -0.63565 -0.78990
C -3.47556 0.18790 1.34576
C -4.58150 -0.13419 0.53080
H -5.58619 0.00120 0.94446
C -5.73795 -0.95642 -1.60316
C -6.56866 -2.03061 -0.85072
C -5.40716 -1.49477 -3.01219
H -4.83349 -0.76453 -3.60941
H -4.83568 -2.43829 -2.97099
H -6.34308 -1.70160 -3.55715
H -5.99431 -2.96467 -0.72901
H -7.48550 -2.26454 -1.41859
H -6.87734 -1.68953 0.15160
C -3.71740 0.68003 2.79016
C -2.39906 0.88685 3.56601
C -4.56163 -0.37525 3.55504
H -5.54722 -0.53878 3.08902
H -4.04155 -1.34737 3.59613
H -4.73767 -0.03743 4.59084
H -1.81091 -0.04517 3.63053
H -2.62383 1.20936 4.59647
H -1.76621 1.66626 3.10941
C -6.58223 0.33699 -1.75715
H -6.02073 1.11741 -2.29885
H -7.50271 0.12203 -2.32659
H -6.88483 0.75287 -0.78151
C -4.48867 2.02624 2.75263
H -3.90833 2.80496 2.22842
H -5.46051 1.92895 2.24051
H -4.68625 2.38076 3.77904

TS(B-10)"$_{C-Ar}$
SCF (BP86) Energy = -1703.67908546
Enthalpy 0K = -1702.752845
Enthalpy 298K = -1702.697291
Free Energy 298K = -1702.837622
Lowest Frequency = -346.8243 cm^{-1}
Second Frequency = 8.5270 cm^{-1}
SCF (BP86-D3BJ) Energy = -1703.967604
SCF (FB) Energy = -1703.718401
SCF (DFB) Energy = -1703.724061
SCF (BS2) Energy = -2373.362192

Rh 1.23136 -0.04824 0.12715
P 1.26997 -2.40092 0.07784
P 1.88361 2.21093 0.12536
N 3.21420 -0.28825 -0.68741
C 3.82636 -1.51785 -0.72493
C 5.08382 -1.70888 -1.31839
H 5.52172 -2.71094 -1.32450
C 5.76850 -0.62154 -1.87016
H 6.75137 -0.75134 -2.33235
C 5.16456 0.63786 -1.80583
H 5.66040 1.52127 -2.21776
C 3.89641 0.77870 -1.22076
C 3.13386 -2.66136 -0.04325
H 3.39565 -3.62340 -0.51462
H 3.49224 -2.71048 1.00141
C 3.22791 2.12355 -1.19239
H 3.97256 2.93256 -1.10166
H 2.70025 2.28222 -2.15106
C 0.60836 -3.20991 -1.55822
C 1.37771 -2.51817 -2.70866
H 2.44898 -2.77905 -2.72158
H 0.94792 -2.84797 -3.67210
H 1.28623 -1.41933 -2.65295
C 0.80786 -4.73510 -1.64684
H 0.17889 -5.28170 -0.92578
H 0.51459 -5.07686 -2.65675
H 1.85739 -5.04026 -1.49608
C -0.88720 -2.86294 -1.70241
H -1.06087 -1.77687 -1.62584
H -1.24542 -3.19829 -2.69310

H	-1.50599	-3.36661	-0.94318
C	0.89984	-3.47304	1.66241
C	1.38038	-2.62637	2.86541
H	0.87403	-1.64864	2.90213
H	1.15736	-3.17312	3.80023
H	2.46859	-2.44272	2.85011
C	-0.61368	-3.72035	1.80893
H	-1.02932	-4.31809	0.98148
H	-0.79988	-4.28264	2.74230
H	-1.16998	-2.77278	1.88152
C	1.64384	-4.82813	1.67212
H	2.73884	-4.71707	1.61751
H	1.42344	-5.33969	2.62710
H	1.32404	-5.49896	0.86182
C	0.79801	3.64881	-0.58646
C	-0.04188	3.00296	-1.71427
H	-0.63031	2.14154	-1.36130
H	-0.73660	3.75751	-2.12721
H	0.58791	2.65559	-2.55200
C	-0.12334	4.19602	0.52493
H	0.43376	4.80381	1.25637
H	-0.89170	4.85138	0.07683
H	-0.64054	3.39377	1.07620
C	1.61369	4.81553	-1.18942
H	2.24285	4.49981	-2.03741
H	0.90703	5.57008	-1.58161
H	2.25404	5.32114	-0.45222
C	2.82013	2.75640	1.73796
C	3.97029	1.74291	1.94460
H	4.74629	1.81823	1.16452
H	4.45812	1.95260	2.91408
H	3.59477	0.70583	1.96575
C	3.40716	4.18047	1.67059
H	2.62839	4.95912	1.64846
H	4.01693	4.35834	2.57558
H	4.07125	4.32545	0.80142
C	1.85031	2.63528	2.93499
H	2.40079	2.84949	3.86944
H	1.00930	3.34342	2.87635
H	1.44254	1.61289	3.00693
C	-0.64007	0.18912	0.87038
C	-0.93863	0.35495	2.11111
H	-1.24538	0.52464	3.13321
C	-2.15992	0.23062	0.45981
C	-2.78695	1.46862	0.16960
C	-2.92855	-0.96029	0.44087
H	-2.20270	2.38526	0.24544
H	-2.44603	-1.89980	0.70805
C	-4.13978	1.52178	-0.21204
C	-4.28703	-0.93964	0.07943
C	-4.85701	0.30761	-0.25466
H	-5.91393	0.33753	-0.53823
C	-5.17151	-2.20568	0.06010
C	-4.40787	-3.46586	0.51978
C	-5.68952	-2.44337	-1.38362
H	-6.28219	-1.59022	-1.75366
H	-4.85322	-2.60274	-2.08560
H	-6.33659	-3.33706	-1.41313
H	-3.56274	-3.70230	-0.14977
H	-5.08685	-4.33473	0.50729
H	-4.02210	-3.36266	1.54892
C	-4.86538	2.84186	-0.55226
C	-5.38744	2.77340	-2.01257
C	-3.94056	4.07064	-0.42419
H	-3.55600	4.19249	0.60349
H	-3.08167	4.00997	-1.11496
H	-4.50319	4.98500	-0.67610
H	-4.55633	2.64518	-2.72702
H	-5.91920	3.70586	-2.26944
H	-6.09126	1.93753	-2.16041
C	-6.06300	3.03436	0.41686
H	-6.79345	2.21191	0.34219
H	-5.72230	3.08984	1.46487
H	-6.59414	3.97241	0.18006
C	-6.37883	-2.00190	1.01524
H	-7.00444	-1.14426	0.71794
H	-7.02158	-2.89905	1.00760
H	-6.04212	-1.83173	2.05212

TS(10-F)$_{C\text{-}Ar}$

SCF (BP86) Energy = -1703.65895427
Enthalpy 0K = -1702.732549
Enthalpy 298K = -1702.677166
Free Energy 298K = -1702.815332
Lowest Frequency = -296.5141 cm^{-1}
Second Frequency = 18.3772 cm^{-1}
SCF (BP86-D3BJ) Energy = -1703.960357
SCF (FB) Energy = -1703.700005
SCF (DFB) Energy = -1703.706172
SCF (BS2) Energy = -2373.344556

Rh	0.73740	0.24221	-0.26323
P	2.45130	-1.42146	-0.51146
P	-0.18807	2.43137	0.04324
N	2.12313	0.93237	1.21356
C	2.97391	0.04245	1.82094
C	3.81288	0.44092	2.87363
H	4.46174	-0.29935	3.34935
C	3.81594	1.77639	3.29316
H	4.45928	2.10017	4.11672
C	3.00734	2.69552	2.61560
H	3.01921	3.75675	2.87796
C	2.18078	2.25443	1.57008
C	3.01785	-1.36806	1.29444
C	1.38766	3.21726	0.73789
C	3.94323	-0.71779	-1.56261
C	4.31907	0.68116	-1.02231
H	4.72299	0.65385	0.00311
H	5.10792	1.09892	-1.67383
H	3.45932	1.37040	-1.04924
C	5.19622	-1.61658	-1.49546
H	5.04912	-2.59216	-1.98257
H	6.01910	-1.10962	-2.03196
H	5.54333	-1.78738	-0.46190
C	3.48175	-0.54042	-3.02735
H	2.57904	0.08705	-3.09135
H	4.29331	-0.04360	-3.58995
H	3.26803	-1.49397	-3.52994
C	2.26975	-3.34115	-0.83332
C	0.89918	-3.77673	-0.27850
H	0.06481	-3.26616	-0.78146
H	0.78625	-4.86422	-0.44167
H	0.81471	-3.60710	0.80809
C	2.29723	-3.65131	-2.34582
H	3.28853	-3.49214	-2.79695
H	2.04733	-4.71911	-2.48345
H	1.55161	-3.05627	-2.89651

C 3.37007 -4.14823 -0.10463
H 3.30027 -4.05920 0.99204
H 3.22528 -5.21709 -0.34631
H 4.39085 -3.88078 -0.41258
C -0.49637 3.55007 -1.51891
C 0.43707 3.00461 -2.62405
H 0.21246 1.95642 -2.87420
H 0.30884 3.62218 -3.53224
H 1.50267 3.06340 -2.33934
C -1.96225 3.41960 -1.98018
H -2.66679 3.89393 -1.27818
H -2.07836 3.92777 -2.95457
H -2.26053 2.36840 -2.11519
C -0.16590 5.04050 -1.27322
H 0.89511 5.20739 -1.02553
H -0.36345 5.59505 -2.20881
H -0.78301 5.49834 -0.48678
C -1.46011 2.78797 1.47629
C -0.85463 2.16090 2.75410
H 0.04815 2.68843 3.10367
H -1.60256 2.22477 3.56532
H -0.60860 1.09544 2.60728
C -1.70294 4.29437 1.70658
H -2.26489 4.75770 0.88025
H -2.31451 4.41436 2.61978
H -0.77516 4.86968 1.86502
C -2.80549 2.09104 1.19069
H -3.49849 2.32047 2.02126
H -3.27913 2.44090 0.26151
H -2.69937 0.99840 1.13355
C 0.00135 -0.53272 -1.91539
H -0.24083 -1.24227 -4.10812
C -0.08289 -0.92275 -3.09382
C -1.25007 -0.69096 -0.49890
C -2.45750 -0.35556 -1.14495
C -1.29112 -1.50705 0.65646
H -2.42268 0.23301 -2.06382
H -0.36523 -1.77886 1.16398
C -3.69523 -0.86214 -0.69119
C -2.52235 -1.94238 1.19372
C -3.70334 -1.63179 0.48646
H -4.65389 -2.01739 0.86254
C -4.97536 -0.60746 -1.52314
C -4.79935 -1.26376 -2.91924
C -5.21321 0.91378 -1.70031
H -5.36419 1.40998 -0.72632
H -4.37058 1.40832 -2.21204
H -6.11563 1.08792 -2.31167
H -3.94020 -0.83991 -3.46632
H -5.70220 -1.10201 -3.53372
H -4.63843 -2.35135 -2.82740
C -2.61279 -2.76519 2.49953
C -1.24239 -2.94409 3.18897
C -3.18500 -4.17209 2.17794
H -4.18919 -4.11168 1.72701
H -2.53195 -4.71512 1.47351
H -3.26764 -4.77186 3.10117
H -0.53860 -3.52316 2.56563
H -1.37257 -3.50130 4.13191
H -0.77700 -1.97385 3.43846
C -6.23248 -1.21338 -0.85926
H -6.15776 -2.30929 -0.75512
H -6.42299 -0.78037 0.13805
H -7.11593 -1.00299 -1.48506
C -3.55643 -2.04244 3.49802
H -3.17027 -1.04116 3.75579
H -4.57269 -1.91726 3.09026
H -3.64172 -2.62634 4.43098
H 2.29132 -2.00371 1.83522
H 4.01667 -1.80543 1.45937
H 1.18740 4.15234 1.28522
H 1.99440 3.48522 -0.14758

F_{C-Ar}

SCF (BP86) Energy = -1703.70149825
Enthalpy 0K = -1702.773932
Enthalpy 298K = -1702.718073
Free Energy 298K = -1702.857447
Lowest Frequency = 18.5143 cm^{-1}
Second Frequency = 25.5712 cm^{-1}
SCF (BP86-D3BJ) Energy = -1704.007772
SCF (FB) Energy = -1703.744948
SCF (DFB) Energy = -1703.751885
SCF (BS2) Energy = -2373.387231

Rh 0.93141 0.06133 -0.40716
P 1.20903 -2.30275 -0.42398
P 1.25162 2.40161 0.04118
N 1.38449 -0.16731 1.68931
C 1.31744 -1.41015 2.27046
C 1.60508 -1.59291 3.63174
H 1.53943 -2.59458 4.06522
C 1.95349 -0.48984 4.42117
H 2.16113 -0.61238 5.48831
C 2.04903 0.76768 3.81671
H 2.35046 1.64616 4.39401
C 1.78514 0.90333 2.44282
C 0.88185 -2.57663 1.41613
C 2.05156 2.21948 1.75111
C 3.09269 -2.71270 -0.68930
C 3.89554 -1.84981 0.31280
H 3.69446 -2.11442 1.36382
H 4.97312 -2.01460 0.13180
H 3.70164 -0.77148 0.18201
C 3.43031 -4.19728 -0.44223
H 2.98334 -4.86211 -1.19700
H 4.52666 -4.32364 -0.50909
H 3.12553 -4.54541 0.55943
C 3.50352 -2.30547 -2.12375
H 3.19914 -1.27516 -2.36905
H 4.60350 -2.37240 -2.20740
H 3.07432 -2.96762 -2.88916
C 0.07194 -3.61884 -1.27990
C -1.36605 -3.05921 -1.28856
H -1.44514 -2.15021 -1.90063
H -2.03004 -3.82927 -1.72208
H -1.74716 -2.82228 -0.28137
C 0.52527 -3.82111 -2.74259
H 1.48117 -4.36285 -2.81989
H -0.23570 -4.43241 -3.26048
H 0.60968 -2.86241 -3.28085
C 0.08028 -4.96357 -0.51665
H -0.33729 -4.87140 0.49973
H -0.56860 -5.67083 -1.06451
H 1.07642 -5.42343 -0.44505
C 2.72037 3.11541 -1.01292
C 3.68390 1.92613 -1.24102

H	3.19366	1.10905	-1.79693
H	4.54188	2.27084	-1.84722
H	4.10079	1.53024	-0.29715
C	2.17600	3.59188	-2.37714
H	1.57124	4.50836	-2.28422
H	3.03055	3.83297	-3.03544
H	1.57844	2.80946	-2.87364
C	3.50340	4.25585	-0.32451
H	3.93361	3.96213	0.64805
H	4.35396	4.52759	-0.97610
H	2.90509	5.16484	-0.17563
C	-0.15490	3.70604	0.31690
C	-0.99550	3.22548	1.52192
H	-0.42965	3.24253	2.46896
H	-1.84739	3.91864	1.64527
H	-1.40477	2.21545	1.37044
C	0.39241	5.11711	0.61676
H	0.87124	5.57757	-0.26120
H	-0.45848	5.76411	0.89826
H	1.10487	5.13681	1.45928
C	-1.04603	3.74293	-0.94307
H	-1.87233	4.45614	-0.76944
H	-0.50123	4.08050	-1.83777
H	-1.48835	2.75777	-1.15275
C	0.82062	0.18538	-2.36174
H	0.89603	0.27019	-4.67208
C	0.85604	0.21627	-3.59877
C	-1.10875	0.03579	-0.13567
C	-1.96803	0.30113	-1.21428
C	-1.64993	-0.27583	1.12986
H	-1.54898	0.53394	-2.19368
H	-1.00126	-0.43606	1.98996
C	-3.37605	0.22586	-1.05557
C	-3.04907	-0.35027	1.31815
C	-3.88777	-0.10304	0.21018
H	-4.96998	-0.16626	0.34760
C	-4.28265	0.48492	-2.28232
C	-3.95765	-0.55339	-3.38908
C	-4.02309	1.91230	-2.83108
H	-4.25225	2.67828	-2.07049
H	-2.97552	2.04669	-3.14926
H	-4.66396	2.10369	-3.70934
H	-2.90593	-0.49000	-3.71602
H	-4.59405	-0.37776	-4.27394
H	-4.14517	-1.58163	-3.03501
C	-3.67713	-0.68433	2.69142
C	-2.61857	-0.92185	3.79016
C	-4.53587	-1.97107	2.56102
H	-5.34164	-1.85459	1.81807
H	-3.91767	-2.83277	2.25443
H	-5.00618	-2.21611	3.52930
H	-1.95768	-1.77418	3.55075
H	-3.12076	-1.15603	4.74383
H	-1.98984	-0.03003	3.95920
C	-5.78324	0.36668	-1.93283
H	-6.04522	-0.64289	-1.57208
H	-6.08789	1.10039	-1.16668
H	-6.38757	0.56106	-2.83475
C	-4.58260	0.49368	3.14130
H	-3.99800	1.42280	3.25483
H	-5.39027	0.69020	2.41754
H	-5.05247	0.26449	4.11368
H	-0.22000	-2.66964	1.47745
H	1.30971	-3.51923	1.79530
H	1.79203	3.06607	2.40802
H	3.14274	2.28921	1.58588

B$_{O-Ar}$

SCF (BP86) Energy = -1775.61055155
Enthalpy 0K = -1774.729112
Enthalpy 298K = -1774.673594
Free Energy 298K = -1774.815429
Lowest Frequency = 12.5151 cm^{-1}
Second Frequency = 16.2450 cm^{-1}
SCF (BP86-D3BJ) Energy = -1775.883185
SCF (FB) Energy = -1775.649205
SCF (DFB) Energy = -1775.6546
SCF (BS2) Energy = -2445.323999

Rh	-1.18940	0.14644	-0.34431
P	-0.81360	2.40023	0.16598
P	-2.18416	-1.94705	-0.54915
N	-2.90677	0.49964	0.79166
C	-3.15805	1.74026	1.32220
C	-4.30460	2.01513	2.08277
H	-4.44963	3.01974	2.48235
C	-5.22193	0.98009	2.29384
H	-6.12409	1.16658	2.88379
C	-4.99897	-0.29017	1.74954
H	-5.69663	-1.11794	1.88370
C	-3.83351	-0.49190	0.99675
O	-2.27568	2.73354	1.10976
O	-3.62391	-1.70490	0.44861
C	-1.09170	3.65691	-1.26093
C	-2.40693	3.21283	-1.94490
H	-3.27965	3.35085	-1.28598
H	-2.56431	3.83407	-2.84503
H	-2.36750	2.15543	-2.25851
C	-1.22038	5.11907	-0.79154
H	-0.26132	5.52948	-0.43870
H	-1.54015	5.73600	-1.65104
H	-1.97399	5.23861	0.00308
C	0.08284	3.50466	-2.25465
H	0.21018	2.46098	-2.58719
H	-0.12909	4.12107	-3.14687
H	1.03908	3.85355	-1.83408
C	0.44982	2.97853	1.49806
C	0.48952	1.81805	2.52007
H	0.80594	0.87175	2.05312
H	1.21778	2.06760	3.31257
H	-0.48908	1.66470	3.00601
C	1.83378	3.14778	0.83501
H	1.87070	4.03005	0.17546
H	2.58684	3.30333	1.62842
H	2.14388	2.25894	0.26044
C	0.03017	4.27404	2.22453
H	-0.98189	4.20007	2.65095
H	0.73538	4.44144	3.05885
H	0.07390	5.16106	1.57696
C	-3.07661	-2.43872	-2.18303
C	-3.59207	-1.10504	-2.77618
H	-2.77923	-0.38117	-2.94896
H	-4.08313	-1.31231	-3.74414
H	-4.34021	-0.62840	-2.12059
C	-2.04148	-3.07873	-3.13524
H	-1.74944	-4.09006	-2.80945
H	-2.49331	-3.17683	-4.13869

H -1.12638 -2.47149 -3.24217
C -4.27820 -3.38348 -1.96875
H -4.99934 -2.97337 -1.24507
H -4.80132 -3.50149 -2.93505
H -3.97881 -4.38642 -1.63365
C -1.42438 -3.42569 0.40943
C -1.17717 -2.88777 1.83950
H -2.12175 -2.70340 2.37633
H -0.60730 -3.64330 2.40956
H -0.59344 -1.95040 1.83126
C -2.33599 -4.66654 0.47631
H -2.41831 -5.17613 -0.49661
H -1.89073 -5.38992 1.18352
H -3.34715 -4.42436 0.84034
C -0.07568 -3.77697 -0.25940
H 0.42818 -4.54779 0.35116
H -0.20247 -4.19436 -1.27067
H 0.60003 -2.90909 -0.32485
C -0.01590 -0.06820 -2.06875
H -0.32720 -0.13315 -3.10586
C 0.83372 -0.25139 -1.13643
C 2.15891 -0.39831 -0.58191
C 3.26054 0.00501 -1.37959
C 2.39523 -0.96003 0.69424
H 3.05950 0.43786 -2.36432
H 1.53435 -1.27082 1.29387
C 4.58098 -0.15337 -0.92487
C 3.70595 -1.13905 1.17747
C 4.77376 -0.72485 0.35287
H 5.79414 -0.85463 0.71577
C 3.93632 -1.79292 2.55891
C 3.36447 -3.23595 2.53620
C 3.20857 -0.97061 3.65469
H 3.59665 0.06135 3.69961
H 2.12071 -0.91845 3.47831
H 3.36244 -1.43513 4.64412
H 2.28254 -3.24312 2.31954
H 3.51410 -3.72153 3.51626
H 3.86766 -3.84991 1.77003
C 5.75738 0.28516 -1.82570
C 5.62333 1.79749 -2.14782
C 5.71551 -0.52874 -3.14701
H 5.81359 -1.60993 -2.95053
H 4.77428 -0.36933 -3.69975
H 6.54634 -0.22482 -3.80707
H 4.68051 2.02594 -2.67310
H 6.45341 2.12199 -2.79886
H 5.65608 2.40436 -1.22673
C 5.43341 -1.87247 2.93173
H 6.00616 -2.48759 2.21678
H 5.90031 -0.87379 2.98347
H 5.54097 -2.33882 3.92536
C 7.12955 0.05288 -1.15533
H 7.23465 0.62530 -0.21745
H 7.30716 -1.01368 -0.93431
H 7.93177 0.38494 -1.83533

TS(B-10)$_{O-Ar}$
SCF (BP86) Energy = -1775.54175745
Enthalpy 0K = -1774.663837
Enthalpy 298K = -1774.608648
Free Energy 298K = -1774.750311
Lowest Frequency = -691.6370 cm^{-1}
Second Frequency = 12.7050 cm^{-1}
SCF (BP86-D3BJ) Energy = -1775.813016
SCF (FB) Energy = -1775.58062
SCF (DFB) Energy = -1775.586143
SCF (BS2) Energy = -2445.254441

Rh 1.22629 0.15325 -0.31561
P 2.43428 -1.82378 -0.52638
P 0.61078 2.33554 0.22987
N 2.90990 0.68203 0.79354
C 3.93510 -0.20779 0.99595
C 5.08963 0.12024 1.71956
H 5.86769 -0.63318 1.84967
C 5.19861 1.41528 2.23965
H 6.09058 1.70064 2.80484
C 4.17666 2.34838 2.03547
H 4.22643 3.36836 2.41906
C 3.04731 1.94748 1.30620
O 3.83243 -1.44999 0.47892
O 2.06014 2.84095 1.10924
C 3.35590 -2.14685 -2.18522
C 3.72950 -0.73923 -2.71039
H 4.43307 -0.22199 -2.03620
H 4.23238 -0.85240 -3.68798
H 2.83790 -0.10670 -2.84997
C 4.63905 -2.98686 -2.01540
H 4.43349 -4.03569 -1.75802
H 5.18058 -2.98507 -2.97855
H 5.31138 -2.56335 -1.25318
C 2.38014 -2.82175 -3.17388
H 1.44909 -2.24228 -3.30047
H 2.86454 -2.87206 -4.16565
H 2.13117 -3.85497 -2.88169
C 1.84644 -3.40339 0.39264
C 1.54554 -2.93724 1.83722
H 0.85528 -2.07537 1.85412
H 1.07241 -3.76997 2.38795
H 2.46486 -2.65650 2.37561
C 0.53922 -3.89022 -0.27403
H 0.68622 -4.21821 -1.31546
H 0.15936 -4.76089 0.29043
H -0.25015 -3.12005 -0.25349
C 2.89204 -4.53573 0.41756
H 3.87388 -4.18970 0.77764
H 2.54093 -5.32238 1.11001
H 3.01994 -5.00675 -0.56960
C 0.66449 3.62523 -1.19354
C 2.01987 3.39841 -1.90483
H 2.13560 2.35228 -2.23549
H 2.05821 4.04403 -2.80062
H 2.87275 3.66670 -1.26069
C -0.48410 3.29335 -2.17257
H -1.48087 3.42331 -1.72158
H -0.41878 3.98186 -3.03427
H -0.40125 2.26550 -2.56861
C 0.56588 5.08486 -0.70858
H 1.31835 5.32027 0.06096
H 0.74947 5.75281 -1.56959
H -0.43451 5.32971 -0.31747
C -0.66204 2.74537 1.61814
C -0.58645 1.54637 2.59276
H 0.41282 1.45197 3.05057
H -1.31188 1.70861 3.41072
H -0.83399 0.59556 2.09440

C -0.32178 4.03962 2.38838
H -0.42825 4.94466 1.77405
H -1.02928 4.12939 3.23267
H   0.69674 4.01715 2.80439
C -2.07372 2.83919 1.00037
H -2.81124 2.91510 1.81962
H -2.19171 3.73800 0.37323
H -2.33889 1.95086 0.40330
C   0.11422 -0.04088 -2.07678
H -0.36273 -1.37014 -1.85348
C -0.75236 -0.46240 -1.14434
C -2.12509 -0.58816 -0.63703
C -2.38721 -1.21961 0.59761
C -3.18750 -0.10129 -1.43491
H -1.54273 -1.58581 1.18678
H -2.95067 0.37284 -2.39001
C -3.70893 -1.37084 1.05725
C -4.51957 -0.23483 -1.00371
C -4.74342 -0.86910 0.23847
H -5.77700 -0.98144 0.58255
C -4.05493 -2.06146 2.39387
C -4.81989 -1.06531 3.30569
C -2.79468 -2.53970 3.14760
H -2.22131 -3.28045 2.56333
H -2.12342 -1.70134 3.40443
H -3.09093 -3.02467 4.09257
H -4.20249 -0.17948 3.53358
H -5.08471 -1.55139 4.26044
H -5.75597 -0.71420 2.84072
C -5.71680 0.27059 -1.83738
C -5.26996 0.93494 -3.15813
C -6.51486 1.31338 -1.00991
H -6.89949 0.88847 -0.06776
H -5.88810 2.18581 -0.75732
H -7.38164 1.67332 -1.59039
H -4.62991 1.81714 -2.98185
H -6.15660 1.27909 -3.71597
H -4.72374 0.23228 -3.81105
C -4.95250 -3.29649 2.11205
H -4.43391 -4.02735 1.46836
H -5.21379 -3.79925 3.05915
H -5.89496 -3.01667 1.61294
C -6.63824 -0.93057 -2.18181
H -6.09968 -1.68777 -2.77658
H -7.02834 -1.42549 -1.27686
H -7.50439 -0.58521 -2.77204

TS(B–C)$_{O-Ar}$
SCF (BP86) Energy = -1775.54175745
Enthalpy 0K = -1774.663837
Enthalpy 298K = -1774.608648
Free Energy 298K = -1774.750311
Lowest Frequency = -691.6370 cm^{-1}
Second Frequency = 12.7050 cm^{-1}
SCF (BP86-D3BJ) Energy = -1775.859728
SCF (FB) Energy = -1775.635557
SCF (DFB) Energy = -1775.64073
SCF (BS2) Energy = -2445.311889

Rh 1.22629 0.15325 -0.31561
P   2.43428 -1.82378 -0.52638
P   0.61078 2.33554 0.22987
N   2.90990 0.68203 0.79354
C   3.93510 -0.20779 0.99595
C   5.08963 0.12024 1.71956
H   5.86769 -0.63318 1.84967
C   5.19861 1.41528 2.23965
H   6.09058 1.70064 2.80484
C   4.17666 2.34838 2.03547
H   4.22643 3.36836 2.41906
C   3.04731 1.94748 1.30620
O   3.83243 -1.44999 0.47892
O   2.06014 2.84095 1.10924
C   3.35590 -2.14685 -2.18522
C   3.72950 -0.73923 -2.71039
H   4.43307 -0.22199 -2.03620
H   4.23238 -0.85240 -3.68798
H   2.83790 -0.10670 -2.84997
C   4.63905 -2.98686 -2.01540
H   4.43349 -4.03569 -1.75802
H   5.18058 -2.98507 -2.97855
H   5.31138 -2.56335 -1.25318
C   2.38014 -2.82175 -3.17388
H   1.44909 -2.24228 -3.30047
H   2.86454 -2.87206 -4.16565
H   2.13117 -3.85497 -2.88169
C   1.84644 -3.40339 0.39264
C   1.54554 -2.93724 1.83722
H   0.85528 -2.07537 1.85412
H   1.07241 -3.76997 2.38795
H   2.46486 -2.65650 2.37561
C   0.53922 -3.89022 -0.27403
H   0.68622 -4.21821 -1.31546
H   0.15936 -4.76089 0.29043
H -0.25015 -3.12005 -0.25349
C   2.89204 -4.53573 0.41756
H   3.87388 -4.18970 0.77764
H   2.54093 -5.32238 1.11001
H   3.01994 -5.00675 -0.56960
C   0.66449 3.62523 -1.19354
C   2.01987 3.39841 -1.90483
H   2.13560 2.35228 -2.23549
H   2.05821 4.04403 -2.80062
H   2.87275 3.66670 -1.26069
C -0.48410 3.29335 -2.17257
H -1.48087 3.42331 -1.72158
H -0.41878 3.98186 -3.03427
H -0.40125 2.26550 -2.56861
C   0.56588 5.08486 -0.70858
H   1.31835 5.32027 0.06096
H   0.74947 5.75281 -1.56959
H -0.43451 5.32971 -0.31747
C -0.66204 2.74537 1.61814
C -0.58645 1.54637 2.59276
H   0.41282 1.45197 3.05057
H -1.31188 1.70861 3.41072
H -0.83399 0.59556 2.09440
C -0.32178 4.03962 2.38838
H -0.42825 4.94466 1.77405
H -1.02928 4.12939 3.23267
H   0.69674 4.01715 2.80439
C -2.07372 2.83919 1.00037
H -2.81124 2.91510 1.81962
H -2.19171 3.73800 0.37323
H -2.33889 1.95086 0.40330
C   0.11422 -0.04088 -2.07678
H -0.36273 -1.37014 -1.85348

C -0.75236 -0.46240 -1.14434
C -2.12509 -0.58816 -0.63703
C -2.38721 -1.21961 0.59761
C -3.18750 -0.10129 -1.43491
H -1.54273 -1.58581 1.18678
H -2.95067 0.37284 -2.39001
C -3.70893 -1.37084 1.05725
C -4.51957 -0.23483 -1.00371
C -4.74342 -0.86910 0.23847
H -5.77700 -0.98144 0.58255
C -4.05493 -2.06146 2.39387
C -4.81989 -1.06531 3.30569
C -2.79468 -2.53970 3.14760
H -2.22131 -3.28045 2.56333
H -2.12342 -1.70134 3.40443
H -3.09093 -3.02467 4.09257
H -4.20249 -0.17948 3.53358
H -5.08471 -1.55139 4.26044
H -5.75597 -0.71420 2.84072
C -5.71680 0.27059 -1.83738
C -5.26996 0.93494 -3.15813
C -6.51486 1.31338 -1.00991
H -6.89949 0.88847 -0.06776
H -5.88810 2.18581 -0.75732
H -7.38164 1.67332 -1.59039
H -4.62991 1.81714 -2.98185
H -6.15660 1.27909 -3.71597
H -4.72374 0.23228 -3.81105
C -4.95250 -3.29649 2.11205
H -4.43391 -4.02735 1.46836
H -5.21379 -3.79925 3.05915
H -5.89496 -3.01667 1.61294
C -6.63824 -0.93057 -2.18181
H -6.09968 -1.68777 -2.77658
H -7.02834 -1.42549 -1.27686
H -7.50439 -0.58521 -2.77204

$C_{O\text{-}Ar}$

SCF (BP86) Energy = -1775.60148460
Enthalpy 0K = -1774.721821
Enthalpy 298K = -1774.666087
Free Energy 298K = -1774.811699
Lowest Frequency = 7.1135 cm^{-1}
Second Frequency = 12.7580 cm^{-1}
SCF (BP86-D3BJ) Energy = -1775.860315
SCF (FB) Energy = -1775.639365
SCF (DFB) Energy = -1775.644736
SCF (BS2) Energy = -2445.317225

Rh -1.60177 -0.00223 0.13226
P -1.92050 -2.28748 0.03517
P -1.93614 2.28367 0.08770
N -3.64453 -0.00355 -0.21871
C -4.32615 -1.18036 -0.40293
C -5.70242 -1.21878 -0.66584
H -6.19192 -2.18455 -0.79901
C -6.39554 -0.00507 -0.74522
H -7.46998 -0.00577 -0.94992
C -5.72294 1.20963 -0.56605
H -6.22849 2.17474 -0.62113
C -4.34637 1.17286 -0.30485
O -3.64735 -2.34559 -0.32612
O -3.68839 2.33831 -0.12152
C -1.90770 -3.28849 1.67153
C -2.87578 -2.53731 2.61675
H -3.92148 -2.61288 2.27811
H -2.81241 -2.99295 3.62112
H -2.61224 -1.46921 2.70850
C -2.36555 -4.75087 1.50741
H -1.63297 -5.35750 0.95165
H -2.46818 -5.20324 2.51043
H -3.34420 -4.82662 1.00695
C -0.47565 -3.22544 2.25102
H -0.13153 -2.18802 2.39670
H -0.47536 -3.71791 3.23993
H 0.26172 -3.74909 1.62248
C -1.27413 -3.23655 -1.50163
C -1.33180 -2.20521 -2.65475
H -0.72728 -1.30984 -2.43183
H -0.93221 -2.67504 -3.57177
H -2.36606 -1.88685 -2.86919
C 0.19463 -3.63421 -1.23582
H 0.27968 -4.43601 -0.48403
H 0.63228 -4.01839 -2.17475
H 0.80441 -2.77245 -0.91316
C -2.12845 -4.46646 -1.87178
H -3.18986 -4.20411 -2.00208
H -1.76038 -4.86679 -2.83378
H -2.05681 -5.27550 -1.13078
C -1.81549 3.29424 1.71249
C -2.38390 2.36064 2.80892
H -1.83478 1.40577 2.86595
H -2.29535 2.86926 3.78579
H -3.45068 2.13618 2.64389
C -0.32204 3.57596 1.99078
H 0.09638 4.32306 1.29725
H -0.22222 3.98499 3.01209
H 0.29641 2.66291 1.93834
C -2.63284 4.60236 1.68727
H -3.68159 4.42688 1.40041
H -2.63128 5.03381 2.70453
H -2.20510 5.35695 1.01156
C -1.40800 3.20850 -1.50939
C -1.91487 2.31101 -2.66493
H -3.01571 2.27346 -2.70741
H -1.55739 2.73322 -3.62126
H -1.52755 1.28046 -2.58160
C -2.00845 4.62111 -1.64459
H -1.55241 5.33666 -0.94304
H -1.80573 4.99340 -2.66519
H -3.10045 4.62665 -1.49855
C 0.13637 3.25536 -1.54237
H 0.45481 3.63703 -2.52918
H 0.55107 3.93436 -0.78015
H 0.58245 2.25629 -1.40472
C 0.40917 -0.00565 0.34093
H -0.23213 -0.00437 1.33708
C 1.66376 -0.00728 0.27346
C 3.07473 -0.00065 0.15859
C 3.78010 1.23066 0.08150
C 3.79807 -1.22341 0.12848
H 3.20466 2.16069 0.10836
H 3.23699 -2.16041 0.19383
C 5.17934 1.25572 -0.02587
C 5.19761 -1.23223 0.02271
C 5.85776 0.01567 -0.05281
H 6.94570 0.02200 -0.13517

C	5.91938	2.60947	-0.10806
C	5.61366	3.43609	1.16967
C	5.42358	3.38628	-1.35701
H	5.63262	2.82477	-2.28344
H	4.33839	3.58090	-1.31715
H	5.93568	4.36167	-1.42577
H	4.53437	3.63361	1.28498
H	6.12852	4.41134	1.12324
H	5.95956	2.91015	2.07570
C	5.95754	-2.57709	-0.00620
C	5.47255	-3.41055	-1.22246
C	5.66492	-3.35627	1.30401
H	6.00379	-2.78944	2.18776
H	4.58873	-3.56473	1.42870
H	6.19397	-4.32497	1.29599
H	4.39040	-3.61947	-1.17352
H	5.99892	-4.38028	-1.25269
H	5.67275	-2.88357	-2.17089
C	7.45039	2.43806	-0.21987
H	7.87410	1.91830	0.65674
H	7.73973	1.88230	-1.12847
H	7.92818	3.43042	-0.27576
C	7.48576	-2.38768	-0.12681
H	7.76615	-1.86394	-1.05699
H	7.90250	-1.82763	0.72805
H	7.97811	-3.37434	-0.14425

TS(C–D)$_{O\text{-}Ar}$

SCF (BP86) Energy = -1775.54168760
Enthalpy 0K = -1774.663114
Enthalpy 298K = -1774.608083
Free Energy 298K = -1774.749224
Lowest Frequency = -235.7898 cm^{-1}
Second Frequency = 9.5233 cm^{-1}
SCF (BP86-D3BJ) Energy = -1775.81188
SCF (FB) Energy = -1775.580964
SCF (DFB) Energy = -1775.586755
SCF (BS2) Energy = -2445.254759

Rh	1.24909	0.08209	-0.14872
P	0.47197	2.24323	0.10265
P	2.70742	-1.77984	-0.26832
N	2.97779	0.94551	0.65200
C	3.00550	2.27804	0.98077
C	4.14524	2.90888	1.49665
H	4.09969	3.97091	1.74175
C	5.30126	2.14106	1.67884
H	6.20564	2.60550	2.08208
C	5.30672	0.78393	1.34000
H	6.18811	0.15185	1.45725
C	4.13121	0.22183	0.82137
O	1.88323	3.01134	0.81764
O	4.14410	-1.08024	0.47399
C	-0.80984	2.77567	1.43060
C	-0.63783	1.76331	2.58698
H	0.38368	1.78123	3.00272
H	-1.33492	2.03323	3.40031
H	-0.86954	0.73429	2.27028
C	-0.53630	4.20057	1.96185
H	-0.66483	4.97570	1.19335
H	-1.26655	4.40688	2.76501
H	0.47242	4.29601	2.39075
C	-2.23383	2.68182	0.84039
H	-2.46028	1.69021	0.41531
H	-2.95843	2.86107	1.65471
H	-2.41388	3.44996	0.07096
C	0.36422	3.20092	-1.55657
C	1.73549	3.00357	-2.24707
H	1.98191	1.93538	-2.37403
H	1.68955	3.46008	-3.25205
H	2.55244	3.49510	-1.69415
C	-0.73984	2.53844	-2.41207
H	-1.74043	2.62351	-1.95939
H	-0.77463	3.04925	-3.39111
H	-0.52696	1.47206	-2.59645
C	0.08973	4.70688	-1.36980
H	0.82093	5.18347	-0.69723
H	0.17429	5.19984	-2.35534
H	-0.92643	4.90879	-0.99495
C	2.46575	-3.25712	0.93560
C	2.25646	-2.62100	2.33082
H	1.41473	-1.90749	2.34112
H	2.02902	-3.42505	3.05328
H	3.15890	-2.09684	2.68404
C	1.19736	-4.04257	0.52694
H	1.30420	-4.54332	-0.44746
H	1.01920	-4.82690	1.28445
H	0.29883	-3.40704	0.48470
C	3.69036	-4.19413	0.97359
H	4.62693	-3.64947	1.17284
H	3.54707	-4.92309	1.79197
H	3.80244	-4.77190	0.04281
C	3.45109	-2.24344	-1.97975
C	3.39788	-0.93392	-2.80358
H	4.01783	-0.13708	-2.35671
H	3.80135	-1.13747	-3.81206
H	2.36253	-0.57202	-2.91087
C	4.91295	-2.72959	-1.87957
H	5.00785	-3.69610	-1.36399
H	5.29603	-2.86518	-2.90701
H	5.56095	-1.99807	-1.37297
C	2.55874	-3.31201	-2.64993
H	2.90157	-3.44544	-3.69198
H	2.64116	-4.29247	-2.15308
H	1.50072	-3.00300	-2.68030
C	0.07222	-1.11543	-1.48747
H	-0.11645	-1.04886	0.55637
C	-0.77264	-1.03871	-0.44639
C	-2.22170	-0.89439	-0.23895
C	-3.06808	-0.62218	-1.33865
C	-2.78453	-1.11524	1.03714
H	-2.61506	-0.47331	-2.32366
H	-2.11788	-1.35578	1.87333
C	-4.46406	-0.56629	-1.17816
C	-4.17758	-1.06713	1.23650
C	-4.98850	-0.78576	0.11611
H	-6.07011	-0.74791	0.25312
C	-5.36579	-0.29378	-2.40311
C	-5.16016	-1.42839	-3.44288
C	-4.97491	1.06570	-3.04166
H	-5.11923	1.89615	-2.32903
H	-3.92287	1.07794	-3.37370
H	-5.60402	1.26503	-3.92637
H	-4.11362	-1.48835	-3.78621
H	-5.79407	-1.25128	-4.32896
H	-5.43322	-2.40923	-3.01832
C	-4.76600	-1.35228	2.63681
C	-4.18437	-0.34286	3.66146

```
C  -4.38079 -2.79510  3.06182
H  -4.78139 -3.53797  2.35156
H  -3.28711 -2.93174  3.11254
H  -4.79285 -3.01950  4.06090
H  -3.08369 -0.39702  3.71586
H  -4.57725 -0.55598  4.67058
H  -4.46373  0.69254  3.40039
C  -6.86350 -0.23958 -2.03030
H  -7.22021 -1.19589 -1.61083
H  -7.08010  0.56209 -1.30307
H  -7.46117 -0.03499 -2.93420
C  -6.30581 -1.23364  2.66370
H  -6.64790 -0.22156  2.38648
H  -6.78845 -1.96144  1.98925
H  -6.67258 -1.43839  3.68348
```

TS(C-10)$_{O-Ar}$

SCF (BP86) Energy = -1775.58495610
Enthalpy 0K = -1774.707946
Enthalpy 298K = -1774.652095
Free Energy 298K = -1774.798294
Lowest Frequency = -603.8489 cm^{-1}
Second Frequency = 4.3197 cm^{-1}
SCF (BP86-D3BJ) Energy = -1775.842685
SCF (FB) Energy = -1775.622451
SCF (DFB) Energy = -1775.62787
SCF (BS2) Energy = -2445.299445

```
Rh -1.58239  0.00048  0.04733
P  -1.92320 -2.27475  0.02094
P  -1.91935  2.27663  0.01562
N  -3.66452  0.00207 -0.06910
C  -4.36477 -1.17293 -0.15254
C  -5.76386 -1.21094 -0.23803
H  -6.26880 -2.17596 -0.29972
C  -6.45832  0.00473 -0.24193
H  -7.55013  0.00574 -0.30874
C  -5.76621  1.21915 -0.16276
H  -6.27291  2.18520 -0.16595
C  -4.36713  1.17855 -0.07822
O  -3.68137 -2.33874 -0.15622
O  -3.68646  2.34316  0.00137
C  -1.75888 -3.25144  1.66753
C  -2.61351 -2.46465  2.68990
H  -3.68960 -2.53163  2.46342
H  -2.45248 -2.89668  3.69399
H  -2.32643 -1.39881  2.72486
C  -2.24911 -4.70975  1.58589
H  -1.57879 -5.34185  0.98190
H  -2.26616 -5.13587  2.60555
H  -3.26986 -4.78278  1.17785
C  -0.27679 -3.19730  2.10197
H   0.07725 -2.15789  2.20509
H  -0.17867 -3.68296  3.08952
H   0.39234 -3.72800  1.40590
C  -1.45294 -3.27009 -1.55164
C  -1.65498 -2.27578 -2.72096
H  -1.05741 -1.35816 -2.58668
H  -1.34229 -2.76437 -3.66174
H  -2.71342 -1.98594 -2.83151
C   0.04012 -3.65250 -1.44835
H   0.22003 -4.42834 -0.68594
H   0.36963 -4.06536 -2.41881
H   0.67780 -2.77900 -1.22678
C  -2.33030 -4.51674 -1.78871
H  -3.40283 -4.26745 -1.78853
H  -2.08233 -4.93308 -2.78193
H  -2.15556 -5.31005 -1.04784
C  -1.62804  3.30802  1.60954
C  -1.95758  2.34108  2.77246
H  -1.34981  1.42132  2.72747
H  -1.75267  2.85176  3.73095
H  -3.02088  2.04857  2.77041
C  -0.13523  3.70022  1.66527
H   0.12263  4.46536  0.91490
H   0.08503  4.12886  2.65948
H   0.53140  2.83118  1.52498
C  -2.53066  4.55453  1.71704
H  -3.59566  4.29793  1.60712
H  -2.39335  4.99936  2.71934
H  -2.27960  5.32819  0.97747
C  -1.56623  3.21470 -1.62242
C  -2.28482  2.38972 -2.71712
H  -3.38110  2.44381 -2.61994
H  -2.01345  2.80162 -3.70577
H  -1.98000  1.32866 -2.69219
C  -2.07716  4.66777 -1.64186
H  -1.49300  5.32468 -0.97780
H  -1.97141  5.06664 -2.66718
H  -3.14148  4.73818 -1.36583
C  -0.04121  3.16364 -1.87041
H   0.16880  3.59393 -2.86626
H   0.52744  3.75231 -1.13259
H   0.33933  2.12918 -1.86151
C   0.36399  0.00061  0.07862
H   0.87412 -0.03639  1.21104
C   1.64529 -0.00019  0.08475
C   3.07872 -0.00109  0.06135
C   3.78532 -1.22887  0.03208
C   3.78658  1.22624  0.05451
H   3.21412 -2.16192  0.03669
H   3.21622  2.15953  0.07453
C   5.18965 -1.24646 -0.00708
C   5.19100  1.24280  0.01461
C   5.85945 -0.00202 -0.01382
H   6.95016 -0.00236 -0.04386
C   5.94280  2.59205  0.00265
C   5.57923  3.38961  1.28381
C   5.51367  3.40223 -1.24999
H   5.76408  2.86185 -2.17863
H   4.42932  3.60547 -1.25875
H   6.03534  4.37462 -1.26939
H   4.49716  3.59465  1.34987
H   6.10273  4.36118  1.28656
H   5.87604  2.83962  2.19300
C   5.93981 -2.59622 -0.04272
C   5.50945 -3.38402 -1.30915
C   5.57554 -3.41552  1.22445
H   5.87316 -2.88181  2.14301
H   4.49320 -3.62032  1.28719
H   6.09783 -4.38764  1.21016
H   4.42490 -3.58600 -1.32107
H   6.03007 -4.35648 -1.34561
H   5.76017 -2.82787 -2.22834
C   7.47616  2.41061 -0.03760
H   7.85245  1.86556  0.84528
H   7.80663  1.87632 -0.94501
```

H 7.96274 3.40013 -0.04417
C 7.47338 -2.41596 -0.08025
H 7.80416 -1.86609 -0.97819
H 7.85062 -1.88707 0.81200
H 7.95874 -3.40579 -0.10451

TS(C-E)$_{O-Ar}$
SCF (BP86) Energy = -1775.59461421
Enthalpy 0K = -1774.717044
Enthalpy 298K = -1774.661456
Free Energy 298K = -1774.806629
Lowest Frequency = -629.9917 cm^{-1}
Second Frequency = 5.0932 cm^{-1}
SCF (BP86-D3BJ) Energy = -1775.854929
SCF (FB) Energy = -1775.6356
SCF (DFB) Energy = -1775.641791
SCF (BS2) Energy = -2445.310446

Rh -1.58438 -0.00000 -0.01117
P -1.91767 -2.29434 0.01510
P -1.91774 2.29433 0.01528
N -3.67579 -0.00003 -0.00600
C -4.37342 -1.17999 -0.01316
C -5.77438 -1.21801 -0.02592
H -6.28428 -2.18237 -0.02941
C -6.46652 -0.00008 -0.03222
H -7.56032 -0.00010 -0.04168
C -5.77442 1.21788 -0.02582
H -6.28435 2.18222 -0.02924
C -4.37346 1.17989 -0.01307
O -3.68487 -2.34353 -0.00662
O -3.68495 2.34346 -0.00644
C -1.58469 -3.27111 1.62767
C -2.33293 -2.50103 2.74190
H -3.42593 -2.54088 2.60881
H -2.09390 -2.97204 3.71191
H -2.02127 -1.44401 2.79355
C -2.08790 -4.72718 1.57061
H -1.49159 -5.34684 0.88261
H -1.98878 -5.17201 2.57709
H -3.14913 -4.79168 1.28130
C -0.06287 -3.22268 1.89757
H 0.33164 -2.19306 1.89588
H 0.12800 -3.65667 2.89530
H 0.51247 -3.81244 1.16696
C -1.56683 -3.23409 -1.61707
C -1.87243 -2.20471 -2.73295
H -1.23882 -1.30531 -2.64349
H -1.66189 -2.67016 -3.71271
H -2.93242 -1.89822 -2.73512
C -0.06431 -3.59272 -1.64088
H 0.17996 -4.40225 -0.93393
H 0.19634 -3.95227 -2.65246
H 0.57473 -2.71943 -1.42239
C -2.45059 -4.48245 -1.81406
H -3.52289 -4.24344 -1.73755
H -2.26607 -4.88035 -2.82828
H -2.21931 -5.28569 -1.09983
C -1.58480 3.27098 1.62793
C -2.33301 2.50078 2.74209
H -2.02132 1.44377 2.79365
H -2.09399 2.97171 3.71215
H -3.42602 2.54062 2.60901
C -0.06297 3.22257 1.89782
H 0.51235 3.81242 1.16726
H 0.12788 3.65648 2.89559
H 0.33157 2.19297 1.89603
C -2.08805 4.72704 1.57099
H -3.14928 4.79153 1.28169
H -1.98895 5.17178 2.57752
H -1.49176 5.34677 0.88305
C -1.56694 3.23423 -1.61681
C -1.87251 2.20493 -2.73278
H -2.93249 1.89841 -2.73497
H -1.66199 2.67047 -3.71250
H -1.23888 1.30554 -2.64339
C -2.45073 4.48258 -1.81369
H -2.21947 5.28577 -1.09941
H -2.26625 4.88056 -2.82788
H -3.52303 4.24353 -1.73719
C -0.06443 3.59290 -1.64060
H 0.19620 3.95255 -2.65216
H 0.17982 4.40238 -0.93358
H 0.57464 2.71961 -1.42219
C 0.37738 0.00002 -0.08646
H -0.64232 -0.00004 1.23596
C 1.62336 0.00002 -0.00572
C 3.04887 0.00002 -0.00687
C 3.76729 1.22306 -0.00496
C 3.76730 -1.22301 -0.00475
H 3.19889 2.15814 -0.00637
H 3.19890 -2.15809 -0.00599
C 5.17192 1.24136 -0.00201
C 5.17192 -1.24131 -0.00178
C 5.84648 0.00003 -0.00045
H 6.93747 0.00003 0.00129
C 5.92076 2.59305 -0.00177
C 5.52160 3.39851 1.26349
C 5.52620 3.39624 -1.26993
H 5.80361 2.85025 -2.18766
H 4.44168 3.59458 -1.31035
H 6.04550 4.37044 -1.28137
H 4.43687 3.59660 1.29967
H 6.04051 4.37291 1.27489
H 5.79601 2.85439 2.18324
C 5.92077 -2.59299 -0.00130
C 5.52624 -3.39640 -1.26934
C 5.52158 -3.39824 1.26408
H 5.79597 -2.85397 2.18375
H 4.43684 -3.59634 1.30027
H 6.04049 -4.37265 1.27566
H 4.44172 -3.59474 -1.30975
H 6.04553 -4.37060 -1.28061
H 5.80367 -2.85056 -2.18715
C 7.45541 2.41742 0.00115
H 7.80825 1.87823 0.89716
H 7.81144 1.87695 -0.89282
H 7.93980 3.40837 0.00127
C 7.45541 -2.41737 0.00163
H 7.81146 -1.87705 -0.89243
H 7.80823 -1.87801 0.89755
H 7.93981 -3.40831 0.00193

E$_{O-Ar}$
SCF (BP86) Energy = -1775.60047370
Enthalpy 0K = -1774.721048
Enthalpy 298K = -1774.665442

Free Energy 298K = -1774.809748
Lowest Frequency = 5.3868 cm^{-1}
Second Frequency = 14.0854 cm^{-1}
SCF (BP86-D3BJ) Energy = -1775.861389
SCF (FB) Energy = -1775.643132
SCF (DFB) Energy = -1775.649931
SCF (BS2) Energy = -2445.315253

Rh	-1.59081	0.00005	0.02325
P	-1.92091	-2.29453	0.03172
P	-1.92062	2.29462	0.03045
N	-3.68709	0.00013	-0.07165
C	-4.37910	-1.17941	-0.09063
C	-5.77941	-1.21851	-0.13949
H	-6.29005	-2.18242	-0.15552
C	-6.47047	0.00029	-0.16422
H	-7.56365	0.00036	-0.20163
C	-5.77925	1.21902	-0.14017
H	-6.28976	2.18298	-0.15672
C	-4.37894	1.17976	-0.09129
O	-3.68498	-2.34267	-0.06803
O	-3.68467	2.34295	-0.06935
C	-1.63704	-3.29764	1.63660
C	-2.49100	-2.61809	2.73225
H	-3.57029	-2.69480	2.52541
H	-2.29285	-3.12762	3.69178
H	-2.22935	-1.55426	2.86357
C	-2.04999	-4.77765	1.50408
H	-1.39356	-5.33374	0.81642
H	-1.96015	-5.25587	2.49590
H	-3.09482	-4.89240	1.17320
C	-0.13687	-3.16763	1.99099
H	0.19278	-2.11629	2.03004
H	0.02607	-3.61696	2.98689
H	0.50998	-3.70309	1.27848
C	-1.48512	-3.19908	-1.59921
C	-1.71385	-2.13863	-2.70442
H	-1.06311	-1.25693	-2.56448
H	-1.46075	-2.58366	-3.68361
H	-2.76543	-1.80836	-2.75485
C	0.01282	-3.57322	-1.54572
H	0.21022	-4.39514	-0.83862
H	0.32480	-3.92079	-2.54692
H	0.64631	-2.70897	-1.27921
C	-2.37188	-4.42981	-1.87673
H	-3.44348	-4.17630	-1.86066
H	-2.13061	-4.80929	-2.88609
H	-2.19614	-5.25083	-1.16706
C	-1.63661	3.29859	1.63477
C	-2.49050	2.61964	2.73086
H	-2.22884	1.55589	2.86276
H	-2.29230	3.12972	3.69008
H	-3.56981	2.69623	2.52404
C	-0.13642	3.16876	1.98913
H	0.51037	3.70386	1.27630
H	0.02658	3.61859	2.98479
H	0.19324	2.11744	2.02868
C	-2.04955	4.77853	1.50150
H	-3.09441	4.89312	1.17065
H	-1.95961	5.25729	2.49305
H	-1.39318	5.33424	0.81347
C	-1.48464	3.19813	-1.60101
C	-1.71331	2.13699	-2.70558
H	-2.76489	1.80671	-2.75588
H	-1.46012	2.58142	-3.68502
H	-1.06259	1.25538	-2.56504
C	-2.37135	4.42870	-1.87938
H	-2.19563	5.25017	-1.17021
H	-2.13001	4.80754	-2.88896
H	-3.44296	4.17524	-1.86320
C	0.01330	3.57225	-1.54761
H	0.32540	3.91917	-2.54900
H	0.21064	4.39462	-0.84102
H	0.64674	2.70816	-1.28047
C	0.36468	-0.00001	0.01686
H	-1.55059	0.00056	1.54282
C	1.61113	-0.00005	0.04177
C	3.03858	-0.00009	0.02218
C	3.75866	1.22168	0.01587
C	3.75861	-1.22188	0.01435
H	3.19074	2.15716	0.02333
H	3.19065	-2.15735	0.02066
C	5.16339	1.24053	0.00295
C	5.16334	-1.24076	0.00137
C	5.83886	-0.00013	-0.00443
H	6.92979	-0.00014	-0.01508
C	5.91158	2.59266	-0.00296
C	5.52673	3.39594	1.26808
C	5.50261	3.39821	-1.26499
H	5.77034	2.85410	-2.18674
H	4.41745	3.59520	-1.29290
H	6.02075	4.37306	-1.28047
H	4.44231	3.59295	1.31717
H	6.04524	4.37068	1.27548
H	5.81180	2.85008	2.18354
C	5.91148	-2.59292	-0.00624
C	5.50229	-3.39699	-1.26915
C	5.52676	-3.39765	1.26392
H	5.81197	-2.85286	2.17997
H	4.44235	-3.59468	1.31293
H	6.04524	-4.37242	1.27014
H	4.41712	-3.59391	-1.29715
H	6.02039	-4.37185	-1.28582
H	5.76993	-2.85183	-2.19030
C	7.44631	2.41818	-0.01780
H	7.80942	1.87763	0.87327
H	7.79235	1.87898	-0.91646
H	7.93027	3.40940	-0.02165
C	7.44621	-2.41849	-0.02110
H	7.79212	-1.87819	-0.91914
H	7.80948	-1.87905	0.87057
H	7.93012	-3.40972	-0.02625

10$_{O-Ar}$

SCF (BP86) Energy = -1775.61581242
Enthalpy 0K = -1774.734340
Enthalpy 298K = -1774.678732
Free Energy 298K = -1774.823642
Lowest Frequency = 8.1893 cm^{-1}
Second Frequency = 11.0302 cm^{-1}
SCF (BP86-D3BJ) Energy = -1775.878639
SCF (FB) Energy = -1775.655648
SCF (DFB) Energy = -1775.661348
SCF (BS2) Energy = -2445.329201

Rh	1.42529	-0.00078	0.25099
P	1.33826	2.32504	0.27074

P	2.16466	-2.19217	0.06726
N	3.42481	0.38984	-0.28637
C	3.86837	1.67864	-0.42504
C	5.19326	1.97520	-0.77647
H	5.50259	3.01649	-0.87601
C	6.07235	0.90508	-0.98630
H	7.11159	1.10732	-1.26196
C	5.64092	-0.42042	-0.84645
H	6.30405	-1.27259	-1.00113
C	4.30288	-0.64147	-0.49074
O	3.00139	2.69286	-0.22368
O	3.86715	-1.90990	-0.33536
C	1.32607	3.16895	1.99195
C	2.42796	2.44475	2.80235
H	3.43523	2.64675	2.40315
H	2.39900	2.81493	3.84281
H	2.26702	1.35260	2.82382
C	1.61698	4.68126	1.94437
H	0.78492	5.25209	1.50316
H	1.74740	5.04614	2.97926
H	2.54132	4.91196	1.39089
C	-0.05134	2.88844	2.63603
H	-0.29318	1.81287	2.64273
H	-0.02445	3.23881	3.68349
H	-0.87123	3.42357	2.13196
C	0.42699	3.27290	-1.12288
C	0.59051	2.37851	-2.37559
H	0.18821	1.36424	-2.21110
H	0.03833	2.83830	-3.21476
H	1.64657	2.28970	-2.68199
C	-1.06621	3.37501	-0.74149
H	-1.23656	4.07996	0.08817
H	-1.62571	3.75697	-1.61387
H	-1.50707	2.39900	-0.47398
C	1.02358	4.66666	-1.40979
H	2.10428	4.61790	-1.61473
H	0.52851	5.07611	-2.30869
H	0.85395	5.37878	-0.58959
C	2.42012	-3.26081	1.63782
C	2.78521	-2.24994	2.75166
H	1.99896	-1.48890	2.89074
H	2.90967	-2.79890	3.70246
H	3.73613	-1.73225	2.54035
C	1.07765	-3.94826	1.97410
H	0.83652	-4.75889	1.26777
H	1.15456	-4.40156	2.97855
H	0.23337	-3.23773	1.99431
C	3.55456	-4.29703	1.49657
H	4.50702	-3.82595	1.20852
H	3.70258	-4.78385	2.47732
H	3.32138	-5.08774	0.76927
C	1.61885	-3.15225	-1.49878
C	1.89583	-2.18953	-2.67842
H	2.97417	-2.01931	-2.82911
H	1.49455	-2.64183	-3.60294
H	1.39919	-1.21386	-2.53308
C	2.37725	-4.47669	-1.71068
H	2.10243	-5.23868	-0.96419
H	2.10612	-4.88217	-2.70224
H	3.47039	-4.33923	-1.69503
C	0.09626	-3.39790	-1.38543
H	-0.25754	-3.83580	-2.33597
H	-0.16144	-4.10635	-0.58283
H	-0.46296	-2.46360	-1.21366
C	-0.29691	-0.35629	0.80144
H	-1.49806	-1.00005	2.31915
C	-1.50610	-0.62657	1.27952
C	-2.83413	-0.47132	0.64616
C	-2.98135	-0.26399	-0.74426
C	-3.98458	-0.54615	1.45827
H	-2.08258	-0.23293	-1.37074
H	-3.85966	-0.71142	2.53429
C	-4.25579	-0.13274	-1.32669
C	-5.27522	-0.41220	0.91253
C	-5.38279	-0.20605	-0.47872
H	-6.37641	-0.10905	-0.91777
C	-6.50862	-0.50001	1.83854
C	-6.42415	0.61966	2.91039
C	-6.52513	-1.88444	2.54029
H	-6.58999	-2.70226	1.80251
H	-5.62011	-2.04935	3.14907
H	-7.39758	-1.96033	3.21240
H	-5.51566	0.53012	3.52972
H	-7.29535	0.56657	3.58635
H	-6.41665	1.61826	2.44096
C	-4.38635	0.06102	-2.85511
C	-3.75930	-1.15967	-3.58033
C	-3.63582	1.35057	-3.28011
H	-4.06544	2.23876	-2.78569
H	-2.56361	1.30298	-3.02353
H	-3.71296	1.49649	-4.37188
H	-2.69030	-1.27820	-3.33383
H	-3.84017	-1.03796	-4.67479
H	-4.27591	-2.09357	-3.30105
C	-7.83650	-0.33236	1.06752
H	-7.90229	0.64868	0.56608
H	-7.97598	-1.12111	0.30825
H	-8.68263	-0.39988	1.77183
C	-5.85676	0.18909	-3.31136
H	-6.44222	-0.71594	-3.07559
H	-6.35849	1.05689	-2.84981
H	-5.89304	0.32940	-4.40491

TS(B-10)'$_{O-Ar}$

SCF (BP86) Energy = -1775.51764068
Enthalpy 0K = -1774.637854
Enthalpy 298K = -1774.582764
Free Energy 298K = -1774.721335
Lowest Frequency = -273.8383 cm^{-1}
Second Frequency = 20.4939 cm^{-1}
SCF (BP86-D3BJ) Energy = -1775.806318
SCF (FB) Energy = -1775.570888
SCF (DFB) Energy = -1775.57623
SCF (BS2) Energy = -2445.24517

Rh	1.09642	-0.12009	-0.23801
P	0.58051	-2.31206	0.14689
P	2.17319	1.93371	-0.55880
N	2.87467	-0.53715	0.71806
C	3.09217	-1.80595	1.21893
C	4.29622	-2.13037	1.87737
H	4.42320	-3.14233	2.26463
C	5.28608	-1.15321	2.00183
H	6.22527	-1.39202	2.50953
C	5.09077	0.12812	1.46750
H	5.84576	0.91324	1.52582
C	3.87613	0.38967	0.82422

O	2.15513	-2.74005	1.08219
O	3.68923	1.61540	0.27047
C	0.94614	-3.54703	-1.32136
C	2.23510	-3.02939	-1.99970
H	3.10405	-3.08070	-1.32512
H	2.45070	-3.66563	-2.87735
H	2.12465	-1.98779	-2.34501
C	1.18910	-4.97579	-0.78939
H	0.26175	-5.45139	-0.43192
H	1.56546	-5.59183	-1.62622
H	1.94248	-5.00190	0.01201
C	-0.19853	-3.57776	-2.35648
H	-0.32857	-2.60875	-2.86200
H	0.05619	-4.32856	-3.12722
H	-1.16372	-3.86713	-1.91268
C	-0.59050	-3.02107	1.52917
C	-0.80261	-1.87854	2.54735
H	-1.35680	-1.03451	2.11309
H	-1.39174	-2.26947	3.39700
H	0.15474	-1.50420	2.94682
C	-1.93962	-3.46989	0.92802
H	-1.81835	-4.31416	0.22942
H	-2.58896	-3.82070	1.75079
H	-2.46241	-2.65584	0.40481
C	0.05151	-4.21215	2.28021
H	1.00880	-3.94296	2.74683
H	-0.64946	-4.51007	3.08072
H	0.21002	-5.09201	1.64203
C	2.88065	2.36308	-2.29685
C	3.19282	0.99563	-2.94982
H	2.29579	0.35698	-3.01441
H	3.57527	1.16594	-3.97270
H	3.96640	0.44210	-2.39174
C	1.78910	3.09002	-3.11203
H	1.60701	4.11411	-2.74808
H	2.12097	3.17044	-4.16274
H	0.82935	2.54359	-3.11367
C	4.17542	3.20104	-2.24379
H	4.94290	2.72556	-1.61390
H	4.58160	3.28055	-3.26845
H	4.00807	4.22355	-1.87670
C	1.62044	3.46812	0.45349
C	1.54346	2.96703	1.91555
H	2.54285	2.74338	2.32142
H	1.09310	3.75804	2.54186
H	0.92389	2.05748	2.00753
C	2.59408	4.66005	0.36883
H	2.58128	5.14037	-0.62250
H	2.28169	5.42447	1.10353
H	3.62857	4.36909	0.61116
C	0.21372	3.88402	-0.03194
H	-0.16777	4.68343	0.62892
H	0.22213	4.28646	-1.05749
H	-0.50157	3.04541	0.00323
C	-0.60162	0.13667	-1.45589
H	-0.71377	0.61830	-2.42662
C	-0.76144	-1.07100	-0.85765
C	-2.01780	0.02821	-0.57248
C	-3.19685	-0.28105	-1.31645
C	-2.11114	0.78782	0.62606
H	-3.08907	-0.87549	-2.22753
H	-1.17660	1.01483	1.14783
C	-4.45146	0.15625	-0.88481
C	-3.36348	1.21624	1.10479
C	-4.49797	0.89089	0.32906
H	-5.47735	1.22616	0.68625
C	-5.75409	-0.13567	-1.65707
C	-6.71670	-0.94475	-0.74648
C	-5.49458	-0.95074	-2.94263
H	-4.83501	-0.41418	-3.64689
H	-5.04769	-1.93650	-2.72499
H	-6.44951	-1.13213	-3.46255
H	-6.27127	-1.91030	-0.45244
H	-7.65659	-1.15286	-1.28538
H	-6.97825	-0.39673	0.17422
C	-3.54158	2.01567	2.41333
C	-2.19790	2.25648	3.13294
C	-4.47379	1.22409	3.37002
H	-5.47238	1.05537	2.93363
H	-4.04364	0.24034	3.62371
H	-4.61401	1.78711	4.30847
H	-1.69932	1.31035	3.40691
H	-2.37444	2.81716	4.06576
H	-1.50232	2.85204	2.51768
C	-6.42062	1.20976	-2.05196
H	-5.76229	1.80457	-2.70779
H	-7.36070	1.01764	-2.59647
H	-6.66827	1.82532	-1.17090
C	-4.18127	3.39241	2.08899
H	-3.53914	3.98214	1.41275
H	-5.16985	3.28844	1.61134
H	-4.32151	3.97106	3.01794

TS(B-10)"$_{O-Ar}$

SCF (BP86) Energy = -1775.54863066
Enthalpy 0K = -1774.669963
Enthalpy 298K = -1774.614420
Free Energy 298K = -1774.756536
Lowest Frequency = -321.7255 cm^{-1}
Second Frequency = 9.5419 cm^{-1}
SCF (BP86-D3BJ) Energy = -1775.82065
SCF (FB) Energy = -1775.587193
SCF (DFB) Energy = -1775.592631
SCF (BS2) Energy = -2445.261953

Rh	1.17143	-0.15463	0.20058
P	0.60346	-2.33443	-0.32425
P	2.40320	1.77023	0.50352
N	2.94678	-0.72760	-0.76702
C	3.10296	-1.99556	-1.25883
C	4.27158	-2.41763	-1.91072
H	4.33385	-3.44208	-2.28060
C	5.31478	-1.49720	-2.06046
H	6.23750	-1.79706	-2.56545
C	5.18334	-0.19449	-1.56409
H	5.97311	0.55248	-1.65603
C	3.98543	0.15052	-0.92334
O	2.09110	-2.87538	-1.11322
O	3.84574	1.40666	-0.44227
C	-0.59998	-2.73148	-1.77759
C	-0.53307	-1.48274	-2.68995
H	0.48136	-1.32284	-3.09297
H	-1.21353	-1.62943	-3.54874
H	-0.84113	-0.56746	-2.15659
C	-0.18623	-3.97813	-2.58780
H	-0.25295	-4.90787	-2.00406
H	-0.87615	-4.07620	-3.44581
H	0.83611	-3.89069	-2.98488

C	-2.03256	-2.89867	-1.22877
H	-2.35204	-2.04293	-0.61088
H	-2.73090	-2.96568	-2.08279
H	-2.14766	-3.82465	-0.64166
C	0.56104	-3.63012	1.09187
C	1.87762	-3.42024	1.87709
H	1.99059	-2.37107	2.20281
H	1.86051	-4.06007	2.77785
H	2.76089	-3.69995	1.28098
C	-0.63225	-3.28244	2.01008
H	-1.60491	-3.42557	1.51266
H	-0.61082	-3.95041	2.89008
H	-0.57136	-2.24292	2.37437
C	0.47236	-5.08789	0.60113
H	1.27593	-5.33233	-0.11189
H	0.58036	-5.76292	1.46972
H	-0.49895	-5.31505	0.13296
C	1.91633	3.38823	-0.40905
C	1.27703	2.90054	-1.73216
H	0.45999	2.17899	-1.55805
H	0.86884	3.77073	-2.27755
H	2.02160	2.41543	-2.38518
C	0.86912	4.13048	0.44994
H	1.31585	4.57170	1.35643
H	0.44396	4.96208	-0.14052
H	0.03405	3.47648	0.75501
C	3.10544	4.31296	-0.74357
H	3.90412	3.77542	-1.27763
H	2.74190	5.11946	-1.40618
H	3.53883	4.79111	0.14616
C	3.25256	2.05360	2.21144
C	4.00649	0.73718	2.51638
H	4.85522	0.58090	1.83167
H	4.40866	0.78881	3.54423
H	3.33763	-0.13902	2.45381
C	4.24322	3.23463	2.21836
H	3.73223	4.20899	2.16152
H	4.80567	3.21797	3.16967
H	4.97488	3.16735	1.39771
C	2.15785	2.26280	3.27985
H	2.64091	2.38576	4.26614
H	1.54377	3.15953	3.09740
H	1.49384	1.38332	3.34449
C	-0.54939	0.43151	1.09211
C	-0.76679	0.86770	2.28144
H	-1.02464	1.25666	3.25607
C	-2.03485	0.58648	0.63485
C	-2.37754	1.60987	-0.27933
C	-3.03686	-0.25400	1.17603
H	-1.59057	2.26026	-0.66521
H	-2.74293	-1.01529	1.90151
C	-3.70716	1.76162	-0.70637
C	-4.38305	-0.11377	0.78058
C	-4.68196	0.89289	-0.15842
H	-5.72253	1.01670	-0.47543
C	-5.51448	-0.99394	1.35481
C	-4.98904	-2.03777	2.36437
C	-6.21951	-1.74642	0.19481
H	-6.65546	-1.05299	-0.54337
H	-5.51523	-2.40827	-0.33779
H	-7.03976	-2.36941	0.59090
H	-4.26807	-2.73431	1.90131
H	-5.83041	-2.64138	2.74317
H	-4.50624	-1.56421	3.23691
C	-4.13410	2.83770	-1.72742
C	-4.77158	2.14717	-2.96285
C	-2.94315	3.69387	-2.20963
H	-2.46534	4.24075	-1.37809
H	-2.17454	3.08390	-2.71566
H	-3.29835	4.44419	-2.93537
H	-4.04987	1.47756	-3.46125
H	-5.09479	2.90651	-3.69552
H	-5.65715	1.54886	-2.69148
C	-5.17625	3.78008	-1.06607
H	-6.07760	3.23652	-0.73793
H	-4.74983	4.29095	-0.18601
H	-5.49758	4.55150	-1.78690
C	-6.54366	-0.08725	2.08225
H	-6.99134	0.65683	1.40275
H	-7.36455	-0.70002	2.49281
H	-6.07355	0.45793	2.91842

TS(10-F)$_{O-Ar}$

SCF (BP86) Energy = -1775.51764068
Enthalpy 0K = -1774.637854
Enthalpy 298K = -1774.582764
Free Energy 298K = -1774.721335
Lowest Frequency = -273.8383 cm^{-1}
Second Frequency = 20.4939 cm^{-1}
SCF (BP86-D3BJ) Energy = -1775.802628
SCF (FB) Energy = -1775.556636
SCF (DFB) Energy = -1775.562191
SCF (BS2) Energy = -2445.232566

Rh	0.71125	0.17249	-0.22619
P	2.46611	-1.40007	-0.49800
P	-0.21641	2.31439	0.14977
N	2.15415	0.94812	1.09323
C	3.19419	0.16592	1.52875
C	4.12938	0.61535	2.47013
H	4.92579	-0.05701	2.79204
C	4.02404	1.93558	2.92734
H	4.74531	2.31660	3.65621
C	3.01935	2.77751	2.43891
H	2.92018	3.81796	2.75128
C	2.10451	2.25047	1.51327
O	3.33179	-1.07319	1.01133
O	1.14949	3.05005	1.01416
C	3.82178	-0.82385	-1.77690
C	4.07338	0.68172	-1.52651
H	4.55218	0.87755	-0.55340
H	4.76306	1.04722	-2.30839
H	3.14267	1.26860	-1.59403
C	5.14658	-1.59172	-1.58401
H	5.07571	-2.64306	-1.90266
H	5.91516	-1.11400	-2.21830
H	5.50568	-1.55514	-0.54311
C	3.30591	-0.99366	-3.22266
H	2.37416	-0.43421	-3.39476
H	4.08029	-0.59811	-3.90498
H	3.13162	-2.04451	-3.49606
C	2.41840	-3.33070	-0.34577
C	1.15462	-3.67280	0.46625
H	0.23460	-3.37860	-0.06086
H	1.12318	-4.76656	0.61962
H	1.16764	-3.20159	1.46419
C	2.30013	-3.96235	-1.75040

```
H   3.22618 -3.86361 -2.33760
H   2.10845 -5.04311 -1.62432
H   1.46135 -3.53883 -2.32670
C   3.65424 -3.88038  0.40257
H   3.73926 -3.46769  1.41880
H   3.52498 -4.97421  0.49192
H   4.59990 -3.70334 -0.12694
C  -0.29941  3.56307 -1.32451
C   0.65244  3.00665 -2.40814
H   0.38389  1.98378 -2.71753
H   0.60087  3.66655 -3.29339
H   1.69993  3.00077 -2.06263
C  -1.74369  3.59514 -1.86854
H  -2.44441  4.08022 -1.17055
H  -1.75355  4.18112 -2.80496
H  -2.12645  2.58926 -2.10624
C   0.15923  4.98413 -0.93098
H   1.18384  4.99106 -0.52990
H   0.14954  5.60652 -1.84417
H  -0.50493  5.46375 -0.19831
C  -1.50748  2.67459  1.55066
C  -1.00454  1.87550  2.77704
H  -0.05367  2.26624  3.17348
H  -1.75688  1.96694  3.58107
H  -0.88778  0.80254  2.54965
C  -1.57163  4.17670  1.89820
H  -2.06557  4.76628  1.10941
H  -2.17888  4.29178  2.81457
H  -0.57842  4.60689  2.09996
C  -2.90834  2.16112  1.16358
H  -3.59794  2.40274  1.99318
H  -3.30662  2.64279  0.25780
H  -2.92914  1.07214  1.01700
C   0.03266 -0.71155 -1.85714
H  -0.18504 -1.65792 -3.95863
C  -0.03627 -1.23579 -2.98109
C  -1.30017 -0.68951 -0.44437
C  -2.43176 -0.27380 -1.18026
C  -1.46680 -1.51540  0.68336
H  -2.28878  0.32471 -2.08289
H  -0.59912 -1.83966  1.26016
C  -3.72263 -0.72056 -0.83556
C  -2.76300 -1.90370  1.10943
C  -3.86356 -1.51524  0.32310
H  -4.85991 -1.85004  0.61274
C  -4.92065 -0.39306 -1.75960
C  -4.69139 -1.09534 -3.12567
C  -5.03742  1.13518 -1.98668
H  -5.21465  1.66846 -1.03710
H  -4.13224  1.55578 -2.45621
H  -5.88440  1.35384 -2.65967
H  -3.76617 -0.74524 -3.61435
H  -5.53357 -0.88712 -3.80852
H  -4.61489 -2.18873 -3.00037
C  -2.92401 -2.76772  2.38251
C  -2.21967 -2.07998  3.58134
C  -2.28303 -4.16007  2.14116
H  -2.76055 -4.67457  1.29015
H  -1.20393 -4.08154  1.92902
H  -2.40273 -4.79563  3.03588
H  -1.13926 -1.93986  3.40537
H  -2.32803 -2.69699  4.49007
H  -2.66291 -1.09088  3.78832
C  -6.26203 -0.89239 -1.17614
H  -6.28006 -1.98917 -1.05820
H  -6.48398 -0.43295 -0.19739
H  -7.08298 -0.62429 -1.86204
C  -4.40839 -2.97851  2.75920
H  -4.93048 -2.02144  2.93051
H  -4.95747 -3.54064  1.98479
H  -4.47064 -3.56351  3.69209
```

$F_{O\text{-}Ar}$

SCF (BP86) Energy = -1775.55986685
Enthalpy 0K = -1774.679157
Enthalpy 298K = -1774.623400
Free Energy 298K = -1774.763408
Lowest Frequency = 18.9493 cm^{-1}
Second Frequency = 24.6728 cm^{-1}
SCF (BP86-D3BJ) Energy = -1775.850476
SCF (FB) Energy = -1775.600138
SCF (DFB) Energy = -1775.606079
SCF (BS2) Energy = -2445.274716

```
Rh  0.94821  0.02381 -0.51587
P   1.29335 -2.29675 -0.19559
P   1.08444  2.36746 -0.24267
N   1.47482  0.07588  1.52212
C   1.77370 -1.08146  2.18651
C   2.14716 -1.08455  3.53897
H   2.37145 -2.03151  4.03203
C   2.21913  0.14611  4.20426
H   2.50715  0.17321  5.25921
C   1.93960  1.34432  3.53298
H   2.00134  2.31797  4.02121
C   1.57411  1.27103  2.18138
O   1.70518 -2.26021  1.52469
O   1.30086  2.41617  1.51106
C   2.97161 -2.90565 -0.92922
C   3.87184 -1.65419 -1.01234
H   4.03155 -1.18038 -0.02851
H   4.86452 -1.95186 -1.39648
H   3.45416 -0.91675 -1.71751
C   3.67053 -3.96427 -0.05025
H   3.13759 -4.92333 -0.01744
H   4.66952 -4.15708 -0.48159
H   3.81506 -3.60925  0.98225
C   2.71460 -3.41905 -2.36461
H   2.18270 -2.67104 -2.97810
H   3.69182 -3.60748 -2.84495
H   2.15430 -4.36647 -2.38623
C  -0.03687 -3.69303 -0.19858
C  -1.03400 -3.41626  0.94737
H  -1.52555 -2.43843  0.85371
H  -1.81669 -4.19518  0.90677
H  -0.54867 -3.48012  1.93432
C  -0.75472 -3.59390 -1.56515
H  -0.08240 -3.78402 -2.41664
H  -1.54901 -4.36136 -1.59398
H  -1.22922 -2.61022 -1.70545
C   0.55563 -5.10334  0.00568
H   1.10398 -5.19323  0.95703
H  -0.28799 -5.81604  0.04484
H   1.20863 -5.42403 -0.81975
C   2.79615  3.06104 -0.80434
C   3.79058  1.89386 -0.62190
H   3.51502  1.03066 -1.24762
```

H 4.79414 2.22463 -0.94572
H 3.87567 1.56960 0.42921
C 2.70043 3.40951 -2.30685
H 2.07571 4.29677 -2.49511
H 3.71528 3.64064 -2.67808
H 2.31037 2.56531 -2.90214
C 3.28677 4.25738 0.03848
H 3.27055 4.03447 1.11715
H 4.33516 4.46352 -0.24402
H 2.71513 5.17758 -0.13710
C -0.33242 3.65197 -0.47420
C -1.43758 3.36652 0.56567
H -1.07314 3.49859 1.59679
H -2.24925 4.09752 0.39896
H -1.86312 2.35943 0.46510
C 0.13301 5.11327 -0.30450
H 0.84649 5.43116 -1.07966
H -0.75787 5.75957 -0.40237
H 0.56825 5.30352 0.68945
C -0.87382 3.41831 -1.90473
H -1.71502 4.11468 -2.07205
H -0.12129 3.61668 -2.68437
H -1.25029 2.39229 -2.03615
C 0.84327 -0.00294 -2.48217
H 1.00355 -0.02055 -4.78915
C 0.93239 -0.01382 -3.71595
C -1.08218 -0.04159 -0.18030
C -1.97065 -0.09947 -1.26450
C -1.57241 -0.02248 1.14230
H -1.58359 -0.11738 -2.28424
H -0.88985 0.01826 1.98989
C -3.37324 -0.13429 -1.04262
C -2.96294 -0.05673 1.38980
C -3.83806 -0.11055 0.28237
H -4.91455 -0.13598 0.46814
C -4.32267 -0.19800 -2.26200
C -4.02271 -1.47991 -3.08329
C -4.09670 1.04767 -3.15903
H -4.30717 1.97874 -2.60543
H -3.06206 1.09983 -3.53817
H -4.76950 1.01299 -4.03344
H -2.98580 -1.49457 -3.45959
H -4.69434 -1.53643 -3.95745
H -4.18039 -2.38693 -2.47486
C -3.54173 -0.03680 2.82374
C -4.44715 1.21313 2.99104
C -2.44330 0.01385 3.90772
H -1.77931 -0.86744 3.86510
H -1.82264 0.92349 3.82603
H -2.91056 0.02444 4.90666
H -3.87129 2.14359 2.84793
H -4.88240 1.23415 4.00528
H -5.28073 1.21738 2.26972
C -5.80917 -0.22895 -1.84102
H -6.04611 -1.11397 -1.22575
H -6.09816 0.67494 -1.27760
H -6.44505 -0.27358 -2.74098
C -4.38838 -1.31816 3.05087
H -5.22077 -1.39501 2.33230
H -3.77006 -2.22672 2.95049
H -4.82249 -1.31173 4.06577

9_{NH}
SCF (BP86) Energy = -2537.88846550
Enthalpy 0K = -2536.549203
Enthalpy 298K = -2536.467549
Free Energy 298K = -2536.660105
Lowest Frequency = 14.1004 cm^{-1}
Second Frequency = 18.1482 cm^{-1}
SCF (BP86-D3BJ) Energy = -2538.34435
SCF (FB) Energy = -2538.015502
SCF (DFB) Energy = -2538.033086
SCF (BS2) Energy = -3877.0767

Rh 2.74235 -2.03509 1.21206
P 4.02904 -2.80345 -0.60374
P 1.95707 -1.69022 3.46091
N 4.08694 -3.33041 2.26012
C 1.20450 -1.40063 -0.23171
H 1.57347 -1.77281 -1.19777
C 1.86610 -0.25067 0.27109
H 2.71371 0.12487 -0.31939
C 1.16736 0.87470 1.01784
H 1.92460 1.52779 1.48388
H 0.53887 0.48444 1.83567
C -0.26527 -1.73365 -0.05213
H -0.59066 -1.57649 0.98820
H -0.41984 -2.80658 -0.26402
C 5.17547 -3.88319 1.62906
C 6.08412 -4.73357 2.28750
H 6.93774 -5.14670 1.74523
C 5.86080 -5.03231 3.63417
H 6.55018 -5.69294 4.16806
C 4.75957 -4.48933 4.29945
H 4.56305 -4.70945 5.35133
C 3.89315 -3.63871 3.58364
N 5.33860 -3.55374 0.30176
N 2.78961 -3.07724 4.18039
C 3.33037 -4.32677 -1.61405
C 2.32859 -5.01520 -0.65774
H 2.81764 -5.36659 0.26617
H 1.89772 -5.89918 -1.16294
H 1.50595 -4.34267 -0.36437
C 4.44095 -5.33824 -1.97719
H 5.22661 -4.91303 -2.62092
H 3.98234 -6.17295 -2.53731
H 4.90989 -5.77905 -1.08246
C 2.60803 -3.87917 -2.90239
H 1.84034 -3.10778 -2.72261
H 2.09115 -4.75318 -3.33870
H 3.30836 -3.50424 -3.66585
C 5.05883 -1.58494 -1.70127
C 5.73404 -0.61408 -0.70384
H 4.99728 -0.09445 -0.06845
H 6.29811 0.14747 -1.27179
H 6.44223 -1.13655 -0.04140
C 4.13150 -0.79387 -2.65081
H 3.68251 -1.42749 -3.43014
H 4.73200 -0.02030 -3.16241
H 3.31970 -0.27460 -2.11670
C 6.14182 -2.30238 -2.53561
H 6.84067 -2.89256 -1.91911
H 6.75188 -1.53847 -3.05101
H 5.71574 -2.95448 -3.31440
C 0.18483 -2.11955 4.14923
C -0.20237 -3.45108 3.46408
H -0.18965 -3.37035 2.36496
H -1.22454 -3.73278 3.77571

H	0.47759	-4.26831	3.75153
C	-0.84342	-1.02782	3.78592
H	-0.64685	-0.07273	4.29559
H	-1.84497	-1.37074	4.10271
H	-0.89363	-0.83766	2.70227
C	0.16951	-2.30947	5.68276
H	0.84933	-3.10518	6.03046
H	-0.84614	-2.62389	5.98485
H	0.39608	-1.38142	6.23101
C	2.78710	-0.21495	4.46593
C	3.99468	0.23338	3.60989
H	4.71920	-0.58476	3.46143
H	4.52108	1.05542	4.12937
H	3.68839	0.59181	2.61401
C	3.31251	-0.67927	5.84337
H	2.52219	-1.07140	6.50345
H	3.75465	0.19274	6.35812
H	4.11122	-1.43317	5.75345
C	1.81729	0.96739	4.67126
H	2.38497	1.82184	5.08265
H	1.02160	0.73181	5.39658
H	1.35044	1.30880	3.73451
C	-1.20450	1.40063	0.23171
H	-1.57347	1.77281	1.19777
C	-1.86610	0.25067	-0.27109
H	-2.71371	-0.12487	0.31939
C	-1.16736	-0.87470	-1.01784
H	-1.92460	-1.52779	-1.48388
H	-0.53887	-0.48444	-1.83567
C	0.26527	1.73365	0.05213
H	0.59066	1.57649	-0.98820
H	0.41984	2.80658	0.26402
Rh	-2.74235	2.03509	-1.21206
P	-4.02904	2.80345	0.60374
P	-1.95707	1.69022	-3.46091
N	-4.08694	3.33041	-2.26012
C	-5.17547	3.88319	-1.62906
C	-6.08412	4.73357	-2.28750
H	-6.93774	5.14670	-1.74523
C	-5.86080	5.03231	-3.63417
H	-6.55018	5.69294	-4.16806
C	-4.75957	4.48933	-4.29945
H	-4.56305	4.70945	-5.35133
C	-3.89315	3.63871	-3.58364
N	-5.33860	3.55374	-0.30176
N	-2.78961	3.07724	-4.18039
C	-3.33037	4.32677	1.61405
C	-2.32859	5.01520	0.65774
H	-2.81764	5.36659	-0.26617
H	-1.89772	5.89918	1.16294
H	-1.50595	4.34267	0.36437
C	-4.44095	5.33824	1.97719
H	-5.22661	4.91303	2.62092
H	-3.98234	6.17295	2.53731
H	-4.90989	5.77905	1.08246
C	-2.60803	3.87917	2.90239
H	-1.84034	3.10778	2.72261
H	-2.09115	4.75318	3.33870
H	-3.30836	3.50424	3.66585
C	-5.05883	1.58494	1.70127
C	-5.73404	0.61408	0.70384
H	-4.99728	0.09445	0.06845
H	-6.29811	-0.14747	1.27179
H	-6.44223	1.13655	0.04140
C	-4.13150	0.79387	2.65081
H	-3.68251	1.42749	3.43014
H	-4.73200	0.02030	3.16241
H	-3.31970	0.27460	2.11670
C	-6.14182	2.30238	2.53561
H	-6.84067	2.89256	1.91911
H	-6.75188	1.53847	3.05101
H	-5.71574	2.95448	3.31440
C	-0.18483	2.11955	-4.14923
C	0.20237	3.45108	-3.46408
H	0.18965	3.37035	-2.36496
H	1.22454	3.73278	-3.77571
H	-0.47759	4.26831	-3.75153
C	0.84342	1.02782	-3.78592
H	0.64685	0.07273	-4.29559
H	1.84497	1.37074	-4.10271
H	0.89363	0.83766	-2.70227
C	-0.16951	2.30947	-5.68276
H	-0.84933	3.10518	-6.03046
H	0.84614	2.62389	-5.98485
H	-0.39608	1.38142	-6.23101
C	-2.78710	0.21495	-4.46593
C	-3.99468	-0.23338	-3.60989
H	-4.71920	0.58476	-3.46143
H	-4.52108	-1.05542	-4.12937
H	-3.68839	-0.59181	-2.61401
C	-3.31251	0.67927	-5.84337
H	-2.52219	1.07140	-6.50345
H	-3.75465	-0.19274	-6.35812
H	-4.11122	1.43317	-5.75345
C	-1.81729	-0.96739	-4.67126
H	-2.38497	-1.82184	-5.08265
H	-1.02160	-0.73181	-5.39658
H	-1.35044	-1.30880	-3.73451
H	-6.07668	4.05577	0.19220
H	-2.74029	3.20078	-5.19217
H	6.07668	-4.05577	-0.19220
H	2.74029	-3.20078	5.19217

8_{NH}

SCF (BP86) Energy = -1424.98194552
Enthalpy 0K = -1424.224952
Enthalpy 298K = -1424.179849
Free Energy 298K = -1424.296869
Lowest Frequency = 18.7555 cm^{-1}
Second Frequency = 29.7453 cm^{-1}
SCF (BP86-D3BJ) Energy = -1425.222455
SCF (FB) Energy = -1425.024157
SCF (DFB) Energy = -1425.03031
SCF (BS2) Energy = -2094.613717

C	2.67483	4.48821	0.41572
H	3.68700	4.83002	0.66857
C	2.27335	4.63403	-0.86426
H	2.98567	5.08543	-1.56707
C	0.93717	4.22438	-1.45352
H	0.84271	4.66100	-2.46185
H	0.10790	4.65320	-0.85611
C	1.88265	3.87336	1.55377
H	0.87291	4.32615	1.60701
H	2.37685	4.11772	2.50899
C	-0.09168	2.14309	-0.39994
H	-1.14523	2.44023	-0.50145

C 0.35278 1.93675 0.92862
H -0.40725 2.03380 1.71712
C 1.73256 2.31393 1.43793
H 1.88849 1.85525 2.42975
H 2.52643 1.93378 0.77570
C 0.73529 2.66982 -1.55442
H 1.71896 2.17999 -1.60307
H 0.21702 2.42848 -2.50040
Rh -0.15855 -0.01488 0.05203
P -2.48984 0.16230 0.10052
P 2.06257 -0.93081 -0.04772
N -0.65409 -2.08080 -0.16066
C -1.94660 -2.51588 0.00310
C -2.31030 -3.87130 -0.11929
H -3.35032 -4.17364 0.02367
C -1.31426 -4.80194 -0.42824
H -1.57092 -5.86049 -0.53139
C 0.00664 -4.38565 -0.60574
H 0.80118 -5.09473 -0.84967
C 0.30530 -3.01424 -0.46174
N -2.87971 -1.54732 0.30264
N 1.58261 -2.53826 -0.62977
C -3.40028 0.56642 -1.58380
C -2.39373 0.17141 -2.68942
H -2.12495 -0.89706 -2.63794
H -2.85485 0.35372 -3.67779
H -1.46016 0.75321 -2.62611
C -4.68406 -0.27521 -1.76367
H -5.43648 -0.09729 -0.97945
H -5.15028 0.00134 -2.72658
H -4.46849 -1.35511 -1.81529
C -3.75353 2.06410 -1.70461
H -2.89098 2.72857 -1.53161
H -4.10928 2.26050 -2.73247
H -4.56489 2.35978 -1.02029
C -3.41597 0.89379 1.63666
C -2.72247 0.25808 2.86479
H -1.63850 0.46064 2.87704
H -3.16341 0.68293 3.78456
H -2.86224 -0.83442 2.89256
C -3.25564 2.43059 1.68685
H -3.79333 2.94175 0.87461
H -3.67879 2.79633 2.63970
H -2.20223 2.75105 1.65773
C -4.91859 0.54062 1.65377
H -5.10554 -0.54614 1.61378
H -5.35354 0.89816 2.60488
H -5.47764 1.02852 0.83959
C 3.40274 -0.58081 -1.42026
C 2.61057 -0.50970 -2.74698
H 1.82438 0.26168 -2.71872
H 3.30690 -0.25786 -3.56731
H 2.13330 -1.47290 -2.98806
C 4.13921 0.75443 -1.17579
H 4.76535 0.73505 -0.27142
H 4.81045 0.94362 -2.03304
H 3.46214 1.61936 -1.10395
C 4.45754 -1.70589 -1.51722
H 4.02155 -2.69641 -1.73204
H 5.13167 -1.47799 -2.36297
H 5.08594 -1.77960 -0.61570
C 2.90057 -1.42139 1.66677
C 1.76929 -1.32102 2.71641
H 0.92857 -1.99498 2.48021
H 2.16707 -1.61396 3.70595
H 1.36533 -0.29940 2.79453
C 3.42617 -2.87504 1.65837
H 4.19888 -3.05552 0.89372
H 3.89302 -3.08031 2.63875
H 2.61630 -3.61167 1.53043
C 4.05744 -0.47267 2.04365
H 4.36706 -0.68989 3.08238
H 4.94363 -0.62294 1.40622
H 3.77444 0.58979 2.00469
H -3.85278 -1.85329 0.29622
H 2.30091 -3.25714 -0.72054

A_{NH}

SCF (BP86) Energy = -1112.91730227
Enthalpy 0K = -1112.338468
Enthalpy 298K = -1112.301753
Free Energy 298K = -1112.403195
Lowest Frequency = 15.6067 cm^{-1}
Second Frequency = 29.5735 cm^{-1}
SCF (BP86-D3BJ) Energy = -1113.092091
SCF (FB) Energy = -1112.968017
SCF (DFB) Energy = -1112.976433
SCF (BS2) Energy = -1782.479111

Rh 0.00003 -0.48544 0.08256
P 2.29812 -0.26780 0.03846
P -2.29808 -0.26787 0.03837
N 0.00004 1.51514 -0.06516
C 1.18707 2.22164 -0.12610
C 1.20971 3.62334 -0.25002
H 2.16967 4.14313 -0.29568
C 0.00000 4.31855 -0.31122
H -0.00001 5.40784 -0.40846
C -1.20968 3.62330 -0.25027
H -2.16966 4.14304 -0.29613
C -1.18701 2.22158 -0.12645
N 2.35172 1.48700 -0.05253
N -2.35165 1.48690 -0.05329
C 3.15615 -0.87912 -1.57649
C 2.49372 -0.08636 -2.72632
H 2.76479 0.98162 -2.70493
H 2.83499 -0.50311 -3.69090
H 1.39281 -0.16414 -2.68817
C 4.68351 -0.68222 -1.61510
H 5.20911 -1.30303 -0.87208
H 5.05856 -0.98023 -2.61117
H 4.97710 0.37083 -1.46380
C 2.79464 -2.37658 -1.72469
H 1.69955 -2.52605 -1.73015
H 3.18322 -2.74932 -2.68954
H 3.22931 -3.00460 -0.93096
C 3.31051 -0.62986 1.63767
C 2.37958 -0.21667 2.80200
H 1.43425 -0.78575 2.79183
H 2.89321 -0.41186 3.76095
H 2.12924 0.85668 2.76589
C 3.59986 -2.14434 1.71778
H 4.32933 -2.47678 0.96143
H 4.02880 -2.37684 2.70889
H 2.68263 -2.75027 1.61041
C 4.61989 0.18230 1.72416
H 4.43124 1.26937 1.71117

H   5.11168 -0.04042 2.68841
H   5.33740 -0.06404 0.92758
C -3.15571 -0.87972 -1.57656
C -2.49300 -0.08739 -2.72654
H -1.39210 -0.16522 -2.68810
H -2.83409 -0.50448 -3.69103
H -2.76400 0.98061 -2.70555
C -2.79423 -2.37724 -1.72414
H -3.22908 -3.00497 -0.93027
H -3.18261 -2.75032 -2.68894
H -1.69914 -2.52677 -1.72933
C -4.68307 -0.68275 -1.61562
H -4.97661 0.37037 -1.46477
H -5.05789 -0.98109 -2.61168
H -5.20890 -1.30327 -0.87252
C -3.31100 -0.62929 1.63739
C -2.38033 -0.21588 2.80184
H -2.12972 0.85740 2.76541
H -2.89426 -0.41055 3.76074
H -1.43512 -0.78517 2.79221
C -4.62028 0.18311 1.72331
H -5.33765 -0.06334 0.92663
H -5.11232 -0.03924 2.68751
H -4.43148 1.27015 1.71006
C -3.60064 -2.14369 1.71792
H -4.02989 -2.37579 2.70898
H -4.32995 -2.47623 0.96147
H -2.68350 -2.74981 1.61099
H   3.22378 2.01109 -0.11574
H -3.22373 2.01096 -0.11653

B_{NH-Ar}
SCF (BP86) Energy = -1735.87141024
Enthalpy 0K = -1734.965595
Enthalpy 298K = -1734.909682
Free Energy 298K = -1735.051739
Lowest Frequency = 11.6033 cm^{-1}
Second Frequency = 16.5167 cm^{-1}
SCF (BP86-D3BJ) Energy = -1736.152509
SCF (FB) Energy = -1735.912782
SCF (DFB) Energy = -1735.91896
SCF (BS2) Energy = -2405.573751

Rh -1.25596 0.14033 -0.30468
P -0.82791 2.42482 0.10204
P -2.22766 -1.98338 -0.47013
N -2.99693 0.54752 0.81448
C -3.25014 1.81435 1.28730
C -4.40632 2.11661 2.03563
H -4.57280 3.13366 2.39807
C -5.32060 1.09220 2.29225
H -6.22344 1.30311 2.87324
C -5.09486 -0.19850 1.80580
H -5.80455 -1.00886 1.98777
C -3.92479 -0.43882 1.05862
N -2.31492 2.77886 0.99353
N -3.65644 -1.67873 0.52413
C -0.98706 3.59460 -1.43046
C -2.28923 3.15543 -2.13988
H -3.18139 3.34755 -1.52157
H -2.39614 3.73078 -3.07737
H -2.26987 2.08157 -2.39232
C -1.07730 5.09033 -1.06768
H -0.13097 5.48344 -0.66383
H -1.30078 5.66447 -1.98542
H -1.88777 5.30910 -0.35095
C   0.21510 3.34802 -2.36903
H   0.32774 2.27990 -2.61691
H   0.04871 3.90039 -3.31168
H   1.16490 3.70525 -1.94230
C   0.48676 3.03161 1.39054
C   0.53605 1.91060 2.45382
H   0.87849 0.95770 2.02176
H   1.24557 2.20266 3.24964
H -0.44787 1.74918 2.92674
C   1.86356 3.17596 0.70970
H   1.90552 4.05191 0.04201
H   2.63196 3.32538 1.48968
H   2.14882 2.27779 0.13749
C   0.09732 4.35426 2.08680
H -0.83968 4.26347 2.66223
H   0.88771 4.60653 2.81690
H   0.01169 5.20574 1.39574
C -3.04742 -2.53834 -2.13545
C -3.55381 -1.23241 -2.79179
H -2.73559 -0.52458 -2.99703
H -4.04691 -1.47951 -3.74968
H -4.29482 -0.71768 -2.15714
C -1.98780 -3.21025 -3.03527
H -1.71740 -4.21678 -2.67719
H -2.40162 -3.32732 -4.05321
H -1.06398 -2.61343 -3.11823
C -4.25388 -3.48205 -1.93788
H -5.06202 -3.00531 -1.35772
H -4.67776 -3.72015 -2.93036
H -3.98813 -4.43680 -1.46112
C -1.38781 -3.43801 0.48336
C -1.15034 -2.89099 1.91049
H -2.09547 -2.73260 2.45490
H -0.54986 -3.62333 2.47955
H -0.60171 -1.93321 1.89383
C -2.24433 -4.71800 0.55961
H -2.34347 -5.21653 -0.41759
H -1.74863 -5.43466 1.23972
H -3.25328 -4.53754 0.96945
C -0.03013 -3.75131 -0.18392
H   0.50080 -4.50090 0.43060
H -0.14269 -4.17815 -1.19241
H   0.61358 -2.86086 -0.25529
C -0.09353 -0.13969 -2.01709
H -0.39413 -0.23921 -3.05506
C   0.75719 -0.29098 -1.07698
C   2.09495 -0.42238 -0.54883
C   3.18120 -0.08538 -1.39943
C   2.35421 -0.89894 0.75825
H   2.95817 0.28116 -2.40414
H   1.50432 -1.16213 1.39208
C   4.50910 -0.22536 -0.96433
C   3.67315 -1.05010 1.22509
C   4.72240 -0.70542 0.34727
H   5.75362 -0.81854 0.69813
C   5.71748 0.11905 -1.86218
C   6.56680 1.22402 -1.17911
C   5.28605 0.62961 -3.25455
H   4.70332 -0.12623 -3.80924
H   4.68505 1.55339 -3.18777
H   6.18060 0.86189 -3.85622

H 5.97834 2.14662 -1.03620
H 7.44207 1.47110 -1.80456
H 6.94222 0.90623 -0.19215
C 4.00351 -1.57774 2.63858
C 2.73379 -1.90211 3.45540
C 4.81766 -0.50541 3.41077
H 5.76278 -0.25792 2.89975
H 4.23985 0.42823 3.52195
H 5.07079 -0.87297 4.42039
H 2.09925 -1.01084 3.60457
H 3.02023 -2.27340 4.45366
H 2.12439 -2.68649 2.97364
C 4.84729 -2.87502 2.51678
H 4.29228 -3.66100 1.97660
H 5.79394 -2.70178 1.97883
H 5.09918 -3.26113 3.51981
C 6.58589 -1.15284 -2.05696
H 6.00966 -1.95606 -2.54701
H 7.45944 -0.92260 -2.69121
H 6.96422 -1.54510 -1.09830
H -4.35231 -2.40468 0.69150
H -2.49493 3.71028 1.36669

10_{NH-Ar}
SCF (BP86) Energy = -1735.87890945
Enthalpy 0K = -1734.973041
Enthalpy 298K = -1734.917054
Free Energy 298K = -1735.062180
Lowest Frequency = 7.5262 cm^{-1}
Second Frequency = 10.9294 cm^{-1}
SCF (BP86-D3BJ) Energy = -1736.15106
SCF (FB) Energy = -1735.921915
SCF (DFB) Energy = -1735.928453
SCF (BS2) Energy = -2405.581169

Rh -1.48352 -0.01304 -0.24774
P -1.60322 2.32208 -0.30479
P -1.91757 -2.29626 -0.04284
N -3.51424 0.15991 0.34436
C -4.09025 1.39785 0.49735
C -5.43660 1.54262 0.88733
H -5.87398 2.53698 1.00231
C -6.18611 0.38527 1.12155
H -7.23314 0.47361 1.42698
C -5.61721 -0.88351 0.96973
H -6.19652 -1.79219 1.14855
C -4.26782 -0.96600 0.57293
N -3.27793 2.48626 0.25775
N -3.63507 -2.17691 0.38216
C -1.65790 3.13604 -2.05366
C -2.72784 2.33584 -2.83304
H -3.73854 2.47443 -2.41548
H -2.74326 2.68990 -3.87951
H -2.49901 1.25578 -2.84013
C -2.03317 4.63098 -2.03227
H -1.24303 5.25599 -1.58676
H -2.17259 4.97756 -3.07238
H -2.98116 4.82721 -1.50168
C -0.28359 2.93468 -2.73044
H 0.02134 1.87594 -2.72784
H -0.35590 3.26780 -3.78157
H 0.51562 3.52264 -2.25374
C -0.65450 3.34393 1.02478
C -0.65334 2.44450 2.28317
H -0.15460 1.47952 2.09196
H -0.11088 2.96279 3.09456
H -1.67645 2.23881 2.64138
C 0.79370 3.57654 0.54495
H 0.84728 4.30435 -0.28080
H 1.38331 3.99062 1.38236
H 1.28754 2.64280 0.22637
C -1.33556 4.68606 1.36749
H -2.35579 4.54514 1.76327
H -0.75338 5.18060 2.16586
H -1.37614 5.38202 0.51650
C -1.98680 -3.38970 -1.62844
C -2.46504 -2.43739 -2.74955
H -1.76612 -1.59744 -2.89625
H -2.53408 -3.00383 -3.69611
H -3.46366 -2.02061 -2.53450
C -0.56699 -3.90428 -1.94926
H -0.24079 -4.69161 -1.25056
H -0.56855 -4.34716 -2.96152
H 0.18209 -3.09443 -1.93946
C -2.97812 -4.56709 -1.51072
H -4.01352 -4.22117 -1.35156
H -2.97458 -5.12600 -2.46387
H -2.71326 -5.27979 -0.71522
C -1.15153 -3.17401 1.49363
C -1.51356 -2.26703 2.69309
H -2.59864 -2.24252 2.88564
H -1.01842 -2.66102 3.59871
H -1.16471 -1.23135 2.53524
C -1.69450 -4.59741 1.72931
H -1.37499 -5.30374 0.94663
H -1.29767 -4.97546 2.68896
H -2.79545 -4.62834 1.80602
C 0.38445 -3.20360 1.32779
H 0.83197 -3.58117 2.26477
H 0.71345 -3.87036 0.51632
H 0.79830 -2.20047 1.13644
C 0.25362 -0.18173 -0.82612
H 1.49077 -0.55600 -2.40374
C 1.47842 -0.32507 -1.32340
C 2.79829 -0.21283 -0.66642
C 3.96031 -0.34179 -1.45551
C 2.93209 0.01079 0.72310
H 3.84790 -0.51527 -2.53179
H 2.02521 0.10541 1.33151
C 5.24582 -0.25350 -0.88884
C 4.20011 0.10168 1.32597
C 5.33853 -0.03237 0.50089
H 6.32744 0.03474 0.95582
C 6.49023 -0.40151 -1.79236
C 6.47096 0.70926 -2.87644
C 6.46205 -1.79266 -2.48027
H 6.48036 -2.60456 -1.73327
H 5.56149 -1.92667 -3.10308
H 7.34212 -1.91159 -3.13614
H 5.56973 0.65141 -3.50994
H 7.35033 0.61284 -3.53694
H 6.49721 1.71208 -2.41684
C 4.31113 0.33624 2.85026
C 3.60061 -0.82037 3.60243
C 3.62827 1.68108 3.21477
H 4.11836 2.52496 2.69962
H 2.56096 1.68592 2.93468
H 3.69128 1.86109 4.30247

H 2.53189 -0.88724 3.33617
H 3.66551 -0.66480 4.69373
H 4.06937 -1.79106 3.36715
C 7.81062 -0.28093 -0.99994
H 7.90922 0.70245 -0.50863
H 7.90284 -1.06539 -0.22907
H 8.66535 -0.39283 -1.68816
C 5.77695 0.39507 3.33464
H 6.31584 -0.54656 3.13336
H 6.33508 1.22165 2.86249
H 5.79930 0.56189 4.42490
H -4.19016 -3.01081 0.57267
H -3.71300 3.40332 0.35547

9_S

SCF (BP86) Energy = -2357.31258953
Enthalpy 0K = -2356.032388
Enthalpy 298K = -2355.950099
Free Energy 298K = -2356.143423
Lowest Frequency = -6.4659 cm^{-1}
Second Frequency = 10.9800 cm^{-1}
SCF (BP86-D3BJ) Energy = -2357.800649
SCF (FB) Energy = -2357.436878
SCF (DFB) Energy = -2357.453655
SCF (BS2) Energy = -5247.449346

Rh 2.71433 -2.03695 1.20413
P 4.16181 -2.57080 -0.61338
P 1.82734 -1.65767 3.42551
N 3.92203 -3.61426 2.30150
C 1.07305 -1.52024 -0.13318
H 1.32579 -2.06208 -1.05219
C 1.84513 -0.34917 0.11439
H 2.59171 -0.11662 -0.65459
C 1.33105 0.92736 0.75935
H 2.19021 1.56834 1.02579
H 0.79209 0.72307 1.69703
C -0.38492 -1.71358 0.23077
H -0.58566 -1.37318 1.25794
H -0.62713 -2.79057 0.20098
C 5.13761 -4.05441 1.83943
C 5.86897 -5.09680 2.43681
H 6.81383 -5.41573 1.99075
C 5.36422 -5.70959 3.58776
H 5.91309 -6.52078 4.07492
C 4.15356 -5.25278 4.11037
H 3.73647 -5.67038 5.02974
C 3.46832 -4.21466 3.44855
S 5.92161 -3.27858 0.45307
S 1.95341 -3.70450 4.19566
C 3.69859 -4.11675 -1.73916
C 3.33608 -5.25560 -0.76021
H 4.21518 -5.62650 -0.20966
H 2.93216 -6.10303 -1.34312
H 2.56548 -4.94530 -0.03324
C 4.85777 -4.56259 -2.65121
H 5.02724 -3.86570 -3.48740
H 4.59282 -5.53942 -3.09520
H 5.80141 -4.69946 -2.09859
C 2.45465 -3.79860 -2.60142
H 1.54314 -3.70386 -1.99048
H 2.28929 -4.64839 -3.28835
H 2.56769 -2.89676 -3.22329
C 5.11129 -1.17154 -1.61738
C 5.35221 -0.00231 -0.63403
H 4.42243 0.43405 -0.24013
H 5.90135 0.79464 -1.16744
H 5.97153 -0.31600 0.22208
C 4.24932 -0.71608 -2.81440
H 4.18468 -1.49279 -3.59349
H 4.72637 0.16793 -3.27508
H 3.22561 -0.42505 -2.52769
C 6.48996 -1.63382 -2.14737
H 7.19086 -1.89903 -1.33887
H 6.94322 -0.78310 -2.68823
H 6.42964 -2.47499 -2.85035
C 0.04484 -1.37139 4.26135
C -0.89700 -2.52168 3.82676
H -0.86539 -2.72739 2.74642
H -1.93251 -2.23704 4.08845
H -0.67917 -3.46398 4.35281
C -0.56016 -0.02764 3.80360
H 0.08666 0.83636 4.01494
H -1.50267 0.12631 4.35998
H -0.80976 -0.02426 2.73083
C 0.13643 -1.37377 5.80438
H 0.62891 -2.27458 6.20411
H -0.89368 -1.36057 6.20587
H 0.64965 -0.48584 6.20327
C 3.19028 -0.61137 4.41149
C 4.47154 -0.63143 3.54418
H 4.96405 -1.61514 3.55194
H 5.18868 0.10241 3.95616
H 4.26579 -0.35699 2.49125
C 3.52629 -1.16713 5.80786
H 2.68384 -1.11898 6.51157
H 4.34609 -0.55890 6.23208
H 3.87839 -2.20989 5.76442
C 2.69904 0.85060 4.51324
H 3.52613 1.46589 4.91165
H 1.85001 0.97047 5.20324
H 2.42599 1.27295 3.53112
C -1.07305 1.52024 0.13318
H -1.32579 2.06208 1.05219
C -1.84513 0.34917 -0.11439
H -2.59171 0.11662 0.65459
C -1.33105 -0.92736 -0.75935
H -2.19021 -1.56834 -1.02579
H -0.79209 -0.72307 -1.69703
C 0.38492 1.71358 -0.23077
H 0.58566 1.37318 -1.25794
H 0.62713 2.79057 -0.20098
Rh -2.71433 2.03695 -1.20413
P -4.16181 2.57080 0.61338
P -1.82734 1.65767 -3.42551
N -3.92203 3.61426 -2.30150
C -5.13761 4.05441 -1.83943
C -5.86897 5.09680 -2.43681
H -6.81383 5.41573 -1.99075
C -5.36422 5.70959 -3.58776
H -5.91309 6.52078 -4.07492
C -4.15356 5.25278 -4.11037
H -3.73647 5.67038 -5.02974
C -3.46832 4.21466 -3.44855
S -5.92161 3.27858 -0.45307
S -1.95341 3.70450 -4.19566
C -3.69859 4.11675 1.73916

C	-3.33608	5.25560	0.76021
H	-4.21518	5.62650	0.20966
H	-2.93216	6.10303	1.34312
H	-2.56548	4.94530	0.03324
C	-4.85777	4.56259	2.65121
H	-5.02724	3.86570	3.48740
H	-4.59282	5.53942	3.09520
H	-5.80141	4.69946	2.09859
C	-2.45465	3.79860	2.60142
H	-1.54314	3.70386	1.99048
H	-2.28929	4.64839	3.28835
H	-2.56769	2.89676	3.22329
C	-5.11129	1.17154	1.61738
C	-5.35221	0.00231	0.63403
H	-4.42243	-0.43405	0.24013
H	-5.90135	-0.79464	1.16744
H	-5.97153	0.31600	-0.22208
C	-4.24932	0.71608	2.81440
H	-4.18468	1.49279	3.59349
H	-4.72637	-0.16793	3.27508
H	-3.22561	0.42505	2.52769
C	-6.48996	1.63382	2.14737
H	-7.19086	1.89903	1.33887
H	-6.94322	0.78310	2.68823
H	-6.42964	2.47499	2.85035
C	-0.04484	1.37139	-4.26135
C	0.89700	2.52168	-3.82676
H	0.86539	2.72739	-2.74642
H	1.93251	2.23704	-4.08845
H	0.67917	3.46398	-4.35281
C	0.56016	0.02764	-3.80360
H	-0.08666	-0.83636	-4.01494
H	1.50267	-0.12631	-4.35998
H	0.80976	0.02426	-2.73083
C	-0.13643	1.37377	-5.80438
H	-0.62891	2.27458	-6.20411
H	0.89368	1.36057	-6.20587
H	-0.64965	0.48584	-6.20327
C	-3.19028	0.61137	-4.41149
C	-4.47154	0.63143	-3.54418
H	-4.96405	1.61514	-3.55194
H	-5.18868	-0.10241	-3.95616
H	-4.26579	0.35699	-2.49125
C	-3.52629	1.16713	-5.80786
H	-2.68384	1.11898	-6.51157
H	-4.34609	0.55890	-6.23208
H	-3.87839	2.20989	-5.76442
C	-2.69904	-0.85060	-4.51324
H	-3.52613	-1.46589	-4.91165
H	-1.85001	-0.97047	-5.20324
H	-2.42599	-1.27295	-3.53112

8_S

SCF (BP86) Energy = -1334.69800849
Enthalpy 0K = -1333.970218
Enthalpy 298K = -1333.924338
Free Energy 298K = -1334.043299
Lowest Frequency = 19.7071 cm^{-1}
Second Frequency = 33.4344 cm^{-1}
SCF (BP86-D3BJ) Energy = -1334.953874
SCF (FB) Energy = -1334.738192
SCF (DFB) Energy = -1334.743732
SCF (BS2) Energy = -2779.803757

C	2.77723	4.47378	0.53422
H	3.75575	4.80718	0.90389
C	2.55570	4.57585	-0.79347
H	3.37011	4.98533	-1.40532
C	1.30712	4.16990	-1.55371
H	1.36854	4.56794	-2.58024
H	0.41148	4.63791	-1.09851
C	1.83056	3.93163	1.58907
H	0.84257	4.42711	1.50857
H	2.22045	4.19415	2.58672
C	0.08750	2.17041	-0.58287
H	-0.92474	2.49774	-0.85535
C	0.32309	2.04475	0.81133
H	-0.53629	2.26890	1.45478
C	1.62295	2.37623	1.51768
H	1.58802	1.96189	2.54115
H	2.49050	1.92364	1.01450
C	1.08803	2.61561	-1.62452
H	2.05542	2.11103	-1.49696
H	0.71189	2.33710	-2.62592
Rh	-0.15004	0.07093	0.00396
P	-2.47348	0.42004	0.03498
P	2.11474	-0.81381	-0.01451
N	-0.72977	-2.10089	-0.11708
C	-1.95690	-2.55807	0.29300
C	-2.32587	-3.91784	0.27618
H	-3.32843	-4.20603	0.60100
C	-1.40767	-4.86685	-0.17595
H	-1.66624	-5.92974	-0.19160
C	-0.15447	-4.43089	-0.61407
H	0.60167	-5.13391	-0.97044
C	0.14208	-3.05508	-0.58339
S	-3.21385	-1.45317	0.87496
S	1.71604	-2.59356	-1.23796
C	-3.29620	0.45825	-1.74933
C	-2.70302	-0.75120	-2.50727
H	-3.05321	-1.71222	-2.09701
H	-3.03782	-0.70001	-3.55923
H	-1.59935	-0.73988	-2.49935
C	-4.83249	0.34717	-1.72716
H	-5.31636	1.28167	-1.40427
H	-5.18021	0.14391	-2.75643
H	-5.18765	-0.48000	-1.09086
C	-2.87109	1.74897	-2.48597
H	-1.78280	1.78819	-2.65036
H	-3.35194	1.74975	-3.48120
H	-3.19097	2.67002	-1.97297
C	-3.38621	1.58274	1.33001
C	-3.21400	3.06015	0.90844
H	-3.74677	3.28794	-0.02867
H	-3.65411	3.70147	1.69337
H	-2.16084	3.36147	0.79403
C	-4.89594	1.27664	1.46565
H	-5.10277	0.22167	1.71229
H	-5.28802	1.88180	2.30346
H	-5.47240	1.54512	0.57037
C	-2.72904	1.32442	2.70846
H	-1.63323	1.42110	2.69943
H	-3.12943	2.06182	3.42733
H	-2.96993	0.31934	3.08942
C	3.75634	-0.35716	-1.03443
C	3.34847	-0.18635	-2.51844
H	2.47277	0.46677	-2.65148

```
H   4.19588  0.27024 -3.06113
H   3.12572 -1.14903 -3.00491
C   4.39471  0.95460 -0.52436
H   4.69210  0.90562  0.53327
H   5.31503  1.12977 -1.11104
H   3.75714  1.84083 -0.66375
C   4.81121 -1.48318 -0.93382
H   4.42389 -2.46967 -1.23656
H   5.64063 -1.23371 -1.62085
H   5.24280 -1.57078  0.07511
C   2.60014 -1.55265  1.76173
C   1.30363 -1.51965  2.60447
H   0.54887 -2.22999  2.23236
H   1.54840 -1.80775  3.64379
H   0.84267 -0.51674  2.62259
C   3.12507 -3.00068  1.70407
H   4.04459 -3.10344  1.10972
H   3.35754 -3.32279  2.73582
H   2.37638 -3.69960  1.30003
C   3.65589 -0.64807  2.43641
H   3.78652 -0.99228  3.47875
H   4.64225 -0.71174  1.95169
H   3.34957  0.40863  2.48107
```

A$_{S}$

SCF (BP86) Energy = -1022.64568109
Enthalpy 0K = -1022.097358
Enthalpy 298K = -1022.060202
Free Energy 298K = -1022.162234
Lowest Frequency = 22.3343 cm^{-1}
Second Frequency = 31.0707 cm^{-1}
SCF (BP86-D3BJ) Energy = -1022.835715
SCF (FB) Energy = -1022.68902
SCF (DFB) Energy = -1022.695228
SCF (BS2) Energy = -2467.682424

```
Rh  0.08685 -0.36747 -0.00475
P  -2.23619 -0.35789  0.09953
P   2.34778 -0.34018 -0.07858
N   0.08081  1.66604 -0.01826
C  -1.02534  2.41355  0.34439
C  -1.03512  3.81927  0.30479
H  -1.94878  4.35154  0.57985
C   0.11398  4.50811 -0.08755
H   0.12903  5.60136 -0.10854
C   1.24363  3.77407 -0.46287
H   2.16429  4.27290 -0.77479
C   1.19796  2.37099 -0.44613
S  -2.52990  1.65322  0.91585
S   2.67438  1.56527 -1.07022
C  -2.99684 -1.42114  1.52784
C  -1.92919 -1.41206  2.64681
H  -1.75885 -0.39763  3.04290
H  -2.27653 -2.05006  3.47999
H  -0.95974 -1.80644  2.29128
C  -4.32422 -0.86027  2.07870
H  -5.12803 -0.84371  1.32823
H  -4.66063 -1.50664  2.90993
H  -4.20579  0.15620  2.49083
C  -3.19529 -2.86444  1.01373
H  -2.27968 -3.27897  0.55665
H  -3.45556 -3.51167  1.87041
H  -4.01830 -2.94220  0.28550
C  -3.26733 -0.38338 -1.54067
C  -2.74816  0.80084 -2.38652
H  -1.65137  0.76677 -2.50879
H  -3.20316  0.74289 -3.39165
H  -3.02530  1.77350 -1.94886
C  -2.91946 -1.70353 -2.26908
H  -3.26869 -2.59838 -1.73161
H  -3.41189 -1.70042 -3.25829
H  -1.83302 -1.79622 -2.44076
C  -4.78585 -0.25299 -1.32573
H  -5.04552  0.64932 -0.74658
H  -5.28053 -0.16455 -2.31011
H  -5.21788 -1.13391 -0.82441
C   3.54176 -0.35997  1.43312
C   3.13354  0.84273  2.31225
H   2.07045  0.79692  2.60325
H   3.74038  0.82427  3.23528
H   3.32280  1.80484  1.80908
C   3.28476 -1.67054  2.21152
H   3.57053 -2.57189  1.64604
H   3.89646 -1.65881  3.13141
H   2.22826 -1.76160  2.51734
C   5.01363 -0.23079  0.99589
H   5.18575  0.68924  0.41156
H   5.64930 -0.16875  1.89779
H   5.35928 -1.09411  0.40722
C   2.51739 -1.82877 -1.30642
C   2.22538 -1.33015 -2.73629
H   3.05950 -0.73156 -3.13646
H   2.09132 -2.20563 -3.39789
H   1.30839 -0.71855 -2.77820
C   3.81811 -2.64662 -1.29310
H   4.06819 -3.05338 -0.30110
H   3.71074 -3.50508 -1.98165
H   4.67148 -2.04835 -1.65139
C   1.31239 -2.67048 -0.78438
H   1.11911 -3.53087 -1.45410
H   1.45556 -3.06226  0.23444
H   0.31471 -2.10553 -0.86837
```

B$_{S\text{-}Ar}$

SCF (BP86) Energy = -1645.59264327
Enthalpy 0K = -1644.716481
Enthalpy 298K = -1644.659786
Free Energy 298K = -1644.805370
Lowest Frequency = 2.9759 cm^{-1}
Second Frequency = 12.8255 cm^{-1}
SCF (BP86-D3BJ) Energy = -1645.890171
SCF (FB) Energy = -1645.631464
SCF (DFB) Energy = -1645.636906
SCF (BS2) Energy = -3090.768764

```
Rh -1.11733  0.01974 -0.43859
P  -1.40795  2.37507 -0.36554
P  -1.33703 -2.31930 -0.57983
N  -2.43952 -0.11315  1.28743
C  -3.14108  0.97911  1.74666
C  -3.87948  0.96889  2.94301
H  -4.38146  1.88087  3.27387
C  -3.95589 -0.21362  3.68621
H  -4.52595 -0.24748  4.61932
C  -3.30920 -1.35442  3.20891
H  -3.37771 -2.30863  3.73663
```

C -2.56550 -1.27466 2.01470
S -3.25055 2.48622 0.80739
S -1.80417 -2.79810 1.50794
C -2.05967 3.20084 -2.00550
C -2.90214 2.12073 -2.72023
H -3.78386 1.83254 -2.12363
H -3.26717 2.53255 -3.67888
H -2.31501 1.21365 -2.93475
C -2.95119 4.43861 -1.76303
H -2.42204 5.26341 -1.26584
H -3.29561 4.81175 -2.74496
H -3.85512 4.19622 -1.17884
C -0.84680 3.58788 -2.88117
H -0.12758 2.75996 -2.99650
H -1.21163 3.85900 -3.88845
H -0.31157 4.46441 -2.48317
C -0.20553 3.53615 0.63867
C -0.08110 2.88247 2.03219
H 0.23259 1.82751 1.96219
H 0.68964 3.42510 2.60817
H -1.02361 2.93706 2.60101
C 1.17798 3.53470 -0.04951
H 1.16066 4.00508 -1.04391
H 1.87296 4.12216 0.57805
H 1.59726 2.52217 -0.14517
C -0.72652 4.97794 0.78653
H -1.75320 5.01720 1.18764
H -0.07398 5.51259 1.50095
H -0.68949 5.53613 -0.16229
C -2.98126 -2.76955 -1.54646
C -3.95147 -1.59303 -1.29156
H -3.50441 -0.62196 -1.57143
H -4.85822 -1.74283 -1.90611
H -4.27104 -1.53898 -0.23833
C -2.64324 -2.82721 -3.05308
H -2.04947 -3.71625 -3.31795
H -3.58802 -2.88561 -3.62372
H -2.10611 -1.92631 -3.39656
C -3.66025 -4.07504 -1.08968
H -3.86414 -4.07572 -0.00650
H -4.63153 -4.15924 -1.61086
H -3.07947 -4.97476 -1.33483
C 0.07126 -3.63224 -0.90160
C 1.12233 -3.48809 0.21970
H 0.74659 -3.83672 1.19476
H 1.99285 -4.11490 -0.04634
H 1.48096 -2.45396 0.33420
C -0.45901 -5.08102 -0.91518
H -1.09094 -5.29162 -1.79175
H 0.40918 -5.76299 -0.97034
H -1.02100 -5.34144 -0.00245
C 0.73599 -3.30289 -2.26025
H 1.53211 -4.04840 -2.43911
H 0.03655 -3.36423 -3.10769
H 1.19970 -2.30568 -2.25943
C 0.10246 0.16796 -2.14264
H -0.08363 0.21172 -3.21049
C 0.90589 0.10099 -1.14884
C 2.18874 0.05482 -0.48580
C 3.35565 0.18634 -1.28037
C 2.31209 -0.12494 0.91143
H 3.24183 0.32851 -2.35963
H 1.39436 -0.22706 1.50049
C 4.63387 0.13845 -0.69824
C 3.57902 -0.18535 1.52483
C 4.71609 -0.04907 0.70012
H 5.70359 -0.09142 1.16135
C 3.68825 -0.40562 3.05076
C 3.01646 -1.75478 3.42237
C 2.96351 0.74758 3.79363
H 3.41702 1.72359 3.55019
H 1.89247 0.79443 3.53338
H 3.03495 0.60133 4.88547
H 1.94965 -1.77387 3.14089
H 3.07878 -1.92401 4.51152
H 3.51689 -2.59888 2.91788
C 5.88562 0.28868 -1.59195
C 5.83828 1.66403 -2.31006
C 5.89731 -0.84578 -2.65133
H 5.93623 -1.83799 -2.17011
H 5.00364 -0.81670 -3.29743
H 6.78279 -0.74796 -3.30297
H 4.94229 1.76736 -2.94531
H 6.72276 1.78442 -2.95931
H 5.83511 2.49256 -1.58133
C 5.15402 -0.44606 3.53714
H 5.72177 -1.27006 3.07190
H 5.68408 0.50016 3.33305
H 5.17545 -0.60669 4.62807
C 7.19887 0.20937 -0.78247
H 7.26937 1.01191 -0.02811
H 7.31123 -0.76156 -0.26986
H 8.05827 0.32259 -1.46428

10$_{S-Ar}$

SCF (BP86) Energy = -1645.60578491
Enthalpy 0K = -1644.729252
Enthalpy 298K = -1644.672617
Free Energy 298K = -1644.818899
Lowest Frequency = 8.1052 cm^{-1}
Second Frequency = 11.1223 cm^{-1}
SCF (BP86-D3BJ) Energy = -1645.893289
SCF (FB) Energy = -1645.645613
SCF (DFB) Energy = -1645.65129
SCF (BS2) Energy = -3090.782308

Rh -1.47069 -0.02732 -0.19928
P -1.59295 2.30962 -0.34536
P -1.49362 -2.37040 -0.06711
N -3.60411 -0.03752 0.32944
C -4.28192 1.12400 0.62260
C -5.64556 1.14780 0.97023
H -6.13455 2.10308 1.17482
C -6.34844 -0.05661 1.04748
H -7.40641 -0.06411 1.32598
C -5.68312 -1.25128 0.76263
H -6.19762 -2.21338 0.81845
C -4.32524 -1.20817 0.39522
S -3.50050 2.72210 0.63662
S -3.63698 -2.79183 -0.03173
C -1.85320 2.99900 -2.14226
C -3.06641 2.23259 -2.71551
H -4.00538 2.49568 -2.20194
H -3.18014 2.50186 -3.78105
H -2.92235 1.13922 -2.65647
C -2.11877 4.51509 -2.18109
H -1.23228 5.10350 -1.89555

H	-2.37904	4.80180	-3.21627
H	-2.96560	4.80801	-1.53771
C	-0.60086	2.64042	-2.97486
H	-0.42450	1.55299	-2.98816
H	-0.77171	2.96866	-4.01620
H	0.31327	3.13729	-2.61661
C	-0.43889	3.39924	0.77025
C	-0.26546	2.59398	2.07746
H	0.16700	1.59730	1.88958
H	0.41650	3.14826	2.74739
H	-1.22312	2.46143	2.60846
C	0.92451	3.56788	0.06462
H	0.85533	4.22067	-0.82050
H	1.62292	4.05199	0.77063
H	1.37069	2.60597	-0.23460
C	-1.03797	4.78344	1.10198
H	-1.99121	4.70791	1.65166
H	-0.32829	5.31535	1.76137
H	-1.19206	5.41093	0.21223
C	-0.99896	-3.43206	-1.61656
C	-1.39376	-2.57882	-2.84278
H	-0.85617	-1.61715	-2.86427
H	-1.13978	-3.13905	-3.76098
H	-2.47676	-2.37046	-2.86606
C	0.52764	-3.66924	-1.60388
H	0.82981	-4.38360	-0.82148
H	0.81660	-4.11121	-2.57475
H	1.09979	-2.73680	-1.47464
C	-1.73856	-4.78692	-1.67876
H	-2.82999	-4.66443	-1.77753
H	-1.39238	-5.32664	-2.57890
H	-1.53223	-5.43050	-0.81082
C	-0.86184	-3.09214	1.61789
C	-1.63043	-2.32014	2.71406
H	-2.70773	-2.55364	2.71012
H	-1.22752	-2.61431	3.69990
H	-1.50307	-1.22798	2.60857
C	-1.10505	-4.60525	1.76750
H	-0.49309	-5.19979	1.07054
H	-0.81955	-4.91009	2.79079
H	-2.16632	-4.87338	1.63010
C	0.64373	-2.76252	1.73511
H	0.98566	-3.05403	2.74457
H	1.26245	-3.30799	1.00755
H	0.83870	-1.68476	1.61249
C	0.28824	-0.02893	-0.71658
H	1.52337	-0.14451	-2.31449
C	1.52135	-0.05242	-1.21249
C	2.85618	0.01047	-0.57903
C	3.03564	0.23558	0.80449
C	3.99104	-0.16312	-1.39972
H	2.15320	0.38437	1.43632
H	3.84311	-0.33320	-2.47226
C	4.32271	0.28068	1.37192
C	5.29383	-0.12477	-0.86902
C	5.43160	0.09638	0.51693
H	6.43432	0.12950	0.94454
C	6.50655	-0.32157	-1.80556
C	6.49900	0.78594	-2.89315
C	6.40735	-1.71286	-2.48673
H	6.41517	-2.52279	-1.73739
H	5.48636	-1.81448	-3.08552
H	7.26453	-1.86681	-3.16509
H	5.58010	0.75947	-3.50301
H	7.35634	0.65483	-3.57608
H	6.57455	1.78855	-2.43875
C	4.48669	0.53932	2.88750
C	3.72399	-0.55049	3.68637
C	3.90015	1.93402	3.23296
H	4.43045	2.73234	2.68621
H	2.82900	2.00051	2.97640
H	4.00013	2.13524	4.31399
H	2.64539	-0.55227	3.45371
H	3.83077	-0.37405	4.77100
H	4.12148	-1.55531	3.46324
C	7.85145	-0.24810	-1.04945
H	8.00049	0.73266	-0.56574
H	7.93533	-1.03281	-0.27788
H	8.68224	-0.39451	-1.76000
C	5.96533	0.51472	3.33471
H	6.44045	-0.46117	3.13560
H	6.56114	1.29888	2.83706
H	6.02498	0.69604	4.42114

HCCNMe$_2$

SCF (BP86) Energy = -211.293321810
Enthalpy 0K = -211.195618
Enthalpy 298K = -211.187833
Free Energy 298K = -211.225131
Lowest Frequency = 158.2611 cm^{-1}
Second Frequency = 180.2648 cm^{-1}
SCF (BP86-D3BJ) Energy = -211.3057554
SCF (FB) Energy = -211.2967408
SCF (DFB) Energy = -211.2974719
SCF (BS2) Energy = -211.3540515

C	0.99442	-0.00148	-0.08578
C	2.20683	-0.00057	0.08839
H	3.27750	-0.00104	0.13008
N	-0.34545	-0.00010	-0.27857
C	-1.05127	1.23987	0.07380
H	-2.05164	1.22815	-0.39171
H	-1.17214	1.36681	1.17046
H	-0.48839	2.09905	-0.32011
C	-1.05404	-1.23844	0.07394
H	-2.05437	-1.22456	-0.39161
H	-0.49312	-2.09895	-0.31987
H	-1.17525	-1.36500	1.17061

TS(C-10)$_{C\text{-}NMe2}$

SCF (BP86) Energy = -1292.13454000
Enthalpy 0K = -1291.436055
Enthalpy 298K = -1291.392318
Free Energy 298K = -1291.508778
Lowest Frequency = -754.6174 cm^{-1}
Second Frequency = 15.8377 cm^{-1}
SCF (BP86-D3BJ) Energy = -1292.349423
SCF (FB) Energy = -1292.175404
SCF (DFB) Energy = -1292.181271
SCF (BS2) Energy = -1961.73039

Rh	0.00568	-0.03306	0.03108
P	2.32323	-0.28015	-0.06863
P	-2.31099	-0.32647	0.09867
N	0.02177	-2.16467	-0.05470
C	1.17007	-2.84386	-0.38388

C	1.20592	-4.24611	-0.43435
H	2.14118	-4.74749	-0.69816
C	0.04615	-4.98239	-0.17026
H	0.05577	-6.07546	-0.21459
C	-1.12657	-4.29032	0.14994
H	-2.05356	-4.82766	0.36824
C	-1.11537	-2.88804	0.21293
C	2.39094	-2.04040	-0.75194
H	3.31459	-2.57611	-0.47399
H	2.41206	-1.92201	-1.85123
C	-2.35532	-2.13882	0.63072
H	-3.26467	-2.66311	0.29069
H	-2.39728	-2.11814	1.73553
C	3.19947	-0.38829	1.65387
C	2.49759	-1.53605	2.41765
H	2.69633	-2.52586	1.97319
H	2.87976	-1.56012	3.45441
H	1.40532	-1.38264	2.46027
C	4.71263	-0.67013	1.57710
H	5.27625	0.17339	1.14746
H	5.10044	-0.83173	2.59973
H	4.94681	-1.57839	0.99572
C	2.93365	0.93152	2.41302
H	1.85135	1.12961	2.49009
H	3.34128	0.84814	3.43703
H	3.41336	1.80038	1.93595
C	3.36341	0.69583	-1.37761
C	2.43960	0.87038	-2.60663
H	1.51040	1.40318	-2.34877
H	2.97626	1.45193	-3.37862
H	2.15850	-0.09431	-3.06405
C	3.71807	2.08855	-0.81392
H	4.45741	2.03437	0.00154
H	4.16305	2.70147	-1.61884
H	2.82232	2.61510	-0.44489
C	4.65102	-0.03854	-1.81368
H	4.44418	-1.02059	-2.27041
H	5.16086	0.56968	-2.58317
H	5.36285	-0.18487	-0.98818
C	-3.38146	0.51448	1.47720
C	-2.47680	0.58674	2.73037
H	-1.54664	1.14512	2.53525
H	-3.02750	1.09293	3.54433
H	-2.19016	-0.41184	3.10366
C	-3.75091	1.94773	1.03788
H	-4.48238	1.95529	0.21362
H	-4.21307	2.48014	1.88916
H	-2.86438	2.52310	0.72241
C	-4.66430	-0.27134	1.83030
H	-4.45043	-1.28925	2.19614
H	-5.18967	0.25718	2.64684
H	-5.36543	-0.34848	0.98667
C	-3.17305	-0.30174	-1.63440
C	-2.43201	-1.35744	-2.48865
H	-2.60728	-2.38770	-2.13561
H	-2.80397	-1.29959	-3.52773
H	-1.34417	-1.17113	-2.50366
C	-4.67800	-0.63091	-1.59919
H	-5.26931	0.15671	-1.10531
H	-5.05195	-0.71439	-2.63621
H	-4.89146	-1.59211	-1.10079
C	-2.93784	1.08794	-2.26907
H	-3.31941	1.08029	-3.30643
H	-3.46326	1.89260	-1.73095
H	-1.86319	1.33305	-2.29914
C	0.00366	1.87190	-0.00681
H	-0.27701	2.60729	1.14927
C	-0.00996	3.16592	0.04749
N	-0.00031	4.48555	-0.24146
C	1.09652	5.30980	0.29752
H	1.19149	6.22027	-0.31632
H	0.91450	5.61042	1.34874
H	2.03881	4.74669	0.24230
C	-1.29601	5.17877	-0.36315
H	-1.71700	5.45670	0.62384
H	-1.15105	6.09865	-0.95287
H	-2.00708	4.52888	-0.89270

The full Gaussian reference (g09, D.01):

Frisch, M. J.; Trucks, G. W.; Schlegel, H. B.; Scuseria, G. E.; Robb, M. A.; Cheeseman, J. R.; Scalmani, G.; Barone, V.; Mennucci, B.; Petersson, G. A.; Nakatsuji, H.; Caricato, M.; Li, X.; Hratchian, H. P.; Izmaylov, A. F.; Bloino, J.; Zheng, G.; Sonnenberg, J. L.; Hada, M.; Ehara, M.; Toyota, K.; Fukuda, R.; Hasegawa, J.; Ishida, M.; Nakajima, T.; Honda, Y.; Kitao, O.; Nakai, H.; Vreven, T.; Montgomery, J. A., Jr.; Peralta, J. E.; Ogliaro, F.; Bearpark, M.; Heyd, J. J.; Brothers, E.; Kudin, K. N.; Staroverov, V. N.; Kobayashi, R.; Normand, J.; Raghavachari, K.; Rendell, A.; Burant, J. C.; Iyengar, S. S.; Tomasi, J.; Cossi, M.; Rega, N.; Millam, J. M.; Klene, M.; Knox, J. E.; Cross, J. B.; Bakken, V.; Adamo, C.; Jaramillo, J.; Gomperts, R.; Stratmann, R. E.; Yazyev, O.; Austin, A. J.; Cammi, R.; Pomelli, C.; Ochterski, J. W.; Martin, R. L.; Morokuma, K.; Zakrzewski, V. G.; Voth, G. A.; Salvador, P.; Dannenberg, J. J.; Dapprich, S.; Daniels, A. D.; Farkas, O.; Foresman, J. B.; Ortiz, J. V.; Cioslowski, J.; Fox, D. J. Gaussian 09 (Revision D.01); Gaussian Inc.: Wallingford, CT, 2009.
